# Supplementary material for: Discovery of Novel Thiosemicarbazides Containing 1,3,5-Triazines Derivatives as Potential Synergists against Fluconazole-Resistant Candida albicans
Source: Pharmaceutics. 2022 Oct 29;14(11):2334. doi: 10.3390/pharmaceutics14112334 (PMC9693882; doi:10.3390/pharmaceutics14112334)
Supplement: Supplementary file 1 [file pharmaceutics-14-02334-s001.zip › pharmaceutics-1898767-supplementary.pdf]

Discovery of Novel Thiosemicarbazides Containing 1,3,5-Triazines Derivatives as Potential Synergists against Fluconazole-Resistant *Candida albicans*

Fei Xie [1,†], Yumeng Hao [1,†], Jiacun Liu [2,†], Junhe Bao [1], Tingjunhong Ni [3], Yu Liu [2], Xiaochen Chi [4], Ting Wang [1], Shichong Yu [1], Yongsheng Jin [1], Liping Li [3], Dazhi Zhang [1,*] and Lan Yan [2,*]

[1] Department of Organic Chemistry, School of Pharmacy, Naval Medical University, No. 325 Guohe Road, Shanghai 200433, China
[2] Center of New Drug Research, School of Pharmacy, Naval Medical University, No. 325 Guohe Road, Shanghai 200433, China
[3] Department of Pharmacy, Shanghai Tenth People's Hospital, School of Medicine, Tongji University, No. 1239 Siping Road, Shanghai 200092, China
[4] School of Traditional Chinese Materia Medica, Shenyang Pharmaceutical University, No. 103 Wenhua Road, Shenyang 110016, China
* Correspondence: zdzhang_yjhx@smmu.edu.cn (D.Z.); ylansmmu@sina.com (L.Y.)
† These authors contributed equally to this work.

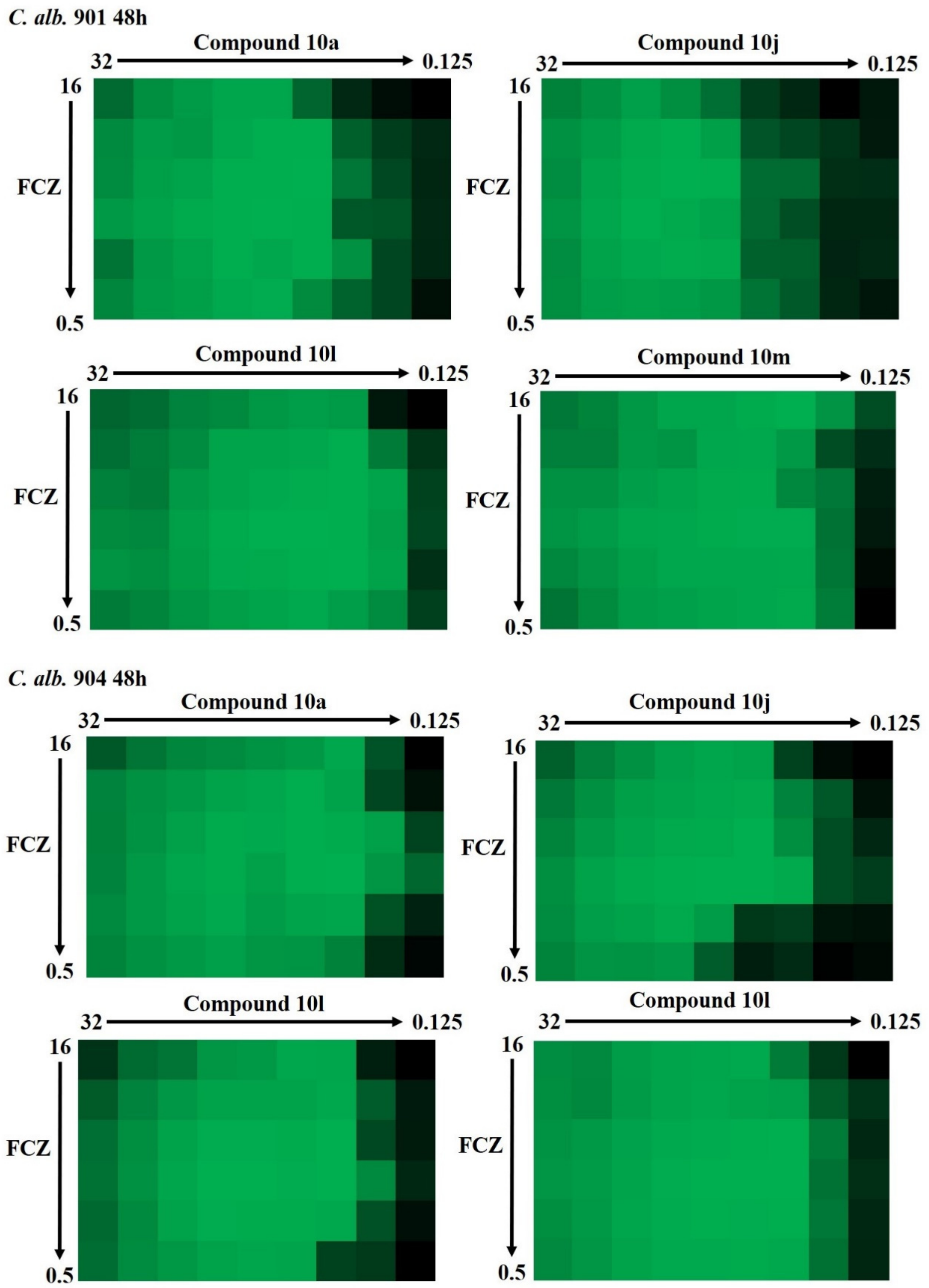

Figure S1. Checkerboard microdilution assay of FCZ with the target compounds **10a**, **10j**, **10l** and **10m**. The *in vitro* synergistic antifungal activity was shown in heat map. The color changes from green to black were used to express the relative growth of the fungi, if the color was greener, the inhibition was stronger. Abbreviations: *C. alb.*, Candida albicans; FCZ, fluconazole.

Table S1. Antifungal activity of compound **10l** against 8 Cryptococcus strains (MIC_{80}, μg/mL, 72h)

Species	Strains	**FCZ**	**8**	**10l**
C. neoformans.	HN-15	16.0	16.0	>64.0
C. neoformans.	HN-17	8.0	16.0	0.25
C. neoformans.	HN-19	16.0	16.0	0.5
C. neoformans.	HN-20	8.0	32.0	0.5
C. neoformans.	HN-68	4.0	16.0	≤0.125
C. neoformans.	BJ-3	8.0	32.0	≤0.125
C. neoformans.	BJ-72	16.0	16.0	0.25
C. neoformans.	BJ-95	4.0	16.0	≤0.125

Abbreviations: *C. neoformans.*, Cryptococcus neoformans; FCZ, fluconazole.

The characterization data of the compounds are given below:

1. *2-(4-((4-((cyclohexylmethyl)amino)-6-((4-fluorobenzyl)amino)-1,3,5-triazin-2-yl)amino)phenyl)-N-(2-fluorophenyl)acetamide* (**8b**)
White solid, yield: 65.2%; ^{1}H NMR (300 MHz, DMSO-d_6) δ 9.88 (s, 1H), 8.85 (dd, J = 45.5, 18.0 Hz, 1H), 7.85 (t, J = 8.8 Hz, 1H), 7.74 – 7.54 (m, 2H), 7.52 – 7.06 (m, 10H), 6.87 (t, J = 33.9 Hz, 1H), 4.44 (d, J = 5.1 Hz, 2H), 3.63 (s, 2H), 3.14 – 2.98 (m, 2H), 1.78 – 1.38 (m, 6H), 1.15 – 1.07 (m, 3H), 0.96 – 0.67 (m, 2H). ^{13}C NMR (75 MHz, DMSO-d_6) δ 170.34, 166.13, 165.99, 164.47, 163.06, 159.85, 155.74, 152.49, 139.66, 137.31, 129.40, 129.29, 128.94, 126.72, 126.57, 125.74, 125.64, 124.77, 124.72, 124.62, 120.24, 119.85, 116.03, 115.77, 115.40, 115.12, 46.98, 46.63, 43.18, 42.85, 42.49, 37.94, 31.07, 26.64, 25.92. MS (ESI) *m/z*: 558.0[M+H]$^+$, $C_{31}H_{33}F_2N_7O$. Purity: 97.4% (LC-MS).
2. *2-(4-((4-((cyclohexylmethyl)amino)-6-((4-fluorobenzyl)amino)-1,3,5-triazin-2-yl)amino)phenyl)-N-(3-fluorophenyl)acetamide* (**8c**)
White solid, yield: 60.1%; ^{1}H NMR (300 MHz, DMSO-d_6) δ 10.31 (s, 1H), 8.95 – 8.65 (m, 1H), 7.80 – 7.54 (m, 3H), 7.46 – 7.04 (m, 9H), 7.02 – 6.65 (m, 2H), 4.43 (s, 2H), 3.55 (s, 2H), 3.08 (s, 2H), 1.78 – 1.30 (m, 6H), 1.29 – 1.00 (d, J = 24.4 Hz, 3H), 0.97 – 0.66 (m, 2H). ^{13}C NMR (75 MHz, DMSO-d_6) δ 170.32, 166.46, 166.28, 166.17, 164.55, 164.19, 163.05, 161.00, 159.84, 141.54, 141.39, 139.81, 137.33, 130.85, 130.73, 129.41, 129.27, 128.65, 120.22, 119.86, 115.38, 115.27, 115.10, 110.18, 109.90, 106.46, 106.11, 46.93, 46.61, 43.22, 37.91, 31.07, 26.64, 25.92. MS (ESI) *m/z*: 558.0[M+H]$^+$, $C_{31}H_{33}F_2N_7O$. Purity: 98.8% (LC-MS).
3. *2-(4-((4-((cyclohexylmethyl)amino)-6-((4-fluorobenzyl)amino)-1,3,5-triazin-2-yl)amino)phenyl)-N-(4-fluorophenyl)acetamide* (**8d**)
White solid, yield: 64.3%; ^{1}H NMR (300 MHz, DMSO-d_6) δ 10.15 (s, 1H), 8.97 – 8.64 (m, 1H), 7.80 – 7.54 (m, 4H), 7.47 – 7.02 (m, 9H), 6.83 (t, J = 34.5 Hz, 1H), 4.43 (s, 2H), 3.53 (s, 2H), 3.17 – 2.96 (m, 2H), 1.78 – 1.30 (m, 6H), 1.22 – 1.01 (m, 3H), 0.98 – 0.67 (m, 2H). ^{13}C NMR (75 MHz, DMSO-d_6) δ 169.81, 166.41, 166.29, 166.12, 164.55, 163.03, 159.96, 159.85, 156.78, 156.51, 139.82, 137.35, 136.16, 129.41, 129.23, 128.90, 121.32, 121.21, 119.86, 115.84, 115.55, 115.39, 115.11, 47.07, 46.61, 43.12, 37.89, 31.06, 26.64, 25.92. MS (ESI) *m/z*: 558.0[M+H]$^+$, $C_{31}H_{33}F_2N_7O$. Purity: 99.1% (LC-MS).
4. *2-(4-((4-((cyclohexylmethyl)amino)-6-((4-fluorobenzyl)amino)-1,3,5-triazin-2-yl)amino)phenyl)-N-(4-methoxyphenyl)acetamide* (**8e**)
White solid, yield: 54.6%; ^{1}H NMR (300 MHz, DMSO-d_6) δ 9.95 (s, 1H), 8.91 – 8.71 (m, 1H), 7.79 – 7.56 (m, 2H), 7.49 (d, J = 8.9 Hz, 2H), 7.42 – 7.05 (m, 7H), 7.03 – 6.65 (m, 3H), 4.44 (d, J = 5.4 Hz, 2H), 3.70 (s, 3H), 3.50 (s, 2H), 3.10 – 3.02 (m, 2H), 1.78 – 1.34 (m, 6H), 1.38 – 1.06 (m, 3H), 0.96 – 0.68 (m, 2H). ^{13}C NMR (75 MHz, DMSO-d_6) δ 169.39, 166.38, 166.29, 166.20, 166.16, 164.58, 163.06, 159.86, 155.60, 139.65, 137.37, 132.93, 129.41, 129.30, 129.19, 121.10, 120.30, 120.23, 120.11, 119.92, 119.83, 115.39, 115.11, 114.28, 55.61, 46.67, 43.12, 37.88, 31.07, 26.64, 25.93. MS (ESI) *m/z*: 570.0[M+H]$^+$, $C_{32}H_{36}FN_7O_2$. Purity: 98.8% (LC-MS).
5. *2-(4-((4-((cyclohexylmethyl)amino)-6-((4-fluorobenzyl)amino)-1,3,5-triazin-2-yl)amino)phenyl)-N-(4-(2-methoxyethoxy)phenyl)acetamide* (**8f**)
White solid, yield: 48.7%; ^{1}H NMR (300 MHz, DMSO-d_6) δ 9.95 (s, 1H), 8.97 – 8.65 (m, 1H), 7.77 – 7.54 (m, 2H), 7.48 (d, J = 8.9 Hz, 2H), 7.22 (dt, J = 17.1, 8.1 Hz, 7H), 7.03 – 6.64 (m, 3H), 4.44 (d, J = 6.2 Hz, 2H), 4.12 – 3.92 (m, 2H), 3.70 – 3.56 (m, 2H), 3.49 (s, 2H), 3.29 (s,

3H), 3.10 – 3.02 (m, 2H), 1.73 – 1.59 (m, 6H), 1.19 – 1.07 (m, 3H), 0.93 – 0.75 (m, 2H). ^{13}C NMR (75 MHz, DMSO-d_6) δ 169.40, 166.30, 164.58, 163.06, 159.86, 154.79, 139.66, 137.37, 133.02, 129.41, 129.31, 129.18, 121.08, 119.84, 115.38, 115.10, 114.90, 70.90, 67.48, 58.61, 46.96, 46.64, 43.14, 37.92, 31.07, 26.65, 25.92. MS (ESI) *m/z*: 614.0[M+H]$^+$, $C_{34}H_{40}FN_7O_3$. Purity: 97.5% (LC-MS).

6. *2-(4-((4-((cyclohexylmethyl)amino)-6-((4-fluorobenzyl)amino)-1,3,5-triazin-2-yl)amino)phenyl)-N-(4-fluorobenzyl)acetamide* (**8g**)
   White solid, yield: 66.4%; ^{1}H NMR (300 MHz, DMSO-d_6) δ 8.96 – 8.65 (m, 1H), 8.47 (s, 1H), 7.78 – 7.52 (m, 2H), 7.46 – 7.21 (m, 5H), 7.20 – 7.02 (m, 6H), 6.84 (t, *J* = 35.7 Hz, 1H), 4.44 (s, 2H), 4.24 (d, *J* = 5.3 Hz, 2H), 3.38 (s, 2H), 3.13 – 2.96 (m, 2H), 1.80 – 1.33 (m, 6H), 1.26 – 1.00 (m, 3H), 0.98 – 0.67 (m, 2H). ^{13}C NMR (75 MHz, DMSO-d_6) δ 170.96, 166.42, 166.29, 166.18, 164.56, 163.21, 163.06, 160.00, 159.86, 139.55, 137.57, 137.38, 136.24, 136.20, 129.67, 129.56, 129.41, 129.35, 129.17, 120.16, 119.74, 115.55, 115.39, 115.27, 115.10, 46.95, 46.66, 43.27, 43.12, 42.96, 42.24, 41.95, 38.07, 37.91, 31.07, 26.65, 25.93. MS (ESI) *m/z*: 572.0[M+H]$^+$, $C_{32}H_{35}F_2N_7O$. Purity: 97.3% (LC-MS).
7. *2-(4-((4-((cyclohexylmethyl)amino)-6-((4-fluorobenzyl)amino)-1,3,5-triazin-2-yl)amino)phenyl)-N-(4-methylbenzyl)acetamide* (**8h**)
   White solid, yield: 62.8%; ^{1}H NMR (300 MHz, DMSO-d_6) δ 8.92 – 8.71 (m, 1H), 8.43 (t, *J* = 5.8 Hz, 1H), 7.78 – 7.53 (m, 2H), 7.45 – 7.03 (m, 11H), 6.86 (t, *J* = 35.4 Hz, 1H), 4.45 (d, *J* = 5.4 Hz, 2H), 4.21 (d, *J* = 5.8 Hz, 2H), 3.38 (s, 2H), 3.15 – 2.99 (m, 2H), 2.26 (s, 3H), 1.77 – 1.50 (m, 6H), 1.17 – 1.14 (m, 3H), 0.98 – 0.73 (m, 2H). ^{13}C NMR (75 MHz, DMSO-d_6) δ 170.85, 166.44, 166.31, 166.19, 164.57, 163.07, 159.87, 139.55, 137.37, 136.95, 136.22, 129.46, 129.24, 129.17, 127.66, 120.16, 120.03, 119.96, 119.71, 115.39, 115.11, 46.96, 46.63, 43.23, 42.97, 42.40, 42.27, 37.94, 31.08, 26.66, 25.93, 21.10. MS (ESI) *m/z*: 568.0[M+H]$^+$, $C_{33}H_{38}FN_7O$. Purity: 97.6% (LC-MS).
8. *2-(4-((4-((cyclohexylmethyl)amino)-6-((4-fluorobenzyl)amino)-1,3,5-triazin-2-yl)amino)phenyl)-N-(furan-2-ylmethyl)acetamide* (**8i**)
   White solid, yield: 70.1%; ^{1}H NMR (300 MHz, DMSO-d_6) δ 9.00 – 8.42 (m, 1H), 8.44 (t, *J* = 5.1 Hz, 1H), 7.69 – 7.56 (m, 3H), 7.40 – 7.09 (m, 7H), 6.84 (t, *J* = 34.6 Hz, 1H), 6.37 (s, 1H), 6.19 (d, *J* = 2.3 Hz, 1H), 4.44 (d, *J* = 4.3 Hz, 2H), 4.24 (d, *J* = 5.4 Hz, 2H), 3.35 (s, 2H), 3.18 – 2.94 (m, 2H), 1.74 – 1.59(m, 6H), 1.19 – 1.07 (m, 3H), 0.94 –0.76 (m, 2H). ^{13}C NMR (75 MHz, DMSO-d_6) δ 170.80, 166.28, 166.15, 164.54, 163.07, 159.86, 152.81, 142.51, 139.52, 137.35, 129.42, 129.30, 129.17, 120.15, 119.96, 119.71, 115.39, 115.11, 110.87, 107.16, 46.95, 46.65, 43.22, 42.97, 42.06, 37.92, 36.05, 31.07, 26.66, 25.93. MS (ESI) *m/z*: 544.0[M+H]$^+$, $C_{30}H_{34}FN_7O_2$. Purity: 97.2% (LC-MS).
9. *2-(4-((4-((cyclohexylmethyl)amino)-6-((4-fluorobenzyl)amino)-1,3,5-triazin-2-yl)amino)phenyl)-N-((5-phenylfuran-2-yl)methyl)acetamide* (**8g**)
   White solid, yield: 62.4%; ^{1}H NMR (300 MHz, DMSO-d_6) δ 8.98 – 8.66 (m, 1H), 8.52 (s, 1H), 7.80 – 7.53 (m, 4H), 7.49 – 7.02 (m, 10H), 7.00 – 6.65 (m, 2H), 6.30 (d, *J* = 2.6 Hz, 1H), 4.43 (s, 2H), 4.32 (d, *J* = 4.7 Hz, 2H), 3.39 (s, 2H), 3.14 – 2.97 (m, 2H), 1.81 – 1.34 (m, 6H), 1.29 – 1.01 (m, 3H), 0.98 – 0.68 (m, 2H). ^{13}C NMR (75 MHz, DMSO-d_6) δ 170.92, 166.42, 166.31, 166.18, 164.58, 163.06, 159.86, 152.75, 152.59, 139.59, 137.37, 130.78, 129.42, 129.28, 129.17, 127.71, 123.65, 120.13, 119.75, 115.38, 115.11, 109.37, 106.92, 46.95, 46.64, 43.21, 43.00, 42.12, 37.94, 36.28, 31.07, 26.65, 25.92. MS (ESI) *m/z*: 620.0[M+H]$^+$, $C_{36}H_{38}FN_7O_2$. Purity:

97.4% (LC-MS).

10. *2-(2-(4-((4-((cyclohexylmethyl)amino)-6-((4-fluorobenzyl)amino)-1,3,5-triazin-2-yl)amino)phenyl)acetyl)-N-(2-chlorophenyl)hydrazine-1-carboxamide* (**9a**)
White solid, yield: 80.5%; ^{1}H NMR (300 MHz, DMSO-*d*$_6$) δ 10.04 (s, 1H), 8.96 – 8.69 (m, 2H), 8.19 (s, 1H), 8.09 (d, *J* = 8.1 Hz, 1H), 7.80 – 7.56 (m, 2H), 7.47 – 7.22 (m, 5H), 7.14 (s, 4H), 7.01 (t, *J* = 7.4 Hz, 1H), 6.79 (d, *J* = 35.0 Hz, 1H), 4.45 (s, 2H), 3.54 – 3.43 (m, 2H), 3.10 (s, 2H), 1.81 – 1.48 (m, 6H), 1.25 – 1.07 (s, 3H), 0.96 – 0.71 (m, 2H). ^{13}C NMR (75 MHz, DMSO-*d*$_6$) δ 170.81, 166.43, 166.29, 166.16, 164.56, 163.07, 162.77, 159.87, 155.24, 139.70, 137.34, 136.22, 129.64, 129.41, 129.24, 128.53, 128.05, 123.87, 122.43, 121.54, 120.06, 119.67, 115.40, 115.12, 46.92, 46.67, 43.18, 42.97, 37.96, 36.24, 31.08, 26.65, 25.93. MS (ESI) *m/z*: 632.0[M+H]$^+$, $C_{32}H_{33}ClFN_9O_2$. Purity: 97.5% (LC-MS).

11. *2-(2-(4-((4-((cyclohexylmethyl)amino)-6-((4-fluorobenzyl)amino)-1,3,5-triazin-2-yl)amino)phenyl)acetyl)-N-(3-chlorophenyl)hydrazine-1-carboxamide* (**9b**)
White solid, yield: 86.2%; ^{1}H NMR (300 MHz, DMSO-*d*$_6$) δ 9.89 (s, 1H), 8.99 – 8.66 (m, 2H), 8.21 (s, 1H), 7.70 – 7.55 (m, 3H), 7.43 – 7.20 (m, 5H), 7.19 – 7.06 (m, 4H), 7.04 – 6.70 (m, 2H), 4.43 (s, 2H), 3.40(s, 2H), 3.09 (s, 2H), 1.74 – 1.35 (m, 6H), 1.25 – 1.05 (m, 3H), 0.97 – 0.66 (m, 2H). ^{13}C NMR (75 MHz, DMSO-*d*$_6$) δ 170.91, 166.29, 164.56, 163.07, 159.86, 155.63, 141.71, 139.68, 137.40, 133.52, 130.72, 129.31, 128.61, 121.94, 120.07, 119.69, 118.23, 117.26, 115.40, 115.12, 56.50, 46.97, 46.64, 43.15, 37.94, 31.08, 26.66, 25.93, 19.02. MS (ESI) *m/z*: 631.9[M+H]$^+$, $C_{32}H_{33}ClFN_9O_2$. Purity: 95.9% (LC-MS).

12. *2-(2-(4-((4-((cyclohexylmethyl)amino)-6-((4-fluorobenzyl)amino)-1,3,5-triazin-2-yl)amino)phenyl)acetyl)-N-(4-chlorophenyl)hydrazine-1-carboxamide* (**9c**)
White solid, yield: 80.4%; ^{1}H NMR (300 MHz, DMSO-*d*$_6$) δ 9.88 (s, 1H), 8.96 – 8.67 (m, 2H), 8.14 (s, 1H), 7.81 – 7.55 (m, 2H), 7.48 (d, *J* = 8.6 Hz, 3H), 7.32 (d, *J* = 10.2 Hz, 4H), 7.12 (s, 4H), 6.85 (t, *J* = 33.5 Hz, 1H), 4.44 (s, 2H), 3.40 (s, 2H), 3.17 – 2.95 (s, 2H), 1.79 – 1.40 (m, 6H), 1.23 – 1.06 (m, 3H), 0.97 – 0.67 (m, 2H). ^{13}C NMR (75 MHz, DMSO-*d*$_6$) δ 170.89, 166.30, 164.55, 163.07, 162.97, 155.68, 139.76, 139.13, 137.41, 129.30, 129.09, 128.94, 128.63, 125.99, 125.87, 120.40, 119.65, 115.40, 115.11, 56.50, 46.93, 46.66, 43.32, 42.87, 37.96, 31.08, 26.65, 25.93, 19.02. MS (ESI) *m/z*: 631.9[M+H]$^+$, $C_{32}H_{33}ClFN_9O_2$. Purity: 97.3% (LC-MS).

13. *2-(2-(4-((4-((cyclohexylmethyl)amino)-6-((4-fluorobenzyl)amino)-1,3,5-triazin-2-yl)amino)phenyl)acetyl)-N-(2-fluorophenyl)hydrazine-1-carboxamide* (**9d**)
White solid, yield: 76.3%; ^{1}H NMR (300 MHz, DMSO-*d*$_6$) δ 9.99 (s, 1H), 8.98 – 8.67 (m, 1H), 8.57 (s, 1H), 8.39 (s, 1H), 8.00 (t, *J* = 7.5 Hz, 1H), 7.81 – 7.54 (m, 2H), 7.49 – 7.27 (m, 3H), 7.25 – 7.08 (m, 6H), 7.03 – 6.94 (m, 1H), 6.79 (d, *J* = 32.7 Hz, 1H), 4.44 (s, 2H), 3.40 (s, 2H), 3.18 – 2.97 (m, 2H), 1.84 – 1.39 (m, 6H), 1.29 – 0.99 (m, 3H), 0.98 – 0.65 (m, 2H). ^{13}C NMR (75 MHz, DMSO-*d*$_6$) δ 170.72, 166.43, 166.30, 166.17, 164.56, 163.07, 159.86, 155.37, 154.32, 154.28, 151.11, 139.66, 137.58, 137.37, 129.41, 129.24, 128.59, 127.81, 127.67, 124.92, 124.88, 123.32, 123.22, 121.51, 120.09, 119.99, 119.82, 119.69, 115.62, 115.38, 115.12, 46.97, 46.64, 43.23, 43.03, 37.96, 31.07, 26.65, 25.92. MS (ESI) *m/z*: 615.9[M+H]$^+$, $C_{32}H_{35}F_2N_9O_2$. Purity: 95.4% (LC-MS).

14. *2-(2-(4-((4-((cyclohexylmethyl)amino)-6-((4-fluorobenzyl)amino)-1,3,5-triazin-2-yl)amino)phenyl)acetyl)-N-(3-fluorophenyl)hydrazine-1-carboxamide* (**9e**)
White solid, yield: 70.4%; ^{1}H NMR (300 MHz, DMSO-*d*$_6$) δ 9.90 (s, 1H), 9.13 – 8.68 (m, 2H), 8.21 (s, 1H), 7.81 – 7.55 (m, 2H), 7.53 – 7.06 (m, 10H), 7.04 – 6.69 (m, 2H), 4.44 (s, 2H), 3.48

–3.41 (m, 2H), 3.17 – 2.97 (m, 2H), 1.83 –1.42 (m, 6H), 1.26 –1.01 (m, 3H), 0.98 – 0.67 (m, 2H). ^{13}C NMR (75 MHz, DMSO-*d$_6$*) δ 170.86, 166.11, 164.37, 163.06, 161.19, 155.61, 152.81, 142.15, 142.00, 139.55, 137.28, 130.70, 130.56, 129.31, 128.71, 120.28, 119.70, 115.41, 115.13, 114.49, 108.71, 108.42, 105.58, 105.23, 46.95, 46.59, 43.18, 37.94, 31.07, 26.64, 25.92. MS (ESI) *m/z*: 616.0[M+H]$^+$, $C_{32}H_{35}F_2N_9O_2$. Purity: 98.0% (LC-MS).

15. *2-(2-(4-((4-((cyclohexylmethyl)amino)-6-((4-fluorobenzyl)amino)-1,3,5-triazin-2-yl)amino)phenyl)acetyl)-N-(4-fluorophenyl)hydrazine-1-carboxamide* (**9f**)
    White solid, yield: 78.3%; ^{1}H NMR (300 MHz, DMSO-*d$_6$*) δ 9.87 (s, 1H), 8.96 – 8.65 (m, 2H), 8.08 (s, 1H), 7.78 – 7.55 (m, 2H), 7.44 (dd, *J* = 8.8, 4.9 Hz, 2H), 7.34 (t, *J* = 4.9 Hz, 2H), 7.25 – 7.01 (m, 6H), 6.85 (t, *J* = 31.3 Hz, 1H), 4.44 (s, 2H), 3.48 – 3.40 (m, 2H), 3.17 – 2.96 (m, 2H), 1.81 – 1.45 (m, 6H), 1.28 – 1.05 (m, 3H), 0.99 – 0.66 (m, 2H). ^{13}C NMR (75 MHz, DMSO-*d$_6$*) δ 170.89, 166.25, 166.12, 164.53, 163.07, 159.86, 159.39, 156.24, 155.89, 139.63, 137.36, 136.44, 129.40, 129.30, 128.68, 120.68, 120.58, 119.94, 119.68, 115.72, 115.42, 115.13, 46.90, 46.67, 43.20, 37.93, 31.07, 26.64, 25.92. MS (ESI) *m/z*: 615.9[M+H]$^+$, $C_{32}H_{35}F_2N_9O_2$. Purity: 96.5% (LC-MS).
16. *2-(2-(4-((4-((cyclohexylmethyl)amino)-6-((4-fluorobenzyl)amino)-1,3,5-triazin-2-yl)amino)phenyl)acetyl)-N-(o-tolyl)hydrazine-1-carboxamide* (**9g**)
    White solid, yield: 74.5%; ^{1}H NMR (300 MHz, DMSO-*d$_6$*) δ 9.94 (s, 1H), 8.96 – 8.65 (m, 1H), 8.35 (s, 1H), 7.91 (s, 1H), 7.78 – 7.54 (m, 3H), 7.34 (s, 3H), 7.25 – 7.02 (m, 6H), 7.02 – 6.69 (m, 2H), 4.44 (s, 2H), 3.51 – 3.41 (m, 2H), 3.17 – 2.93 (m, 2H), 2.16 (s, 3H), 1.81 – 1.41 (m, 6H), 1.21 – 1.05 (m, 3H), 0.91 – 0.70 (m, 2H). ^{13}C NMR (75 MHz, DMSO-*d$_6$*) δ 170.71, 166.29, 164.55, 159.93, 155.95, 139.68, 137.62, 137.38, 130.62, 129.41, 129.27, 128.98, 128.68, 126.55, 123.59, 122.30, 120.06, 119.67, 115.41, 115.12, 56.51, 46.93, 46.65, 43.19, 37.92, 31.07, 26.65, 25.93, 19.02, 18.15. MS (ESI) *m/z*: 612.0[M+H]$^+$, $C_{33}H_{38}FN_9O_2$. Purity: 96.3% (LC-MS).
17. *2-(2-(4-((4-((cyclohexylmethyl)amino)-6-((4-fluorobenzyl)amino)-1,3,5-triazin-2-yl)amino)phenyl)acetyl)-N-(m-tolyl)hydrazine-1-carboxamide* (**9h**)
    White solid, yield: 72.6%; ^{1}H NMR (300 MHz, DMSO-*d$_6$*) δ 10.12 (s, 1H), 9.22 – 8.85 (m, 1H), 8.85 (s, 1H), 8.28 (s, 1H), 8.04 – 7.80 (m, 2H), 7.71 – 7.45 (m, 5H), 7.41 – 7.31 (m, 5H), 7.28 – 6.96 (m, 2H), 4.69 (s, 2H), 3.73 – 3.65 (m, 2H), 3.41 – 3.23 (m, 2H), 2.76 (s, 3H), 2.01 – 1.62 (m, 6H), 1.51 – 1.29 (m, 3H), 1.24 – 0.92 (m, 2H). ^{13}C NMR (75 MHz, DMSO-*d$_6$*) δ 170.85, 166.35, 166.13, 164.54, 155.74, 139.97, 139.66, 138.26, 137.35, 129.41, 129.29, 128.96, 128.69, 123.06, 119.68, 119.34, 116.02, 115.41, 115.12, 101.03, 46.74, 46.63, 43.36, 37.97, 31.07, 26.66, 25.93, 21.64. MS (ESI) *m/z*: 612.0[M+H]$^+$, $C_{33}H_{38}FN_9O_2$. Purity: 97.2% (LC-MS).
18. *2-(2-(4-((4-((cyclohexylmethyl)amino)-6-((4-fluorobenzyl)amino)-1,3,5-triazin-2-yl)amino)phenyl)acetyl)-N-(p-tolyl)hydrazine-1-carboxamide* (**9i**)
    White solid, yield: 78.6%; ^{1}H NMR (300 MHz, DMSO-*d$_6$*) δ 9.86 (s, 1H), 9.03 – 8.67 (m, 1H), 8.58 (s, 1H), 8.00 (s, 1H), 7.78 – 7.51 (m, 2H), 7.43 – 7.00 (m, 11H), 6.85 (t, *J* = 27.3 Hz, 1H), 4.44 (s, 2H), 3.48 – 3.40 (m, 2H), 3.19 – 2.95 (m, 2H), 2.22 (s, 3H), 1.82 – 1.38 (m, 6H), 1.27 – 1.02 (m, 3H), 0.98 – 0.66 (m, 2H). ^{13}C NMR (75 MHz, DMSO-*d$_6$*) δ 170.85, 166.40, 166.29, 166.17, 164.56, 163.07, 159.87, 155.82, 139.65, 137.50, 131.12, 129.50, 129.29, 128.70, 120.02, 119.68, 118.94, 115.40, 115.12, 46.89, 46.67, 43.20, 42.91, 37.95, 31.07, 26.65, 25.93, 20.78. MS (ESI) *m/z*: 612.0[M+H]$^+$, $C_{33}H_{38}FN_9O_2$. Purity: 97.1% (LC-MS).
19. *2-(2-(4-((4-((cyclohexylmethyl)amino)-6-((4-fluorobenzyl)amino)-1,3,5-triazin-2-yl)amino)phenyl)acetyl)-N-(4-(trifluoromethyl)phenyl)hydrazine-1-carboxamide* (**9j**)

White solid, yield: 79.8%; ^{1}H NMR (300 MHz, DMSO-d_6) δ 9.93 (s, 1H), 9.16 (s, 1H), 8.97 – 8.66 (m, 1H), 8.28 (s, 1H), 7.78 – 7.53 (m, 6H), 7.42 – 7.06 (m, 7H), 6.85 (t, J = 30.7 Hz, 1H), 4.40 (s, 2H), 3.55 – 3.41 (m, 2H), 3.19 – 2.95 (m, 2H), 1.78 – 1.41 (m, 6H), 1.27 – 1.03 (m, 3H), 0.98 – 0.68 (m, 2H). ^{13}C NMR (75 MHz, DMSO-d_6) δ 170.89, 166.43, 166.30, 166.17, 164.57, 163.06, 159.87, 155.51, 143.91, 139.68, 137.35, 129.41, 129.30, 128.60, 126.81, 126.37, 123.22, 122.51, 122.09, 120.05, 119.69, 118.51, 115.40, 115.11, 46.94, 46.62, 43.24, 42.98, 37.96, 31.07, 26.64, 25.93. MS (ESI) *m/z*: 665.9[M+H]$^+$, $C_{33}H_{35}F_4N_9O_2$. Purity: 97.2% (LC-MS).

20. *2-(2-(4-((4-((cyclohexylmethyl)amino)-6-((4-fluorobenzyl)amino)-1,3,5-triazin-2-yl)amino)phenyl)acetyl)-N-(4-(trifluoromethoxy)phenyl)hydrazine-1-carboxamide* (**9k**)
White solid, yield: 82.6%; ^{1}H NMR (300 MHz, DMSO-d_6) δ 9.90 (s, 1H), 8.99 – 8.68 (m, 2H), 8.17 (s, 1H), 7.77 – 7.47 (m, 4H), 7.39 – 7.07 (m, 9H), 6.90 (d, J = 32.3 Hz, 1H), 4.44 (s, 2H), 3.48 – 3.41 (m, 2H), 3.15 – 2.97 (m, 2H), 1.74 – 1.36 (m, 6H), 1.25 – 1.03 (m, 3H), 0.97 – 0.67 (m, 2H). ^{13}C NMR (75 MHz, DMSO-d_6) δ 172.35, 166.43, 166.30, 166.18, 164.58, 163.08, 159.88, 139.98, 137.61, 137.33, 129.50, 129.42, 129.31, 127.25, 120.09, 119.97, 119.82, 115.38, 115.10, 52.02, 46.98, 46.66, 43.27, 43.15, 42.98, 38.08, 37.92, 31.07, 26.65, 25.93. MS (ESI) *m/z*: 681.9[M+H]$^+$, $C_{33}H_{35}F_4N_9O_3$. Purity: 97.6% (LC-MS).
21. *2-(2-(4-((4-((cyclohexylmethyl)amino)-6-((4-fluorobenzyl)amino)-1,3,5-triazin-2-yl)amino)phenyl)acetyl)-N-(4-cyanophenyl)hydrazine-1-carboxamide* (**9l**)
White solid, yield: 83.3%; ^{1}H NMR (300 MHz, DMSO-d_6) δ 9.95 (s, 1H), 9.44 (s, 1H), 8.97 – 8.66 (m, 1H), 8.40 (s, 1H), 7.67 (dd, J = 18.0, 8.7 Hz, 6H), 7.47 – 7.07 (m, 7H), 6.85 (t, J = 29.8 Hz, 1H), 4.44 (s, 2H), 3.50 – 3.41 (m, 2H), 3.18 – 2.96 (m, 2H), 1.67 (m, 6H), 1.28 – 0.99 (m, 3H), 0.99 – 0.66 (m, 2H). ^{13}C NMR (75 MHz, DMSO-d_6) δ 170.84, 166.29, 164.54, 163.06, 159.86, 155.31, 144.72, 139.64, 137.34, 133.62, 129.41, 129.30, 128.59, 119.76, 118.62, 115.40, 115.12, 103.73, 99.99, 46.93, 42.94, 37.96, 31.06, 26.65, 25.92. MS (ESI) *m/z*: 665.9[M+H]$^+$, $C_{33}H_{35}FN_{10}O_2$. Purity: 96.5% (LC-MS).
22. *2-(2-(4-((4-((cyclohexylmethyl)amino)-6-((4-fluorobenzyl)amino)-1,3,5-triazin-2-yl)amino)phenyl)acetyl)-N-(4-nitrophenyl)hydrazine-1-carboxamide* (**9m**)
Yellow solid, yield: 75.4%; ^{1}H NMR (300 MHz, DMSO-d_6) δ 9.98 (s, 1H), 9.59 (d, J = 45.7 Hz, 1H), 8.97 – 8.64 (m, 1H), 8.45 (s, 1H), 8.17 (d, J = 9.1 Hz, 2H), 7.81 – 7.55 (m, 4H), 7.45 – 7.08 (m, 7H), 6.84 (t, J = 35.3 Hz, 1H), 4.44 (s, 2H), 3.51 – 3.42 (m, 2H), 3.18 – 2.95 (m, 2H), 1.74 – 1.39 (m, 6H), 1.28 – 1.01 (m, 3H), 0.99 – 0.66 (m, 2H). ^{13}C NMR (75 MHz, DMSO-d_6) δ 170.87, 166.30, 166.16, 164.56, 163.07, 159.86, 155.18, 146.84, 141.57, 139.69, 137.37, 129.41, 129.31, 128.54, 126.84, 125.46, 119.71, 118.15, 115.40, 115.12, 112.84, 46.97, 46.63, 43.27, 37.95, 31.07, 26.65, 25.93. MS (ESI) *m/z*: 642.9[M+H]$^+$, $C_{32}H_{35}FN_{10}O_4$. Purity: 97.3% (LC-MS).
23. *2-(2-(4-((4-((cyclohexylmethyl)amino)-6-((4-fluorobenzyl)amino)-1,3,5-triazin-2-yl)amino)phenyl)acetyl)-N-benzylhydrazine-1-carboxamide* (**9n**)
White solid, yield: 69.5%; ^{1}H NMR (300 MHz, DMSO-d_6) δ 9.74 (s, 1H), 8.98 – 8.65 (m, 1H), 7.89 (s, 1H), 7.82 – 7.53 (m, 2H), 7.47 – 7.21 (m, 8H), 7.15 – 7.03 (m, 4H), 7.01 – 6.71 (m, 2H), 4.44 (s, 2H), 4.24 (d, J = 5.8 Hz, 2H), 3.36 (s, 2H), 3.17 – 2.97 (m, 2H), 1.76 – 1.35 (m, 6H), 1.28 – 1.03 (m, 3H), 0.94 – 0.74 (m, 2H). ^{13}C NMR (75 MHz, DMSO-d_6) δ 170.88, 166.40, 166.28, 166.20, 164.56, 163.06, 159.87, 158.68, 140.96, 139.61, 137.63, 137.35, 129.41, 129.33, 128.76, 128.58, 127.53, 127.39, 126.99, 120.06, 119.95, 119.85, 119.65, 115.40, 115.12, 46.93,

46.63, 43.29, 43.11, 37.94, 31.08, 26.65, 25.93. MS (ESI) *m/z*: 612.0$[M+H]^+$, $C_{33}H_{38}FN_9O_2$. Purity: 97.5% (LC-MS).

24. *2-(2-(4-((4-((cyclohexylmethyl)amino)-6-((4-fluorobenzyl)amino)-1,3,5-triazin-2-yl)amino)phenyl)acetyl)-N-(2-chlorophenyl)hydrazine-1-carbothioamide* (**10a**)
White solid, yield: 78.6%; ^{1}H NMR (300 MHz, DMSO-d_6) δ 10.33 – 9.99 (m, 1H), 9.95 – 9.66 (m, 1H), 9.45 (s, 1H), 8.99 – 8.65 (m, 1H), 7.79 – 7.56 (m, 2H), 7.49 (d, *J* = 7.8 Hz, 2H), 7.38 – 7.02 (m, 9H), 6.84 (t, *J* = 37.2 Hz, 1H), 4.44 (d, *J* = 5.0 Hz, 2H), 3.43 (t, *J* = 37.5 Hz, 2H), 3.16 – 2.95 (m, 2H), 1.79 – 1.29 (m, 6H), 1.26 – 1.01 (m, 3H), 0.98 – 0.67 (m, 2H). ^{13}C NMR (75 MHz, DMSO-d_6) δ 182.18, 170.90, 166.42, 166.35, 166.19, 164.55, 163.04, 159.88, 139.70, 137.33, 131.00, 129.76, 129.56, 129.42, 128.30, 127.53, 119.91, 119.62, 115.41, 115.13, 46.92, 46.64, 43.31, 43.00, 37.94, 31.07, 26.65, 25.93. MS (ESI) *m/z*: 647.9$[M+H]^+$, $C_{32}H_{35}ClFN_9OS$. Purity: 96.4% (LC-MS).

25. *2-(2-(4-((4-((cyclohexylmethyl)amino)-6-((4-fluorobenzyl)amino)-1,3,5-triazin-2-yl)amino)phenyl)acetyl)-N-(3-chlorophenyl)hydrazine-1-carbothioamide* (**10b**)
White solid, yield: 75.4%; ^{1}H NMR (300 MHz, DMSO-d_6) δ 10.14 (s, 1H), 9.92 – 9.54 (m, 2H), 8.99 – 8.63 (m, 1H), 7.65 (s, 3H), 7.47 – 7.03 (m, 10H), 6.84 (t, *J* = 32.1 Hz, 1H), 4.44 (s, 2H), 3.45 (s, 2H), 3.15 – 2.97 (m, 2H), 1.80 – 1.31 (m, 6H), 1.24 – 1.03 (m, 3H), 0.99 – 0.67 (m, 2H). ^{13}C NMR (75 MHz, DMSO-d_6) δ 181.43, 170.69, 166.41, 166.28, 166.16, 164.54, 163.07, 159.86, 141.18, 139.68, 137.44, 132.66, 130.17, 129.53, 129.41, 129.31, 128.35, 125.19, 119.93, 119.65, 115.40, 115.13, 46.94, 46.74, 43.21, 42.93, 37.97, 31.07, 26.66, 25.93. MS (ESI) *m/z*: 647.9$[M+H]^+$, $C_{32}H_{35}ClFN_9OS$. Purity: 95.4% (LC-MS).

26. *2-(2-(4-((4-((cyclohexylmethyl)amino)-6-((4-fluorobenzyl)amino)-1,3,5-triazin-2-yl)amino)phenyl)acetyl)-N-(4-chlorophenyl)hydrazine-1-carbothioamide* (**10c**)
White solid, yield: 70.3%; ^{1}H NMR (300 MHz, DMSO-d_6) δ 10.27 – 9.91 (m, 1H), 9.71 (s, 2H), 8.99 – 8.66 (m, 1H), 7.79 – 7.56 (m, 2H), 7.54 – 7.42 (m, 2H), 7.43 – 7.01 (m, 9H), 6.85 (t, *J* = 34.2 Hz, 1H), 4.44 (d, *J* = 5.6 Hz, 2H), 3.44 (s, 2H), 3.16 – 2.97 (m, 2H), 1.86 – 1.32 (m, 6H), 1.25 – 1.03 (m, 3H), 0.97 – 0.66 (m, 2H). ^{13}C NMR (75 MHz, DMSO-d_6) δ 181.53, 170.71, 166.46, 166.28, 166.11, 164.52, 163.05, 159.84, 139.70, 138.62, 137.40, 129.53, 129.41, 129.33, 128.47, 127.68, 119.97, 119.65, 115.40, 115.12, 46.94, 46.65, 43.31, 43.08, 38.00, 31.07, 26.65, 25.93. MS (ESI) *m/z*: 647.9$[M+H]^+$, $C_{32}H_{35}ClFN_9OS$. Purity: 96.5% (LC-MS).

27. *2-(2-(4-((4-((cyclohexylmethyl)amino)-6-((4-fluorobenzyl)amino)-1,3,5-triazin-2-yl)amino)phenyl)acetyl)-N-(2-fluorophenyl)hydrazine-1-carbothioamide* (**10d**)
White solid, yield: 78.9%; ^{1}H NMR (300 MHz, DMSO-d_6) δ 10.17 (s, 1H), 9.77 (s, 1H), 9.48 (s, 1H), 8.93 – 8.64 (m, 1H), 7.76 – 7.54 (m, 2H), 7.53 – 6.99 (m, 11H), 6.85 (t, *J* = 32.4 Hz, 1H), 4.44 (d, *J* = 4.8 Hz, 2H), 3.42 (t, *J* = 22.8 Hz, 2H), 3.16 – 2.98 (m, 2H), 1.79 – 1.31 (m, 6H), 1.26 – 1.00 (m, 3H), 0.97 – 0.67 (m, 2H). ^{13}C NMR (75 MHz, DMSO-d_6) δ 182.65, 170.67, 166.33, 164.53, 163.06, 159.86, 139.66, 137.38, 129.54, 129.42, 128.37, 127.61, 124.40, 119.85, 119.60, 116.29, 116.02, 115.40, 115.13, 46.92, 46.63, 43.24, 43.01, 37.95, 31.06, 26.64, 25.93. MS (ESI) *m/z*: 631.9$[M+H]^+$, $C_{32}H_{35}F_2N_9OS$. Purity: 97.2% (LC-MS).

28. *2-(2-(4-((4-((cyclohexylmethyl)amino)-6-((4-fluorobenzyl)amino)-1,3,5-triazin-2-yl)amino)phenyl)acetyl)-N-(3-fluorophenyl)hydrazine-1-carbothioamide* (**10e**)
White solid, yield: 72.2%; ^{1}H NMR (300 MHz, DMSO-d_6) δ 10.14 (s, 1H), 9.90 – 9.54 (m, 2H), 8.96 – 8.66 (m, 1H), 7.78 – 7.57 (m, 2H), 7.51 (d, *J* = 9.0 Hz, 1H), 7.45 – 7.06 (m, 9H), 6.98 (t, *J* = 8.5 Hz, 1H), 6.79 (t, *J* = 31.2 Hz, 1H), 4.43 (s, 2H), 3.44 (s, 2H), 3.15 – 2.95 (m, 2H), 1.80

– 1.36 (m, 6H), 1.80 – 1.37 (m, 3H), 0.98 – 0.67 (m, 2H). ^{13}C NMR (75 MHz, DMSO-d_6) δ 181.40, 170.70, 166.30, 164.54, 163.62, 163.07, 160.42, 159.87, 141.51, 141.36, 139.73, 137.39, 130.10, 129.54, 129.42, 128.38, 120.04, 119.65, 115.41, 115.13, 111.89, 46.96, 46.62, 43.21, 42.98, 37.98, 31.08, 26.64, 25.93. MS (ESI) *m/z*: 631.9[M+H]$^+$, $C_{32}H_{35}F_2N_9OS$. Purity: 97.0% (LC-MS).

29. *2-(2-(4-((4-((cyclohexylmethyl)amino)-6-((4-fluorobenzyl)amino)-1,3,5-triazin-2-yl)amino)phenyl)acetyl)-N-(4-fluorophenyl)hydrazine-1-carbothioamide* (**10f**)
White solid, yield: 65.7%; ^{1}H NMR (300 MHz, DMSO-d_6) δ 10.21 (s, 1H), 9.64 (s, 2H), 8.98 – 8.63 (m, 1H), 7.82 – 7.54 (m, 2H), 7.49 – 7.02 (m, 11H), 6.84 (t, *J* = 31.3 Hz, 1H), 4.44 (s, 2H), 3.55 – 3.39 (m, 2H), 3.19 – 2.96 (m, 2H), 1.83 – 1.36 (m, 6H), 1.25 – 0.99 (m, 3H), 0.98 – 0.66 (m, 2H). ^{13}C NMR (75 MHz, DMSO-d_6) δ 181.83, 170.73, 166.42, 166.30, 166.17, 164.55, 163.08, 161.53, 159.87, 158.31, 139.69, 137.39, 135.93, 129.54, 129.42, 129.31, 128.41, 120.01, 119.62, 115.38, 115.08, 46.93, 46.63, 43.24, 42.93, 37.95, 31.07, 26.66, 25.93. MS (ESI) *m/z*: 631.9[M+H]$^+$, $C_{32}H_{35}F_2N_9OS$. Purity: 97.5% (LC-MS).

30. *2-(2-(4-((4-((cyclohexylmethyl)amino)-6-((4-fluorobenzyl)amino)-1,3,5-triazin-2-yl)amino)phenyl)acetyl)-N-(o-tolyl)hydrazine-1-carbothioamide* (**10g**)
White solid, yield: 73.6%; ^{1}H NMR (300 MHz, DMSO-d_6) δ 10.09 (s, 1H), 9.51 (s, 1H), 9.40 (s, 1H), 8.98 – 8.65 (m, 1H), 7.81 – 7.56 (m, 2H), 7.34 (s, 3H), 7.22 – 7.09 (m, 8H), 6.85 (t, *J* = 29.7 Hz, 1H), 4.44 (s, 2H), 3.43 (s, 2H), 3.19 – 2.97 (s, 2H), 2.16 (s, 3H), 1.73 – 1.43 (m, 6H), 1.21 – 1.06 (m, 3H), 0.96 – 0.72 (m, 2H). ^{13}C NMR (75 MHz, DMSO-d_6) δ 182.14, 170.66, 166.41, 166.31, 166.14, 164.55, 163.07, 159.87, 139.67, 138.35, 137.37, 136.14, 135.27, 131.25, 130.54, 129.99, 129.57, 129.42, 129.32, 128.46, 127.71, 127.12, 126.34, 126.29, 119.96, 119.59, 115.40, 115.12, 46.96, 46.64, 43.26, 43.11, 42.95, 39.18, 37.97, 31.08, 26.66, 25.94, 25.60, 18.28, 18.10. MS (ESI) *m/z*: 628.0[M+H]$^+$, $C_{33}H_{38}FN_9OS$. Purity: 97.3% (LC-MS).

31. *2-(2-(4-((4-((cyclohexylmethyl)amino)-6-((4-fluorobenzyl)amino)-1,3,5-triazin-2-yl)amino)phenyl)acetyl)-N-(m-tolyl)hydrazine-1-carbothioamide* (**10h**)
White solid, yield: 68.2%; ^{1}H NMR (300 MHz, DMSO-d_6) δ 10.21 (s, 1H), 9.58 (s, 2H), 8.99 – 8.66 (m, 1H), 7.80 – 7.55 (m, 2H), 7.43 – 7.04 (m, 10H), 7.02 – 6.66 (m, 2H), 4.44 (s, 2H), 3.53 – 3.40 (m, 2H), 3.18 – 2.97 (m, 2H), 2.29 (s, 3H), 1.81 – 1.35 (m, 6H), 1.29 – 1.01 (m, 3H), 0.99 – 0.66 (m, 2H). ^{13}C NMR (75 MHz, DMSO-d_6) δ 181.44, 170.69, 166.27, 164.54, 163.08, 159.87, 139.71, 139.46, 137.83, 137.36, 129.53, 129.42, 129.32, 128.46, 126.11, 119.95, 119.66, 115.40, 115.12, 46.67, 43.26, 37.96, 31.08, 26.66, 25.94, 21.40. MS (ESI) *m/z*: 627.9[M+H]$^+$, $C_{33}H_{38}FN_9OS$. Purity: 98.1% (LC-MS).

32. *2-(2-(4-((4-((cyclohexylmethyl)amino)-6-((4-fluorobenzyl)amino)-1,3,5-triazin-2-yl)amino)phenyl)acetyl)-N-(p-tolyl)hydrazine-1-carbothioamide* (**10i**)
White solid, yield: 66.5%; ^{1}H NMR (300 MHz, DMSO-d_6) δ 10.16 (s, 1H), 9.53 (s, 2H), 8.97 – 8.63 (m, 1H), 7.80 – 7.53 (m, 2H), 7.50 – 7.01 (m, 11H), 6.84 (t, *J* = 35.8 Hz, 1H), 4.44 (d, *J* = 4.7 Hz, 2H), 3.52 – 3.38 (m, 2H), 3.15 – 2.96 (m, 2H), 2.28 (s, 3H), 1.80 – 1.32 (m, 6H), 1.27 – 1.02 (m, 3H), 0.94 – 0.68 (m, 2H). ^{13}C NMR (75 MHz, DMSO-d_6) δ 181.61, 170.63, 166.29, 164.55, 163.07, 159.87, 139.68, 137.34, 137.01, 134.65, 129.53, 129.41, 129.31, 129.04, 128.46, 126.00, 119.90, 119.64, 115.39, 115.12, 46.88, 46.64, 43.24, 43.01, 37.94, 31.06, 26.65, 25.93, 21.01. MS (ESI) *m/z*: 627.9[M+H]$^+$, $C_{33}H_{38}FN_9OS$. Purity: 98.0% (LC-MS).

33. *2-(2-(4-((4-((cyclohexylmethyl)amino)-6-((4-fluorobenzyl)amino)-1,3,5-triazin-2-yl)amino)phenyl)acetyl)-N-(4-(trifluoromethyl)phenyl)hydrazine-1-carbothioamide* (**10j**)

White solid, yield: 67.4%; ^{1}H NMR (300 MHz, DMSO-d_6) δ 10.17 (s, 1H), 9.86 (s, 2H), 8.95 – 8.67 (m, 1H), 7.90 – 7.51 (m, 6H), 7.49 – 7.03 (m, 7H), 6.85 (t, J = 31.3 Hz, 1H), 4.43 (s, 2H), 3.45 (s, 2H), 3.16 – 2.96 (m, 2H), 1.80 – 1.30 (m, 6H), 1.28 – 1.00 (m, 3H), 0.98 – 0.65 (m, 2H). ^{13}C NMR (75 MHz, DMSO-d_6) δ 181.43, 166.24, 164.53, 163.07, 159.80, 143.47, 139.72, 137.34, 129.52, 129.40, 129.31, 128.37, 126.60, 125.97, 125.69, 125.53, 123.00, 120.00, 119.62, 115.40, 115.13, 46.92, 46.66, 43.27, 43.04, 37.95, 31.06, 30.98, 26.64, 25.92. MS (ESI) *m/z*: 681.9[M+H]$^+$, $C_{33}H_{35}F_4N_9OS$. Purity: 97.6% (LC-MS).

34. *2-(2-(4-((4-((cyclohexylmethyl)amino)-6-((4-fluorobenzyl)amino)-1,3,5-triazin-2-yl)amino)phenyl)acetyl)-N-(4-(trifluoromethoxy)phenyl)hydrazine-1-carbothioamide* (**10k**)
    White solid, yield: 74.3%; ^{1}H NMR (300 MHz, DMSO-d_6) δ 10.13 (s, 1H), 9.74 (s, 2H), 8.96 – 8.62 (m, 1H), 7.82 – 7.48 (m, 4H), 7.47 – 7.02 (m, 9H), 6.84 (t, J = 31.7 Hz, 1H), 4.43 (s, 2H), 3.43 (t, J = 14.4 Hz, 2H), 3.17 – 2.95 (m, 2H), 1.80 – 1.32 (m, 6H), 1.28 – 1.01 (m, 3H), 0.99 – 0.66 (m, 2H). ^{13}C NMR (75 MHz, DMSO-d_6) δ 181.58, 166.57, 166.39, 166.16, 164.54, 163.06, 159.86, 139.68, 138.84, 137.45, 129.52, 129.41, 129.31, 128.35, 121.24, 120.00, 119.62, 118.89, 115.40, 115.12, 46.88, 46.64, 43.08, 37.89, 31.07, 26.65, 25.92. MS (ESI) *m/z*: 697.9[M+H]$^+$, $C_{33}H_{35}F_4N_9O_2S$. Purity: 95.4% (LC-MS).
35. *2-(2-(4-((4-((cyclohexylmethyl)amino)-6-((4-fluorobenzyl)amino)-1,3,5-triazin-2-yl)amino)phenyl)acetyl)-N-(4-cyanophenyl)hydrazine-1-carbothioamide* (**10l**)
    White solid, yield: 80.3%; ^{1}H NMR (300 MHz, DMSO-d_6) δ 10.54 – 9.70 (m, 3H), 8.99 – 8.66 (m, 1H), 7.90 – 7.55 (m, 6H), 7.53 – 7.02 (m, 7H), 6.85 (t, J = 30.8 Hz, 1H), 4.44 (s, 2H), 3.46 (s, 2H), 3.18 – 2.96 (m, 2H), 1.77 – 1.33 (m, 6H), 1.27 – 1.00 (m, 3H), 0.99 – 0.66 (m, 2H). ^{13}C NMR (75 MHz, DMSO-d_6) δ 181.13, 166.27, 166.12, 164.51, 163.07, 159.86, 144.21, 139.73, 137.37, 132.82, 129.51, 129.42, 129.31, 128.34, 125.72, 125.28, 120.17, 120.00, 119.66, 119.43, 115.41, 115.12, 46.91, 46.63, 43.27, 42.97, 37.97, 31.07, 26.65, 25.93. MS (ESI) *m/z*: 639.0[M+H]$^+$, $C_{33}H_{35}FN_{10}OS$. Purity: 96.8% (LC-MS).
36. *2-(2-(4-((4-((cyclohexylmethyl)amino)-6-((4-fluorobenzyl)amino)-1,3,5-triazin-2-yl)amino)phenyl)acetyl)-N-(4-nitrophenyl)hydrazine-1-carbothioamide* (**10m**)
    Yellow solid (203 mg), yield: 77.2%; ^{1}H NMR (300 MHz, DMSO-d_6) δ 10.59 – 9.61 (m, 3H), 9.03 – 8.60 (m, 1H), 8.21 (d, J = 8.6 Hz, 2H), 7.90 (d, J = 8.8 Hz, 2H), 7.66 (s, 2H), 7.48 – 7.03 (m, 7H), 6.85 (t, J = 33.4 Hz, 1H), 4.44 (s, 2H), 3.47 (s, 2H), 3.09 (s, 2H), 1.81 – 1.31 (m, 6H), 1.27 – 1.06 (m, 3H), 0.99 – 0.66 (m, 2H). ^{13}C NMR (75 MHz, DMSO-d_6) δ 181.09, 170.78, 166.21, 166.04, 164.49, 163.07, 159.86, 146.25, 143.59, 139.71, 137.33, 129.51, 129.42, 129.32, 128.34, 125.03, 124.70, 124.40, 124.28, 120.03, 119.93, 119.67, 115.41, 115.13, 46.94, 46.65, 43.23, 42.98, 37.94, 31.07, 26.64, 25.93. MS (ESI) *m/z*: 658.9[M+H]$^+$, $C_{32}H_{35}FN_{10}O_3S$. Purity: 97.5% (LC-MS).
37. *2-(2-(4-((4-((cyclohexylmethyl)amino)-6-((4-fluorobenzyl)amino)-1,3,5-triazin-2-yl)amino)phenyl)acetyl)-N-phenylhydrazine-1-carbothioamide* (**10n**)
    White solid, yield: 65.8%; ^{1}H NMR (300 MHz, DMSO-d_6) δ 10.11 (s, 1H), 9.61 (s, 2H), 8.98 – 8.63 (m, 1H), 7.80 – 7.56 (m, 2H), 7.44 (d, J = 7.5 Hz, 2H), 7.33 (t, J = 7.6 Hz, 4H), 7.15 (dd, J = 15.6, 7.2 Hz, 6H), 6.84 (t, J = 34.0 Hz, 1H), 4.44 (s, 2H), 3.52 – 3.40 (m, 2H), 3.16 – 2.95 (m, 2H), 1.74 – 1.41 (m, 6H), 1.23 – 1.02 (m, 3H), 0.96 – 0.73 (m, 2H). ^{13}C NMR (75 MHz, DMSO-d_6) δ 181.52, 170.70, 170.64, 166.44, 166.29, 166.16, 164.55, 163.07, 159.87, 139.70, 139.61, 137.38, 129.53, 129.42, 129.31, 128.57, 128.44, 125.93, 125.58, 125.44, 119.96, 119.63, 115.40, 115.12, 46.65, 43.24, 37.96, 31.08, 26.66, 25.93. MS (ESI) *m/z*: 614.0[M+H]$^+$,

$C_{32}H_{36}FN_9OS$. Purity: 97.0% (LC-MS).

38. *2-(2-(4-((4-((cyclohexylmethyl)amino)-6-((4-fluorobenzyl)amino)-1,3,5-triazin-2-yl)amino)phenyl)acetyl)-N-benzylhydrazine-1-carbothioamide* (**10o**)

    White solid, yield: 60.3%; ^{1}H NMR (300 MHz, DMSO-*d₆*) δ 10.08 (s, 1H), 9.42 (s, 1H), 8.97 – 8.64 (m, 1H), 8.50 (s, 1H), 7.86 – 7.52 (m, 2H), 7.49 – 7.05 (m, 11H), 7.03 – 6.64 (m, 1H), 4.74 (d, *J* = 5.7 Hz, 2H), 4.45 (d, *J* = 5.0 Hz, 2H), 3.41 (s, 2H), 3.17 – 2.98 (m, 2H), 1.87 – 1.29 (m, 6H), 1.25 – 1.05 (m, 3H), 0.99 – 0.66 (m, 2H). ^{13}C NMR (75 MHz, DMSO-d_6) δ 182.69, 170.73, 166.42, 166.29, 166.13, 164.55, 163.08, 159.87, 139.76, 139.68, 137.38, 129.50, 129.31, 128.52, 128.40, 127.48, 127.07, 119.99, 119.86, 119.58, 115.40, 115.13, 47.17, 46.91, 46.62, 43.22, 42.93, 37.97, 31.07, 30.95, 26.66, 25.93. MS (ESI) *m/z*: 627.9[M+H]$^+$, $C_{33}H_{38}FN_9OS$. Purity: 96.9% (LC-MS).

39. *N^2-(cyclohexylmethyl)-N^4-(4-fluorobenzyl)-N^6-(4-((4-chlorophenyl)amino)-1,3,4-oxadiazol-2-yl)methyl)phenyl)-1,3,5-triazine-2,4,6-triamine* (**11c**)

    White solid, yield: 55.2%; ^{1}H NMR (300 MHz, DMSO-d_6) δ 10.55 (s, 1H), 9.06 – 8.72 (m, 1H), 7.81 – 7.62 (m, 2H), 7.55 (d, *J* = 8.9 Hz, 2H), 7.47 – 7.23 (m, 5H), 7.21 – 7.04 (t, *J* = 8.7 Hz, 4H), 6.85 (t, *J* = 34.5 Hz, 1H), 4.43 (s, 2H), 4.08 (s, 2H), 3.16 – 2.95 (m, 2H), 1.72 – 1.45 (m, 6H), 1.19 – 1.00 (m, 3H), 0.97 – 0.73 (m, 2H). ^{13}C NMR (75 MHz, DMSO-d_6) δ 166.39, 166.28, 166.13, 164.55, 164.51, 163.07, 162.77, 160.14, 159.86, 159.73, 140.25, 140.22, 140.20, 138.23, 137.36, 137.31, 129.34, 129.01, 127.57, 125.69, 120.34, 120.11, 118.84, 115.39, 115.11, 100.03, 46.97, 46.63, 43.27, 37.90, 31.05, 30.57, 26.63, 25.91. MS (ESI) *m/z*: 614.0[M+H]$^+$, $C_{32}H_{33}ClFN_9O$. Purity: 96.7% (LC-MS).

40. *N^2-(cyclohexylmethyl)-N^4-(4-fluorobenzyl)-N^6-(4-((5-(p-tolylamino)-1,3,4-oxadiazol-2-yl)methyl)phenyl)-1,3,5-triazine-2,4,6-triamine* (**11i**)

    White solid, yield: 48.6%; ^{1}H NMR (300 MHz, DMSO-d_6) δ 10.23 (s, 1H), 9.03 – 8.71 (m, 1H), 7.83 – 7.61 (m, 2H), 7.49 – 7.05 (m, 11H), 6.86 (t, *J* = 34.7 Hz, 1H), 4.43 (s, 2H), 4.06 (s, 2H), 3.15 – 2.97 (m, 2H), 2.23 (s, 3H), 1.77 – 1.46 (m, 6H), 1.19 – 1.01 (m, 3H), 0.97 – 0.68 (m, 2H). ^{13}C NMR (75 MHz, DMSO-d_6) δ 170.85, 166.40, 166.29, 166.17, 164.56, 163.07, 159.87, 155.82, 139.65, 137.50, 131.12, 129.50, 129.29, 128.70, 120.02, 119.68, 118.94, 115.40, 115.12, 46.89, 46.67, 43.20, 42.91, 37.95, 31.07, 26.65, 25.93, 20.78. MS (ESI) *m/z*: 594.0[M+H]$^+$, $C_{33}H_{36}FN_9O$. Purity: 96.4% (LC-MS).

41. *N^2-(cyclohexylmethyl)-N^4-(4-fluorobenzyl)-N^6-(4-((5-((4-(trifluoromethyl)phenyl)amino)-1,3,4-oxadiazol-2-yl)methyl)phenyl)-1,3,5-triazine-2,4,6-triamine* (**11j**)

    White solid, yield: 47.1%; ^{1}H NMR (300 MHz, DMSO-d_6) δ 10.87 (s, 1H), 9.09 – 8.70 (m, 1H), 7.70 (s, 6H), 7.51 –7.05 (m, 7H), 6.85 (t, *J* = 35.9 Hz, 1H), 4.43 (s, 2H), 4.11 (s, 2H), 3.12 – 2.97 (m, 2H), 1.81 –1.38 (m, 6H), 1.25 –1.08 (m, 3H), 0.96 –0.69 (m, 2H). ^{13}C NMR (75 MHz, DMSO-d_6) δ 166.35, 166.26, 166.18, 164.54, 163.06, 160.08, 159.92, 142.73, 140.27, 137.33, 129.41, 129.31, 129.03, 127.49, 126.87, 126.82, 122.34, 121.91, 120.31, 120.13, 117.21, 115.39, 115.11, 46.92, 46.66, 43.27, 42.91, 37.90, 31.05, 30.57, 26.62, 25.90. MS (ESI) *m/z*: 647.9[M+H]$^+$, $C_{33}H_{33}F_4N_9O$. Purity: 96.2% (LC-MS).

^{1}H NMR, ^{13}C NMR, HPLC purity and ESI-MS of All Compounds

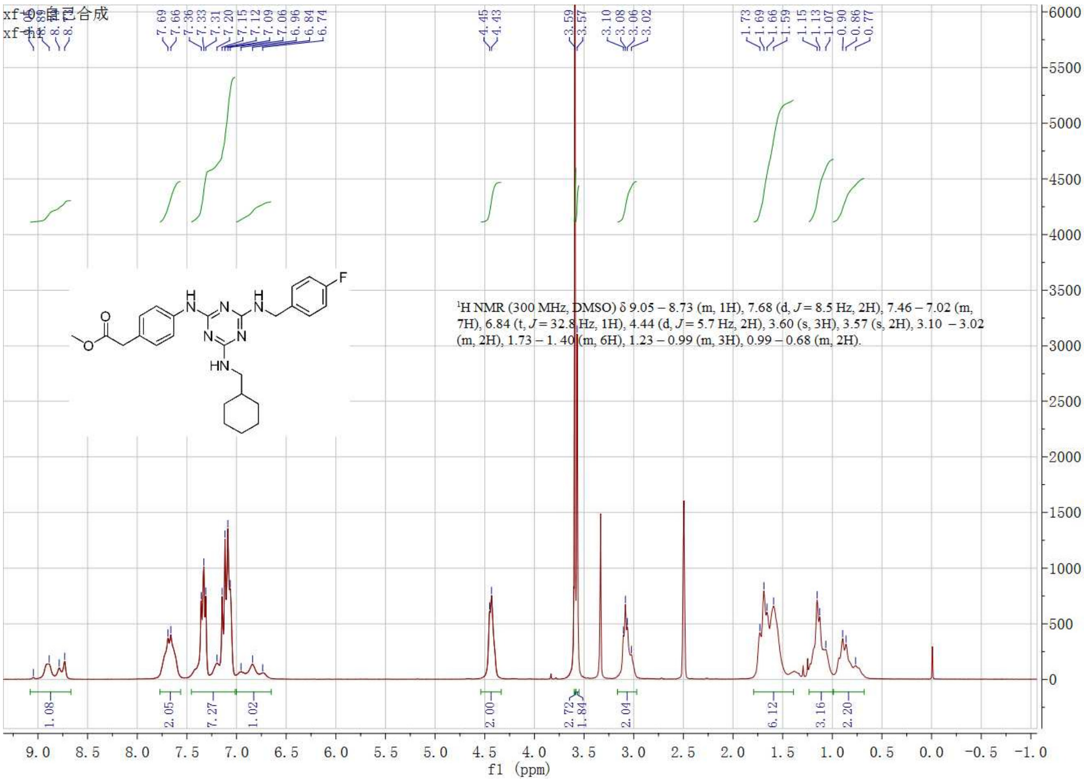

^{1}H-NMR of 4

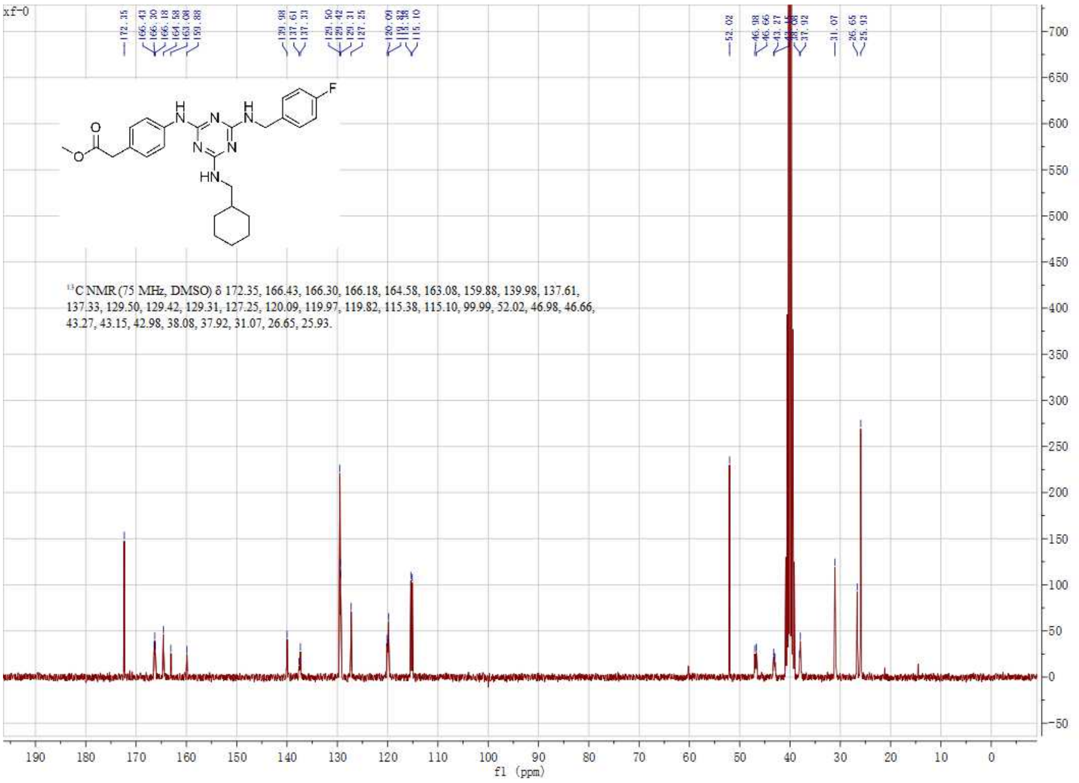

^{13}C-NMR of 4

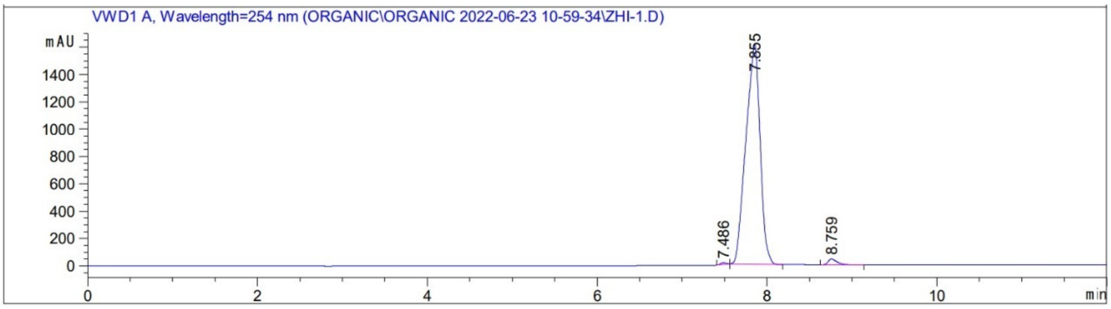

HPLC purity of 4

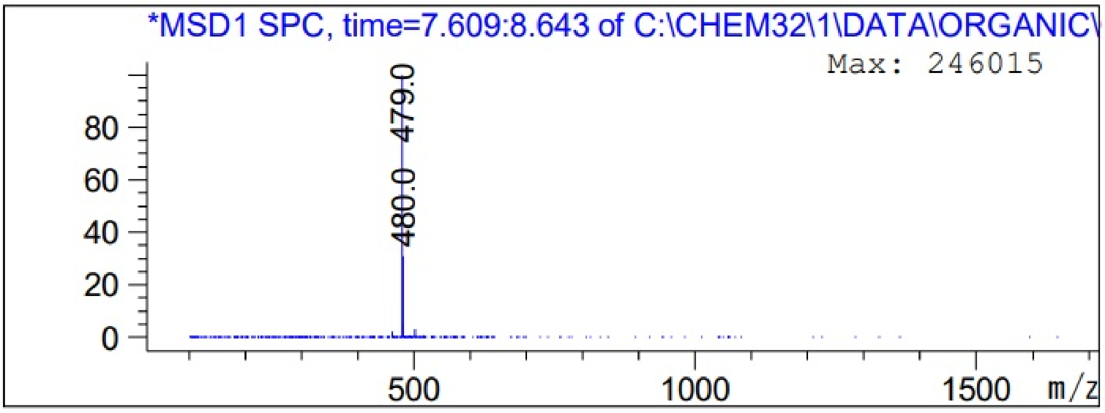

ESI-MS of 4

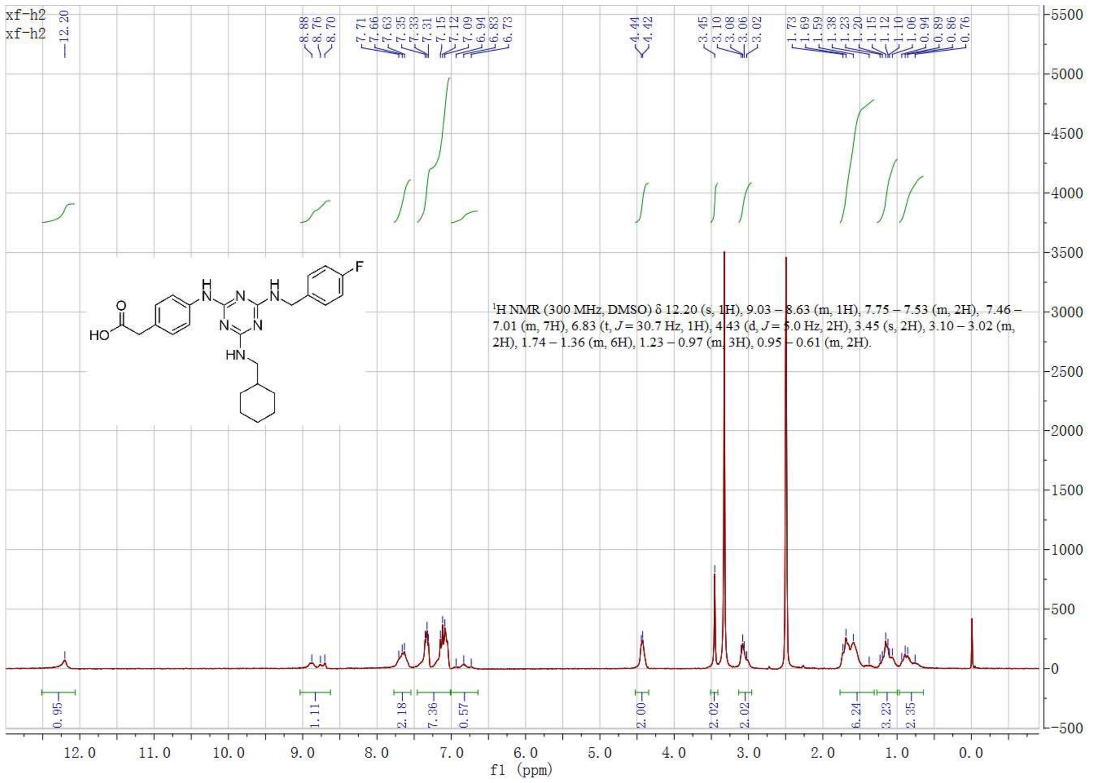

^{1}H-NMR of 5

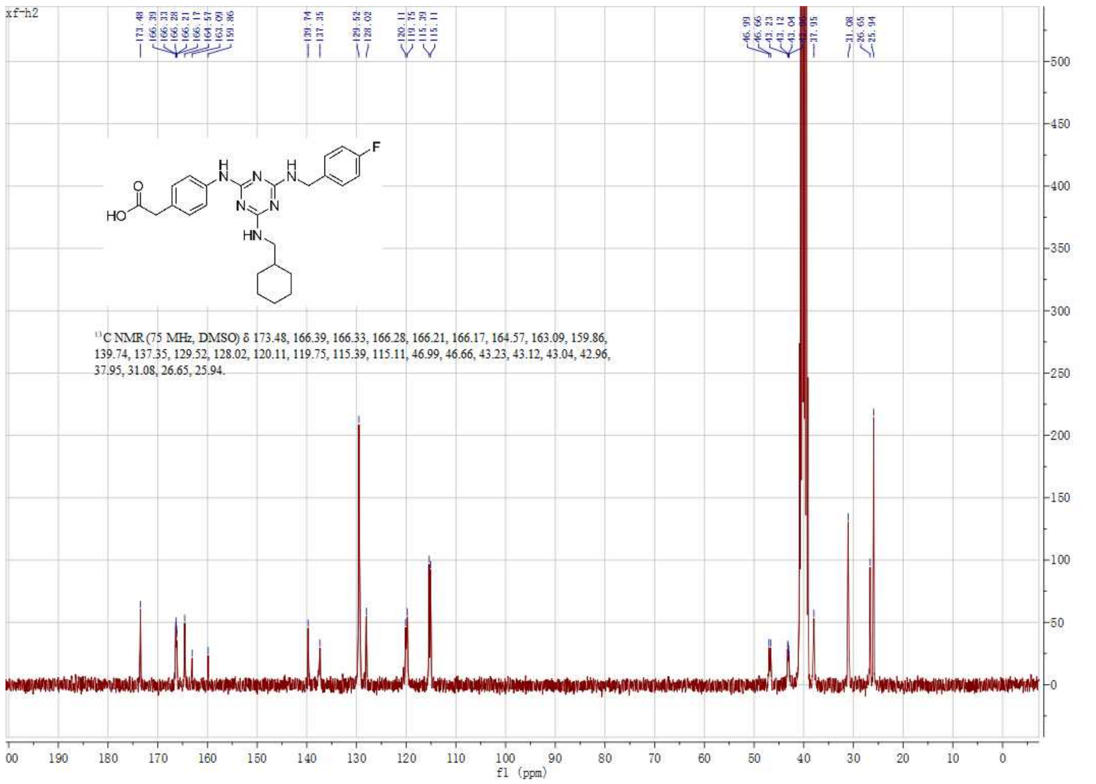

^{13}C-NMR of 5

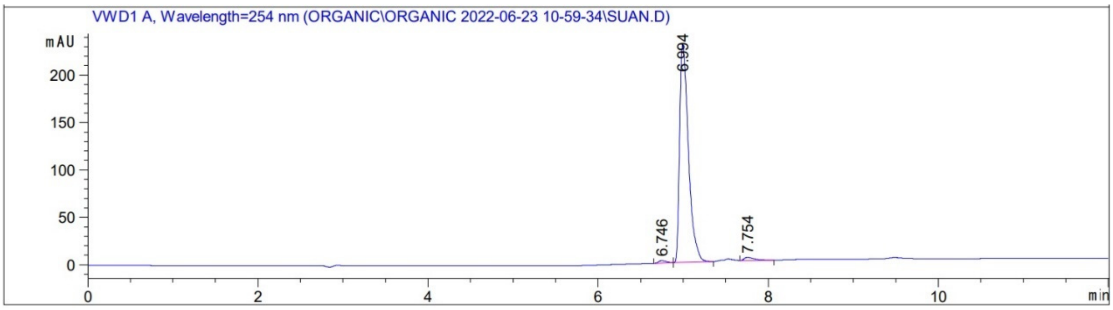

HPLC purity of 5

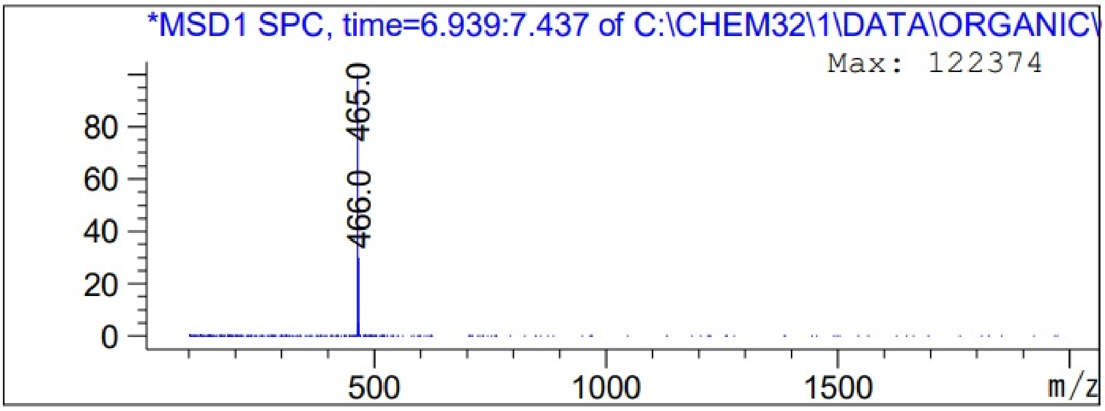

ESI-MS of 5

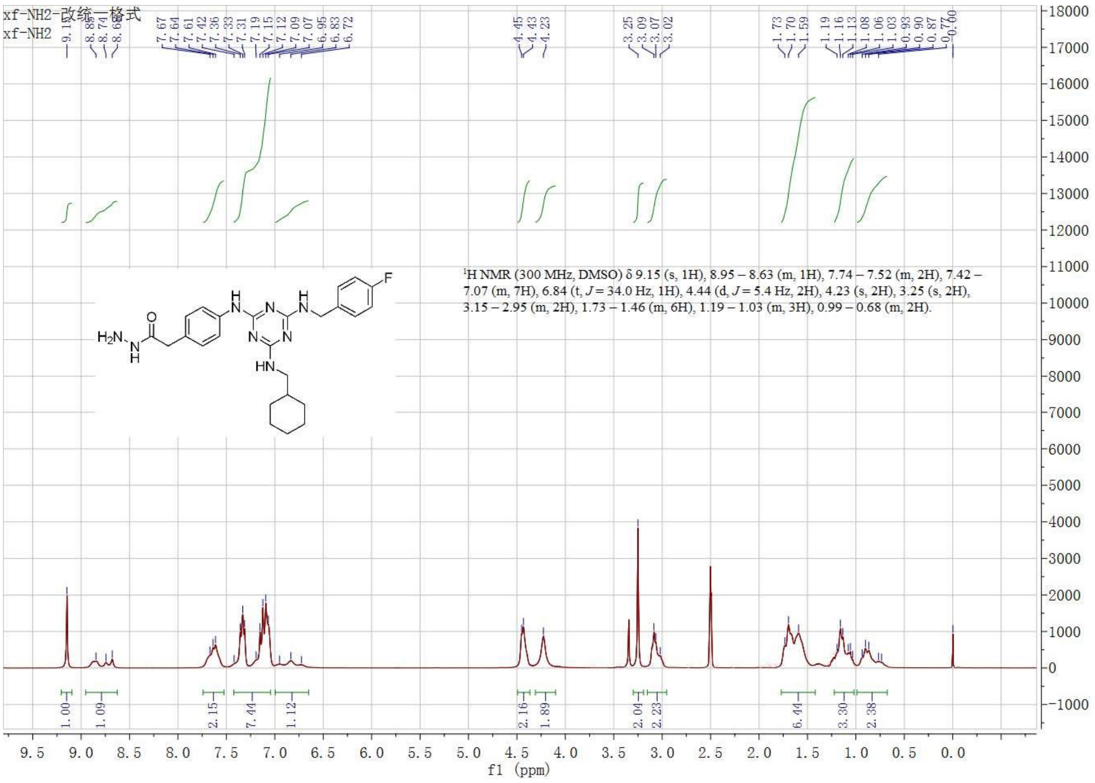

^{1}H-NMR of 6

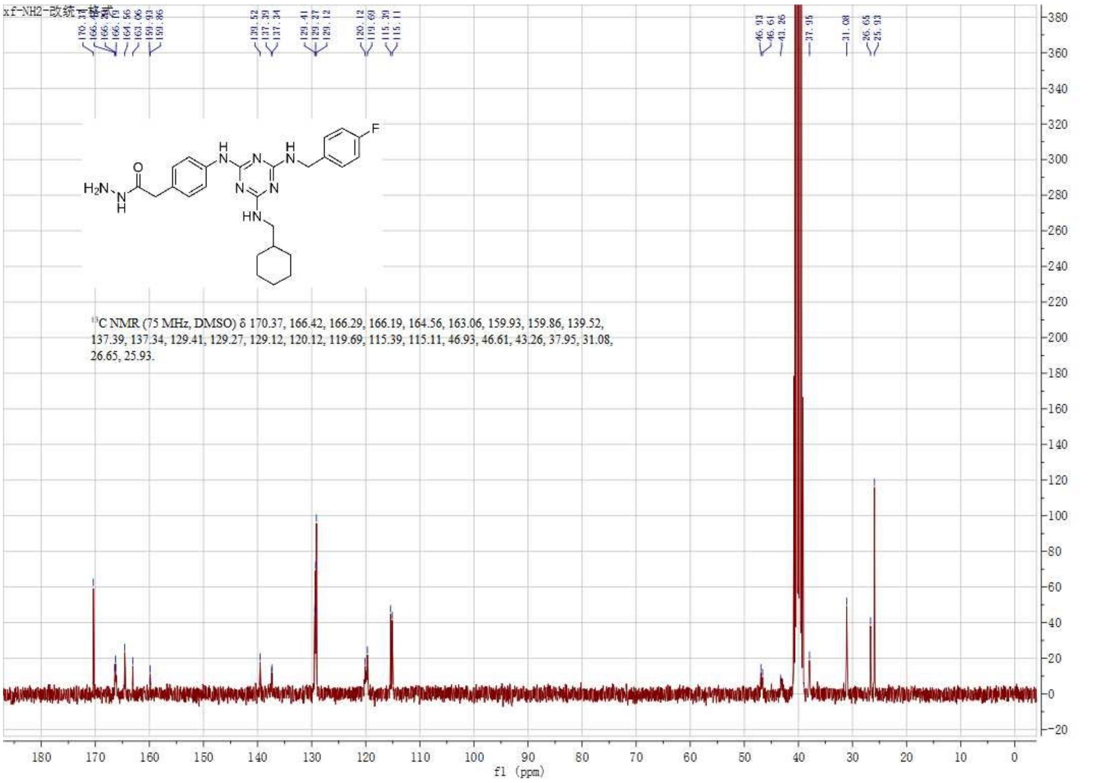

^{13}C-NMR of 6

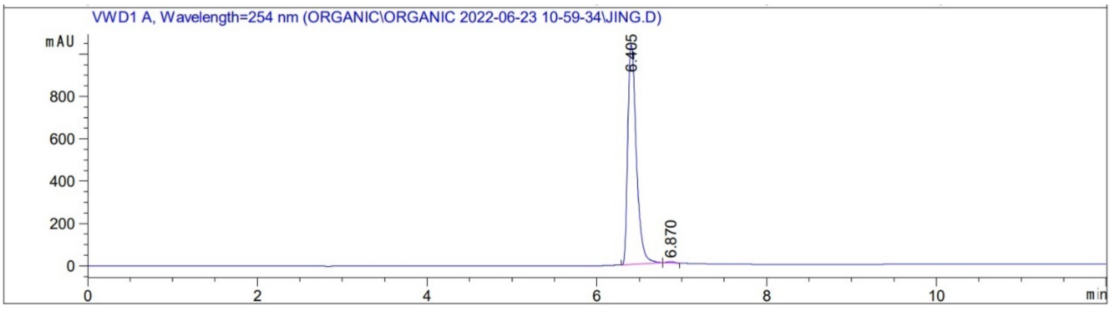

HPLC purity of 6

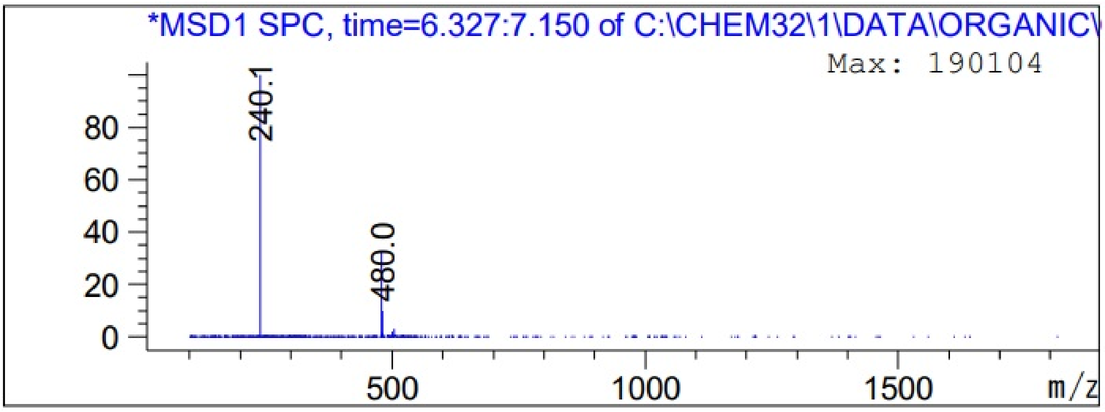

ESI-MS of 6

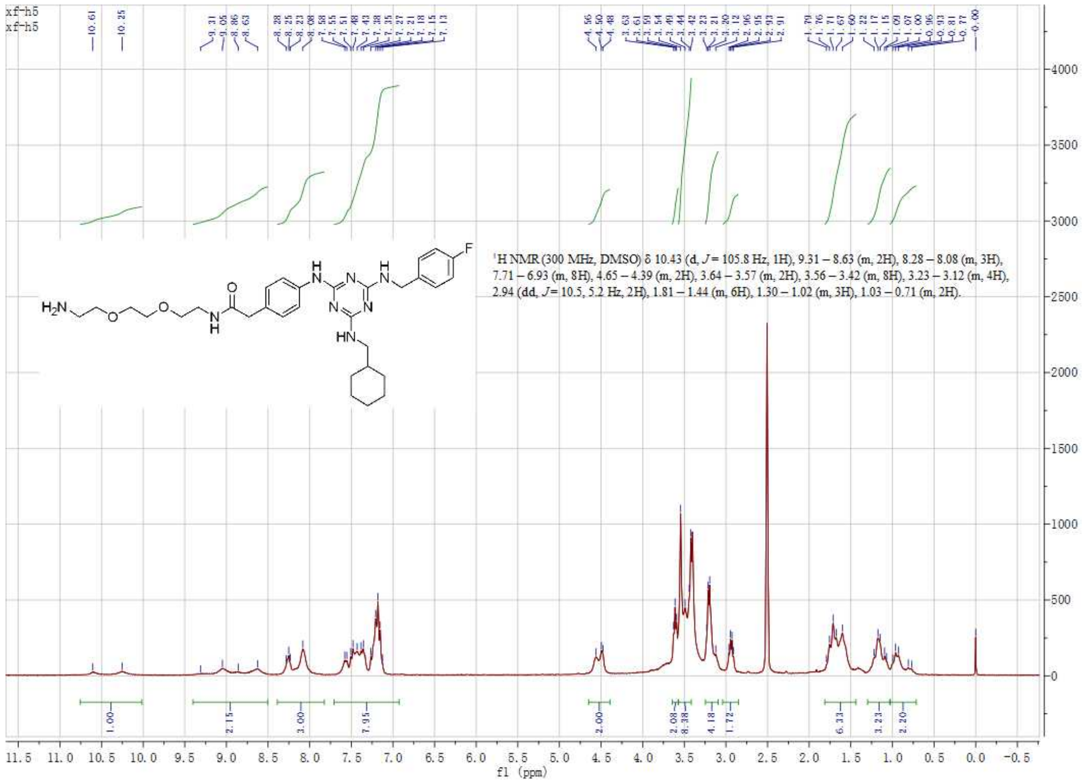

^{1}H-NMR of 8

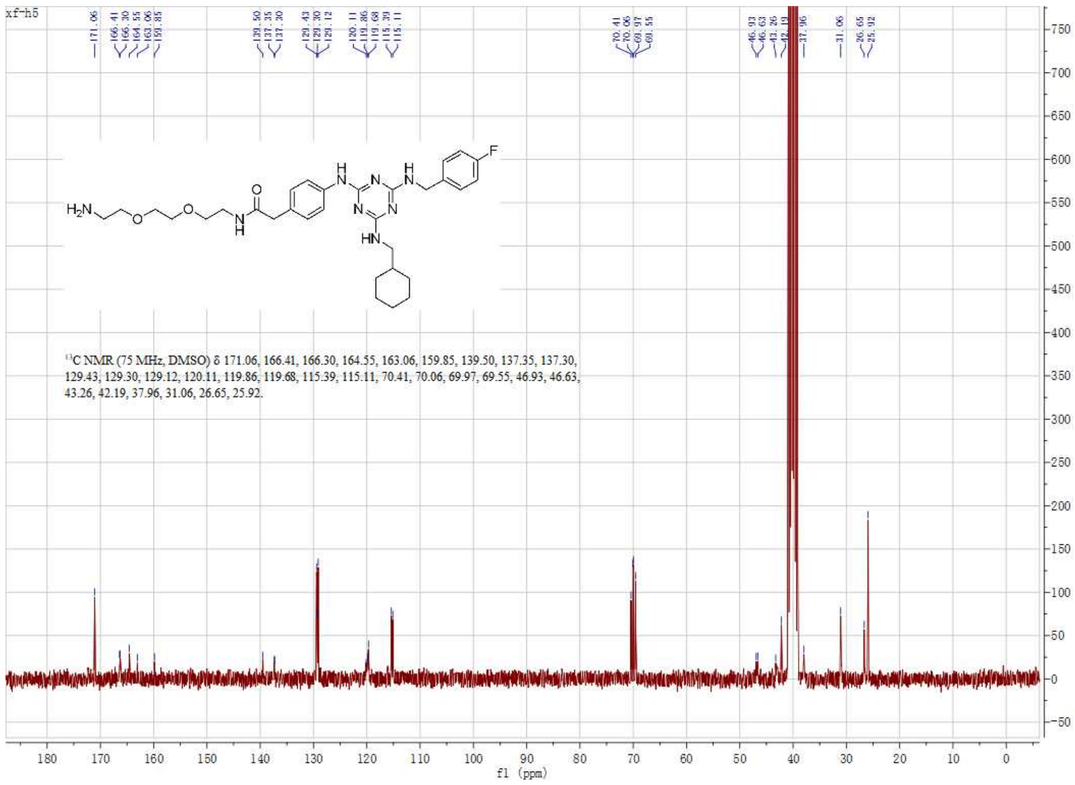

^{13}C-NMR of 8

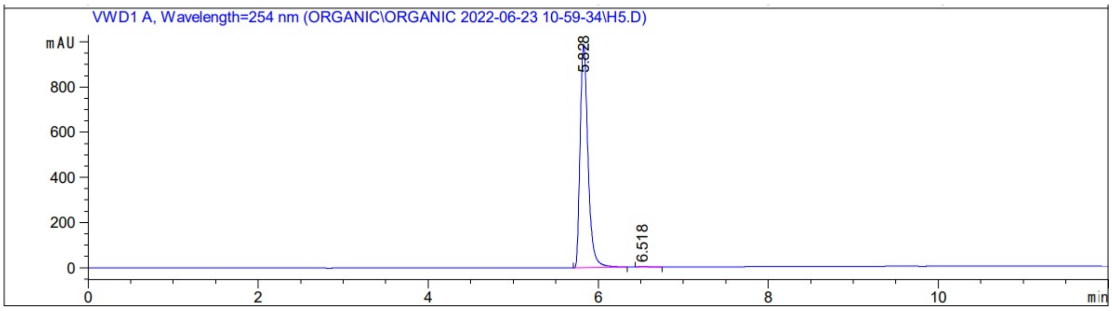

HPLC purity of 8

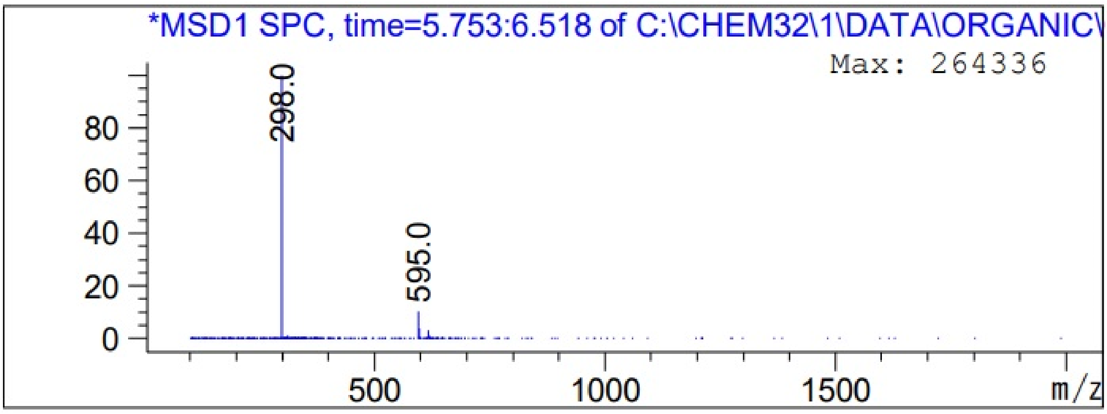

ESI-MS of 8

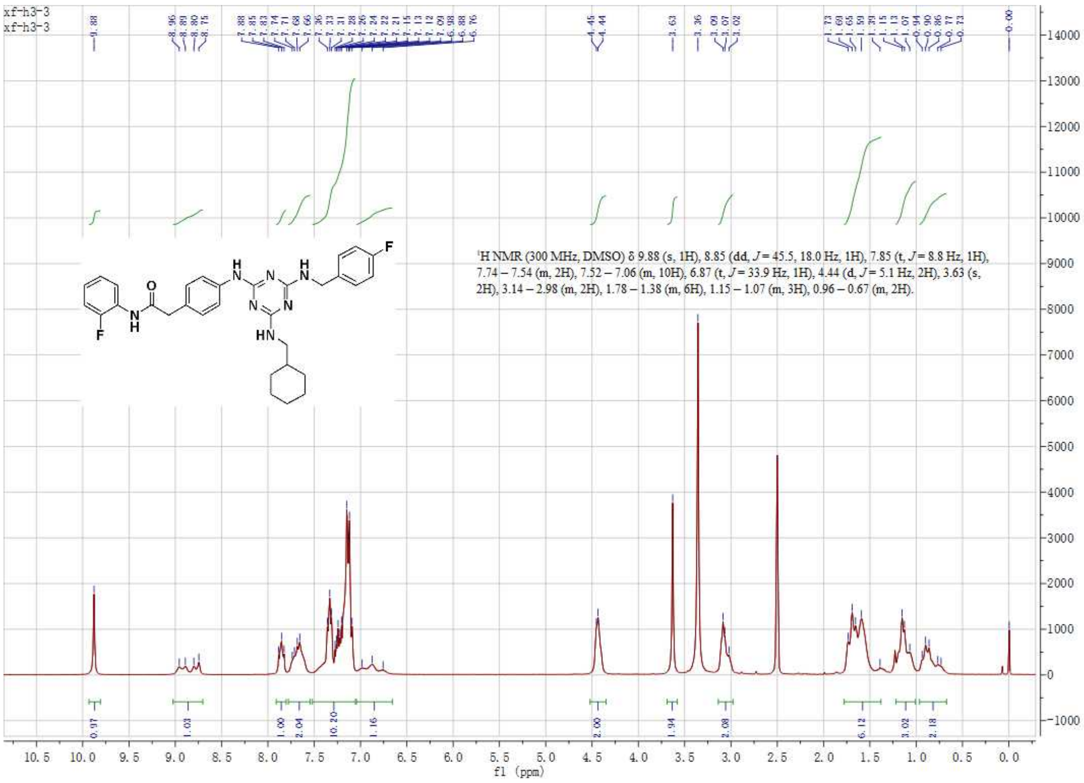

^{1}H-NMR of 8b

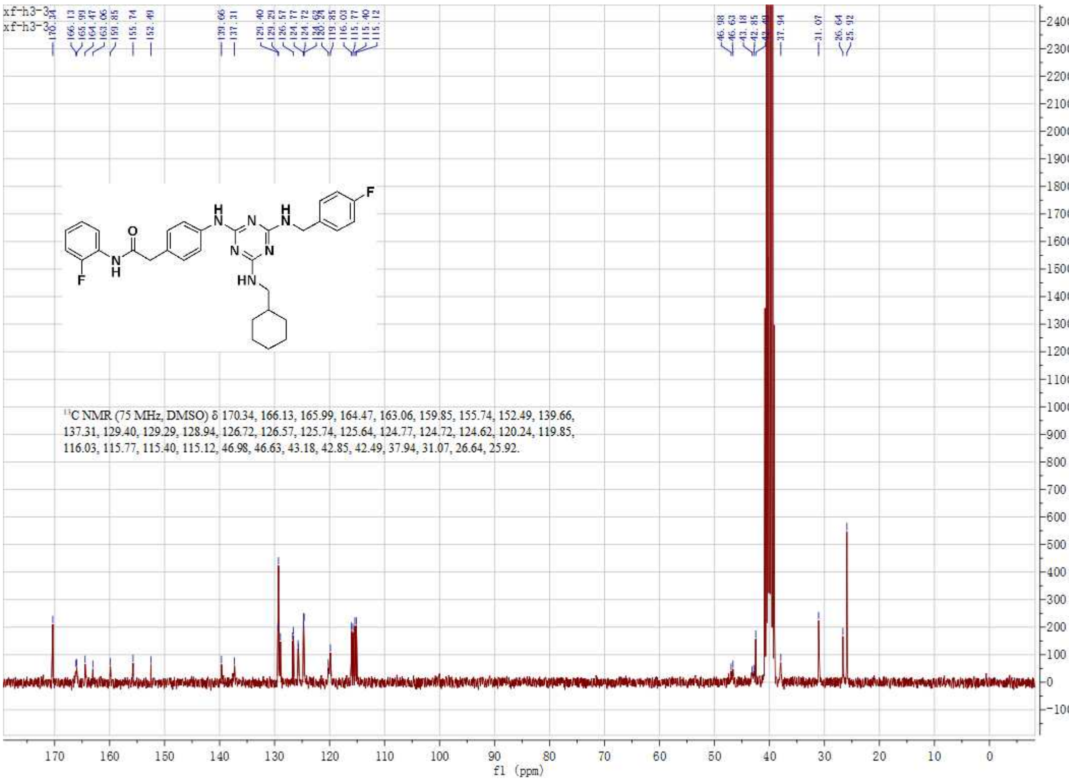

^{13}C-NMR of 8b

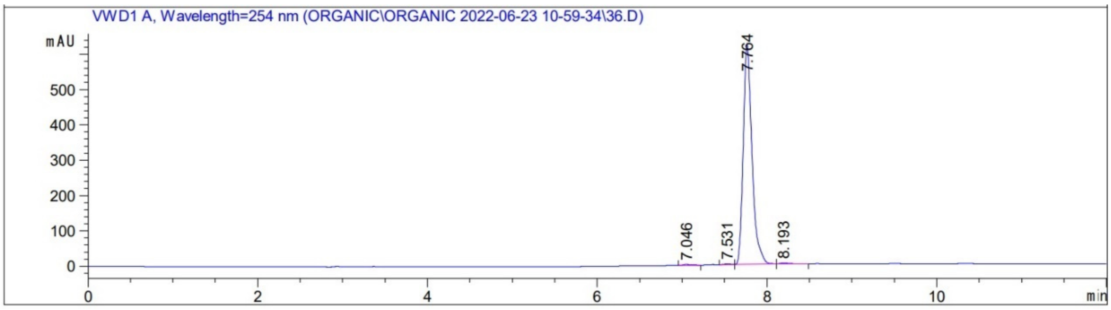

HPLC purity of 8b

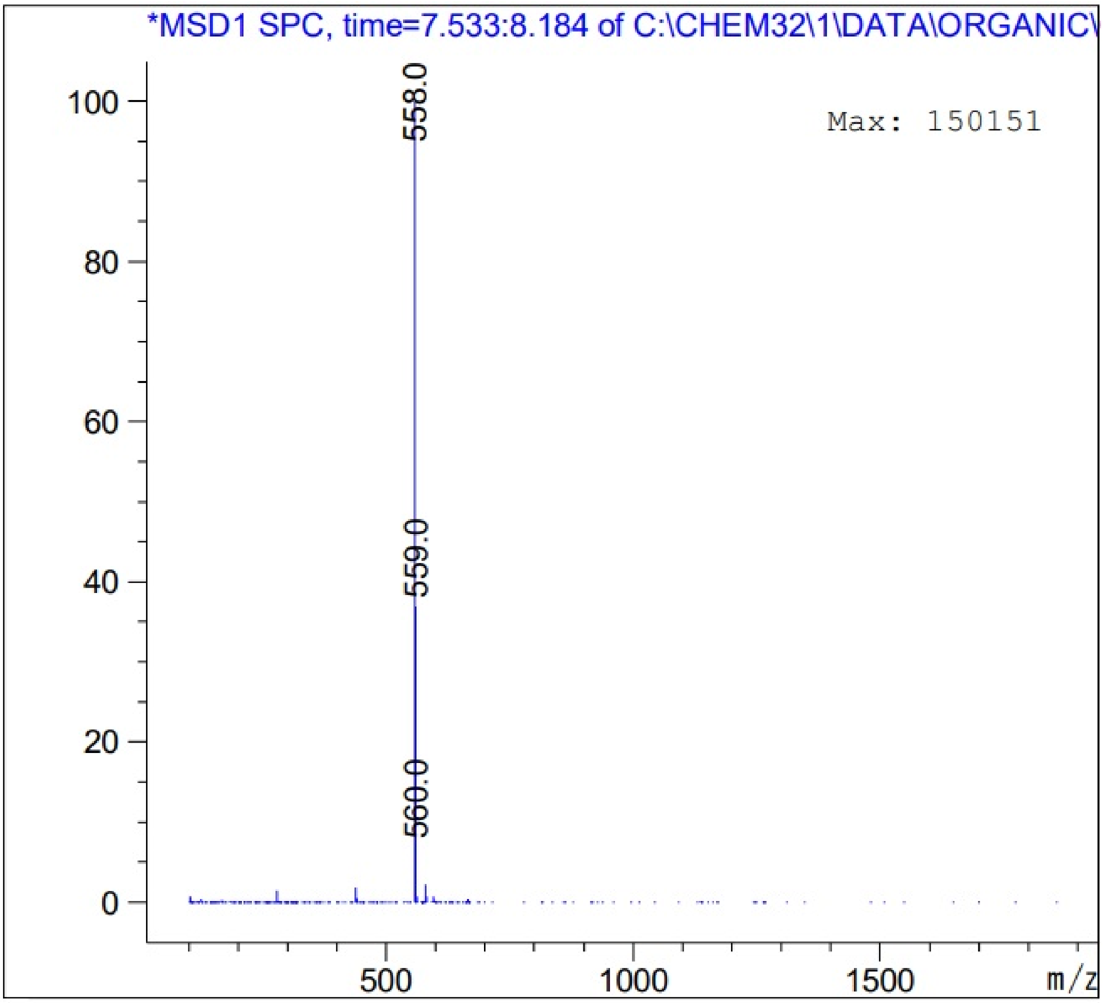

ESI-MS of 8b

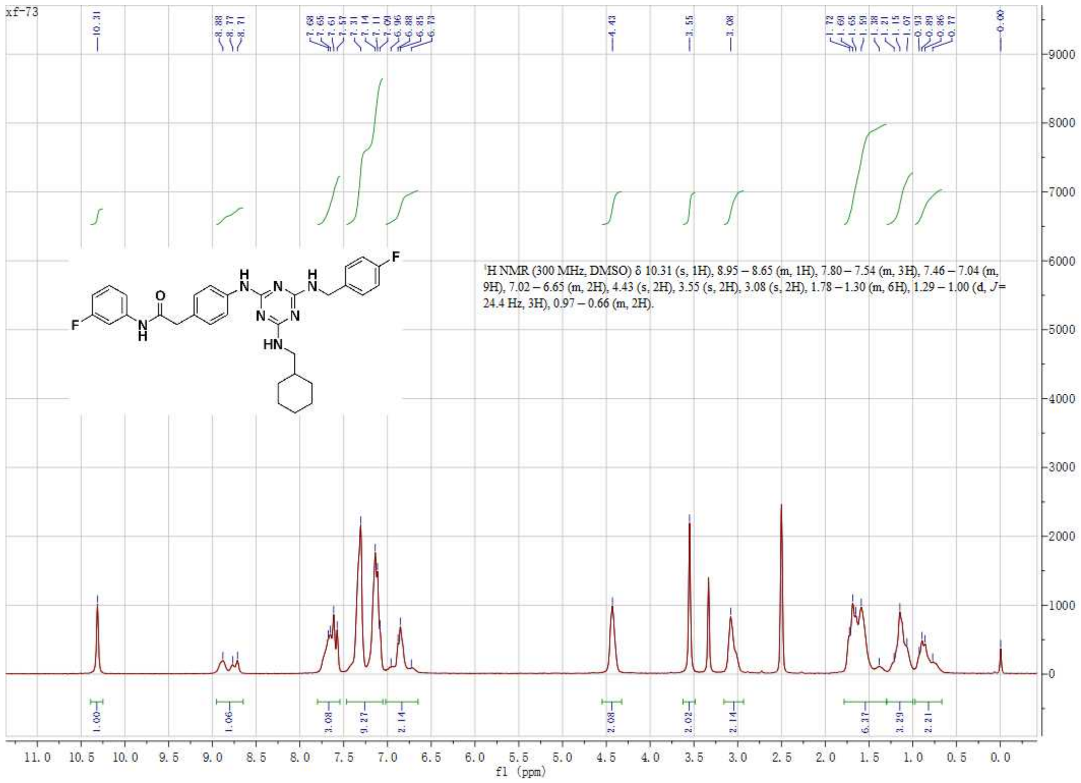

^{1}H-NMR of 8c

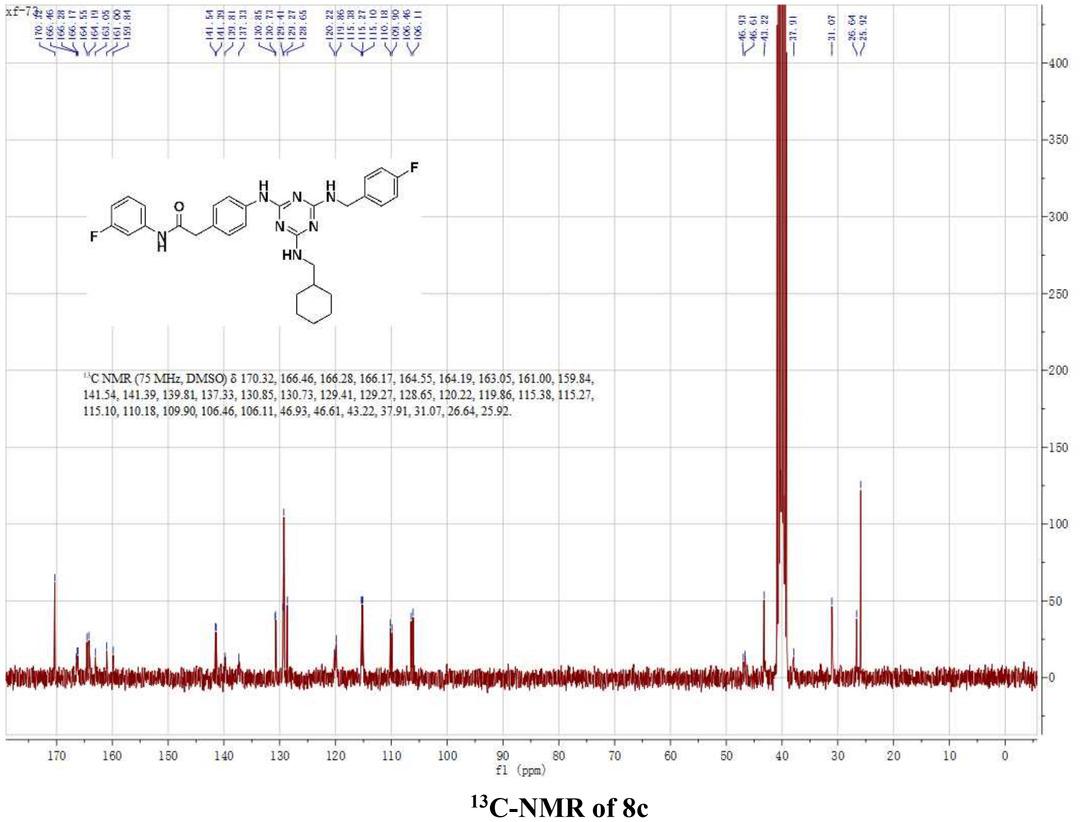

^{13}C-NMR of 8c

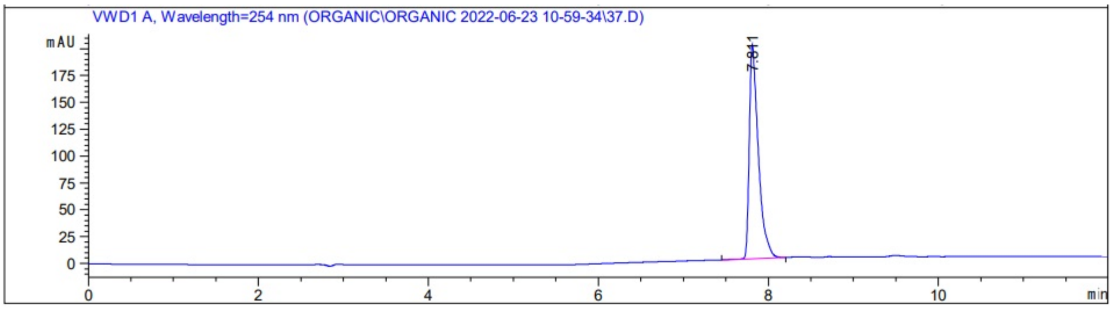

HPLC purity of 8c

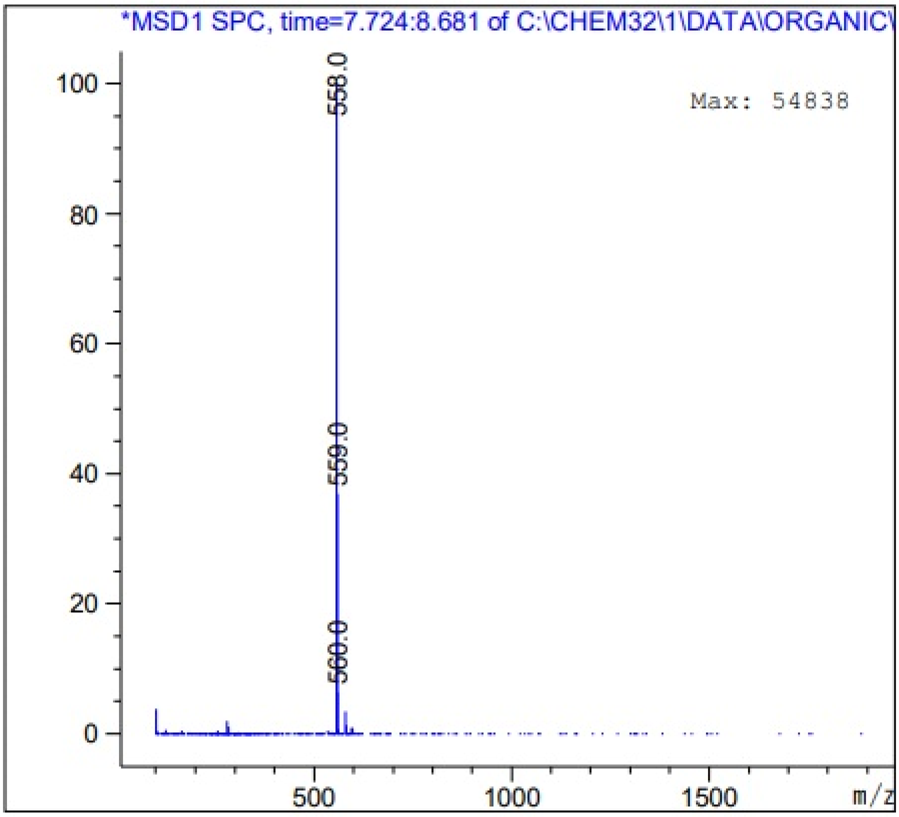

ESI-MS of 8c

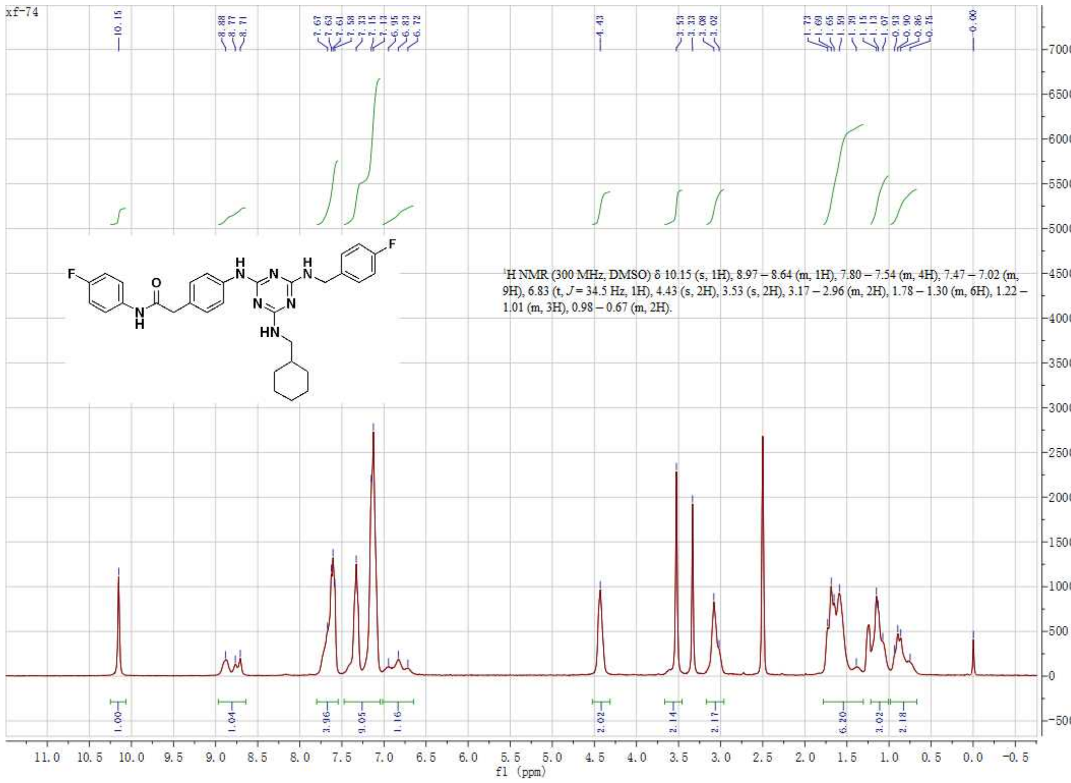

^{1}H-NMR of 8d

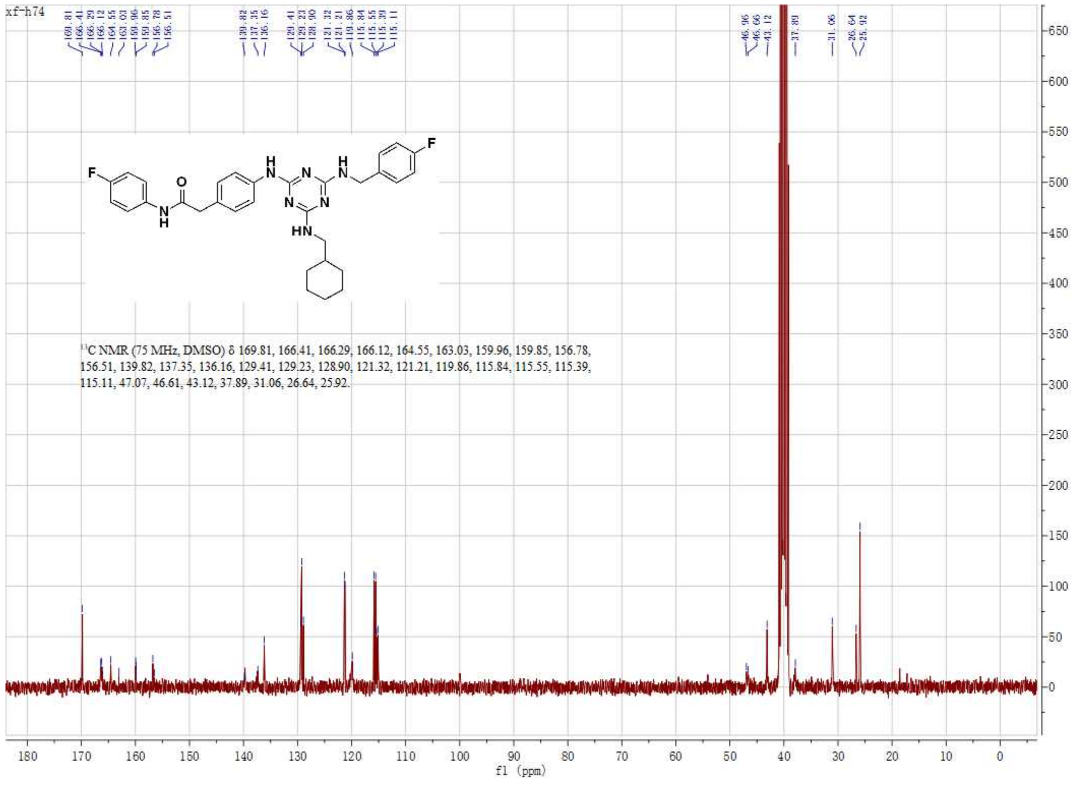

^{13}C-NMR of 8d

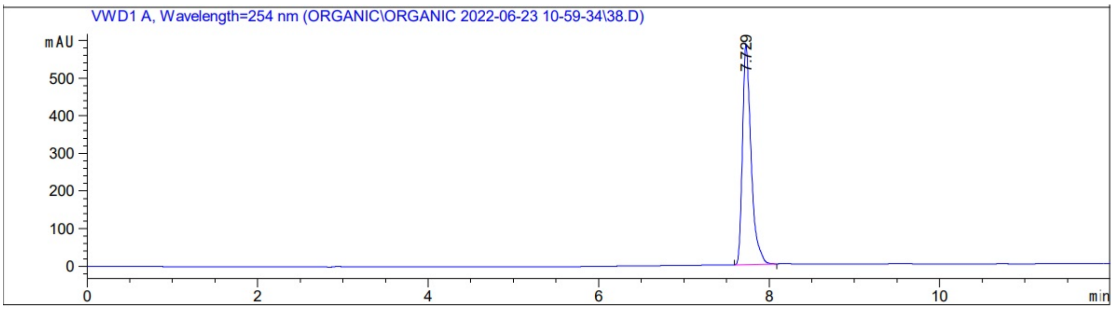

HPLC purity of 8d

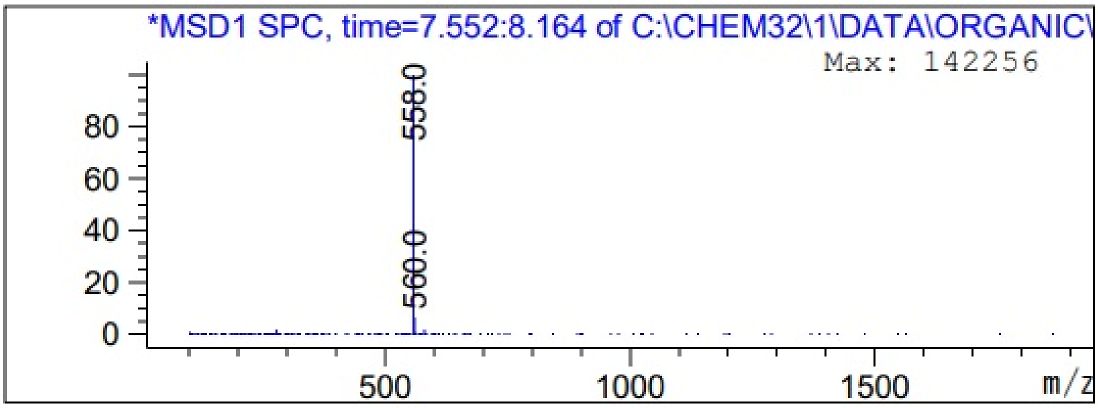

ESI-MS of 8d

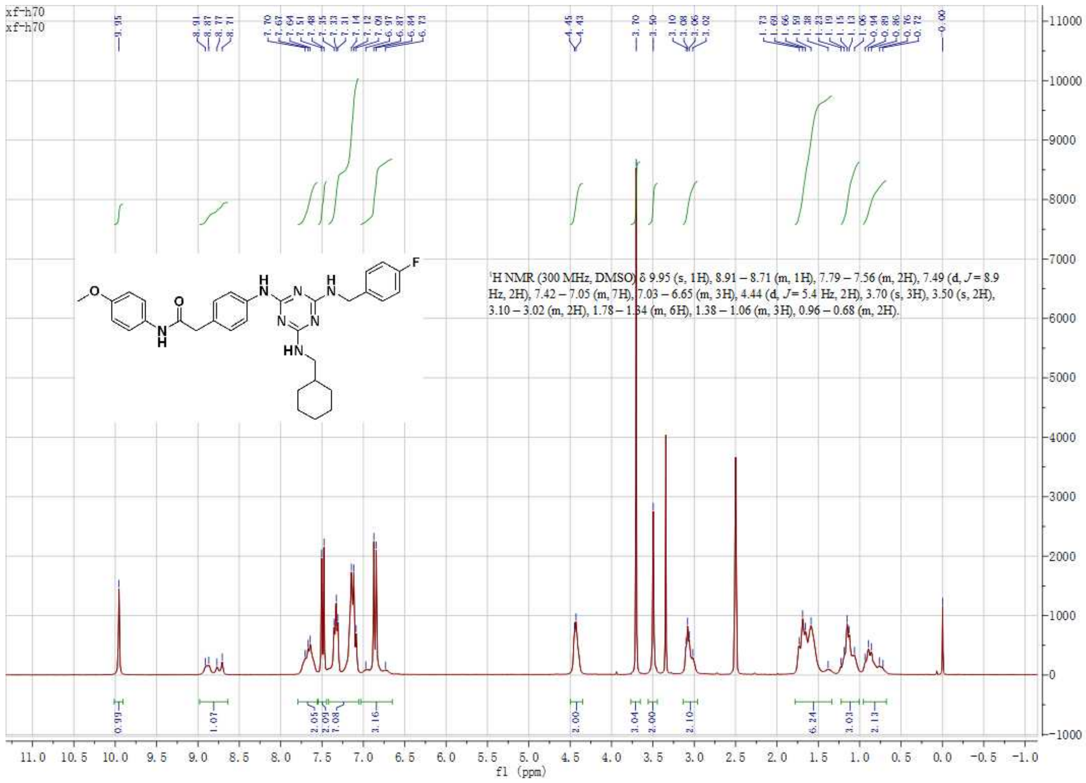

^{1}H-NMR of 8e

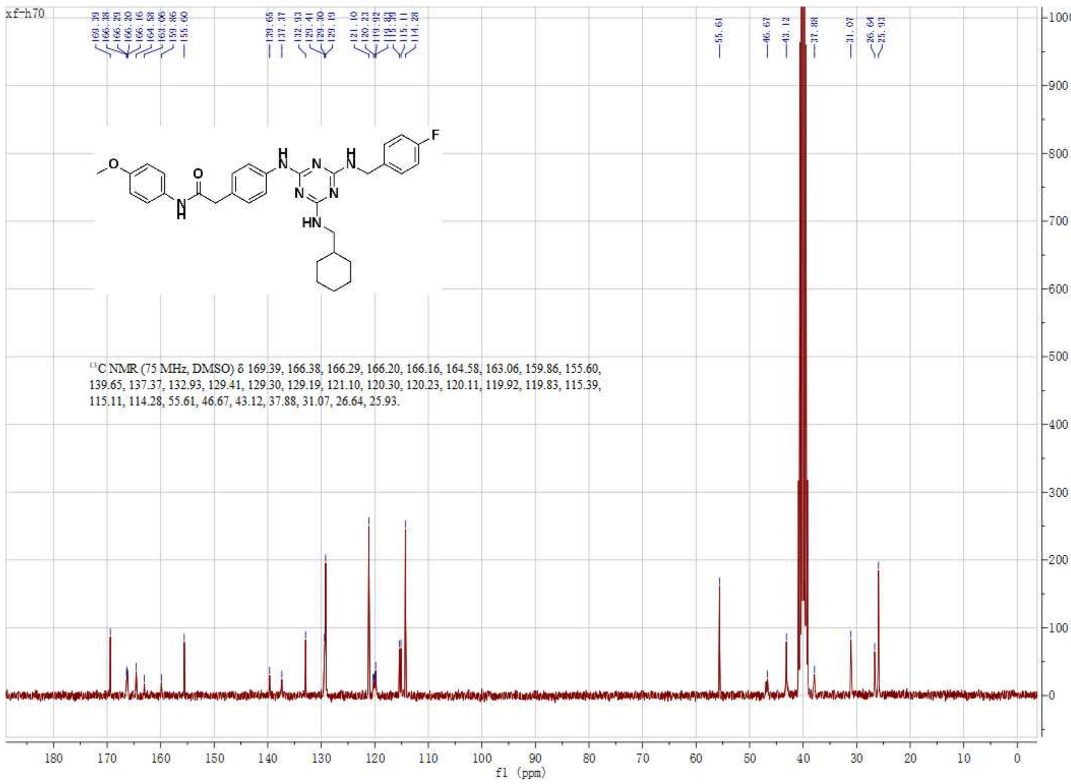

^{13}C-NMR of 8e

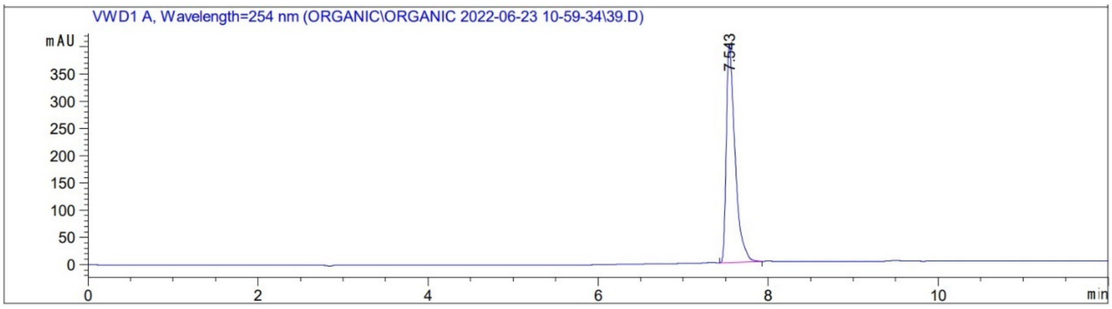

HPLC purity of 8e

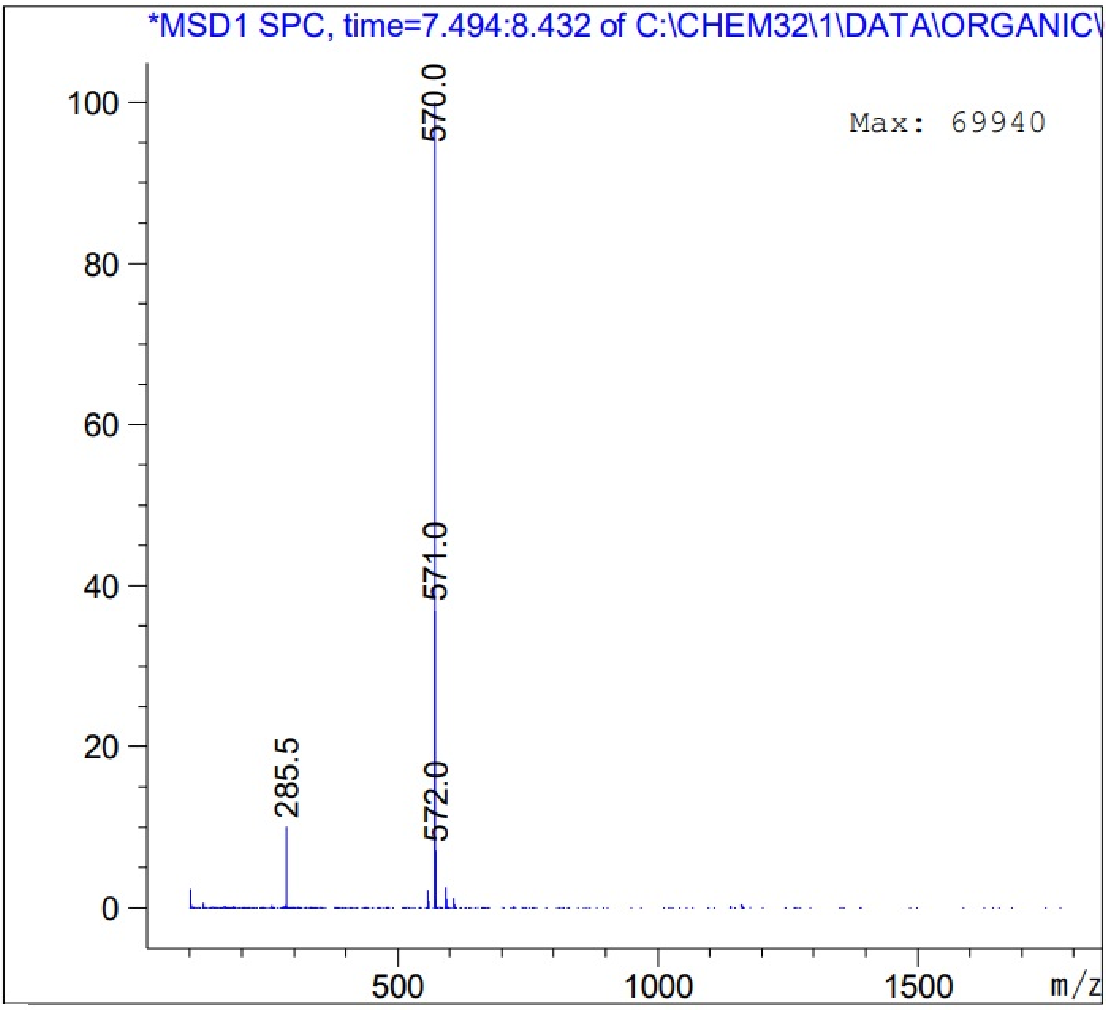

ESI-MS of 8e

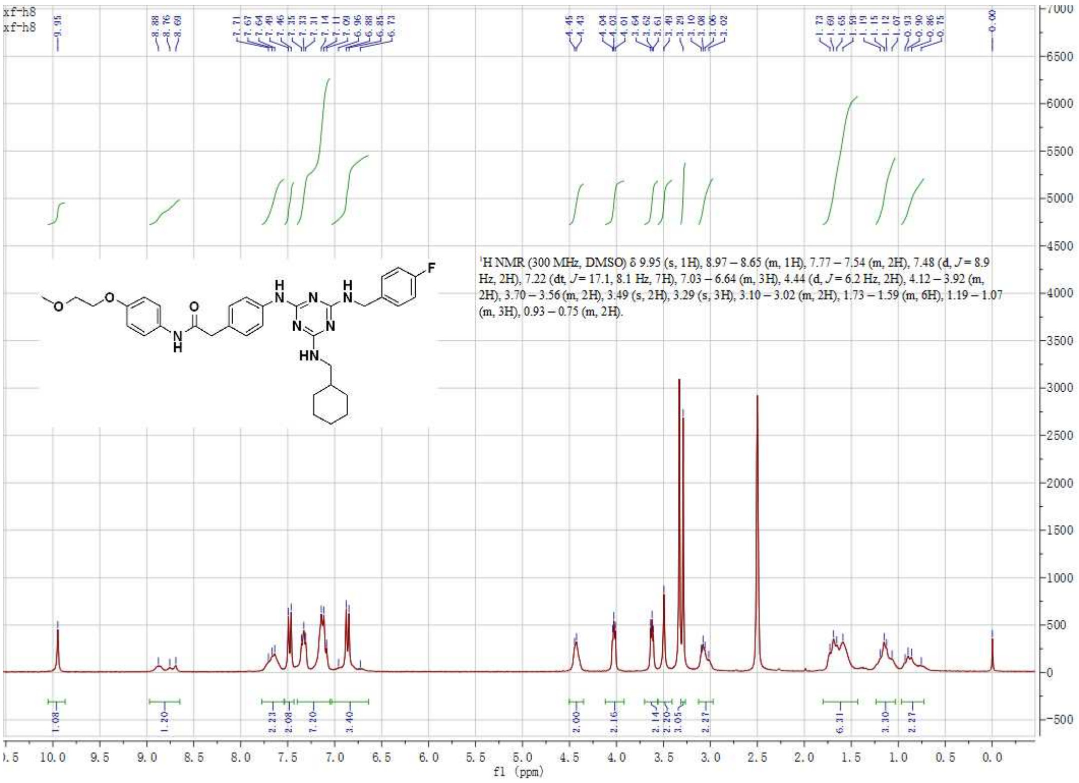

^{1}H-NMR of 8f

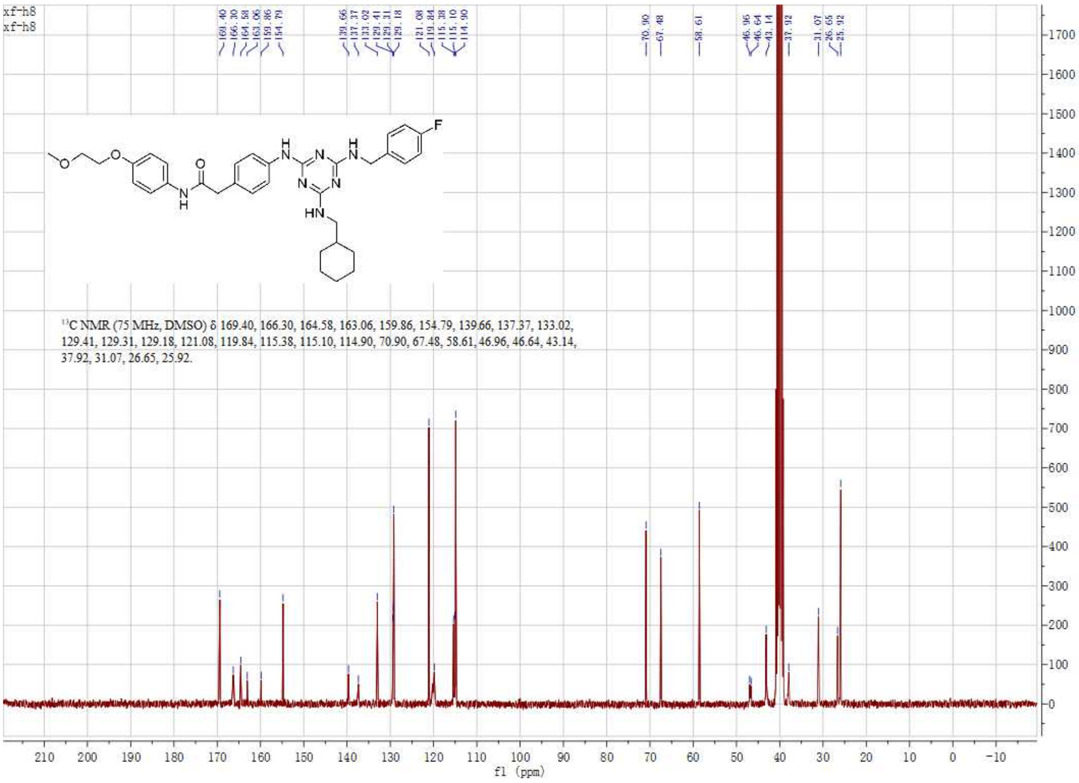

^{13}C-NMR of 8f

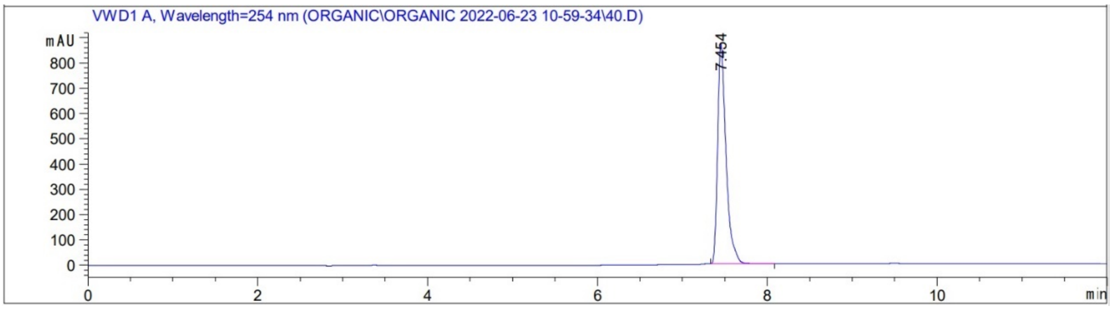

HPLC purity of 8f

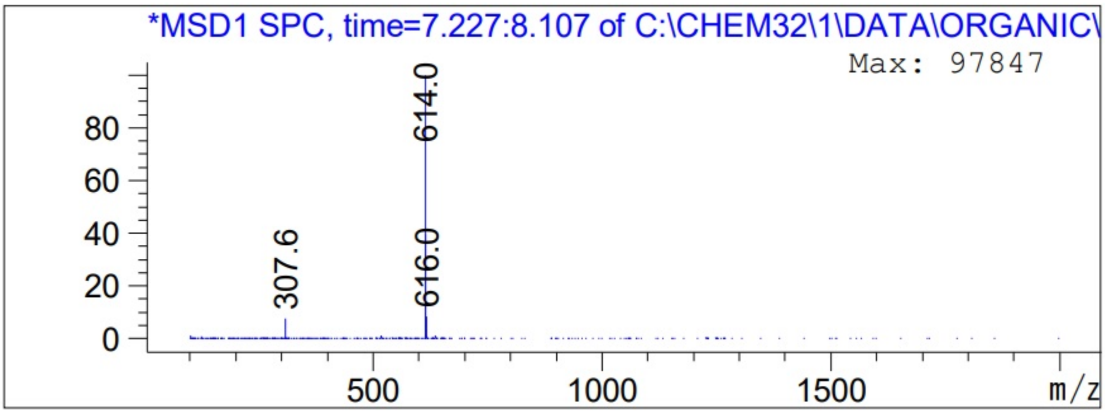

ESI-MS of 8f

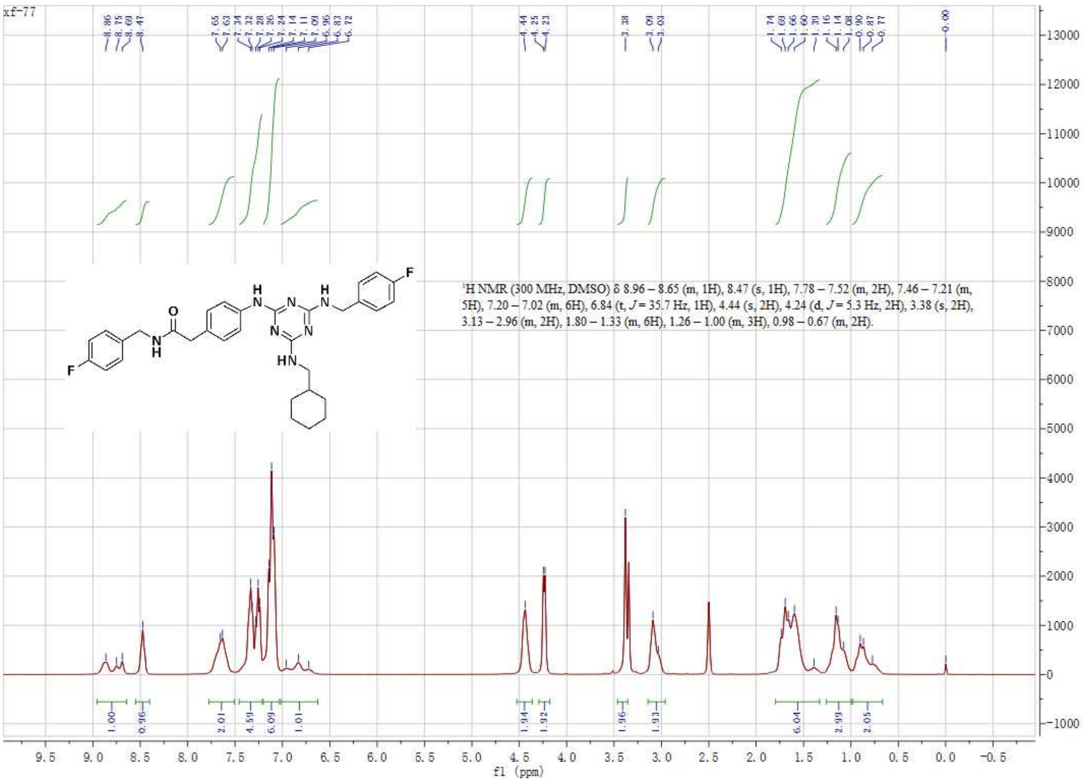

^{1}H-NMR of 8g

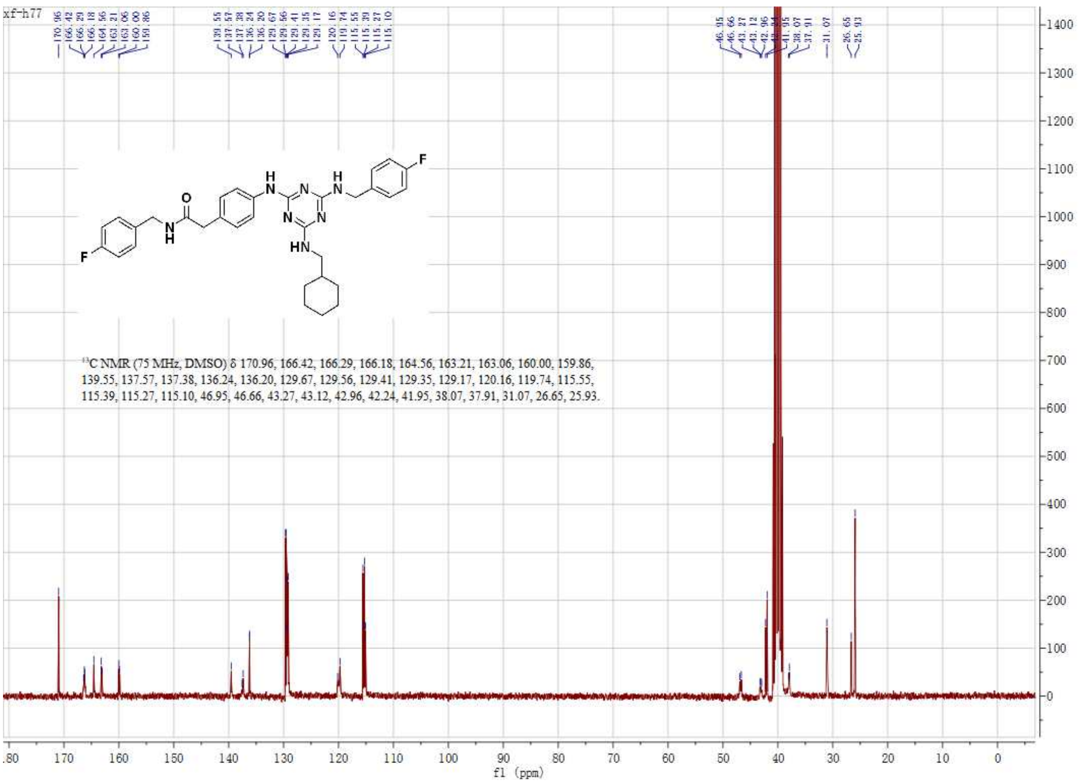

^{13}C-NMR of 8g

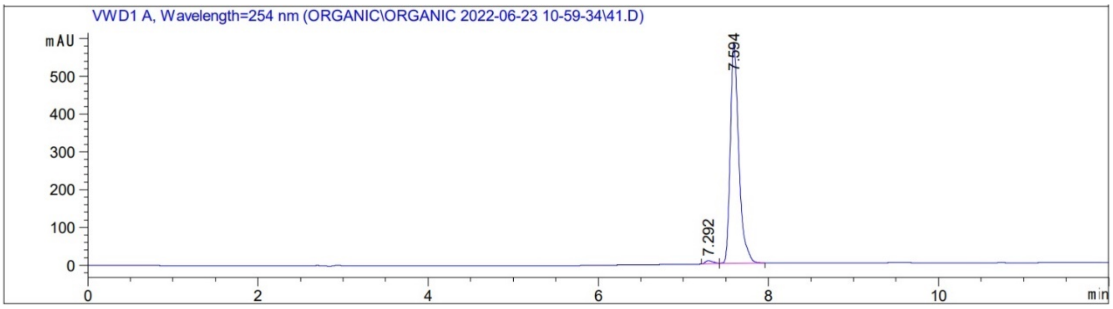

HPLC purity of 8g

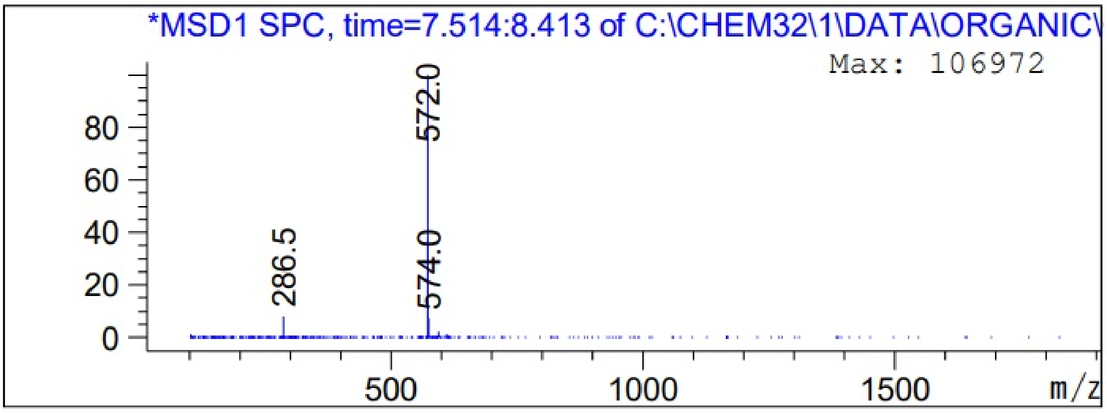

ESI-MS of 8g

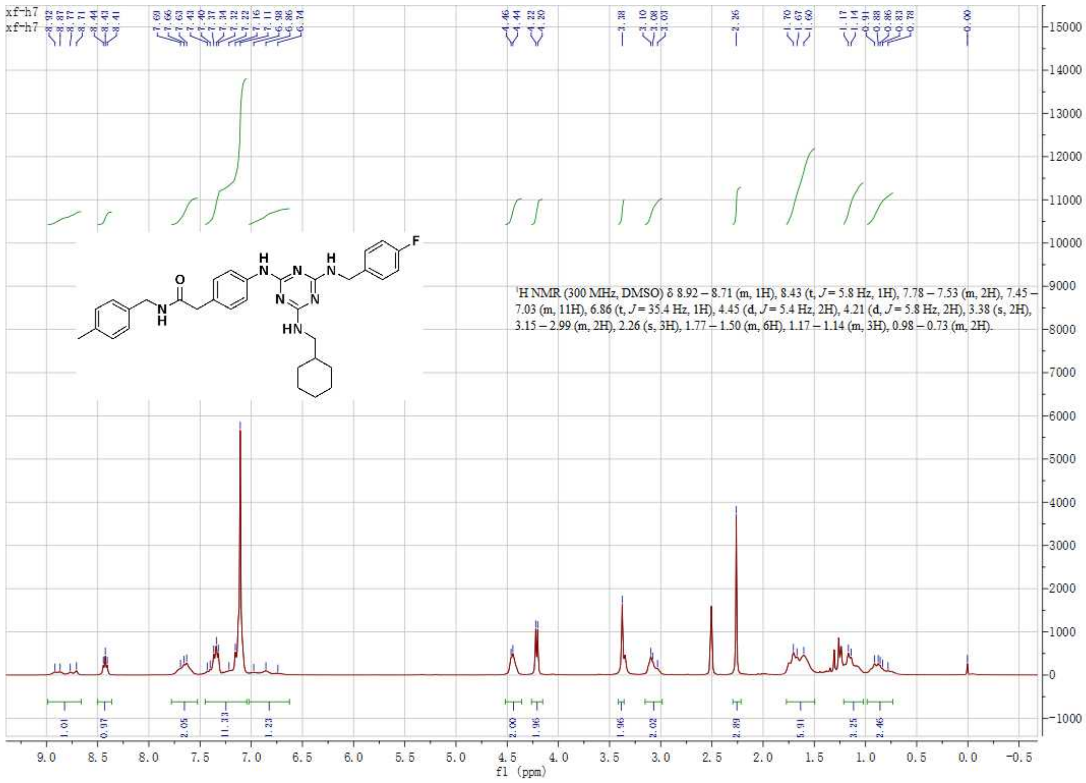

^{1}H-NMR of 8h

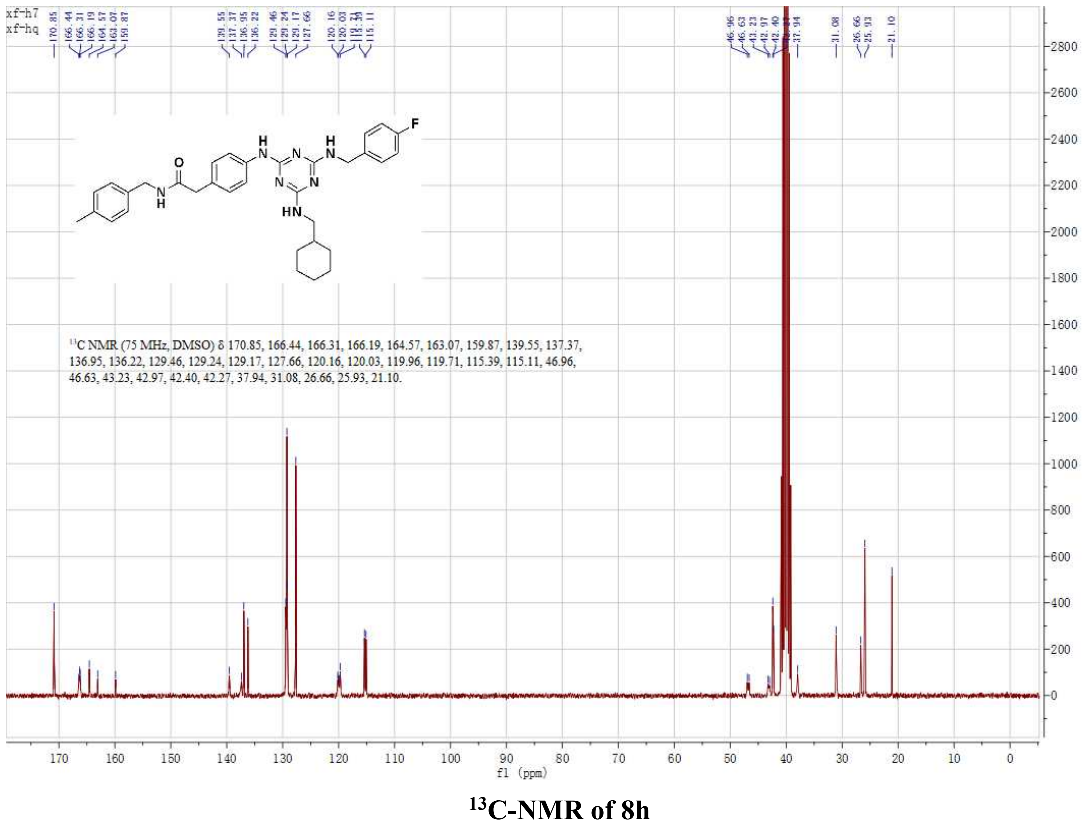

^{13}C-NMR of 8h

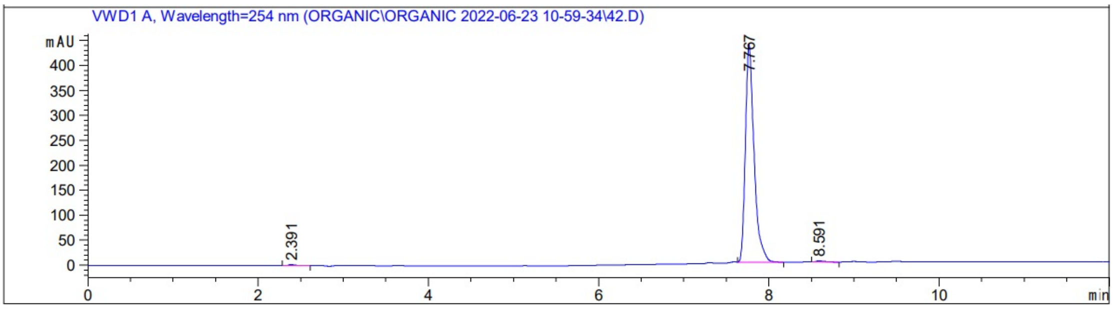

HPLC purity of 8h

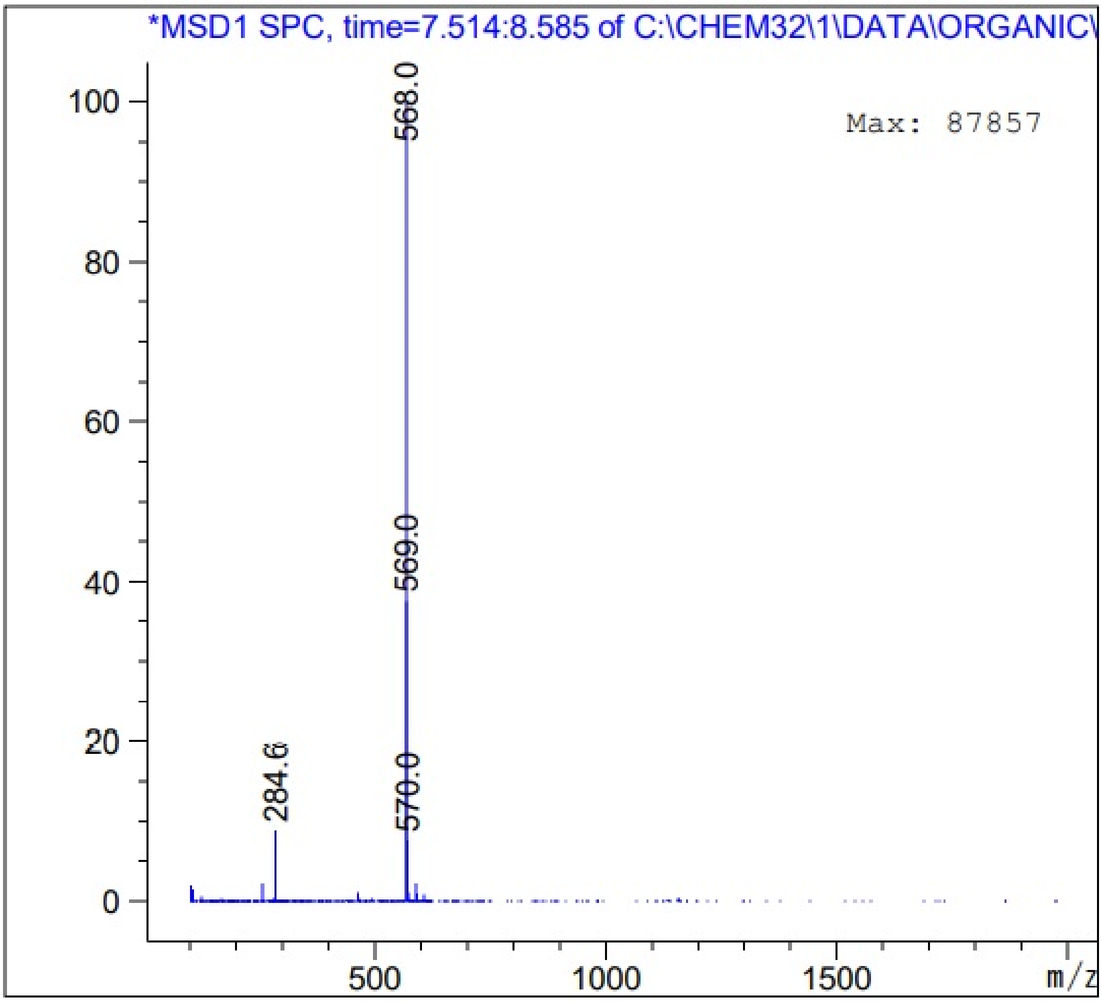

ESI-MS of 8h

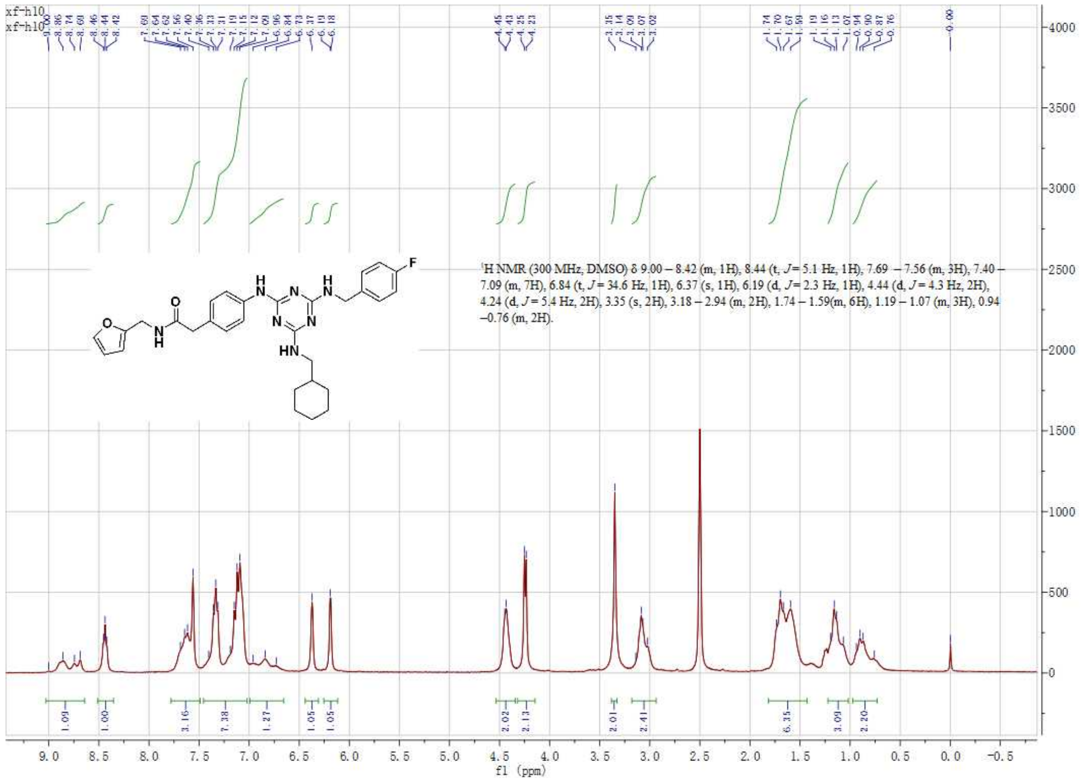

^{1}H-NMR of 8i

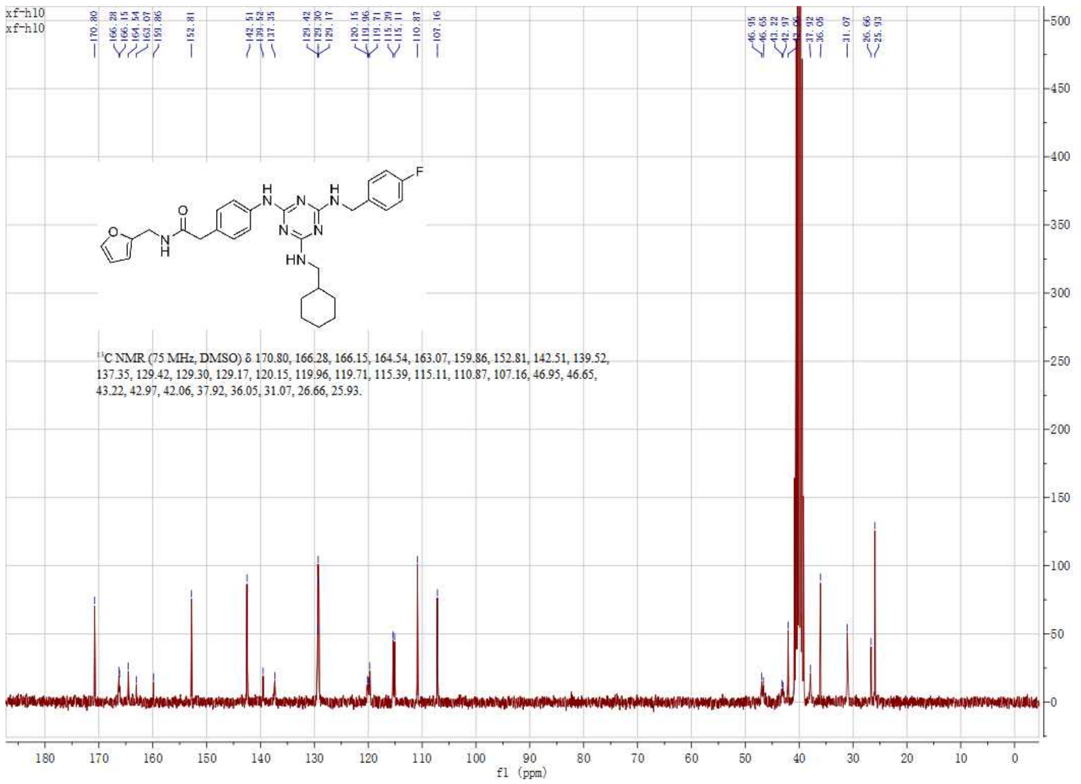

^{13}C-NMR of 8i

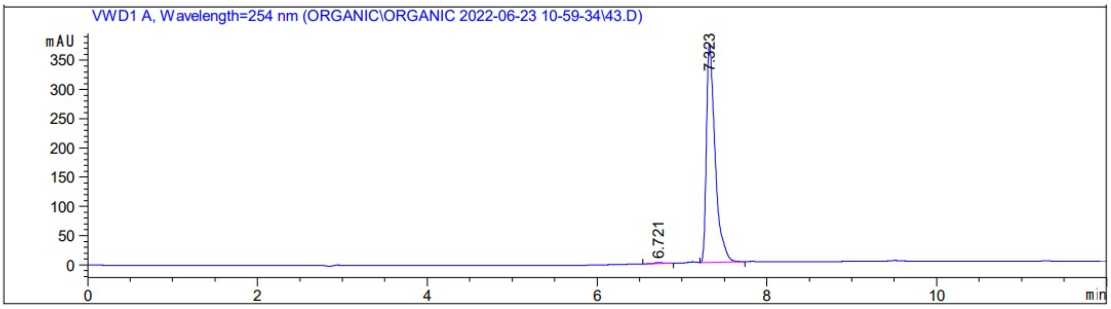

HPLC purity of 8i

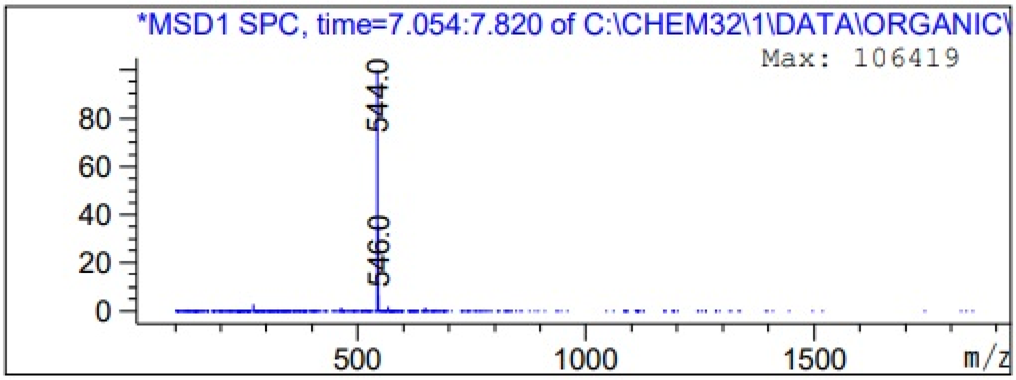

ESI-MS of 8i

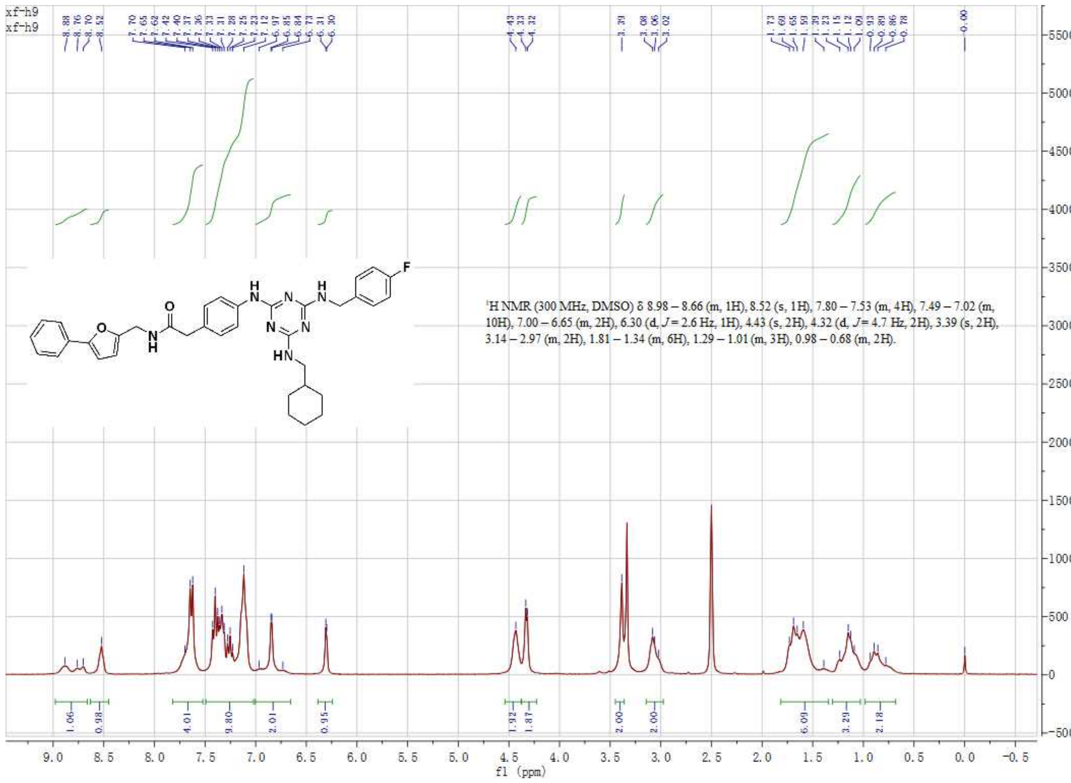

^{1}H-NMR of 8j

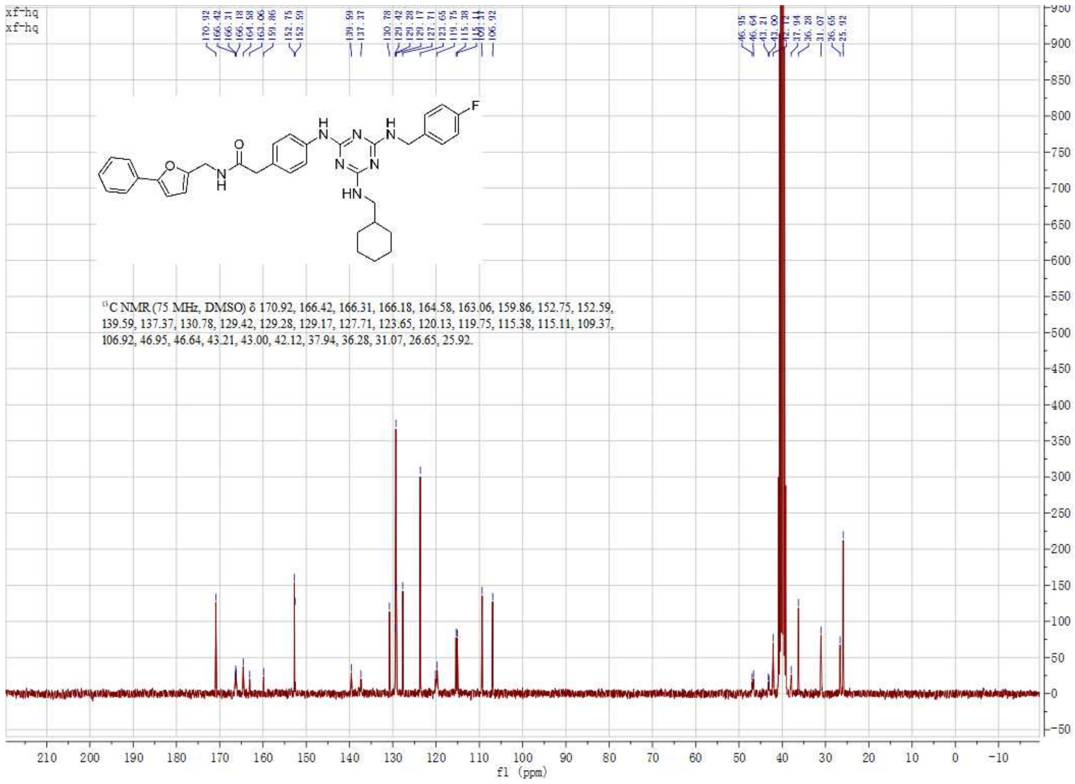

^{13}C-NMR of 8j

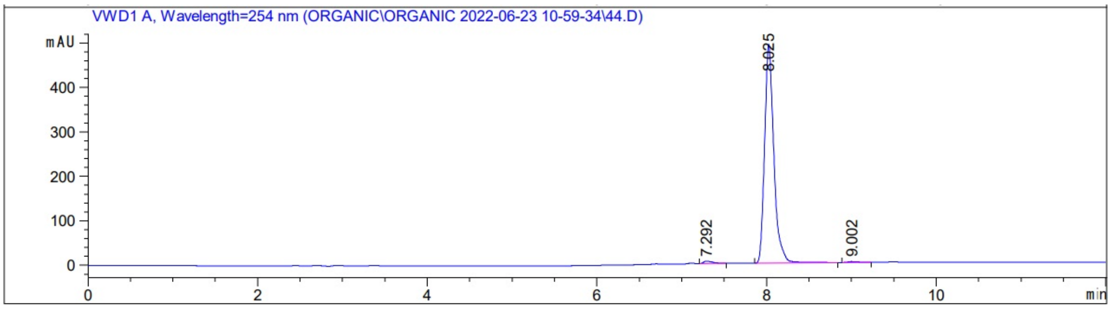

HPLC purity of 8j

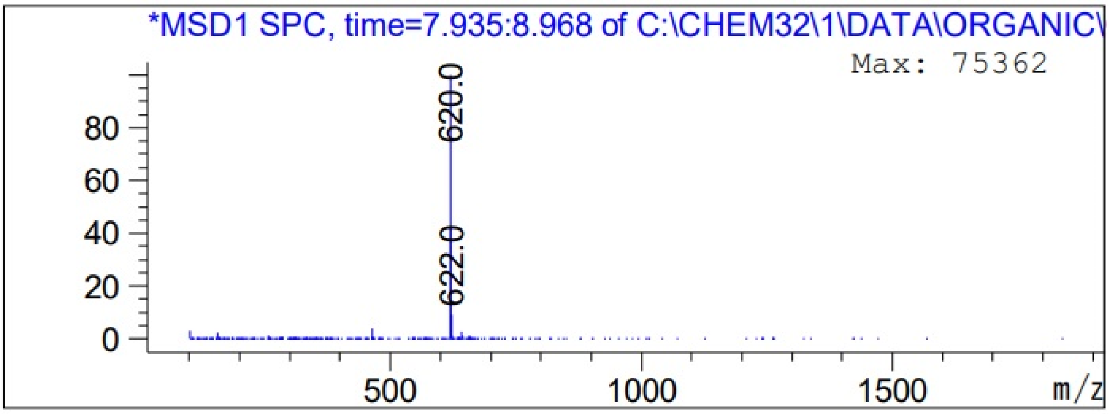

ESI-MS of 8j

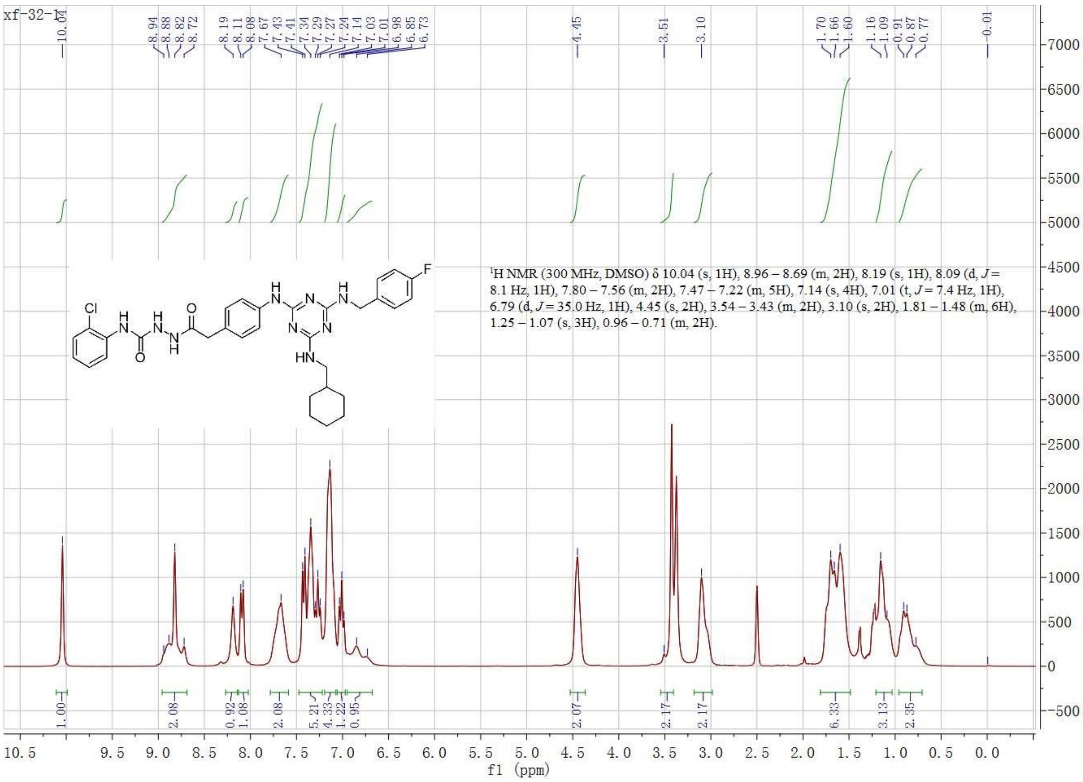

^{1}H-NMR of 9a

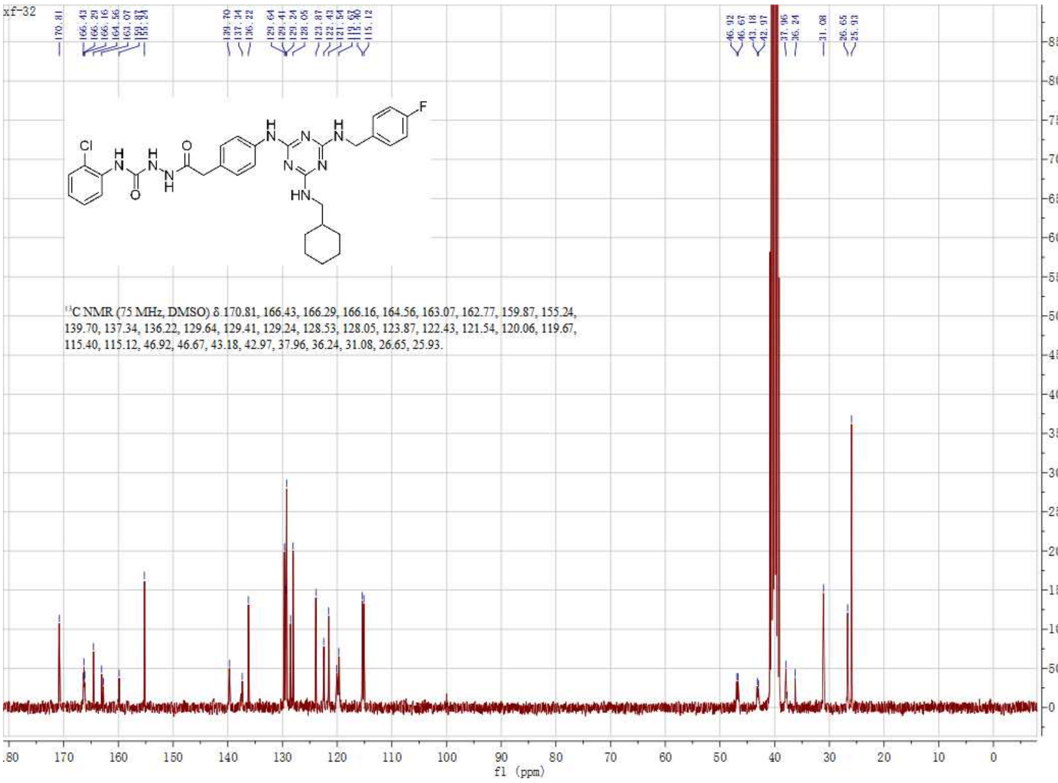

^{13}C-NMR of 9a

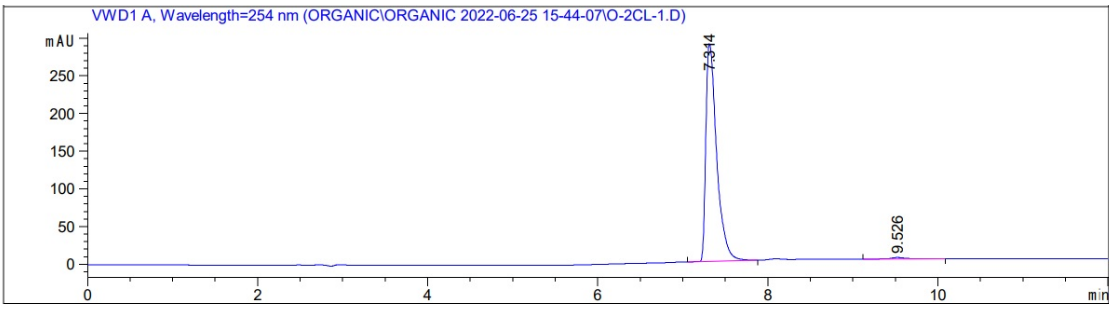

HPLC purity of 9a

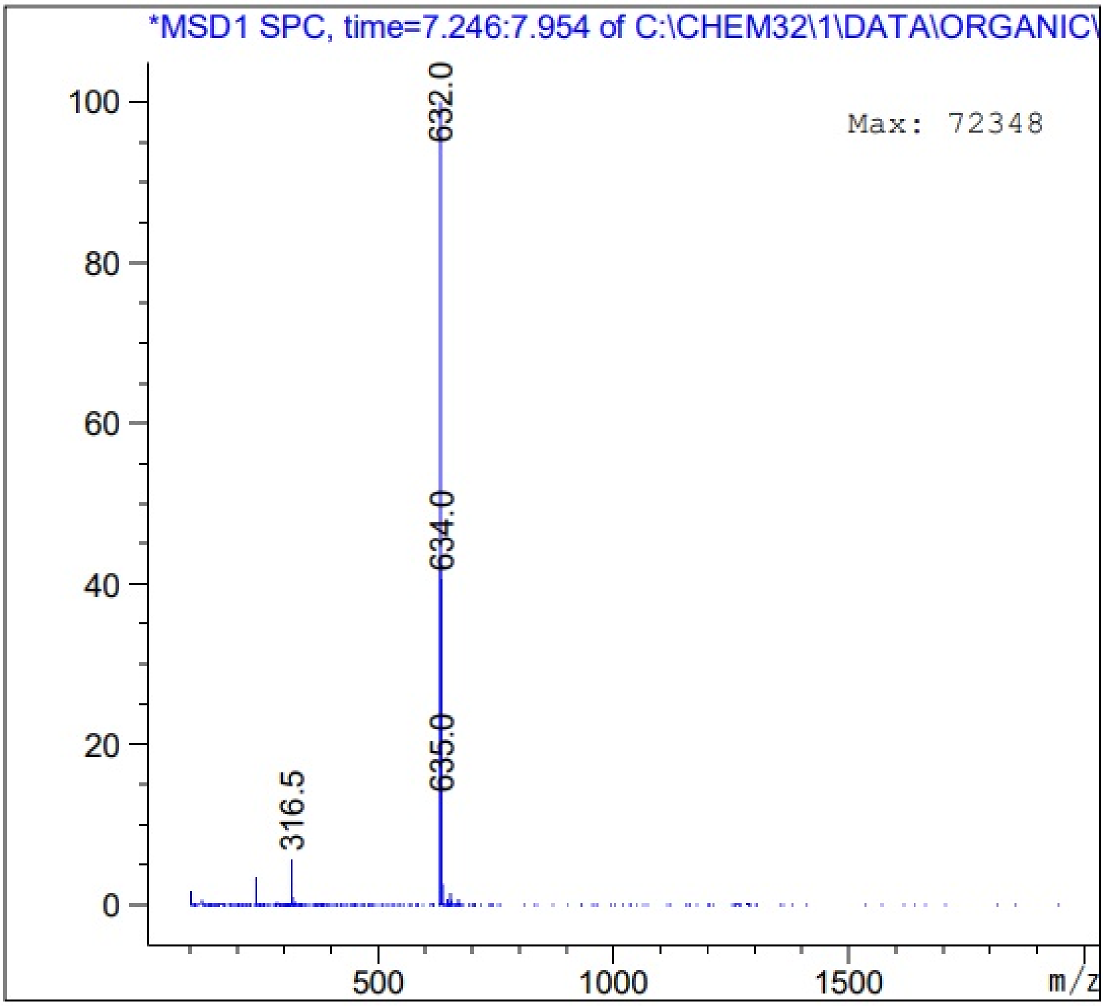

ESI-MS of 9a

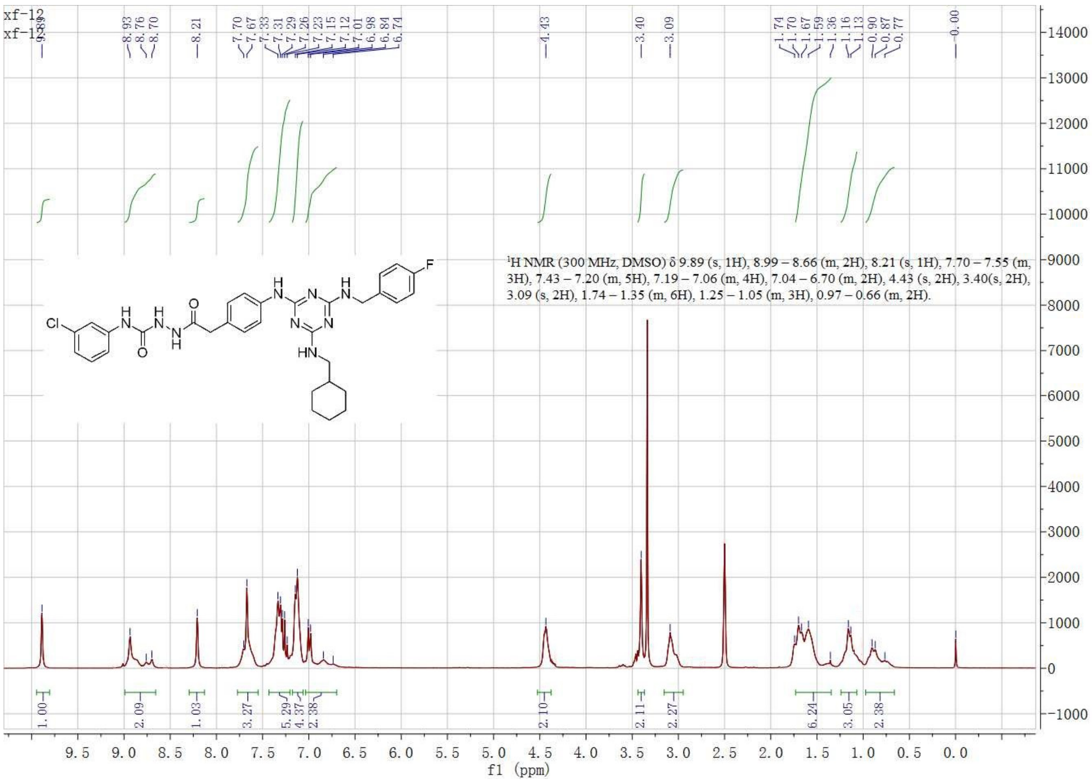

^{1}H-NMR of 9b

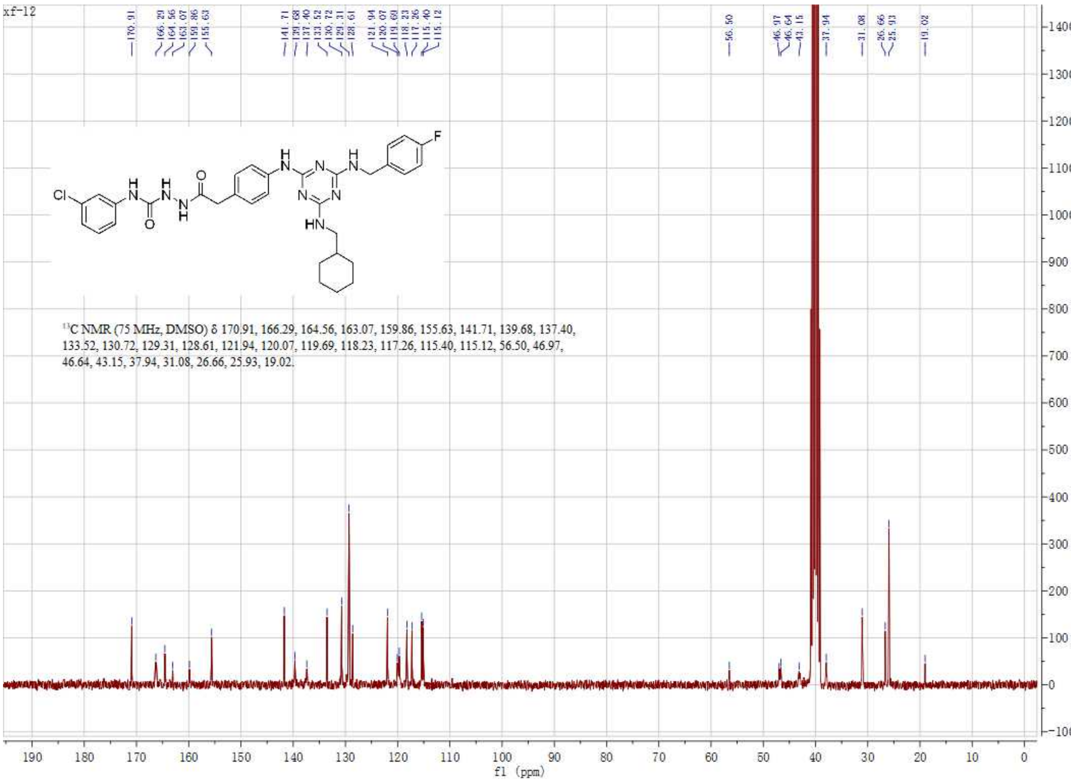

^{13}C-NMR of 9b

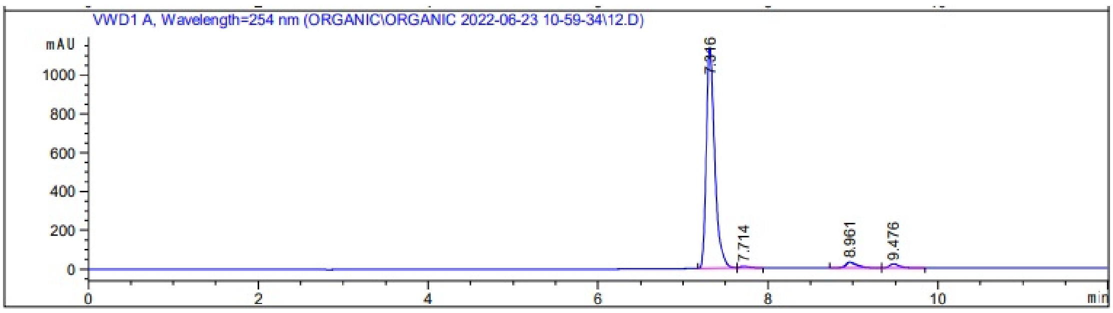

HPLC purity of 9b

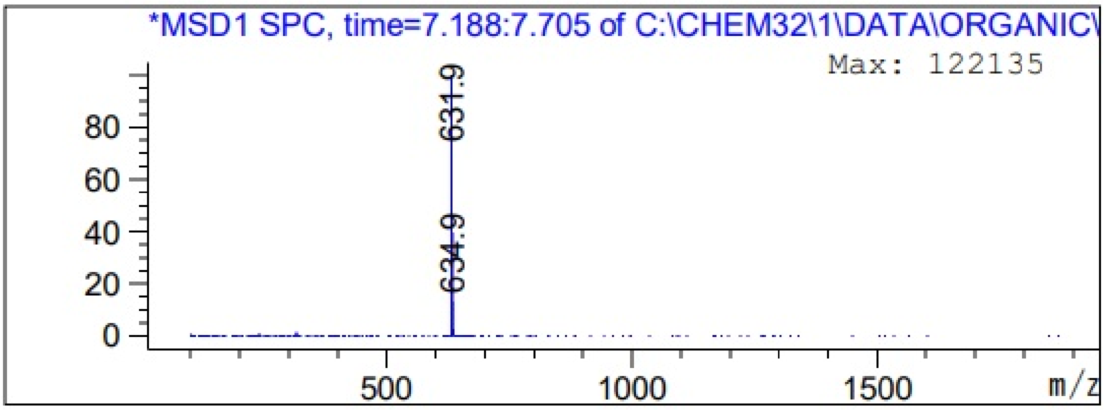

ESI-MS of 9b

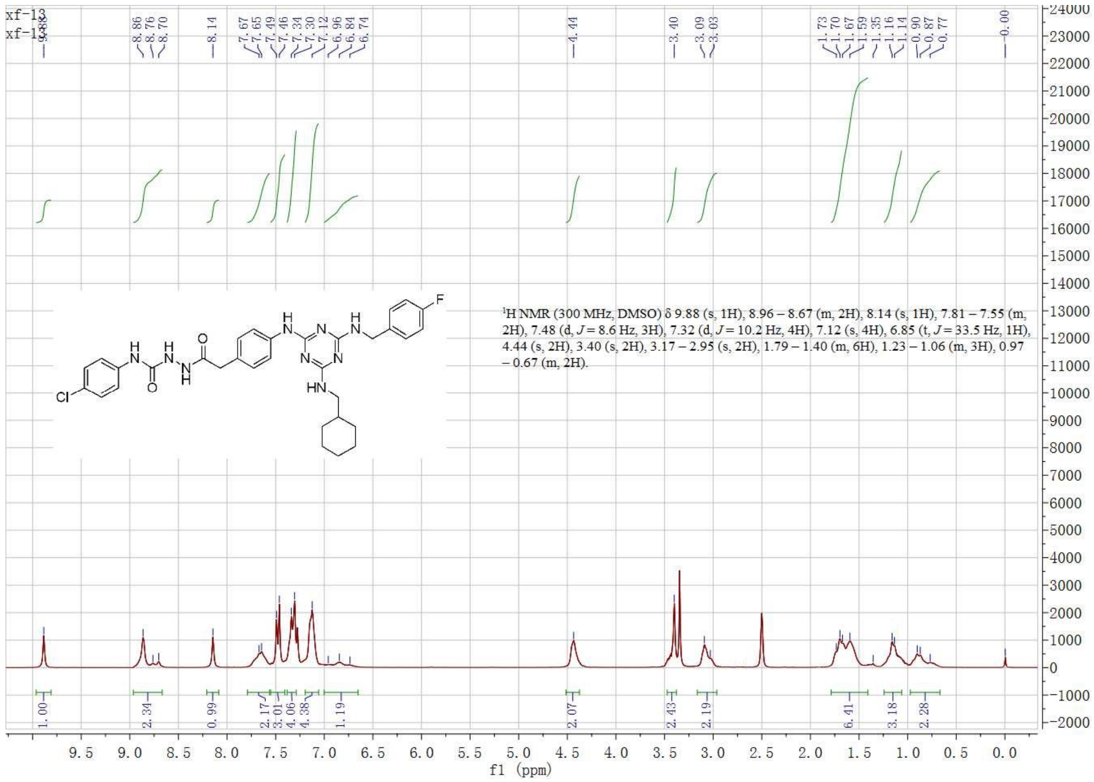

^{1}H-NMR of 9c

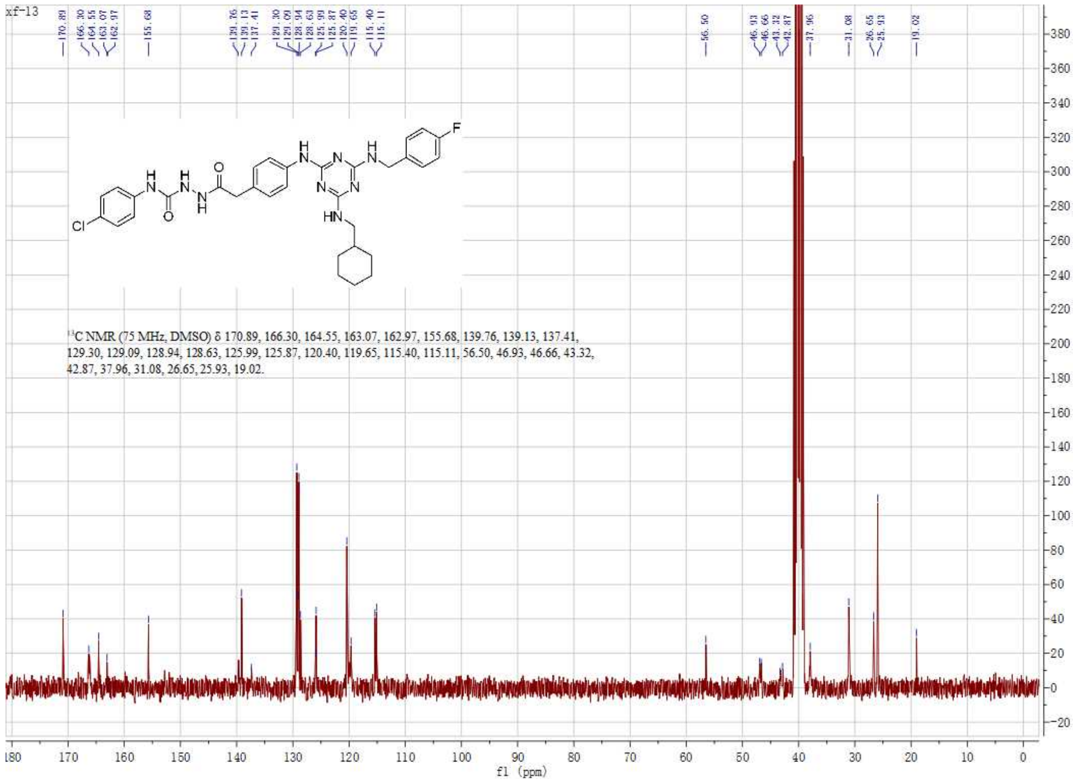

^{13}C-NMR of 9c

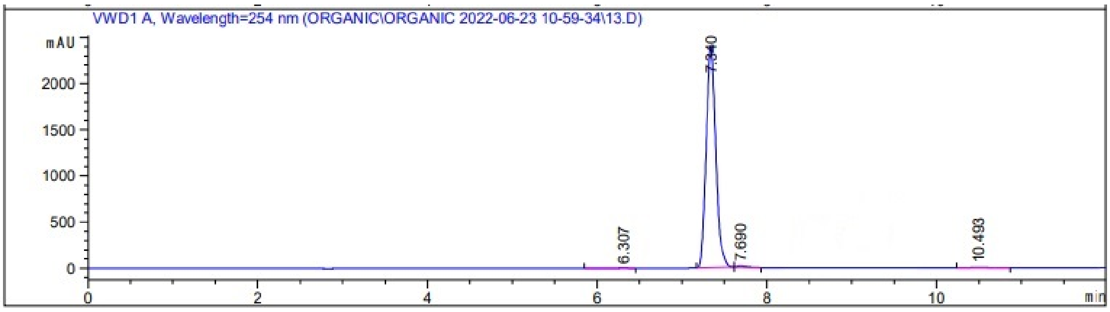

HPLC purity of 9c

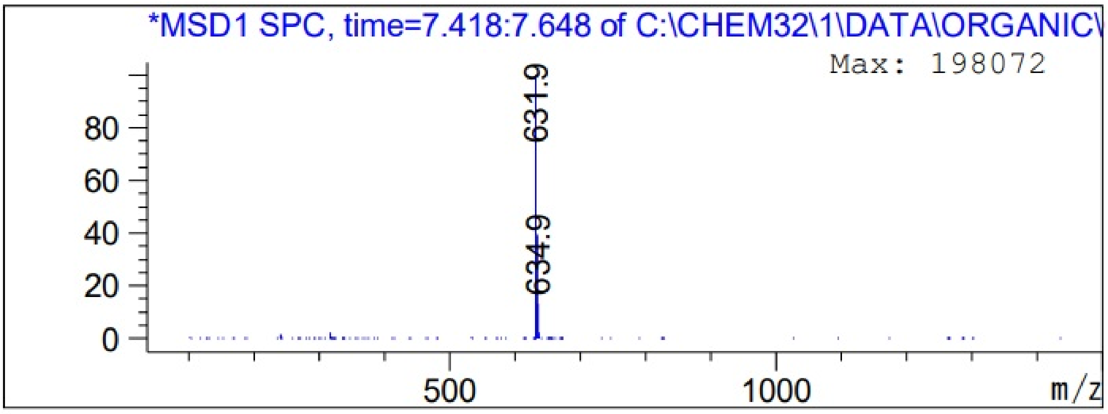

ESI-MS of 9c

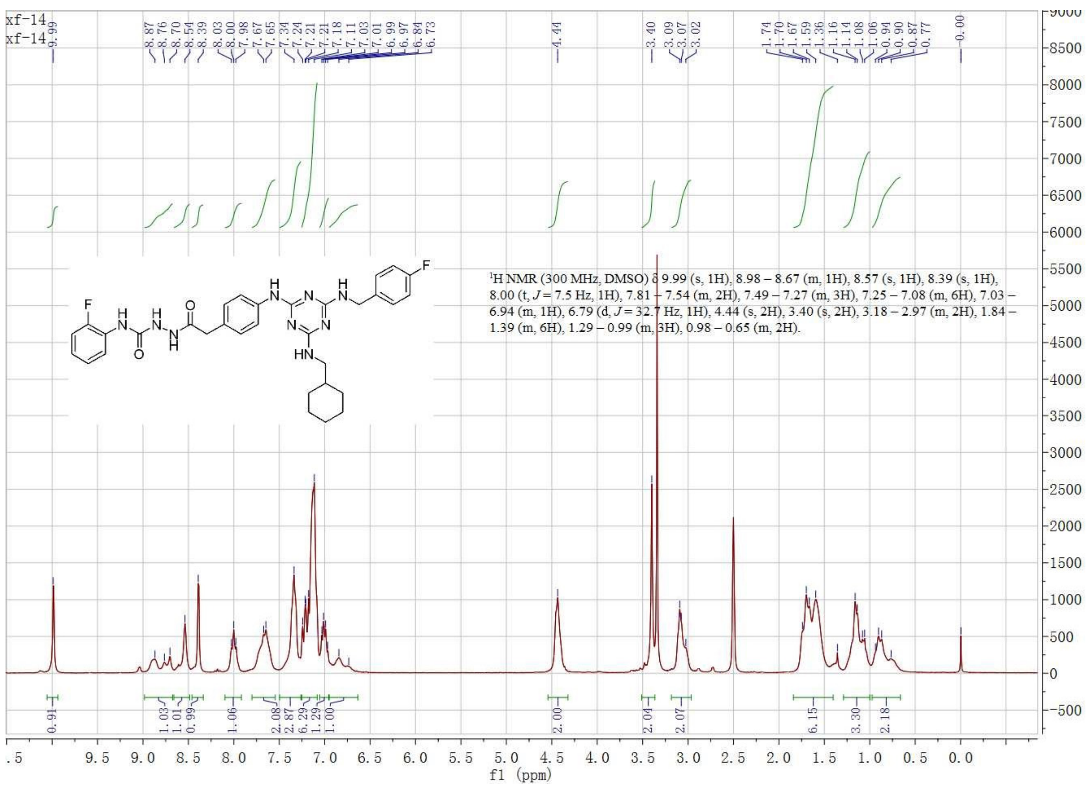

^{1}H-NMR of 9d

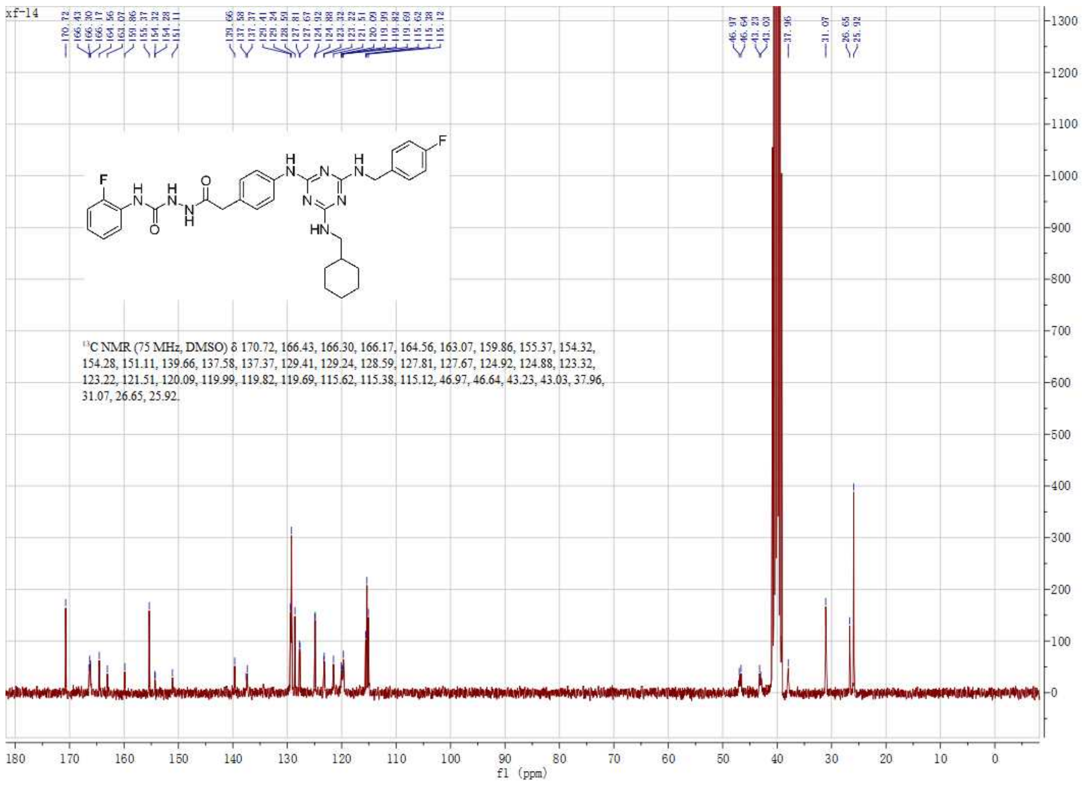

^{13}C-NMR of 9d

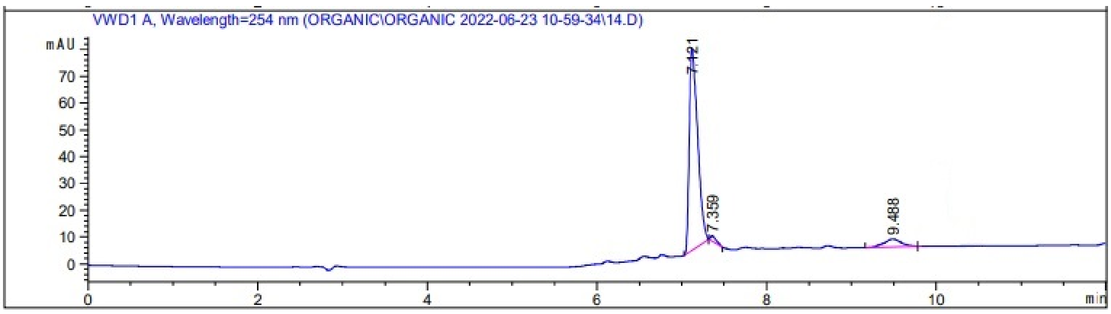

HPLC purity of 9d

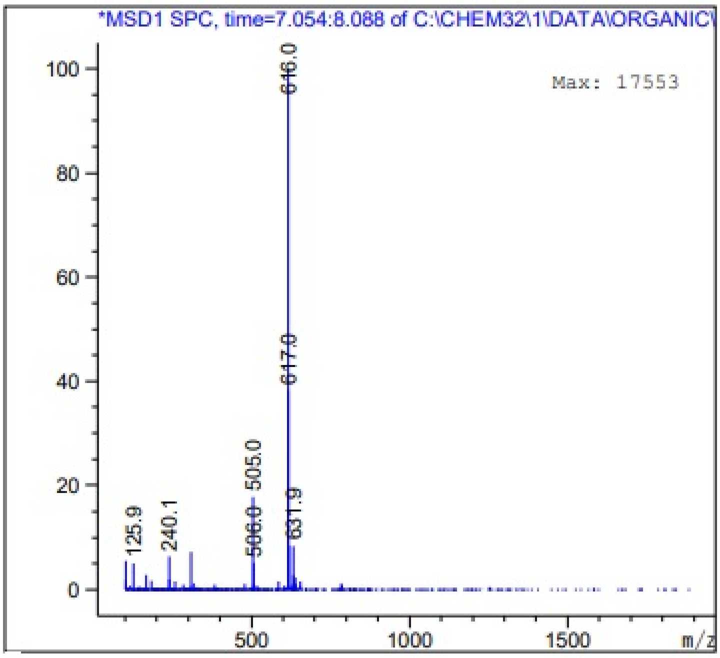

ESI-MS of 9d

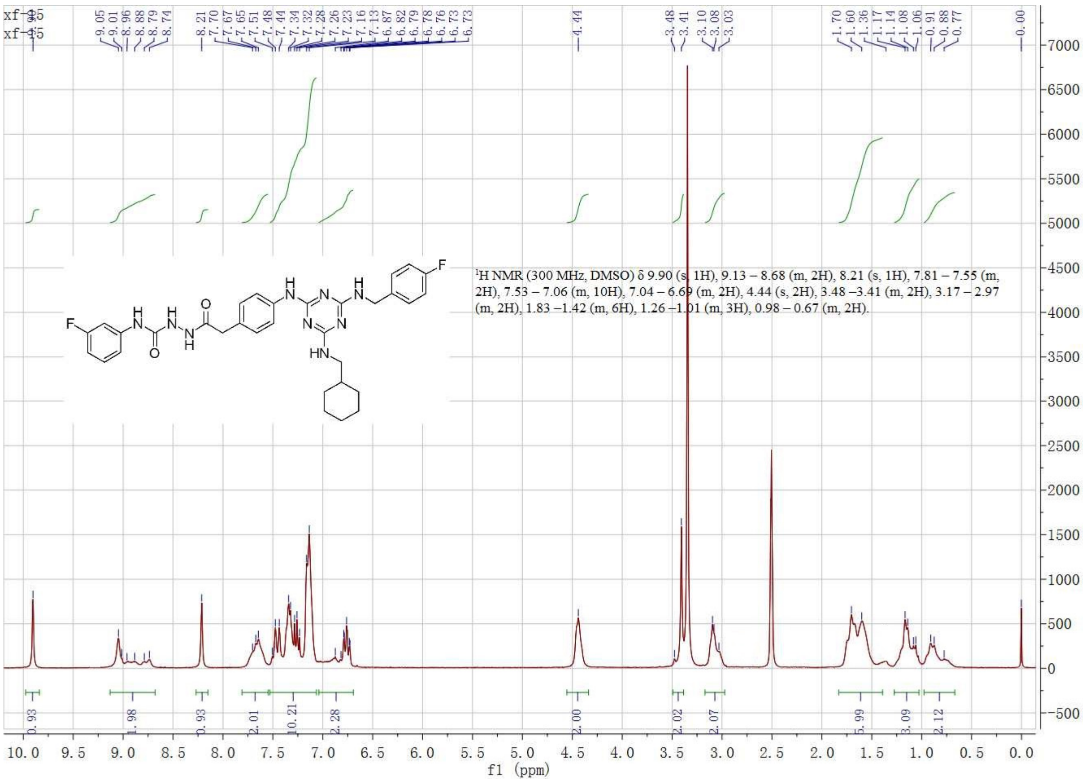

^{1}H-NMR of 9e

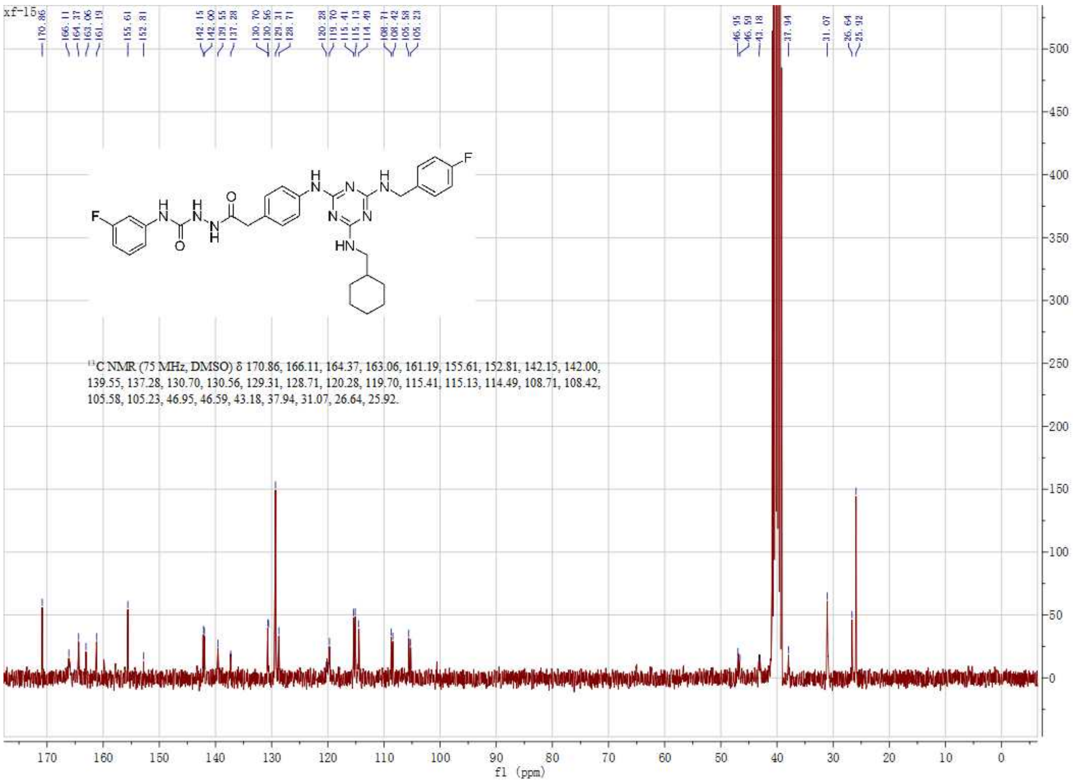

^{13}C-NMR of 9e

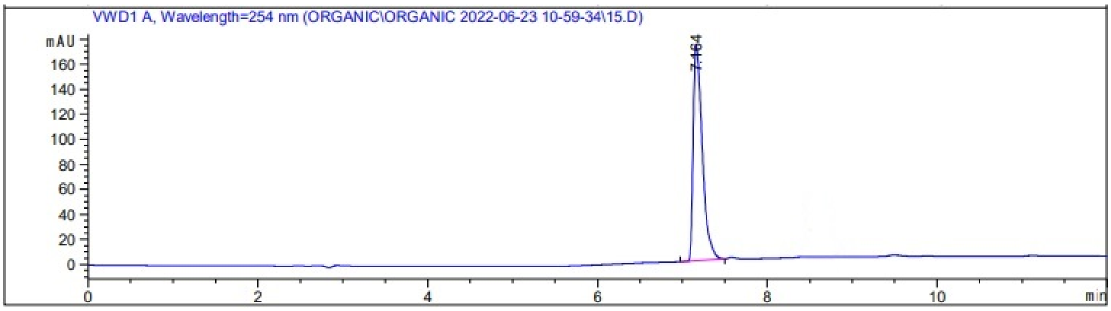

HPLC purity of 9e

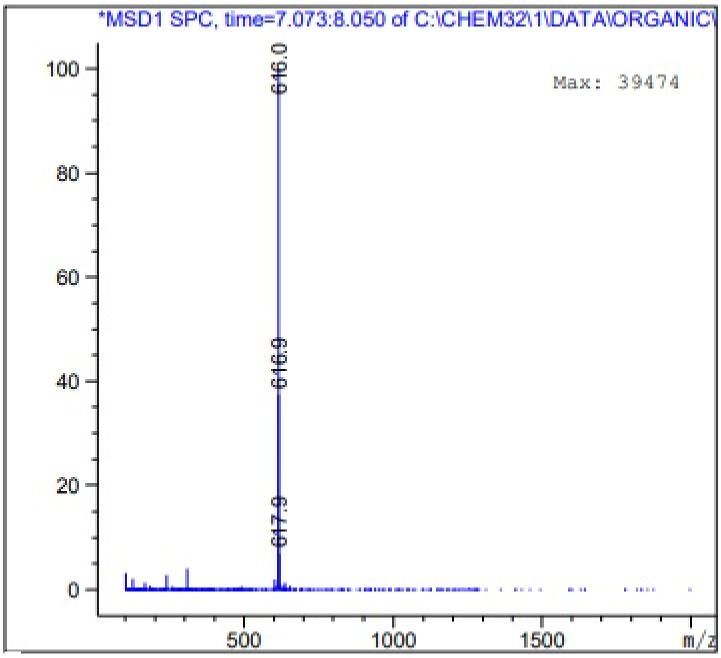

ESI-MS of 9e

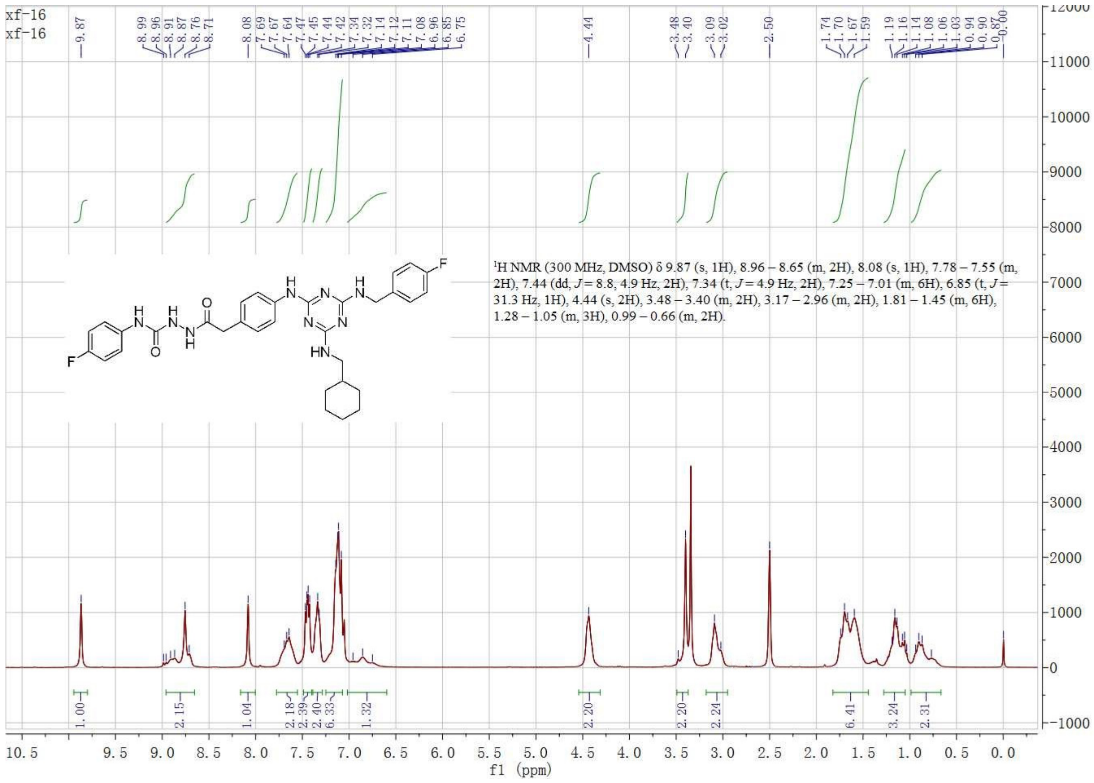

^{1}H-NMR of 9f

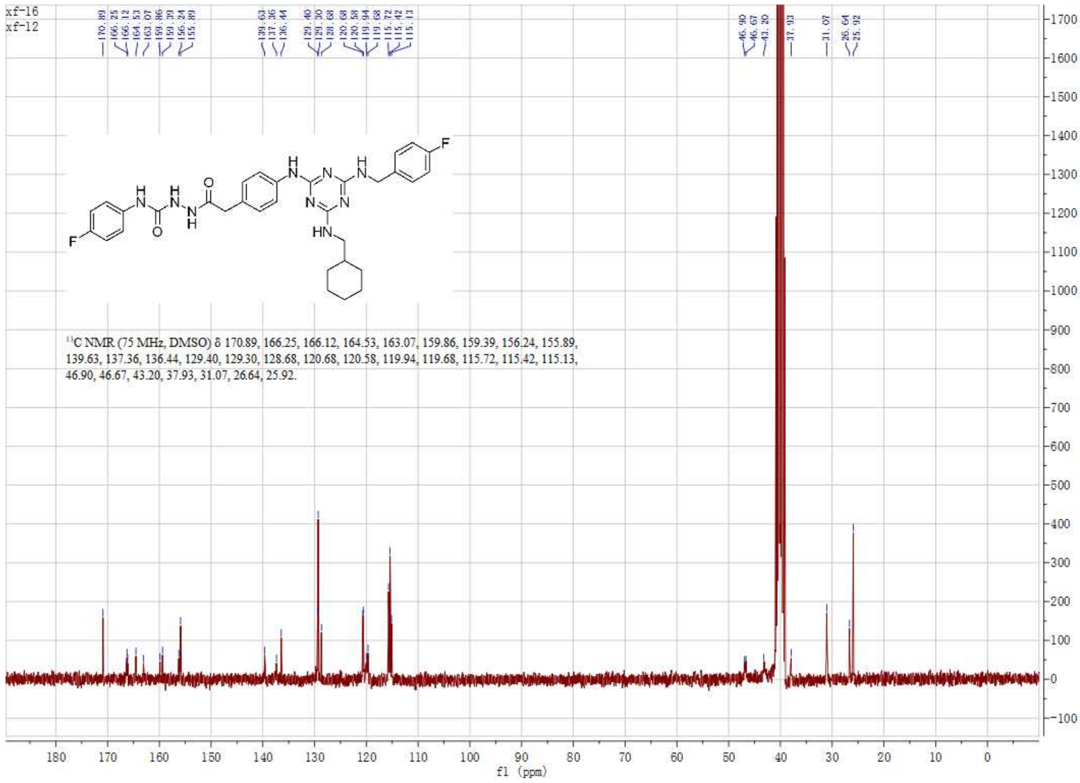

^{13}C-NMR of 9f

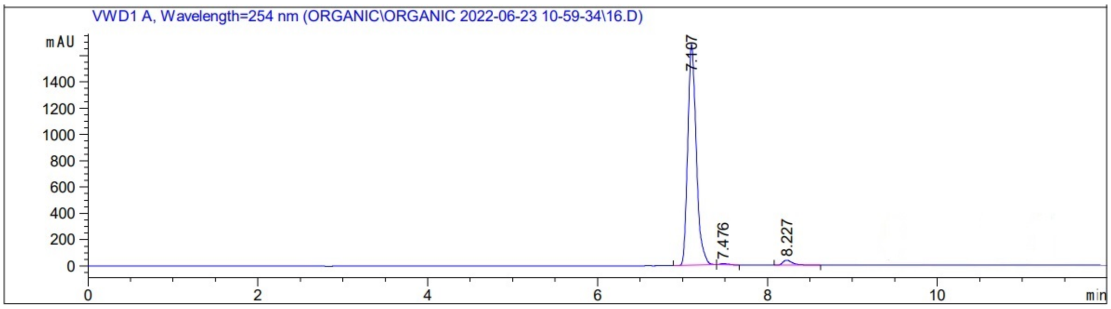

HPLC purity of 9f

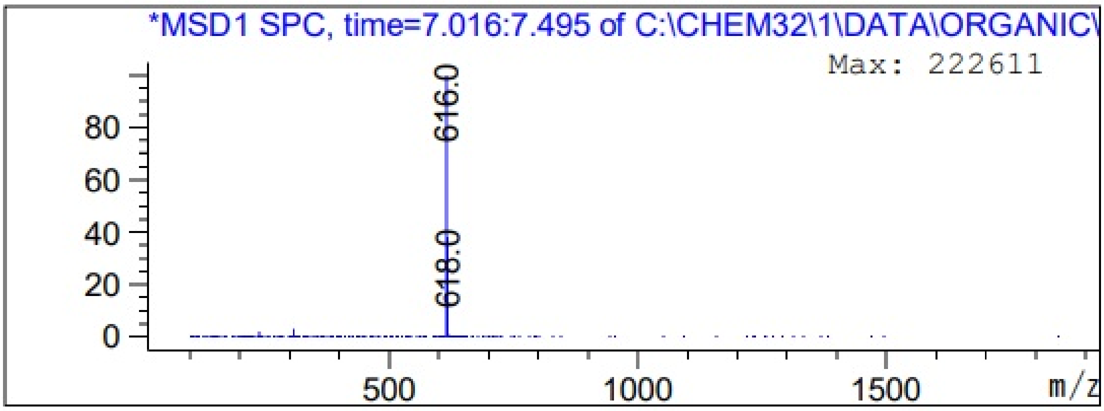

ESI-MS of 9f

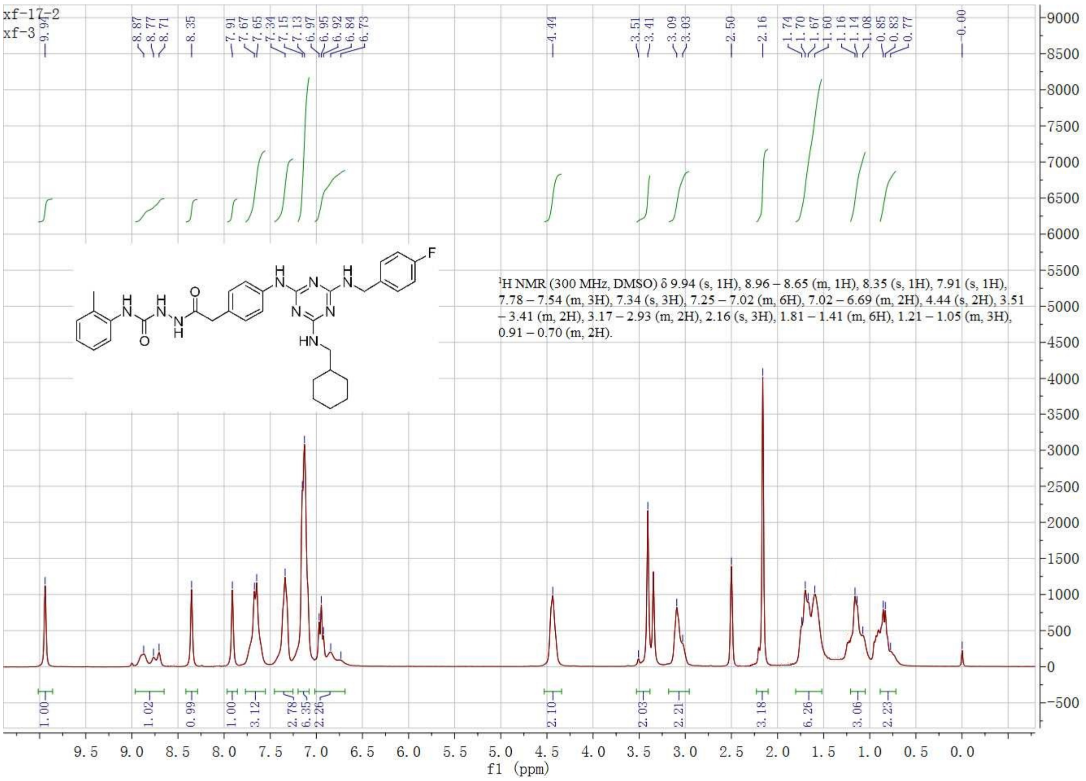

^{1}H-NMR of 9g

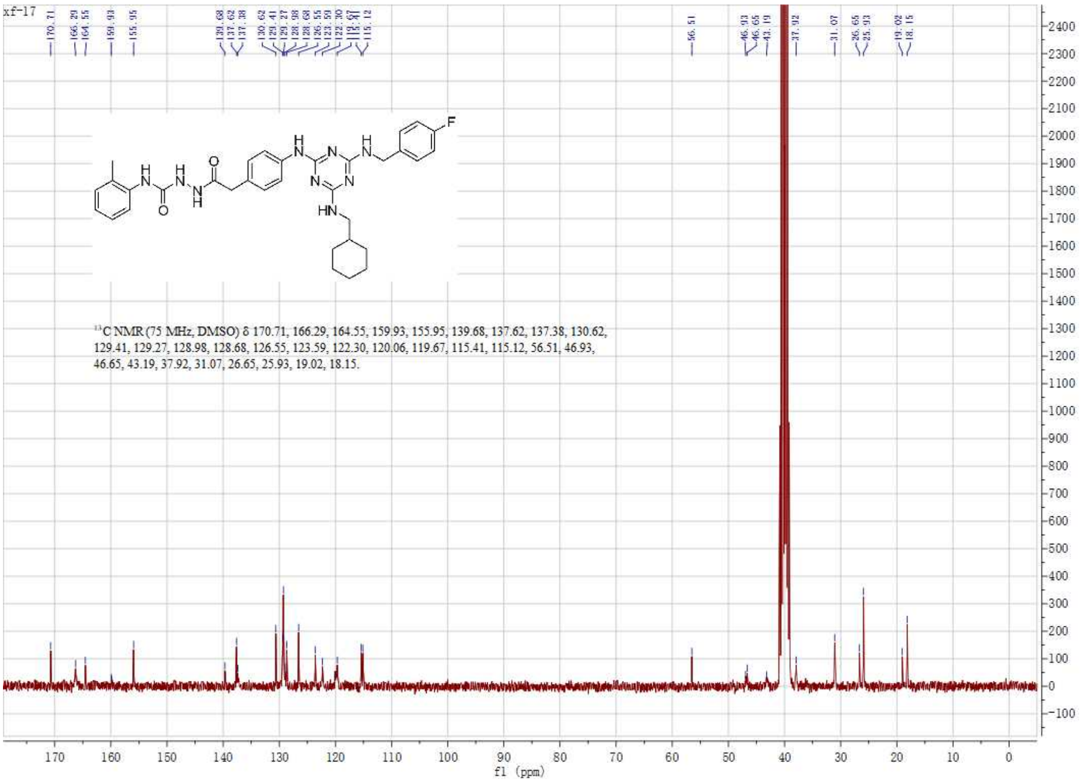

^{13}C-NMR of 9g

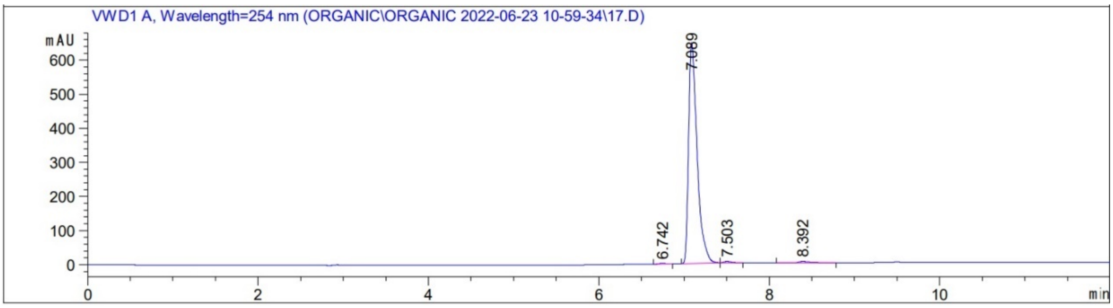

HPLC purity of 9g

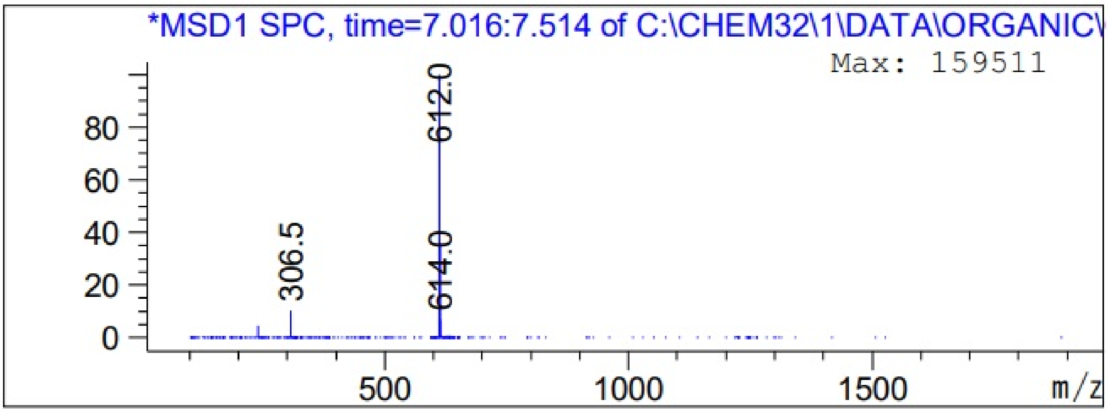

ESI-MS of 9g

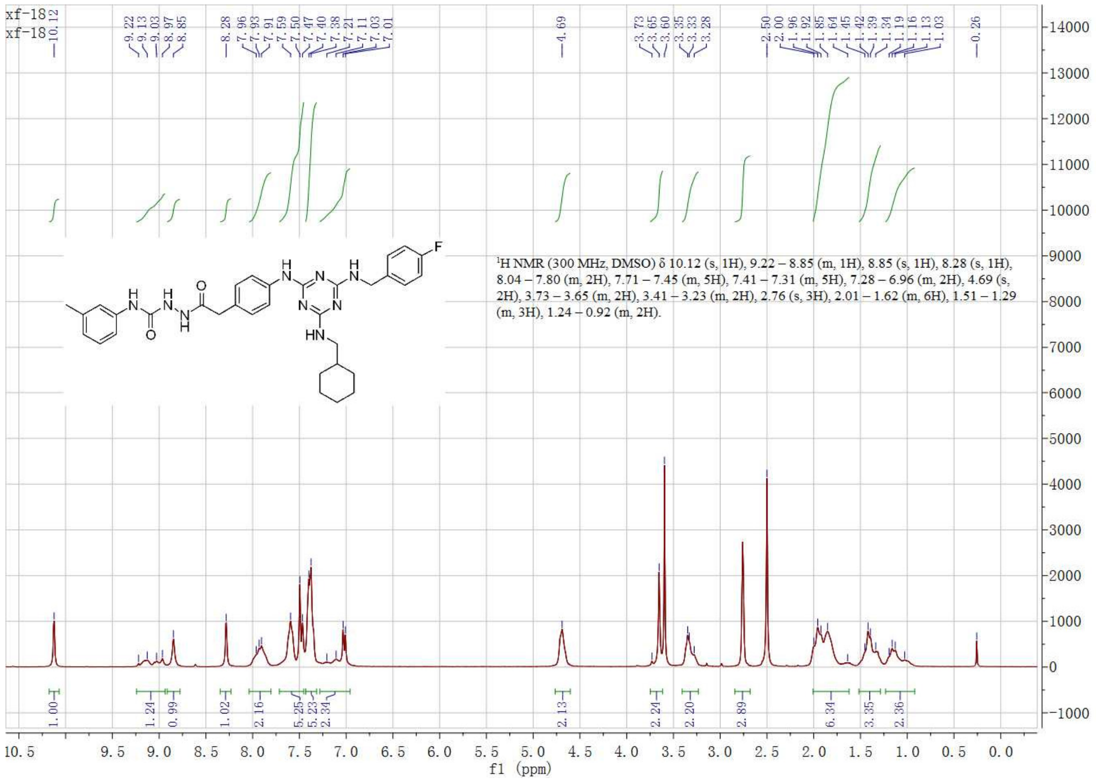

^{1}H-NMR of 9h

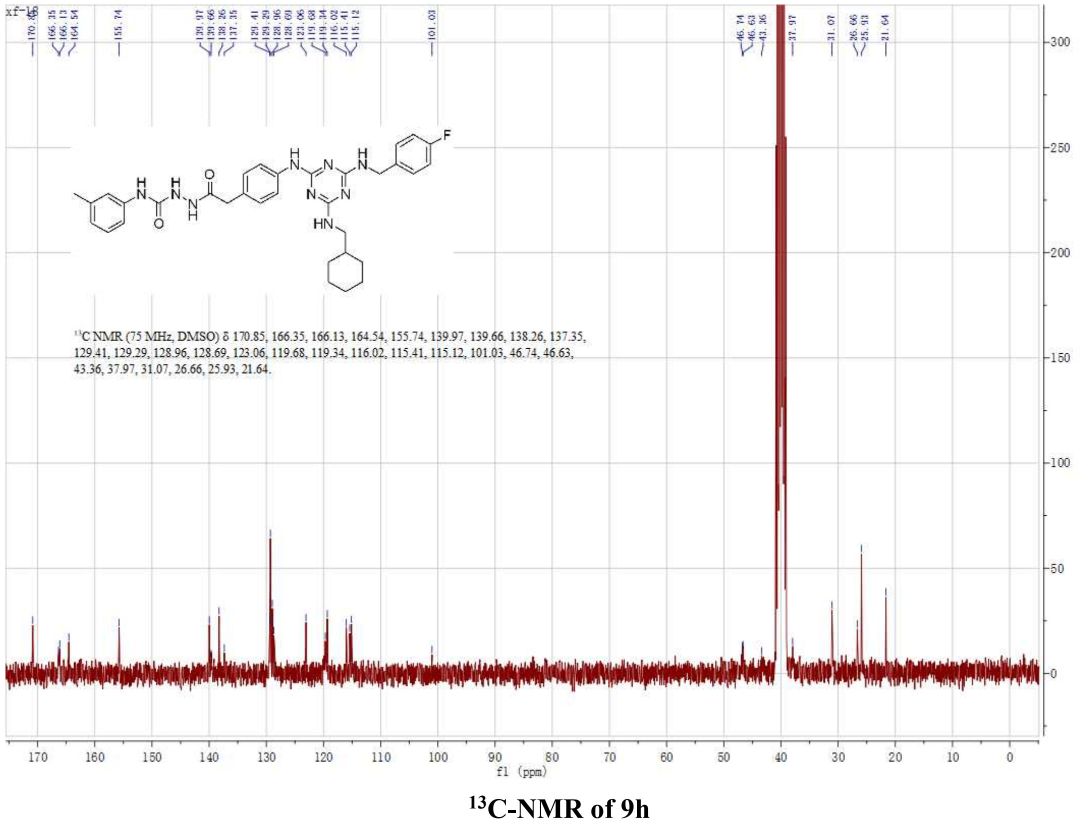

^{13}C-NMR of 9h

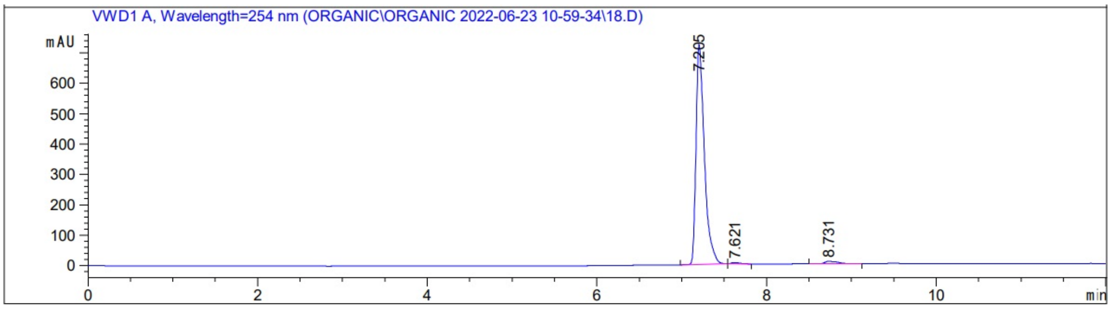

HPLC purity of 9h

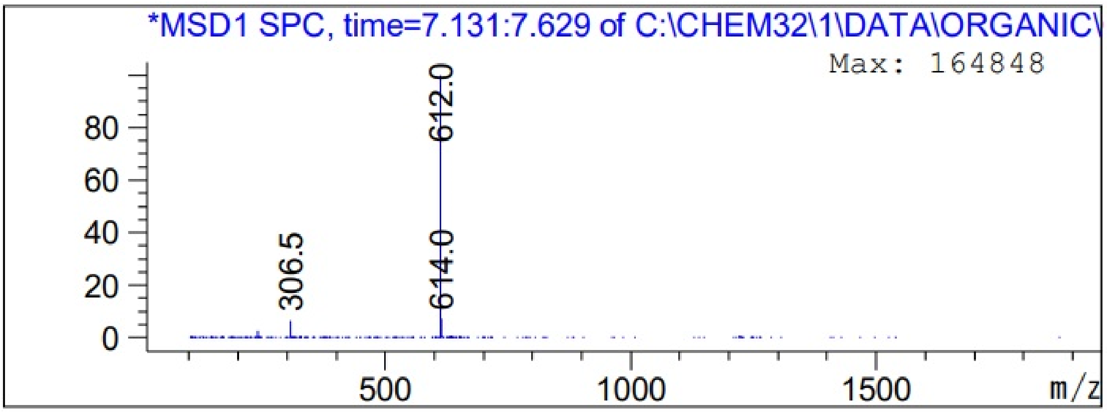

ESI-MS of 9h

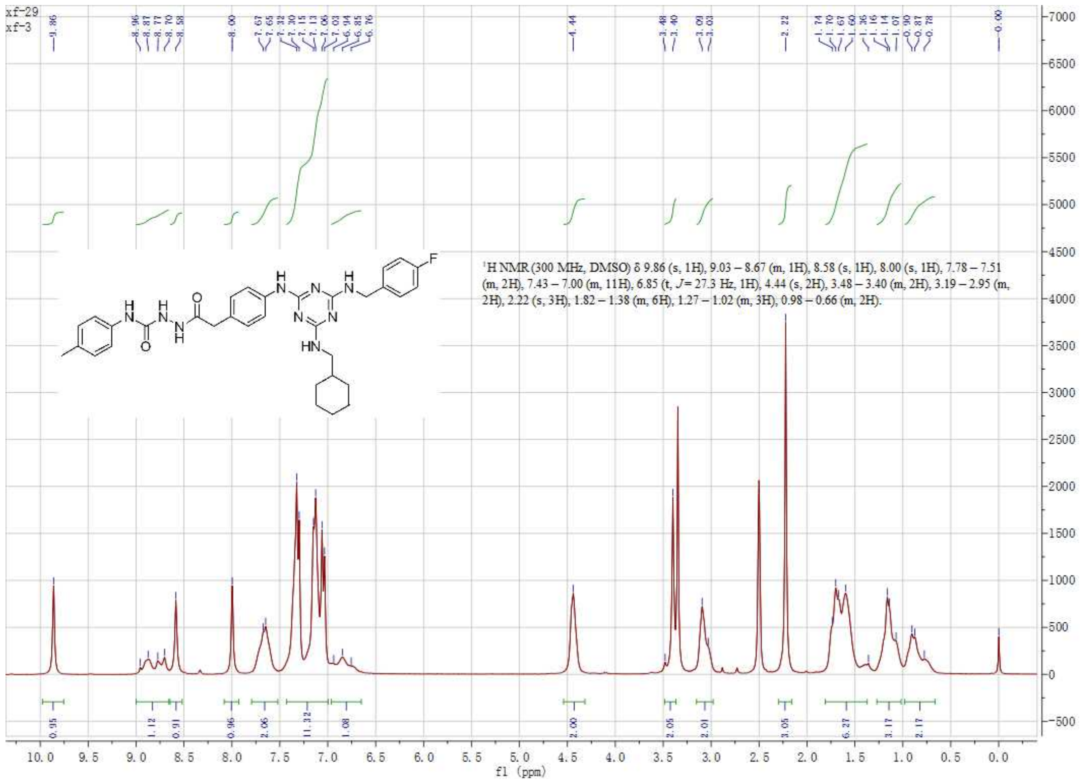

^{1}H-NMR of 9i

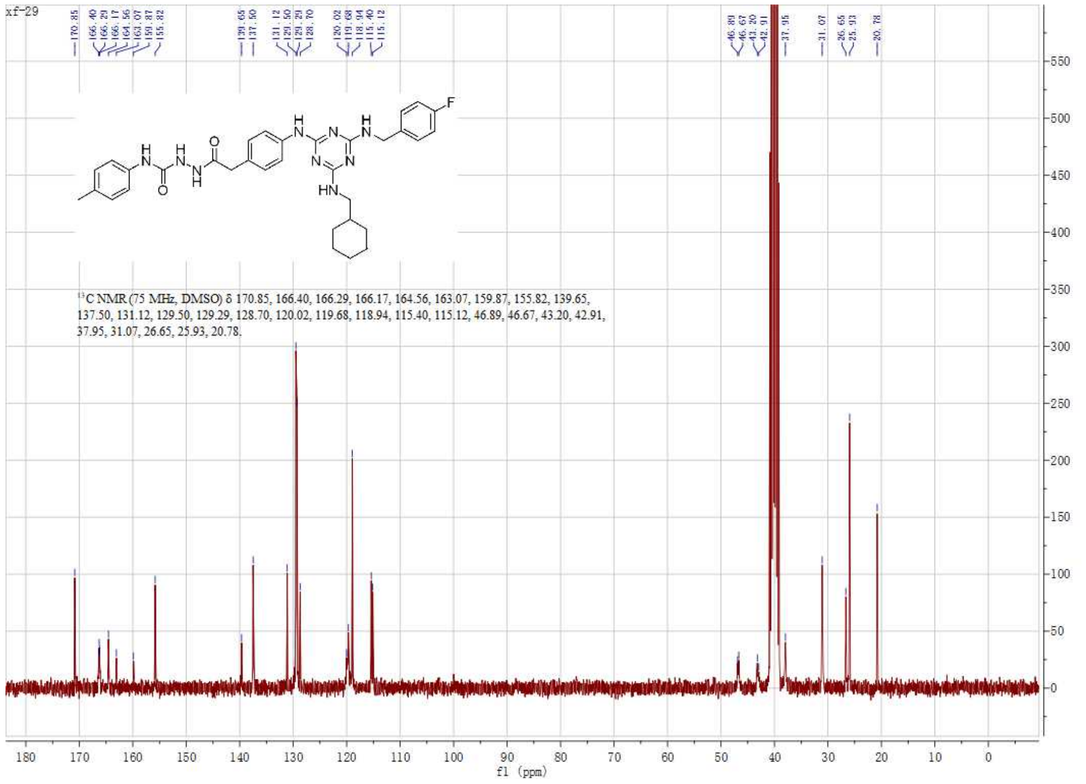

^{13}C-NMR of 9i

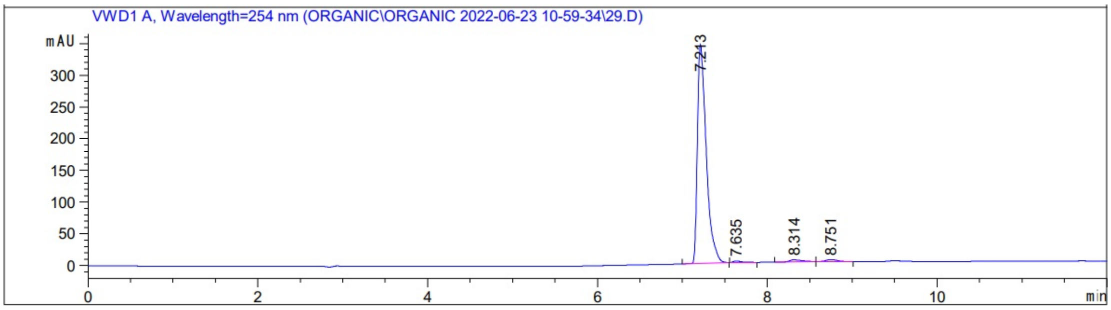

HPLC purity of 9i

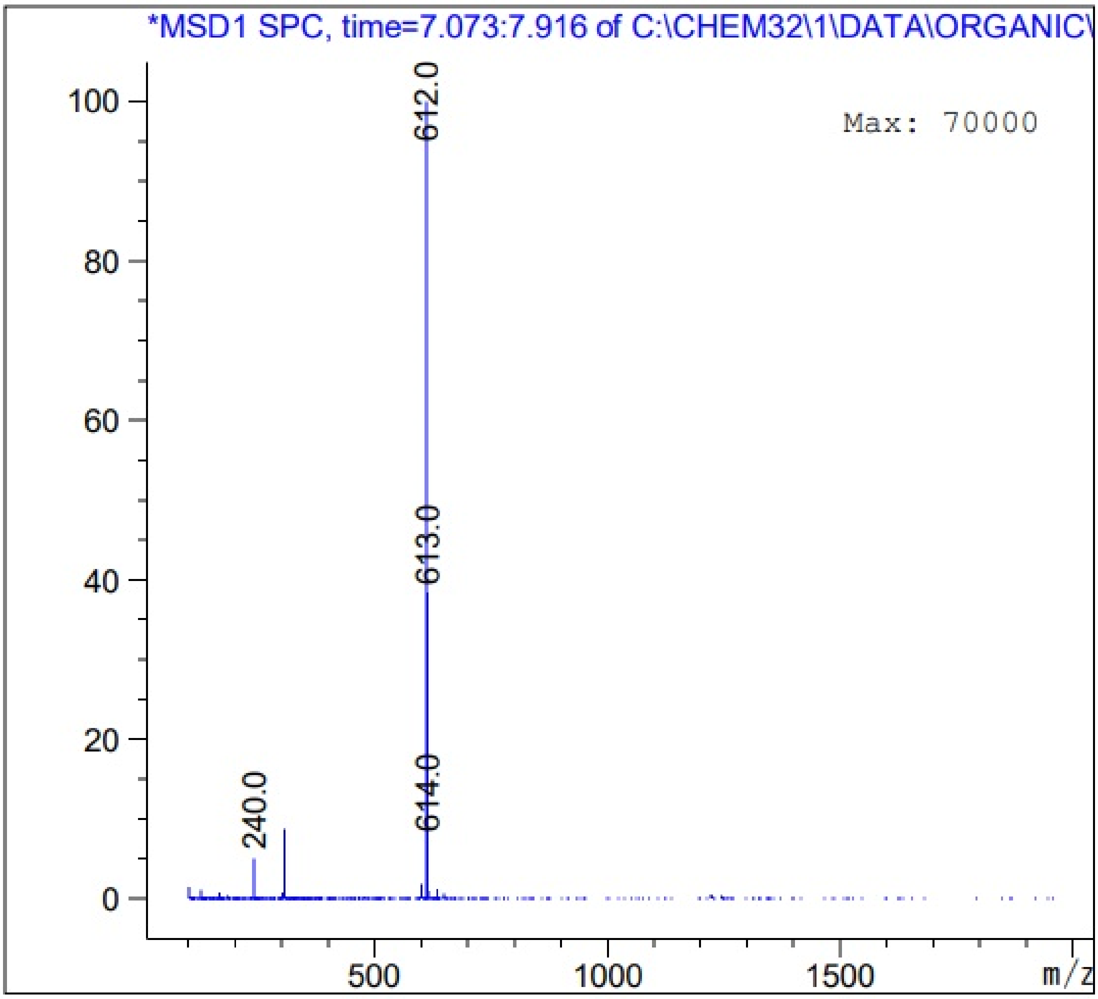

ESI-MS of 9i

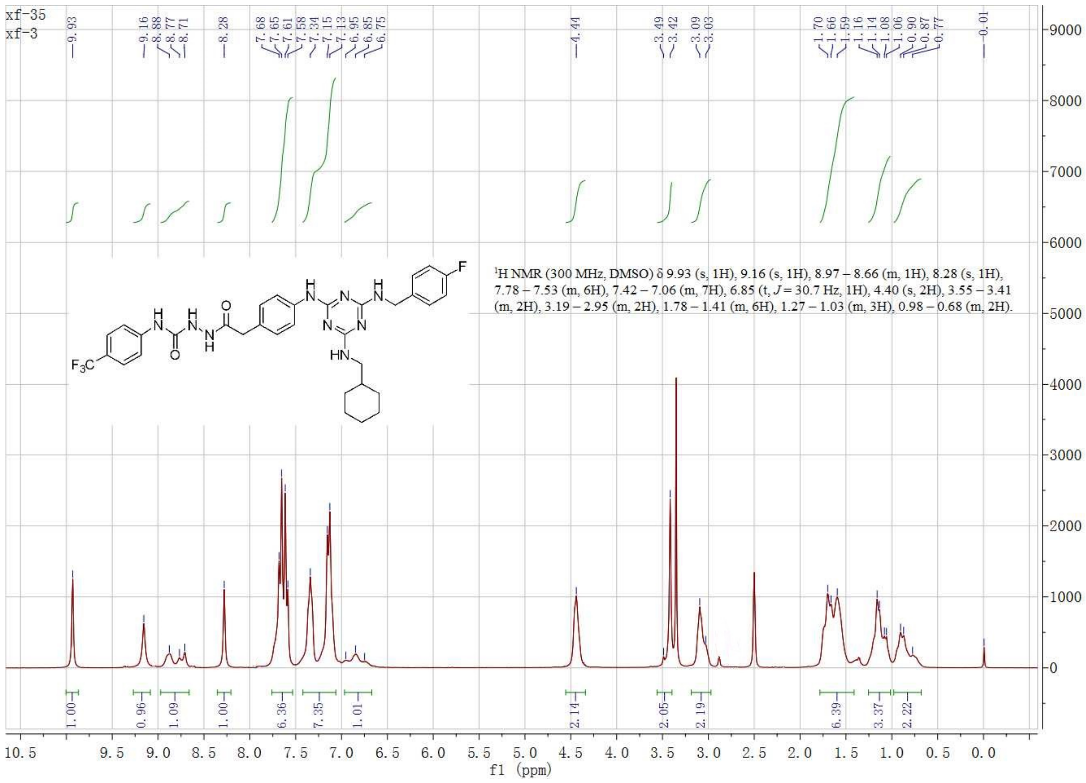

^{1}H-NMR of 9j

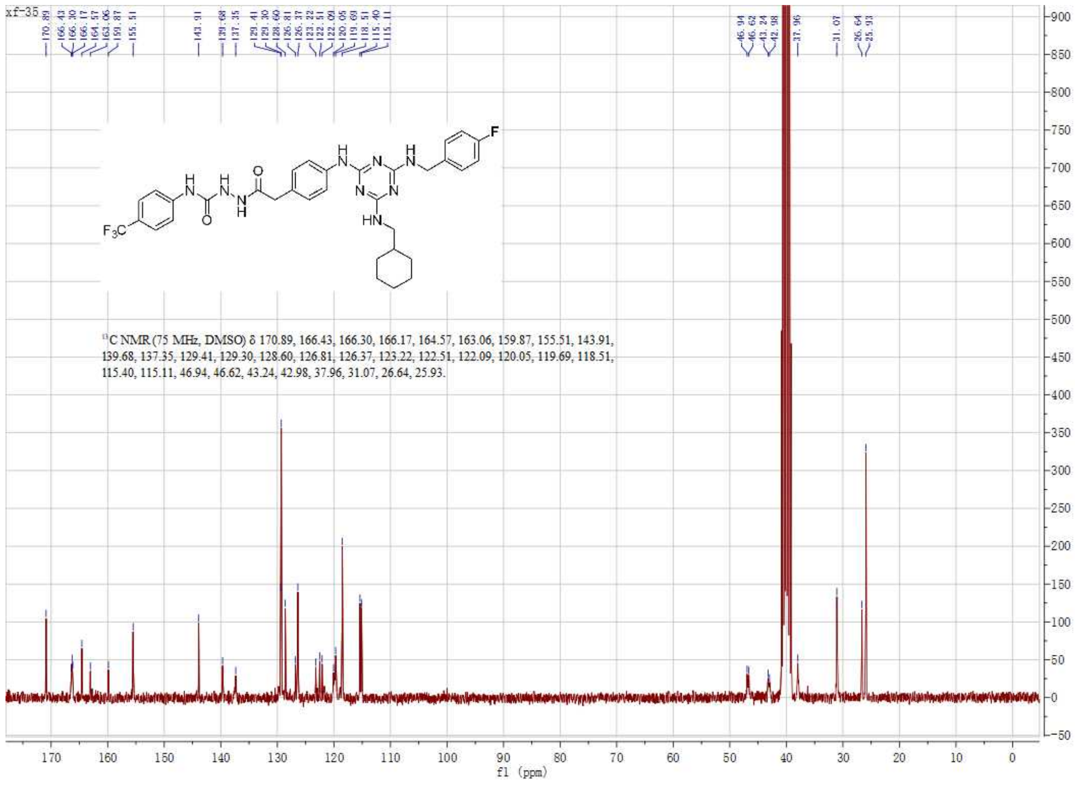

^{13}C-NMR of 9j

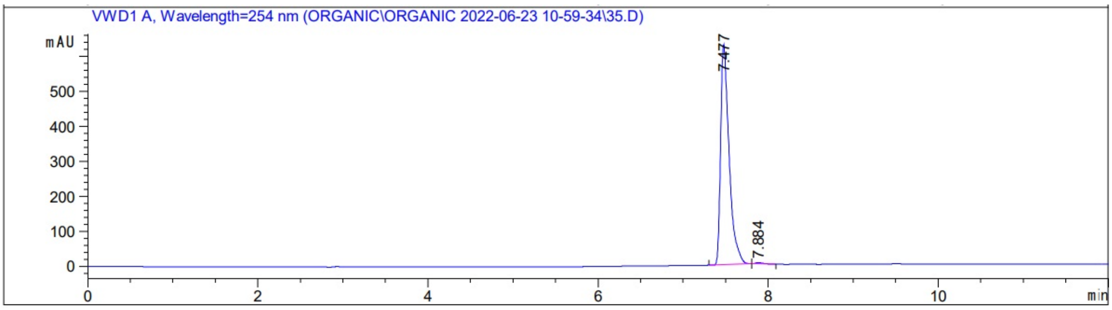

HPLC purity of 9j

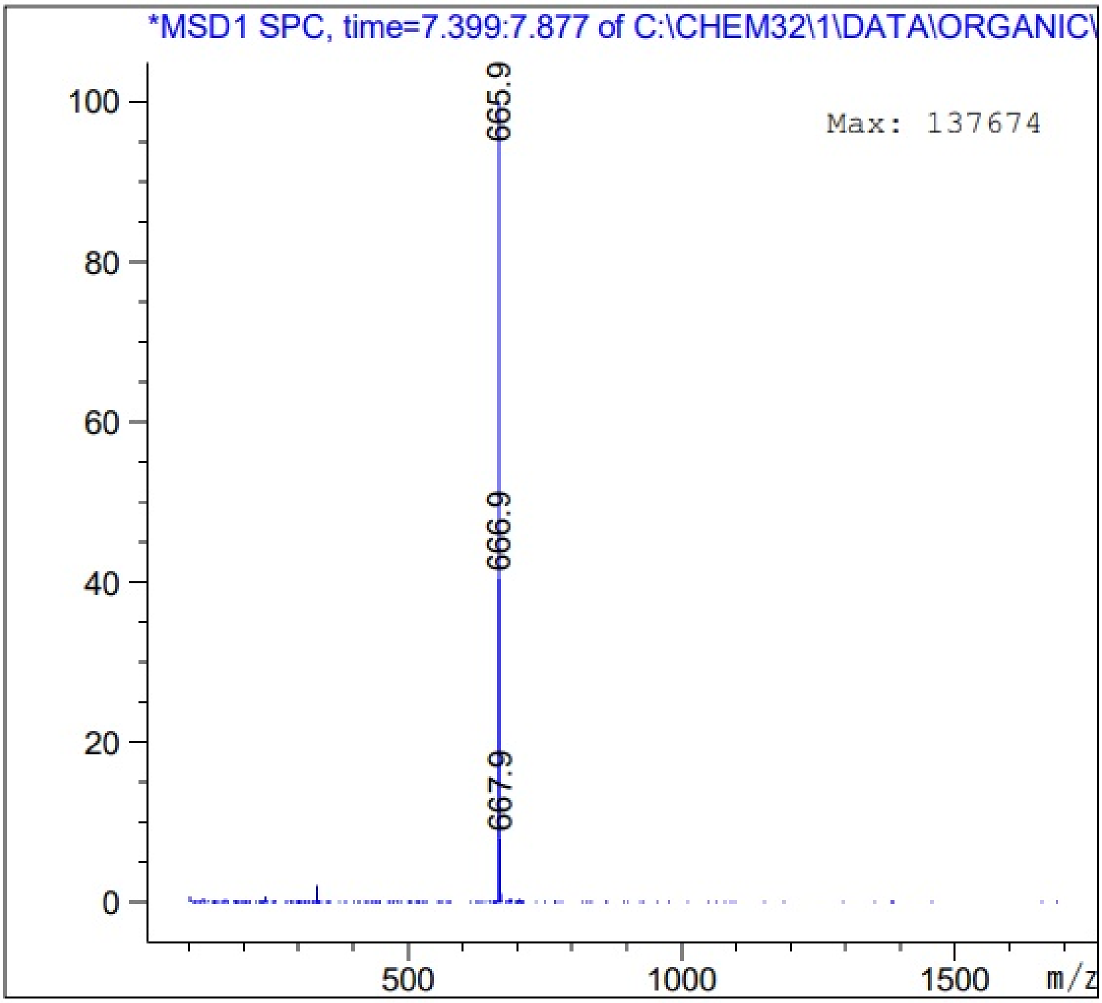

ESI-MS of 9j

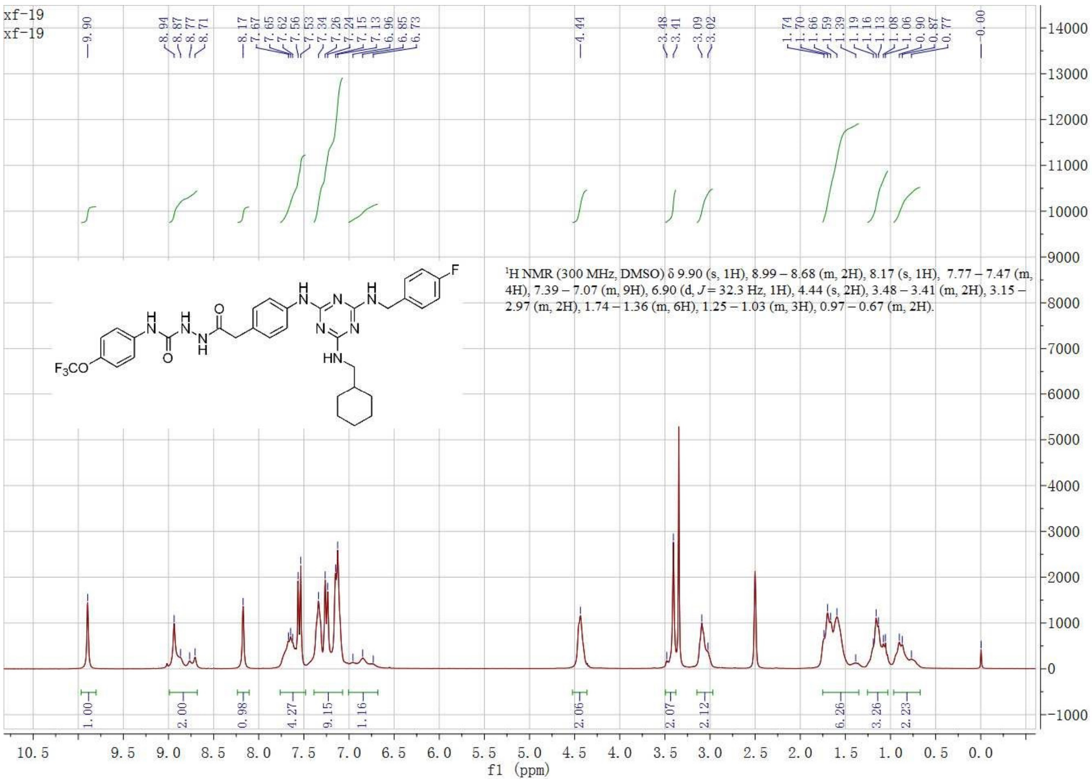

^{1}H-NMR of 9k

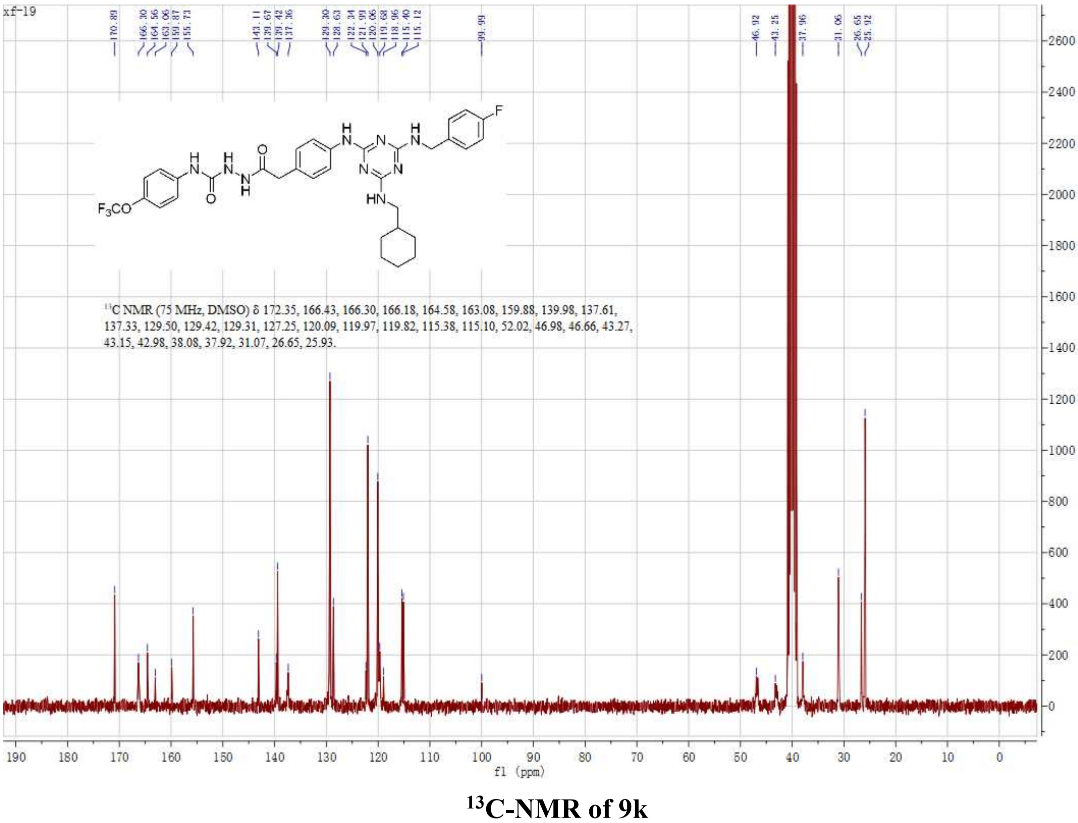

^{13}C-NMR of 9k

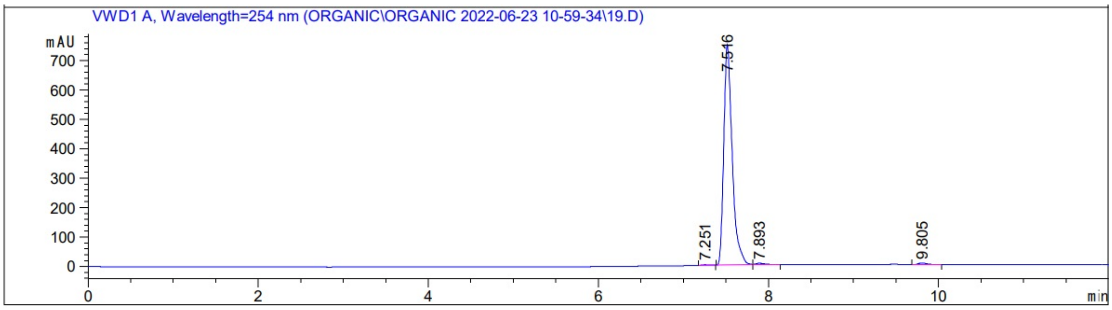

HPLC purity of 9k

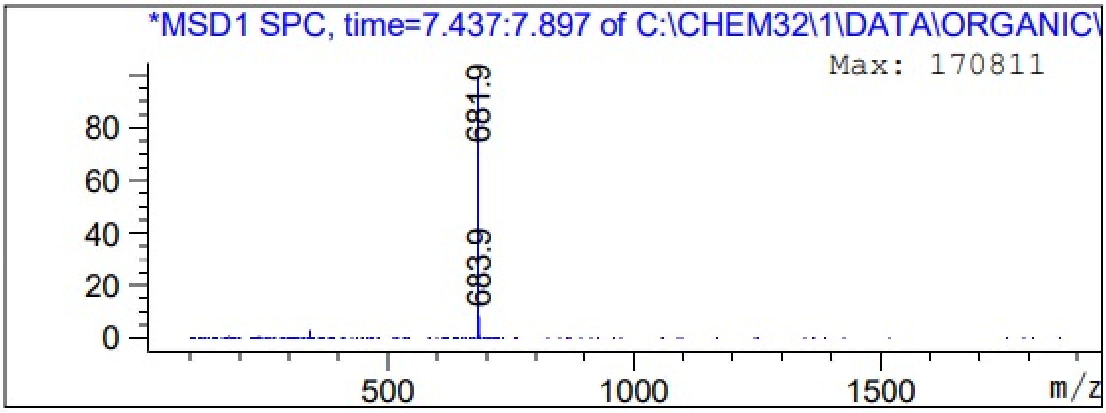

ESI-MS of 9k

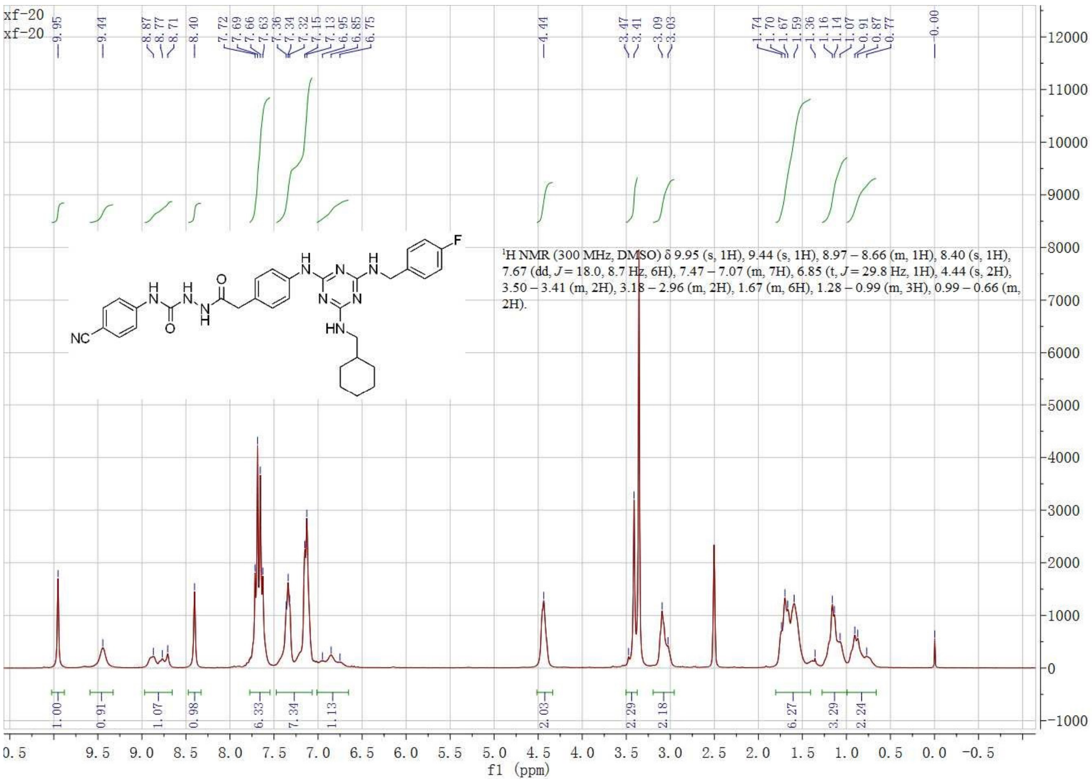

^{1}H-NMR of 9l

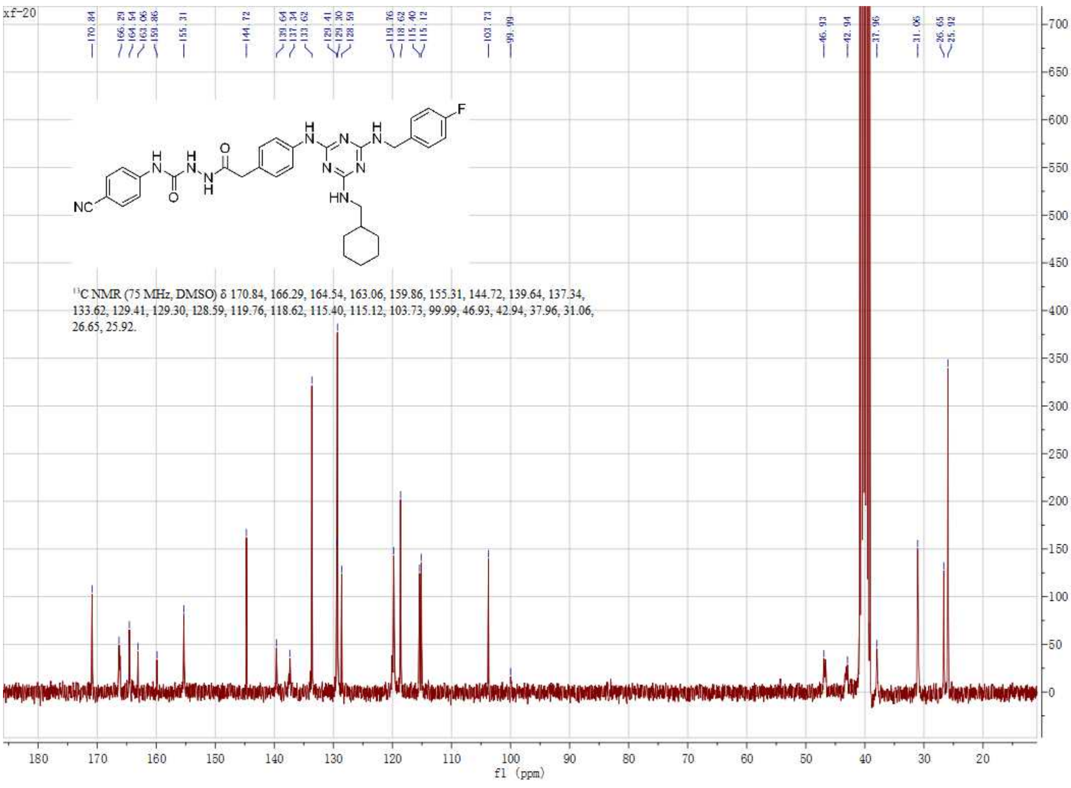

^{13}C-NMR of 9l

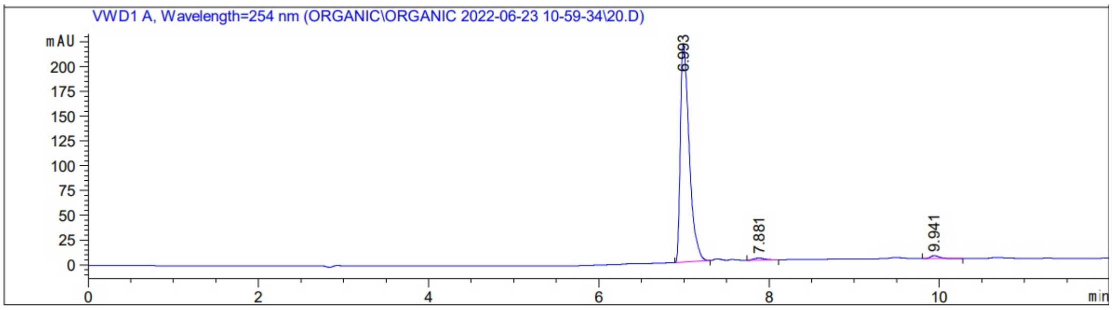

HPLC purity of 9l

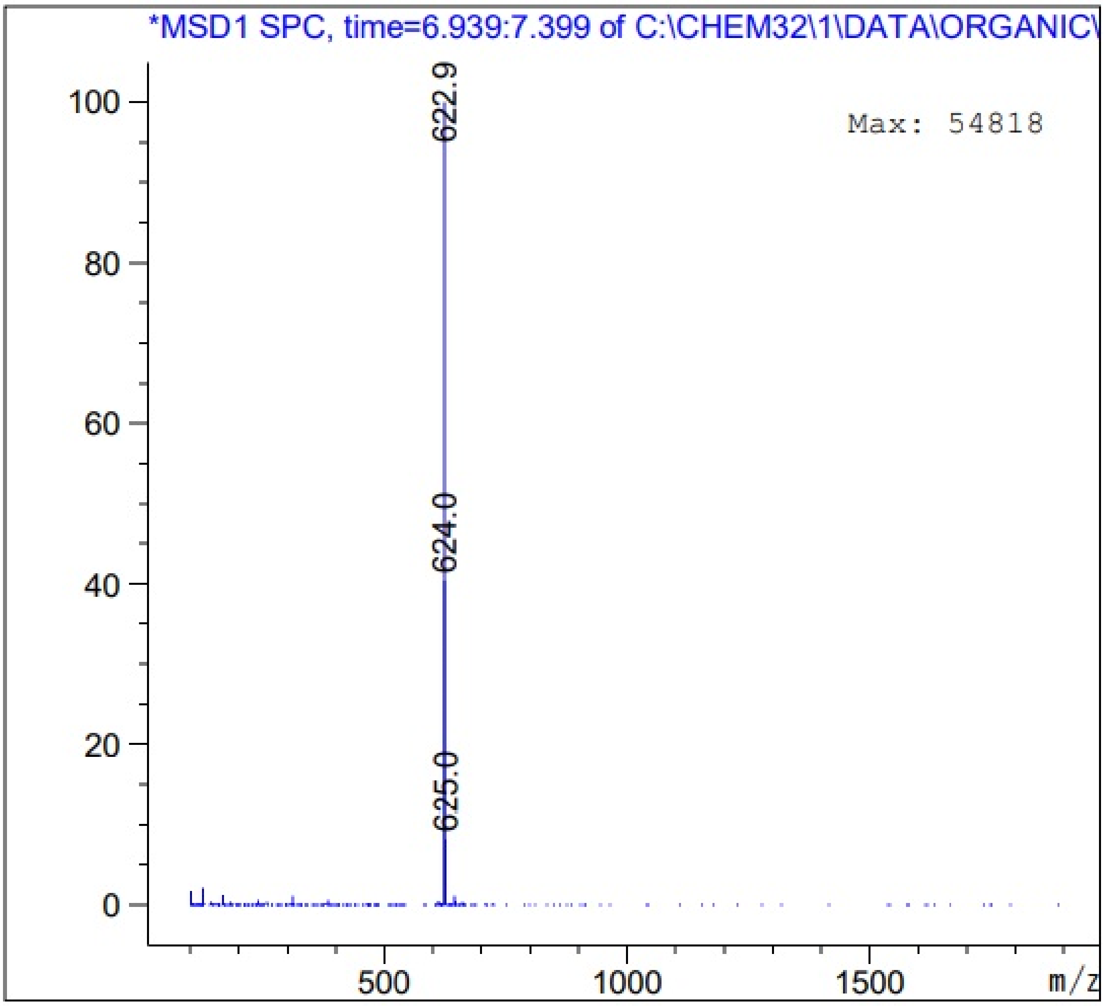

ESI-MS of 9l

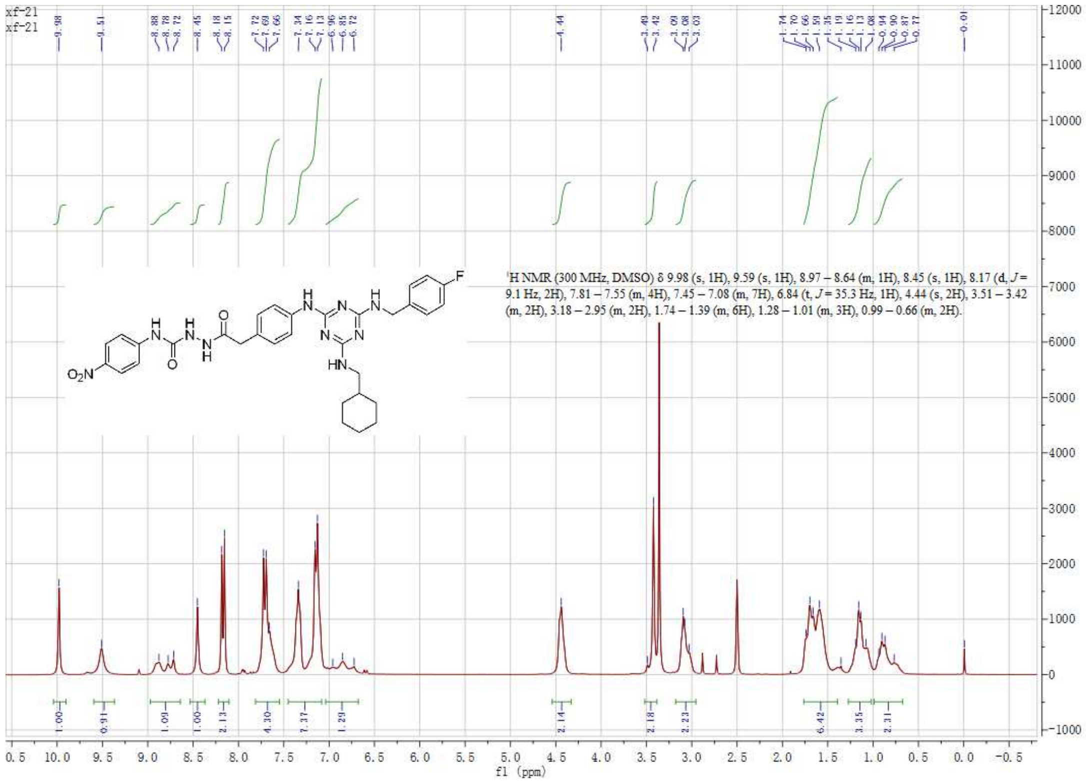

^{1}H-NMR of 9m

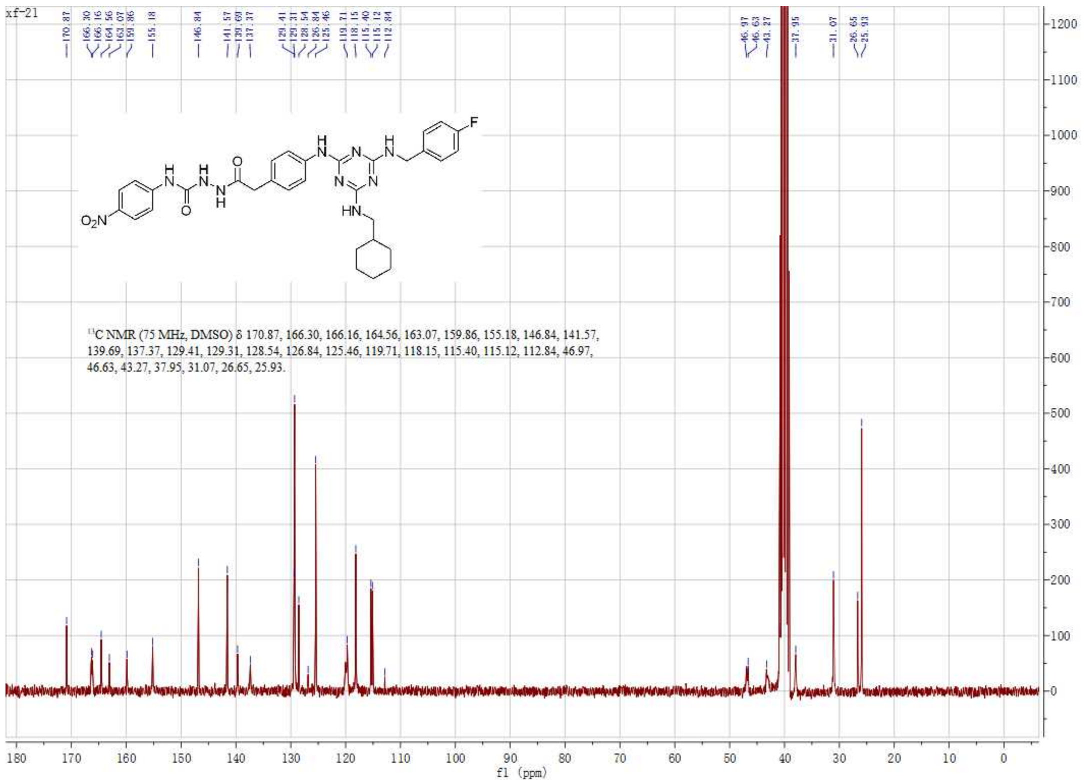

^{13}C-NMR of 9m

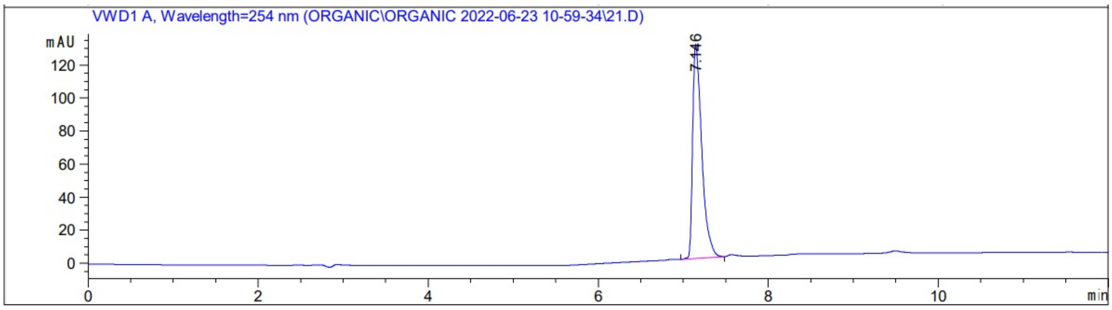

HPLC purity of 9m

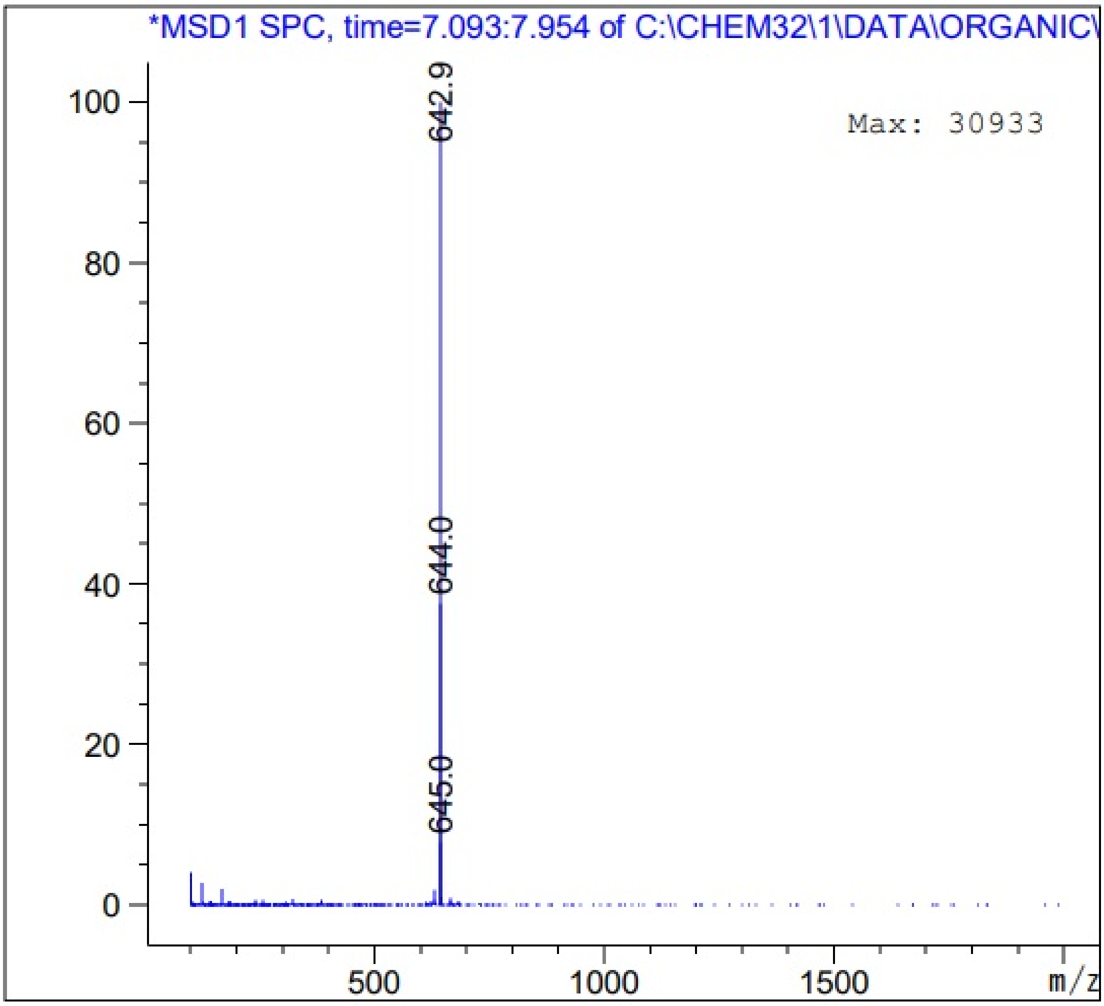

ESI-MS of 9m

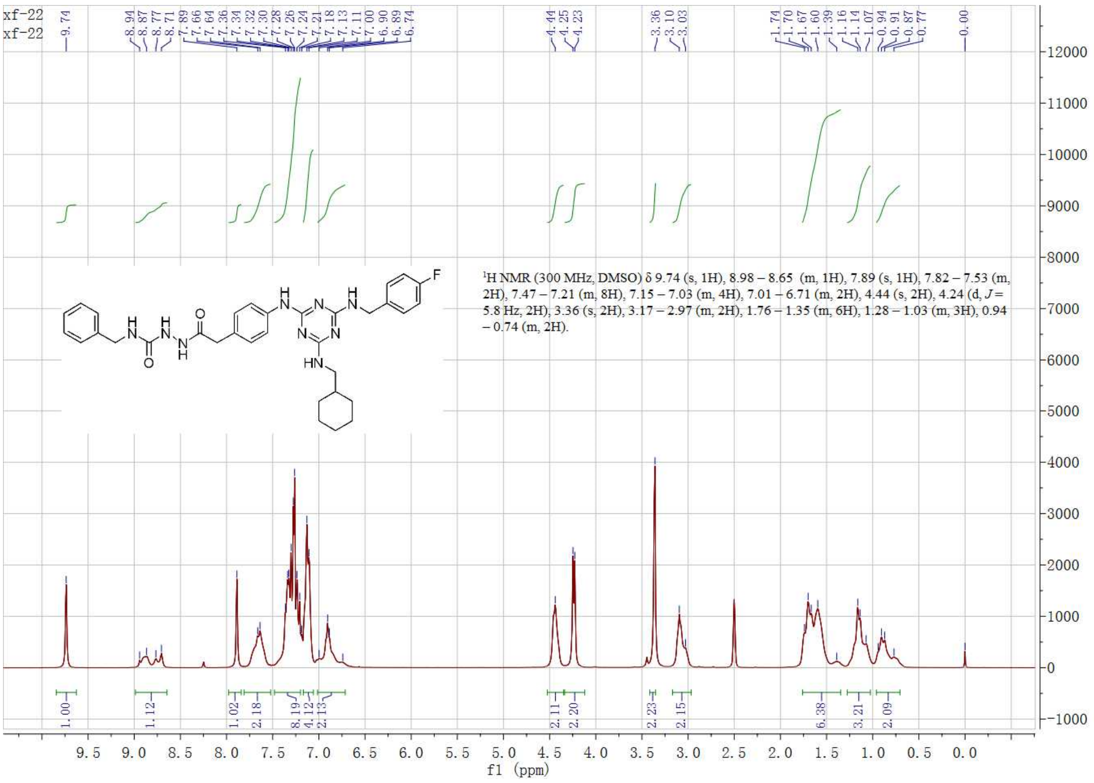

^{1}H-NMR of 9n

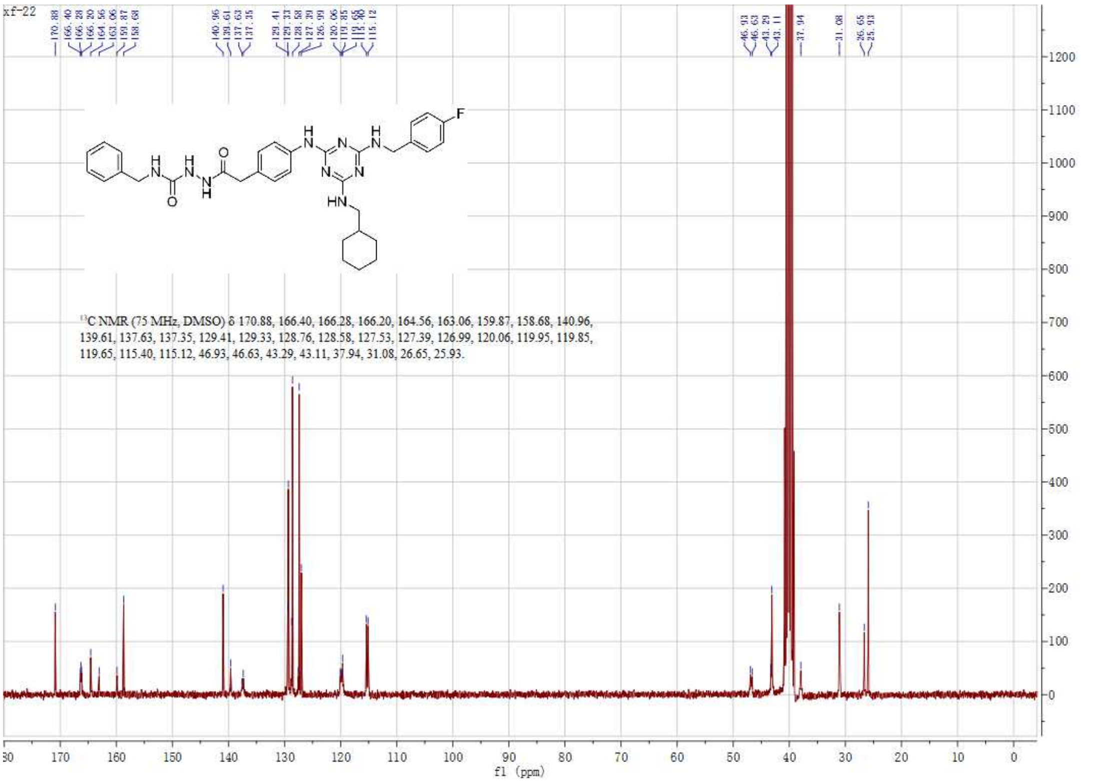

^{13}C-NMR of 9n

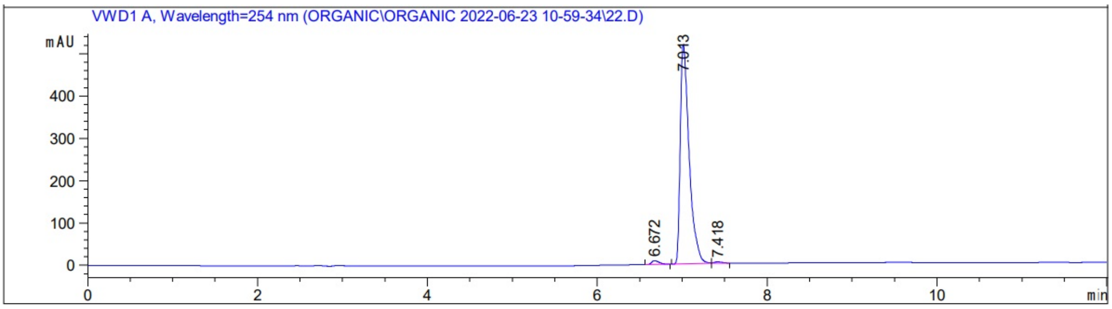

HPLC purity of 9n

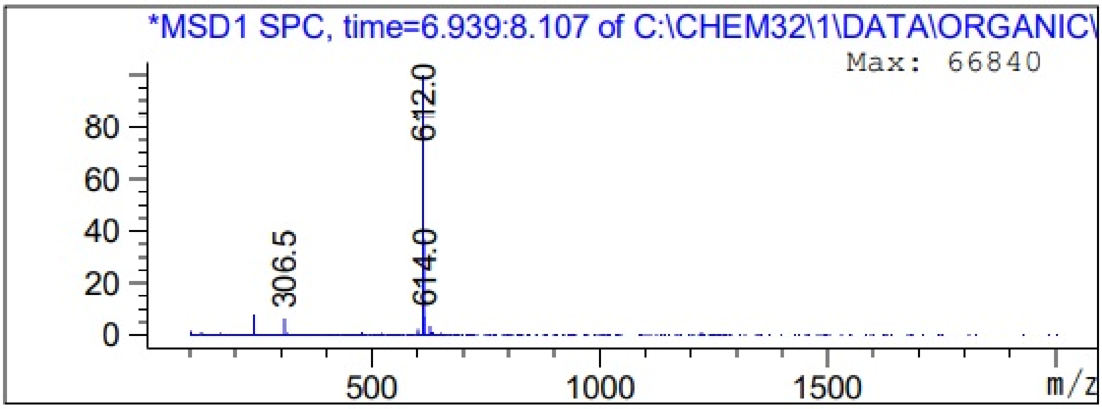

ESI-MS of 9n

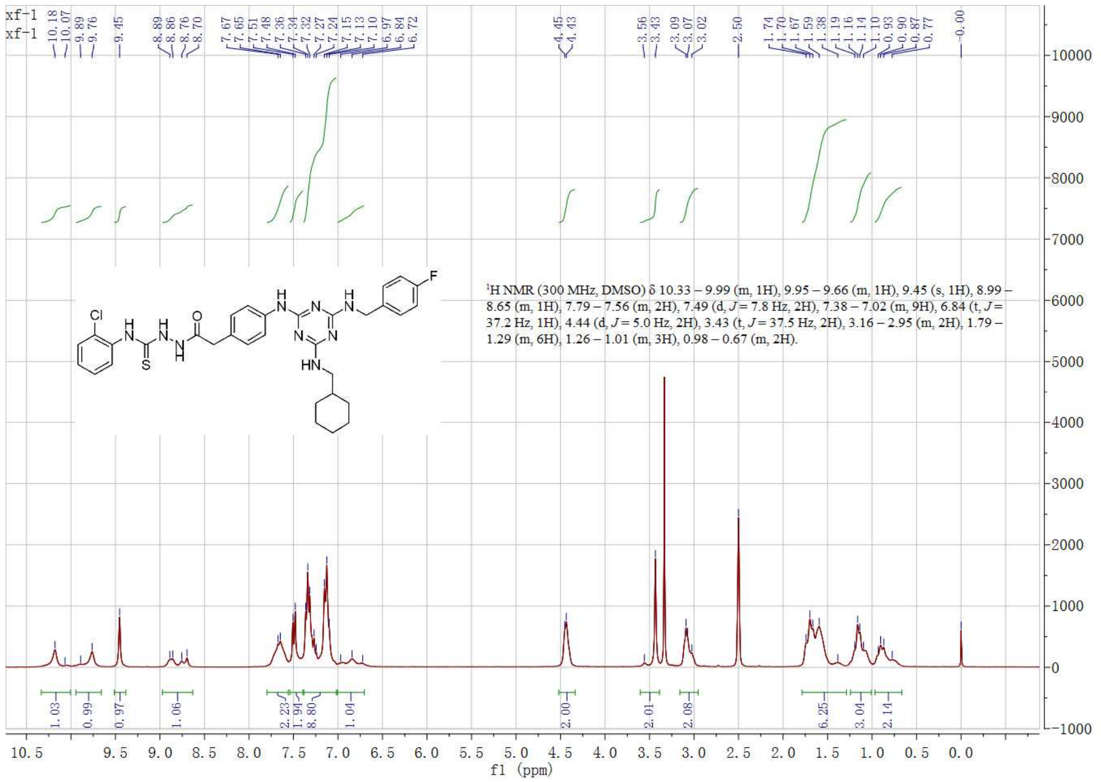

^{1}H-NMR of 10a

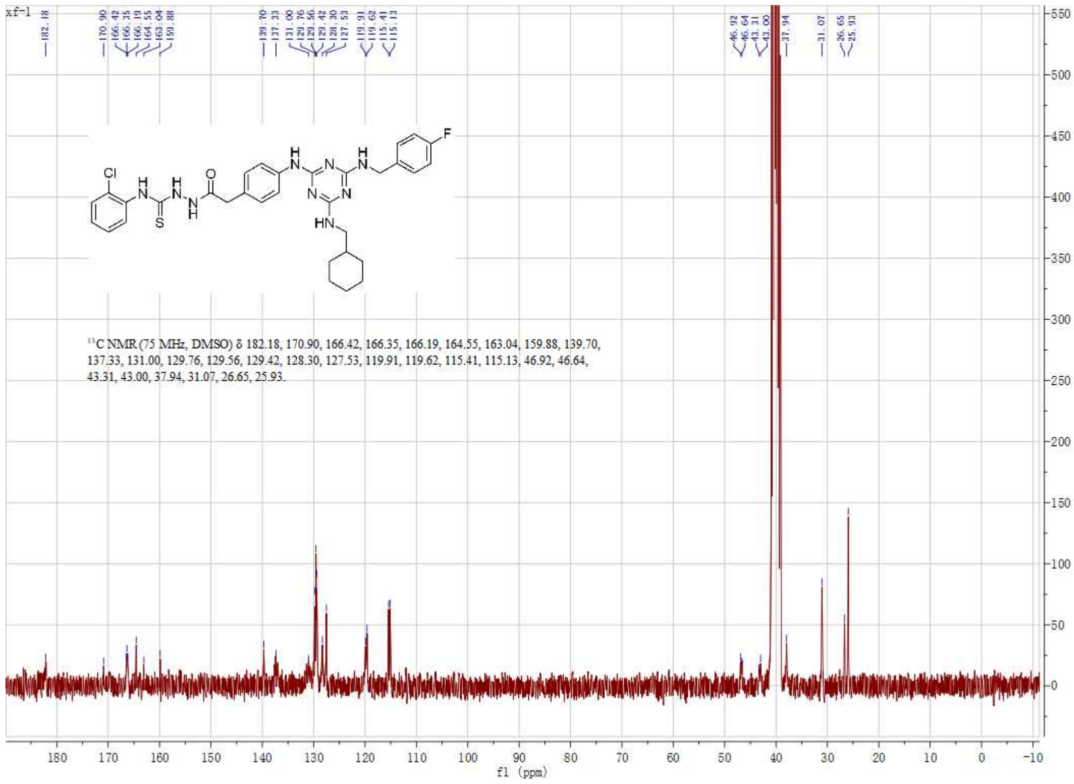

^{13}C-NMR of 10a

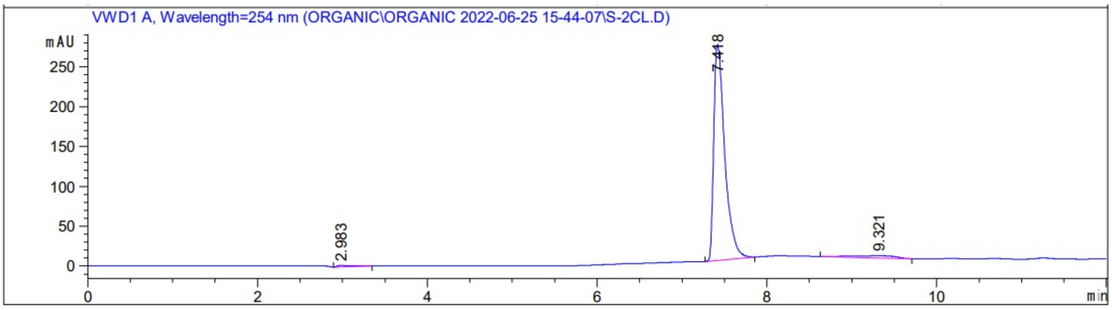

HPLC purity of 10a

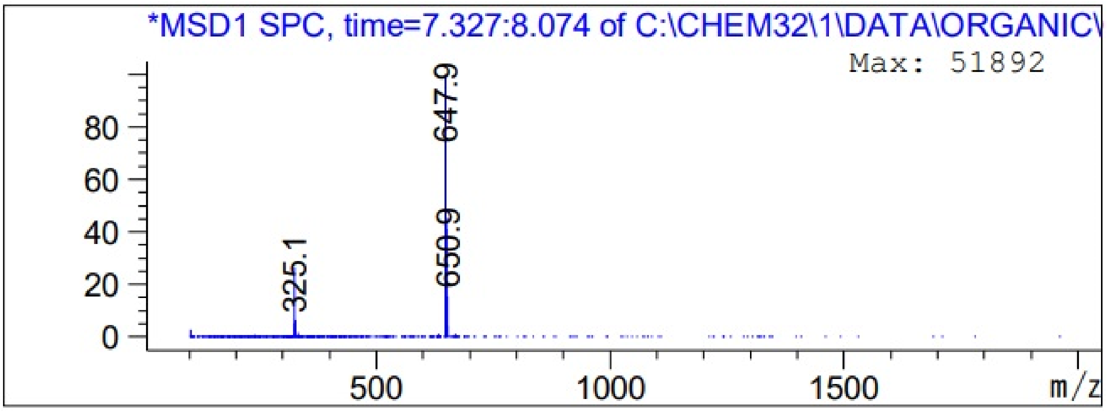

ESI-MS of 10a

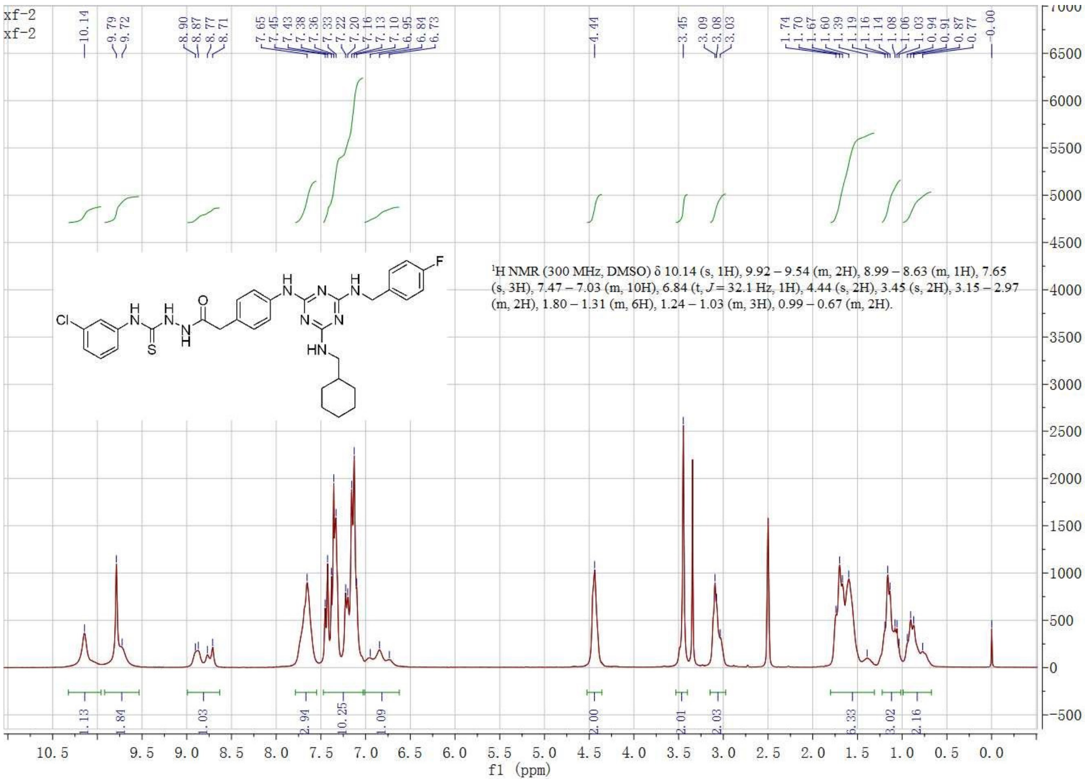

^{1}H-NMR of 10b

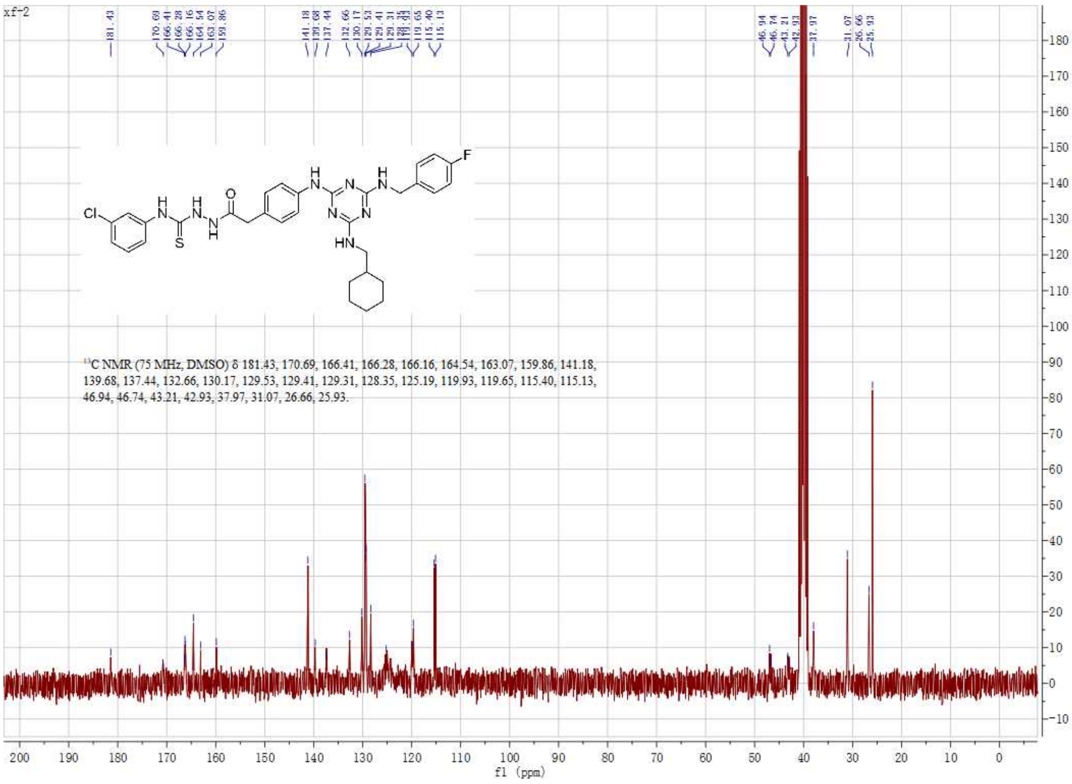

^{13}C-NMR of 10b

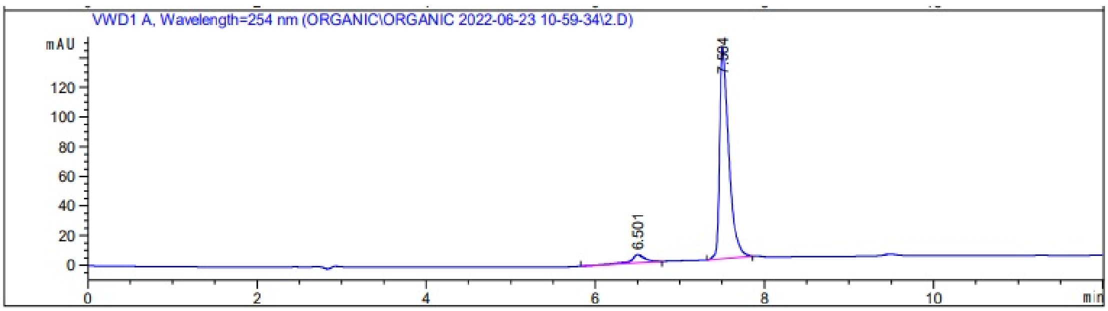

HPLC purity of 10b

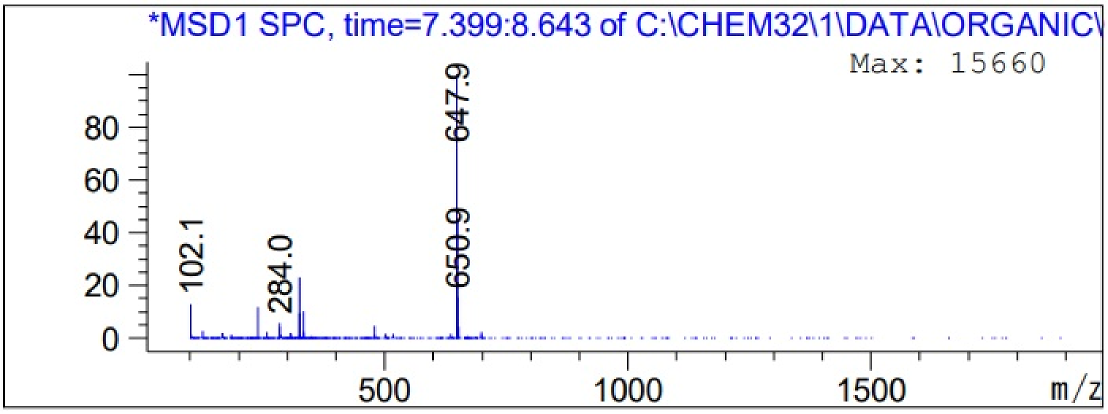

ESI-MS of 10b

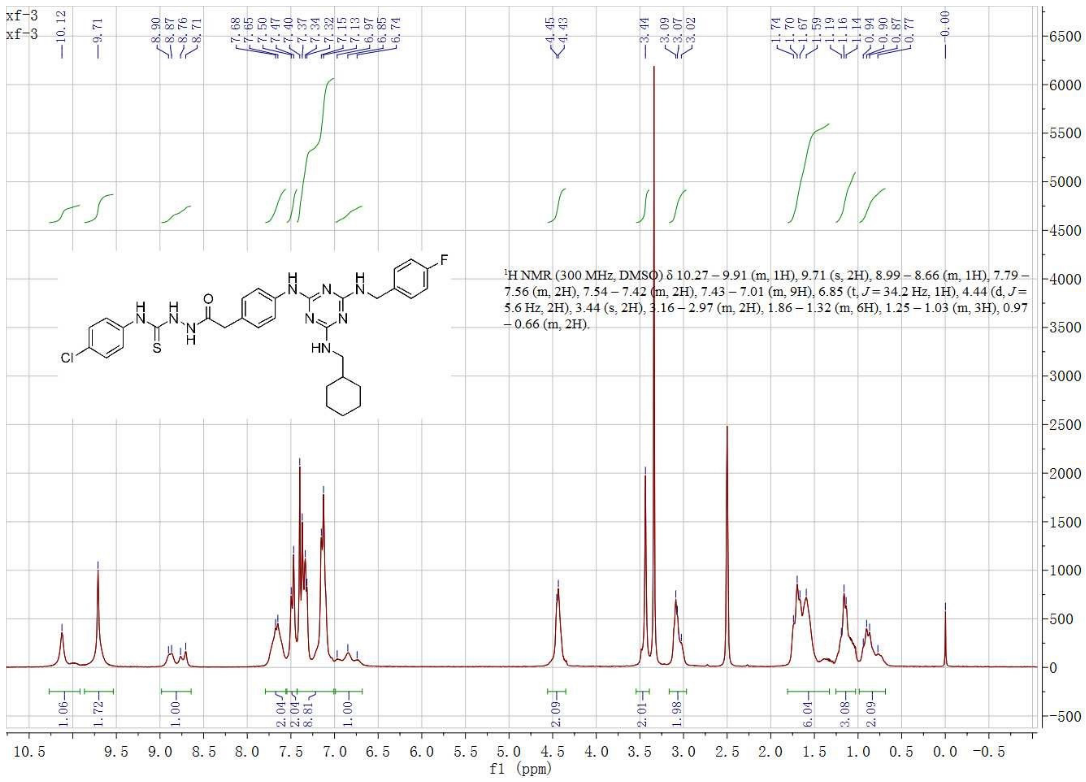

¹H-NMR of 10c

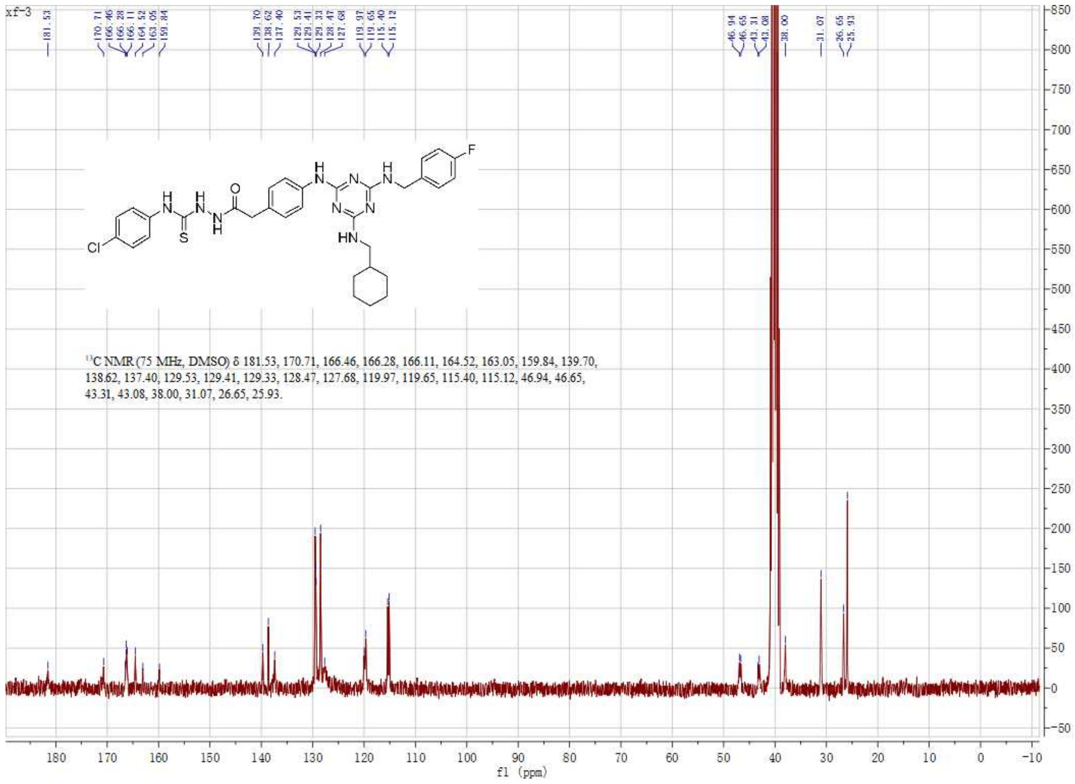

¹³C-NMR of 10c

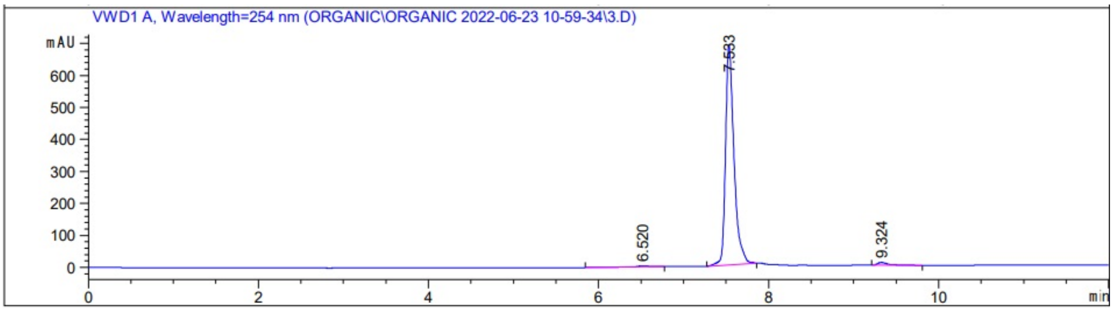

HPLC purity of 10c

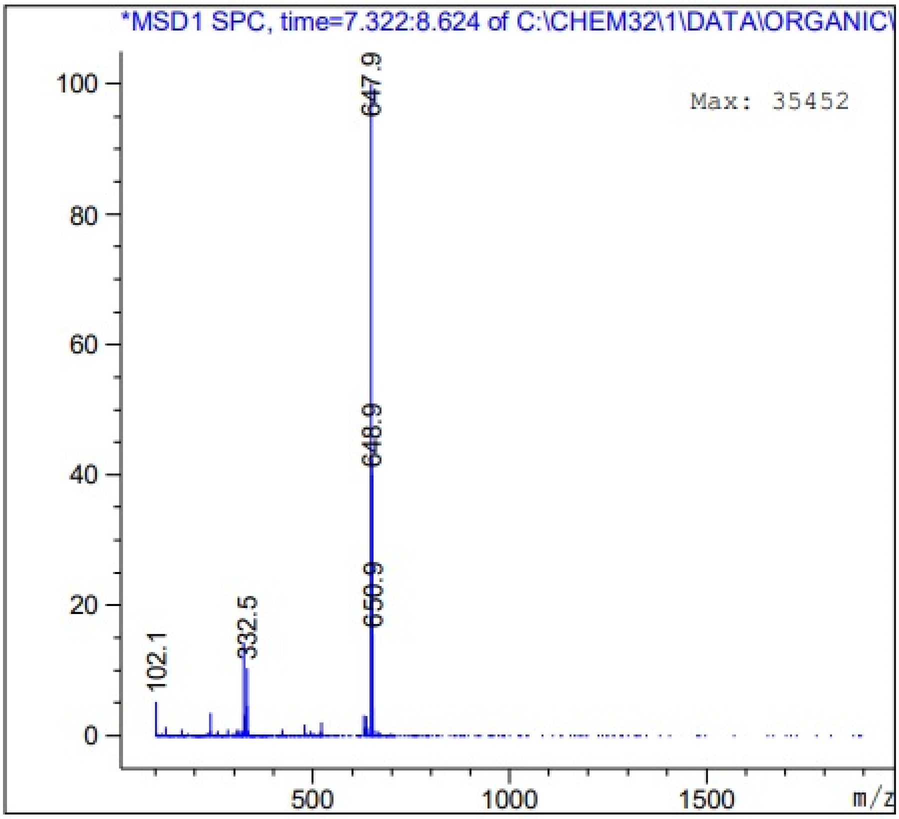

ESI-MS of 10c

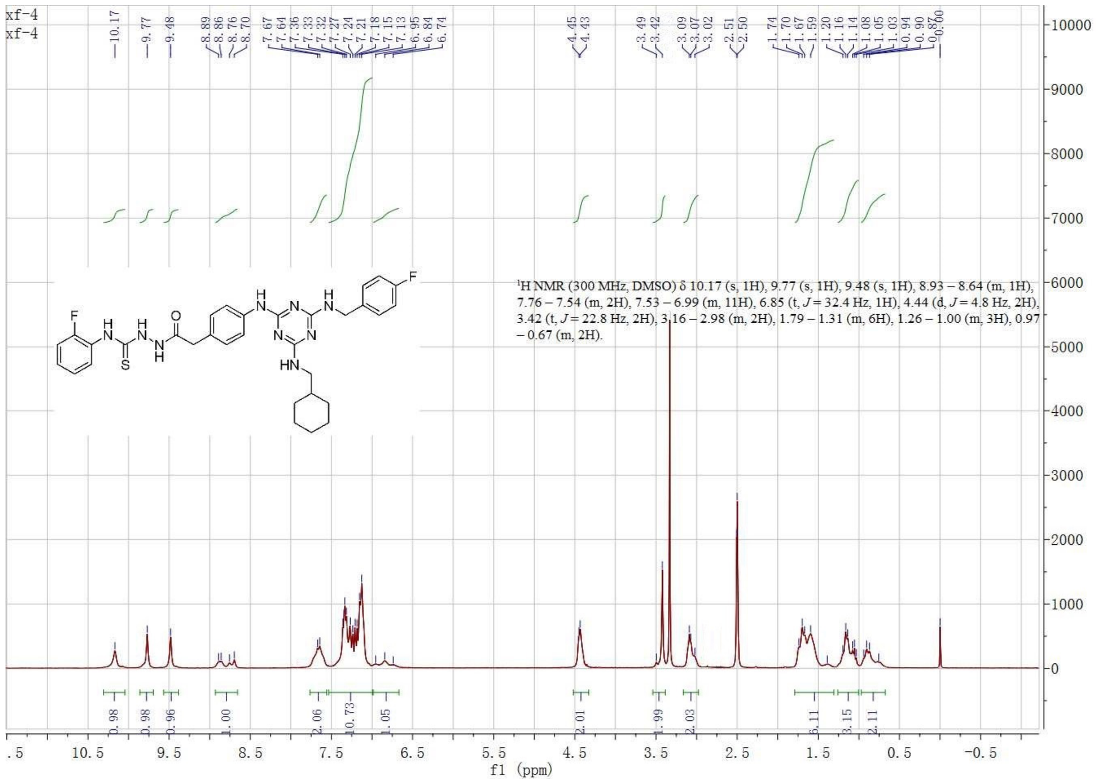

^{1}H-NMR of 10d

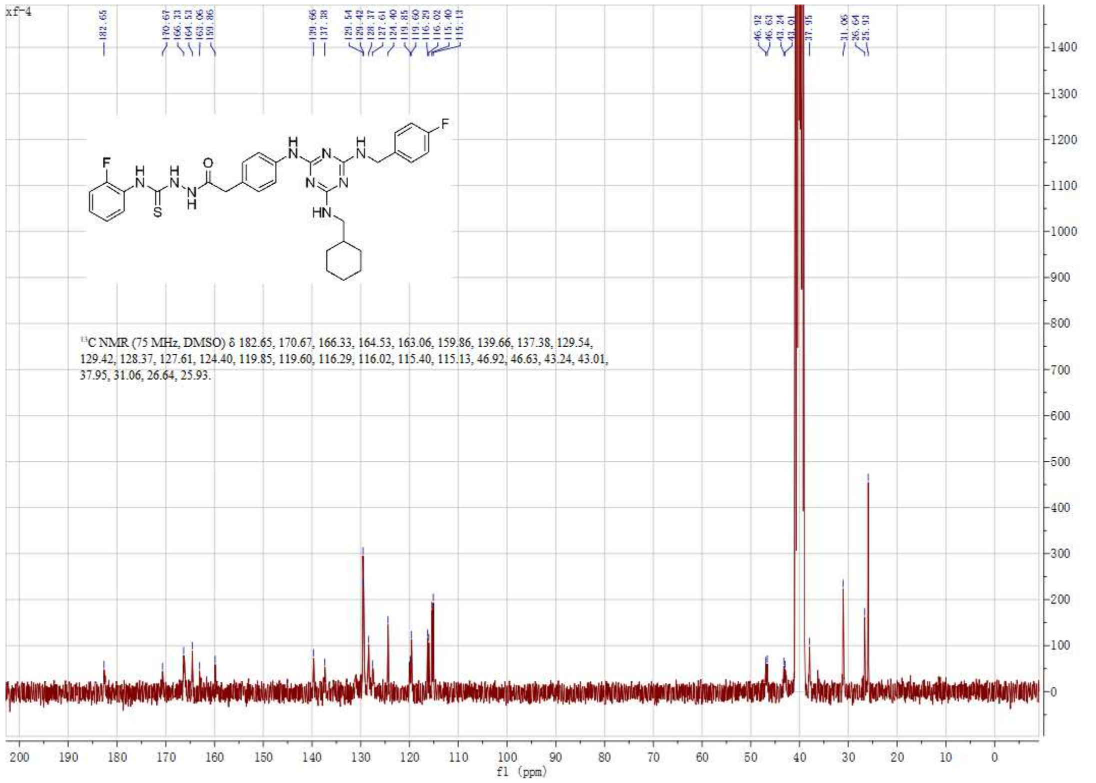

^{13}C-NMR of 10d

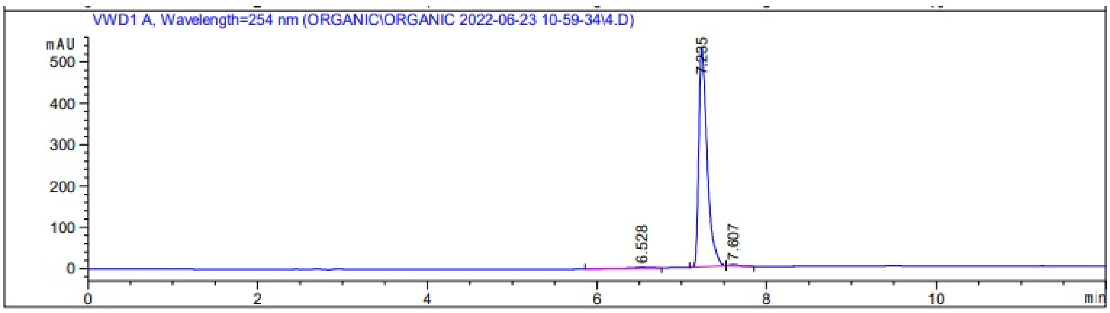

HPLC purity of 10d

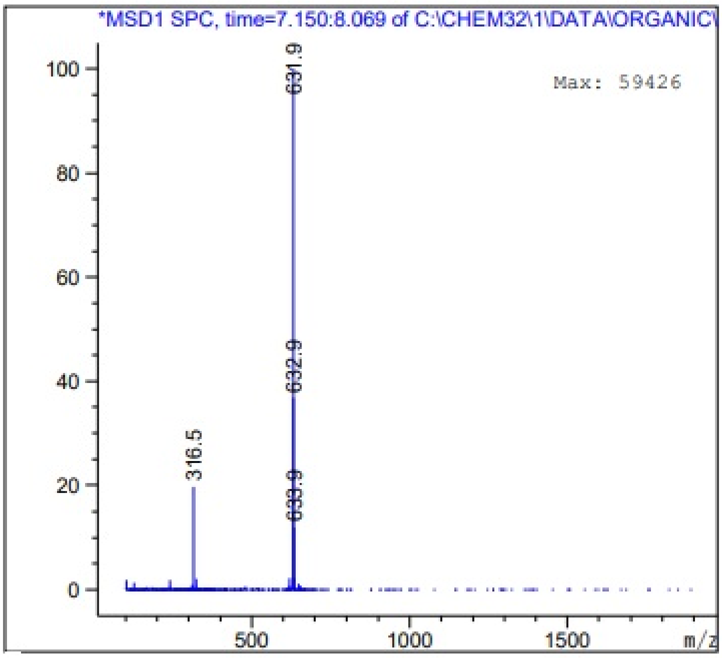

ESI-MS of 10d

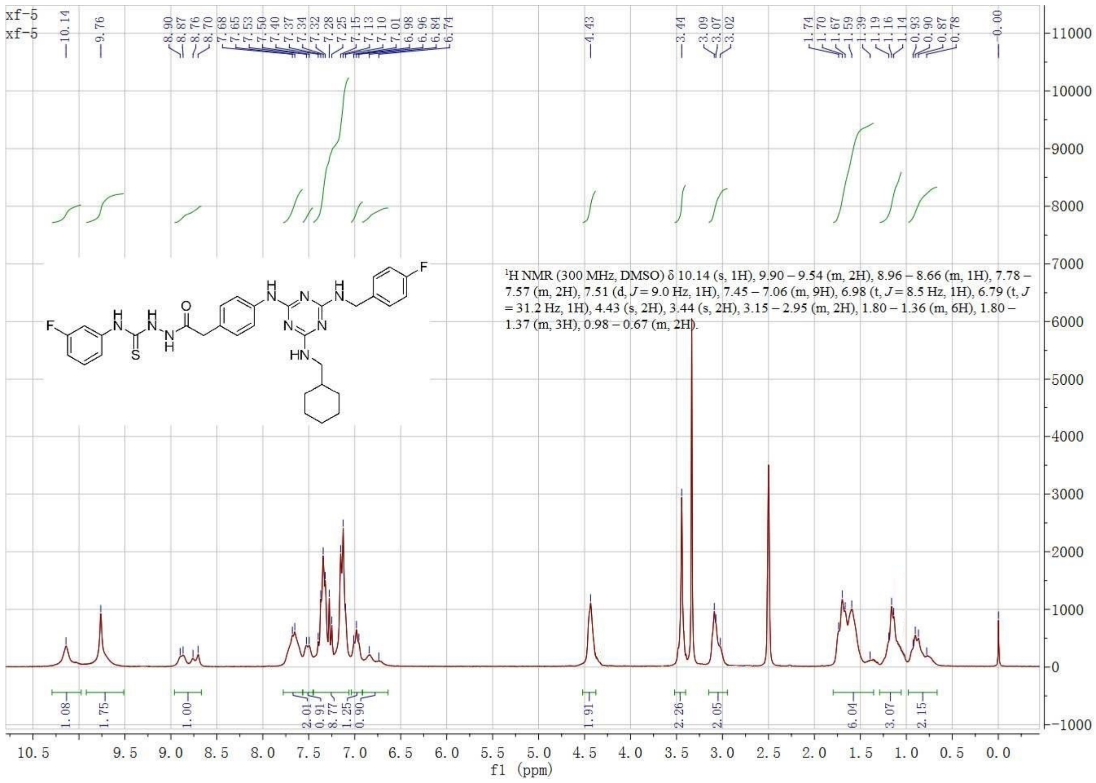

^{1}H-NMR of 10e

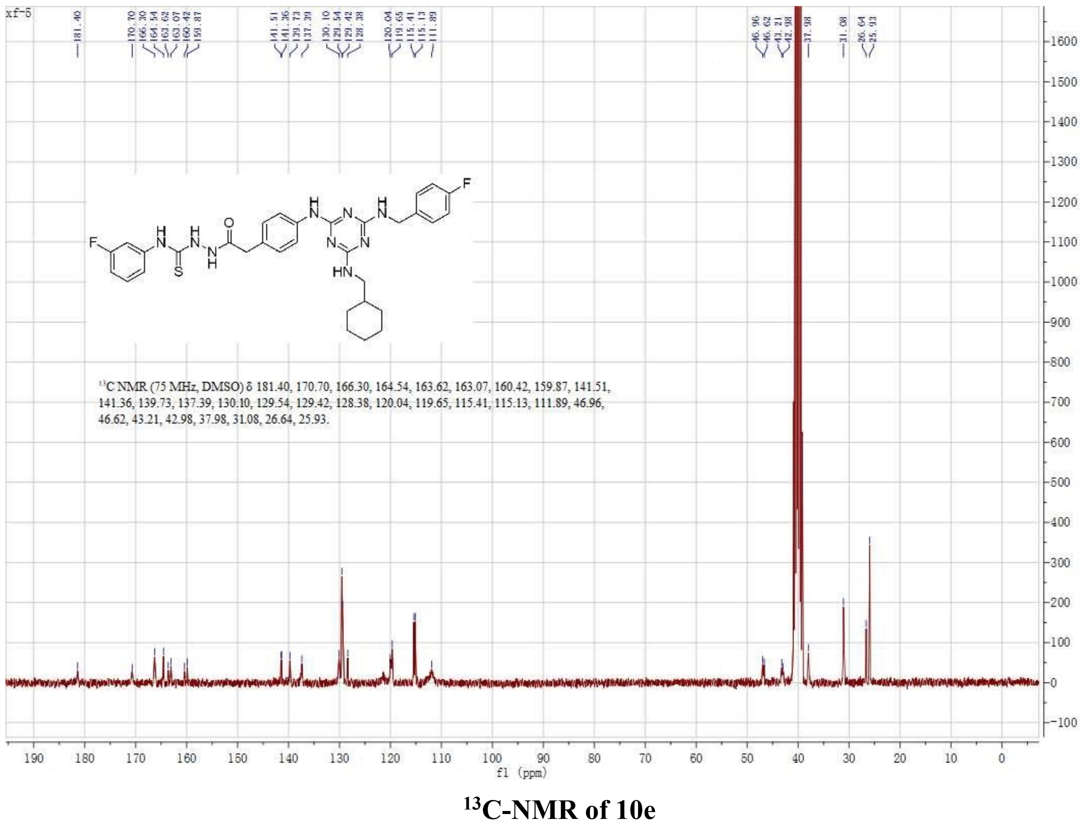

^{13}C-NMR of 10e

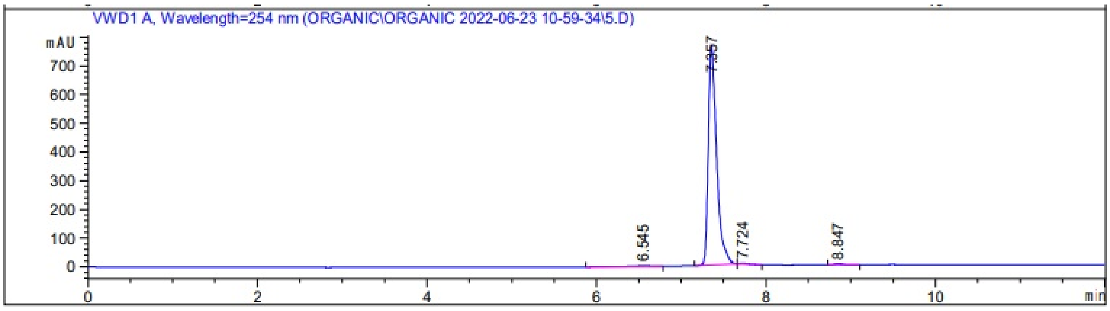

HPLC purity of 10e

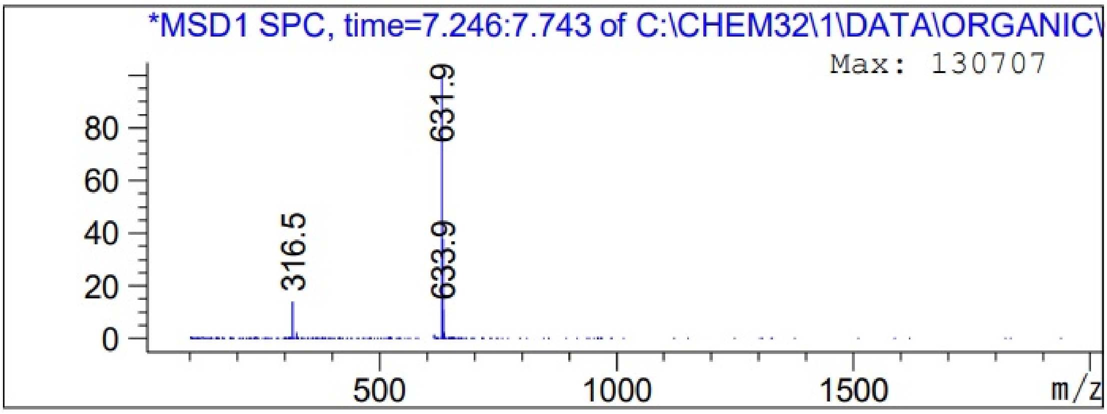

ESI-MS of 10e

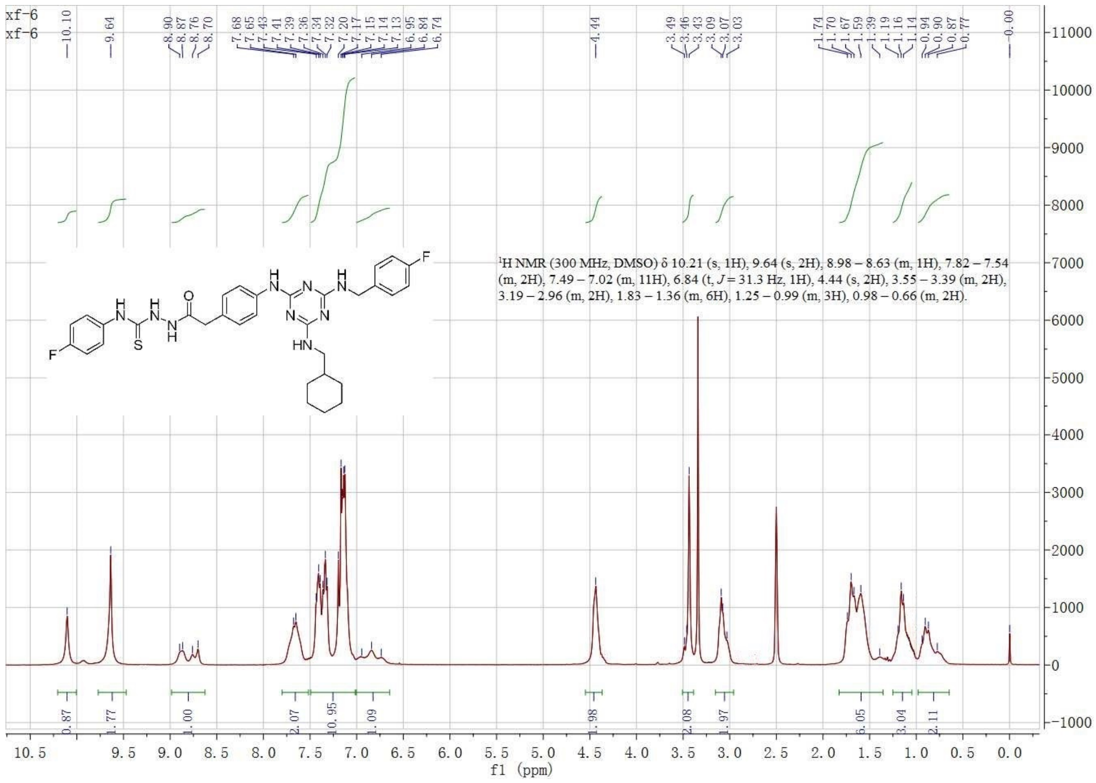

^{1}H-NMR of 10f

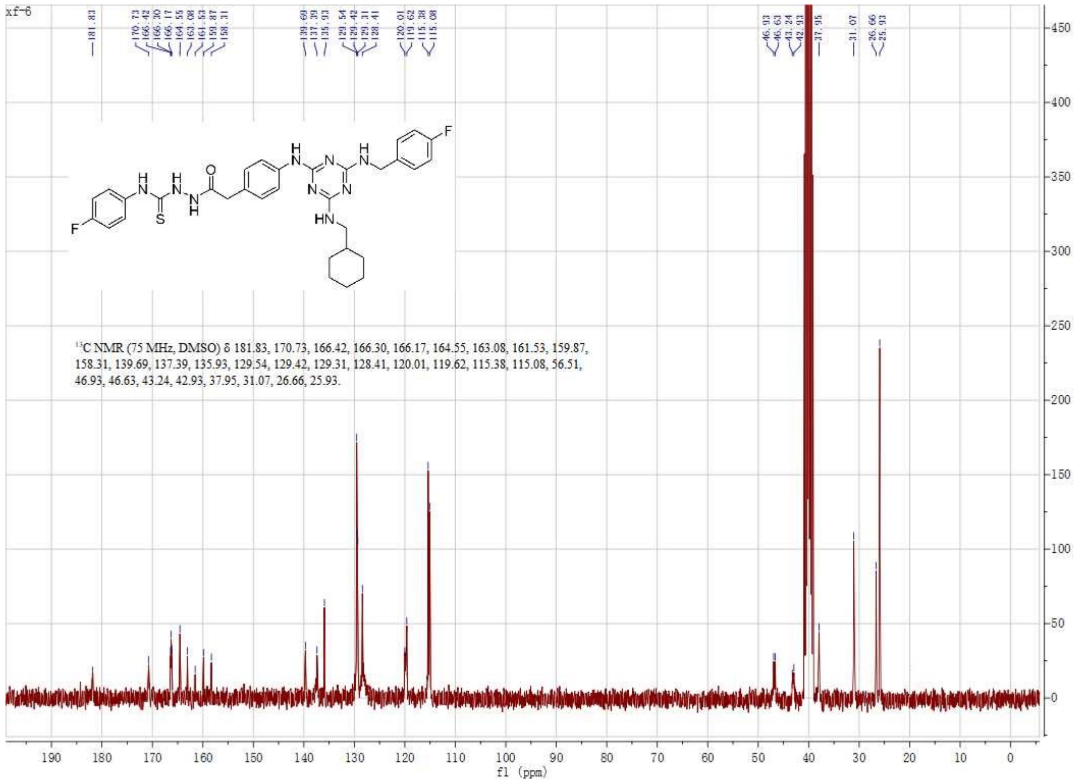

^{13}C-NMR of 10f

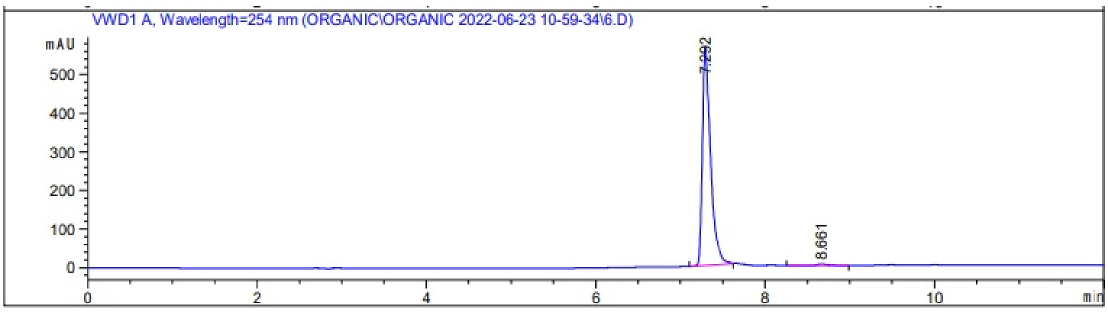

HPLC purity of 10f

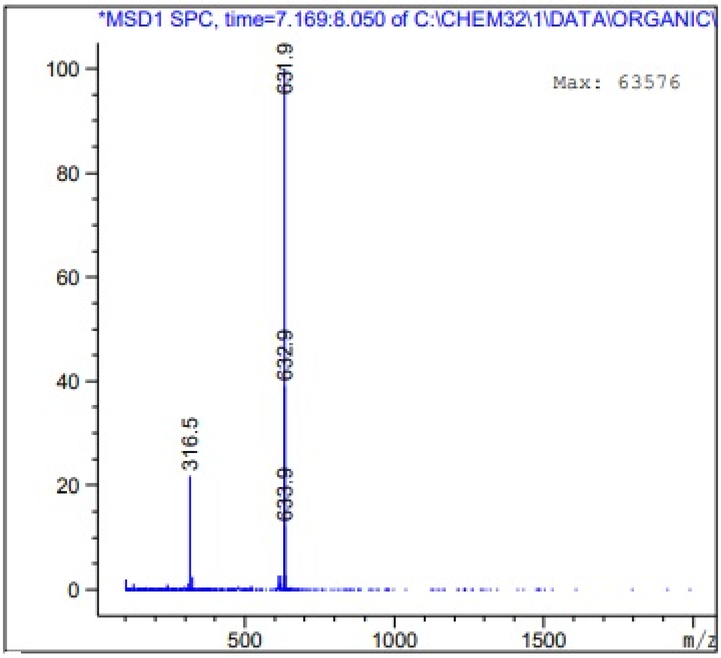

ESI-MS of 10f

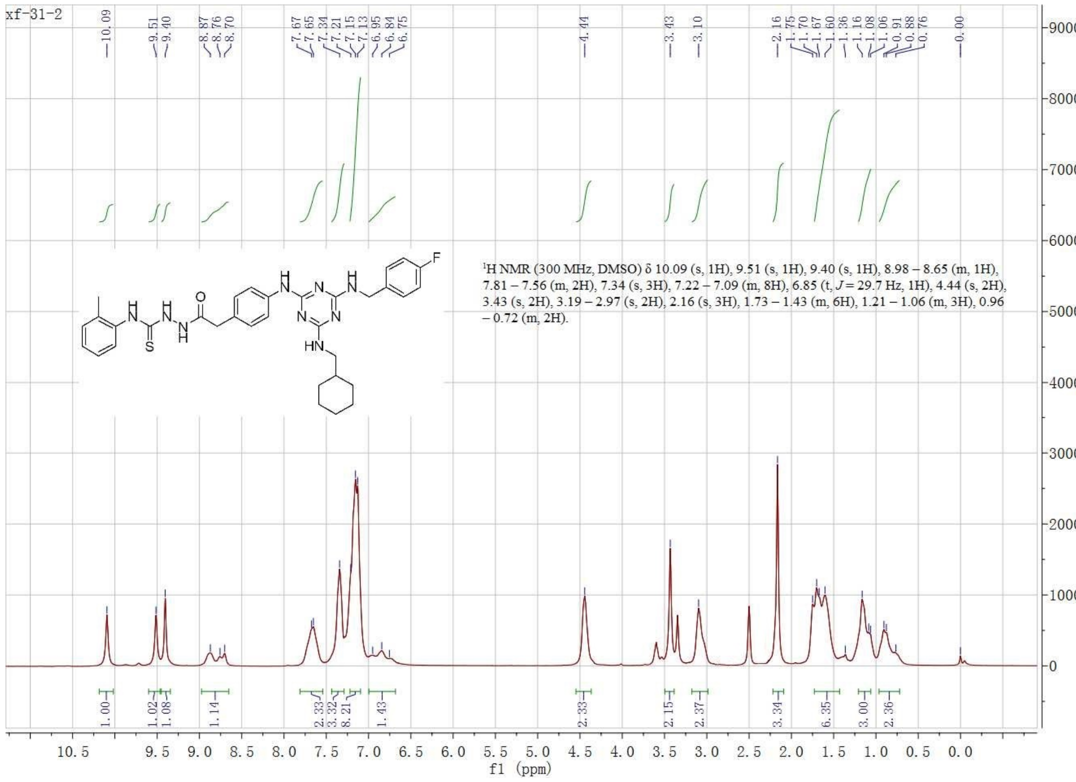

^{1}H-NMR of 10g

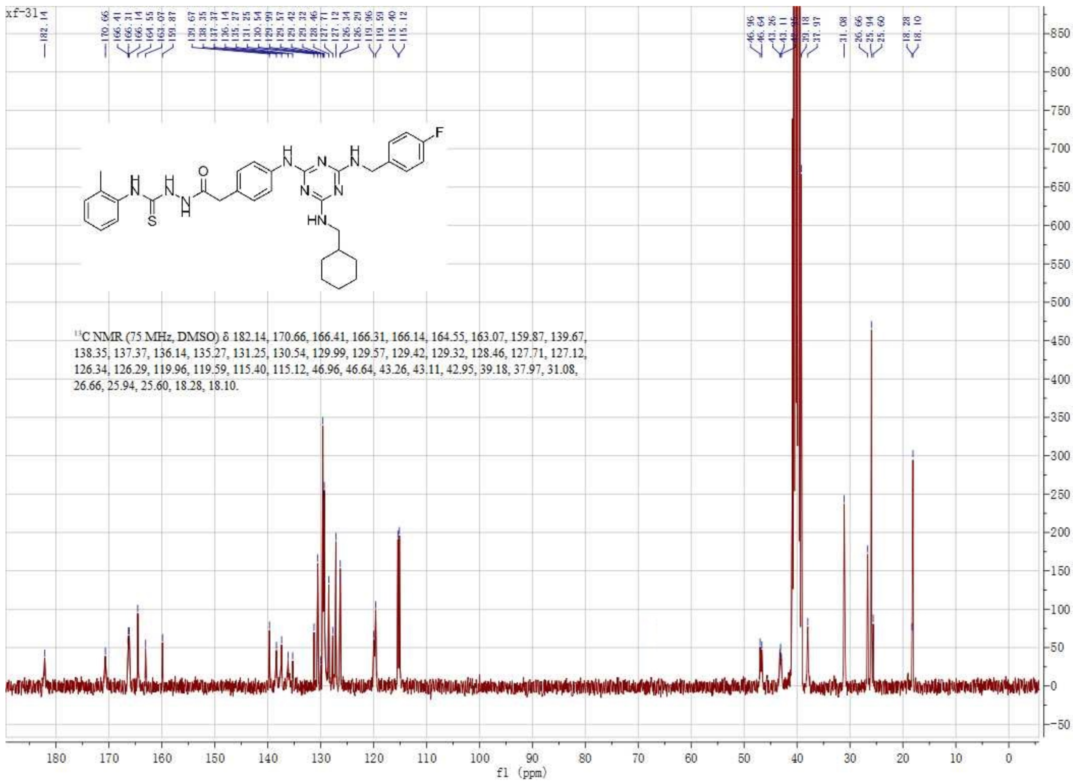

^{13}C-NMR of 10g

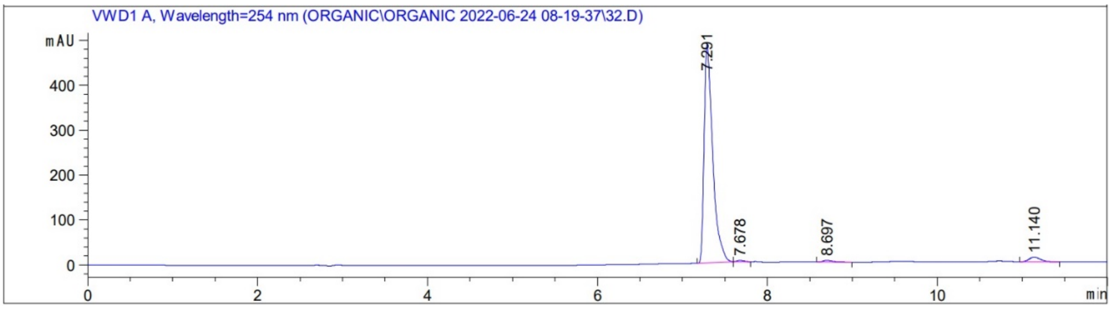

HPLC purity of 10g

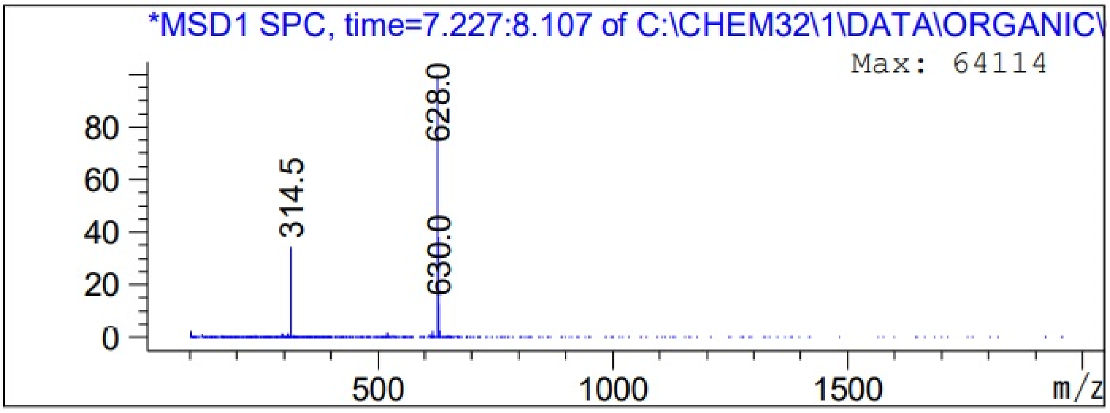

ESI-MS of 10g

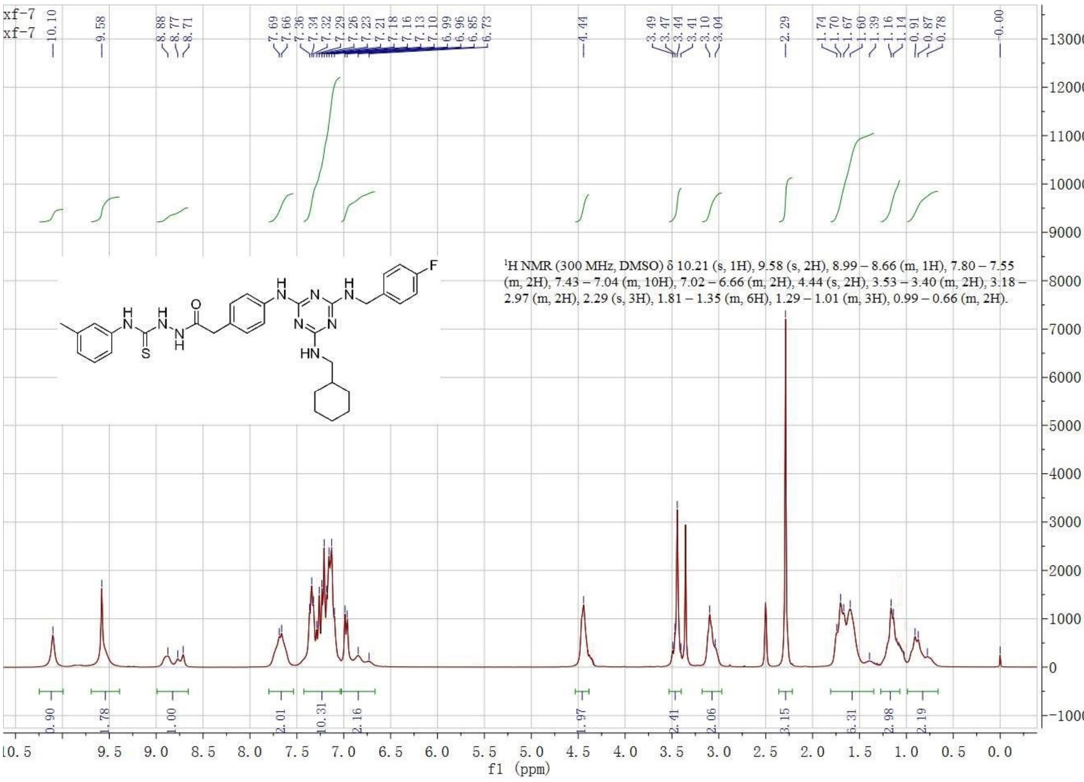

^{1}H-NMR of 10h

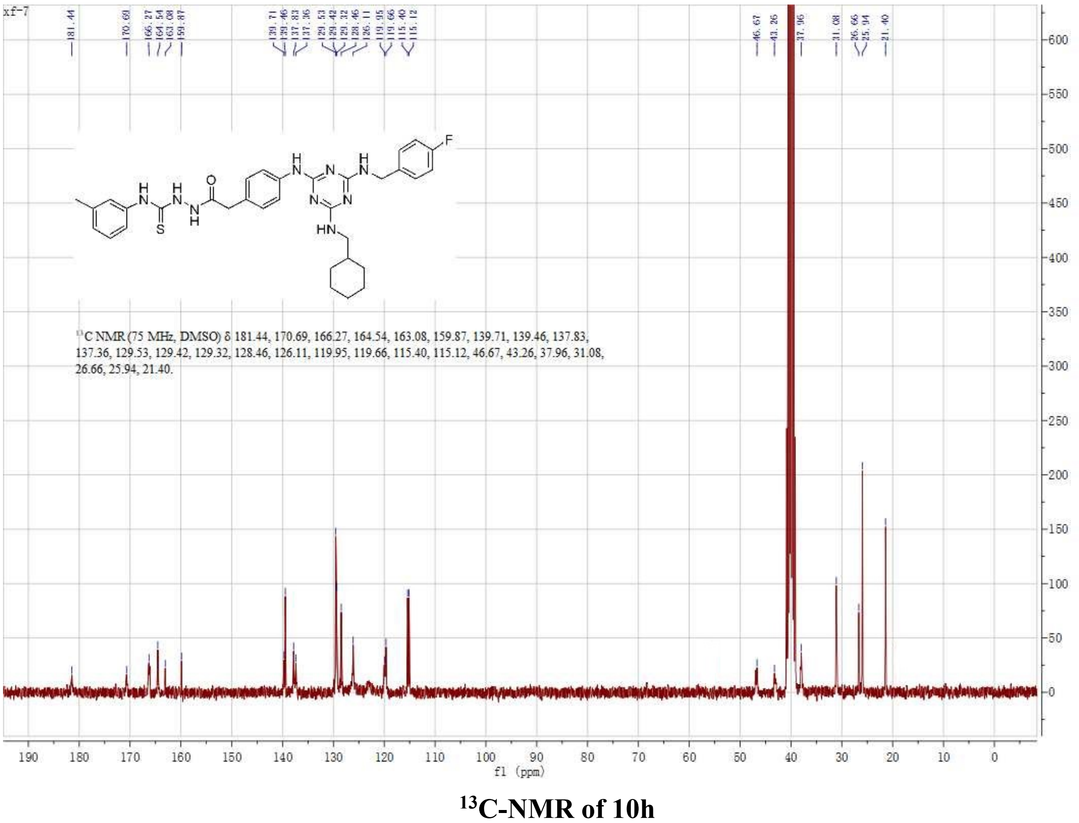

^{13}C-NMR of 10h

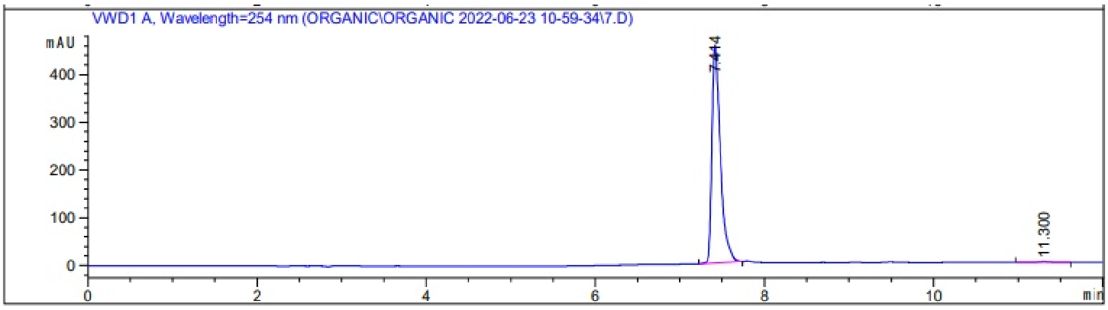

HPLC purity of 10h

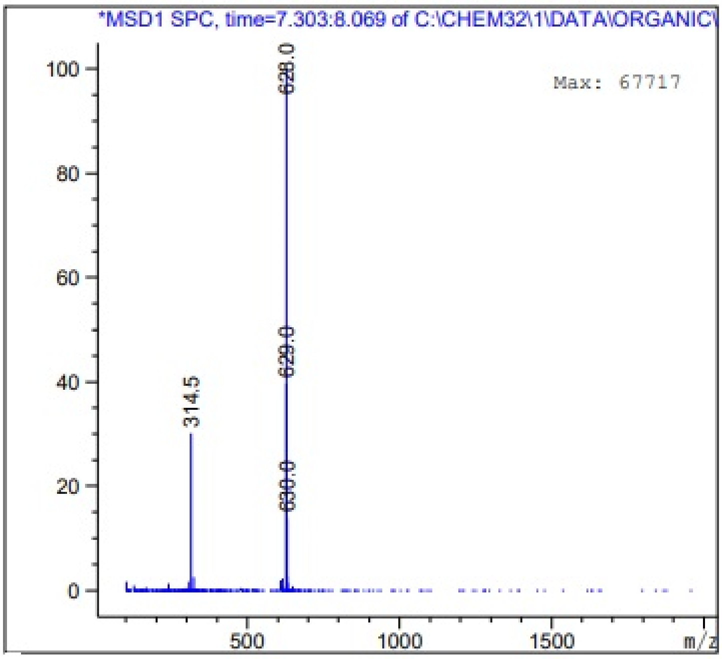

ESI-MS of 10h

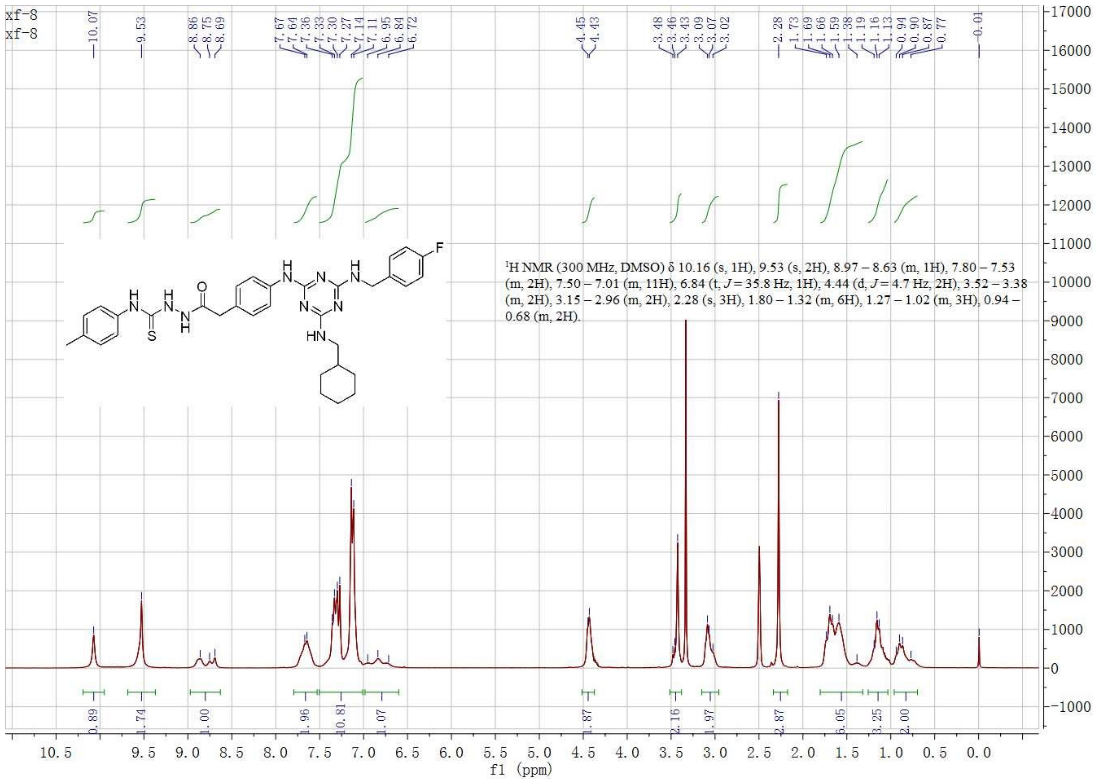

^{1}H-NMR of 10i

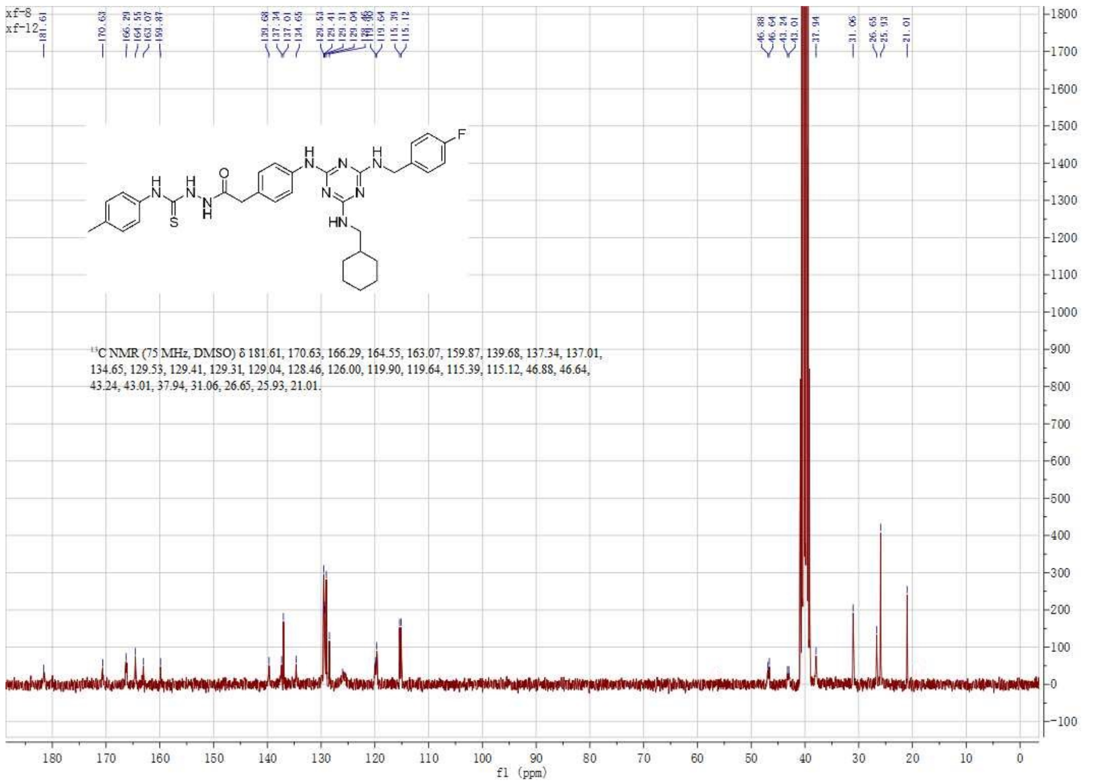

^{13}C-NMR of 10i

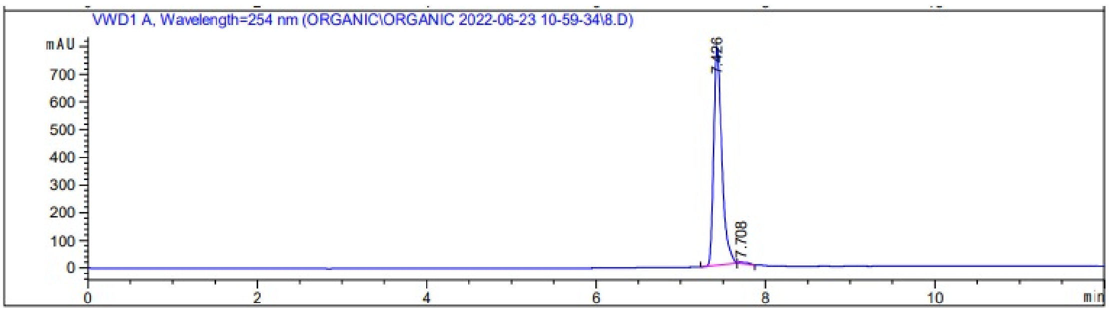

HPLC purity of 10i

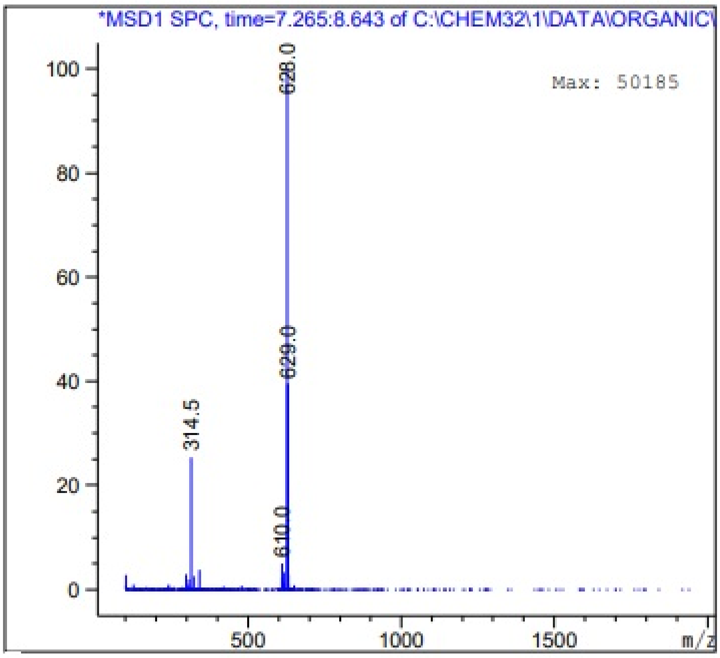

ESI-MS of 10i

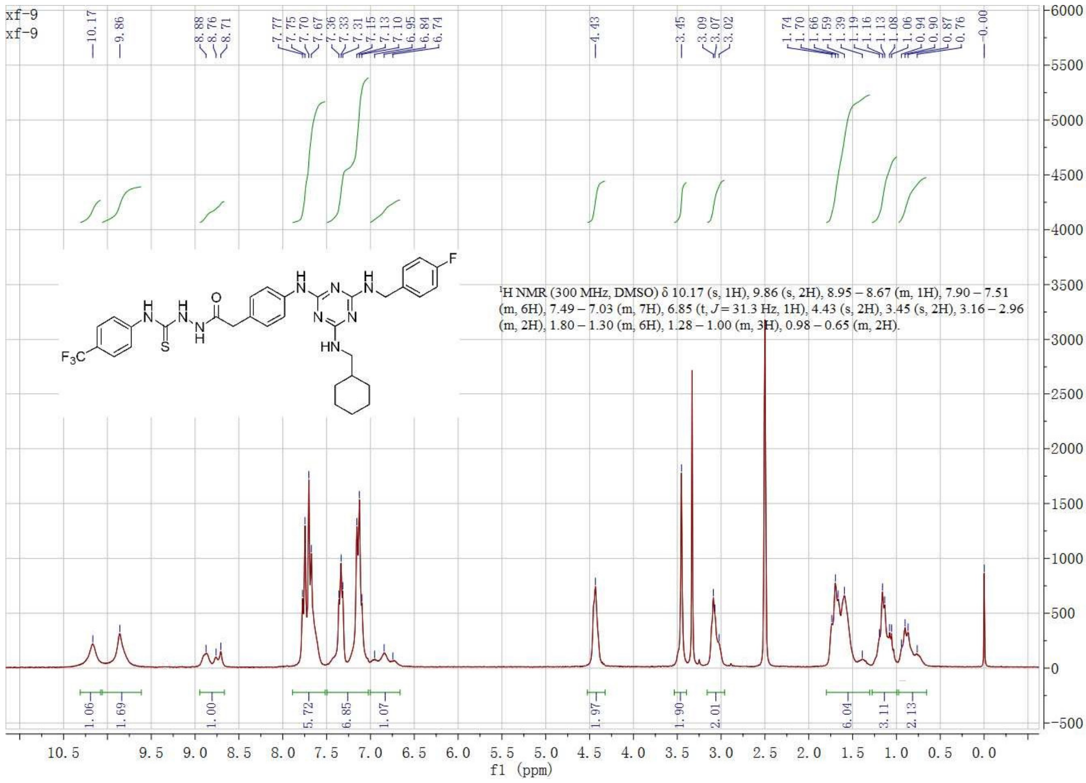

^{1}H-NMR of 10j

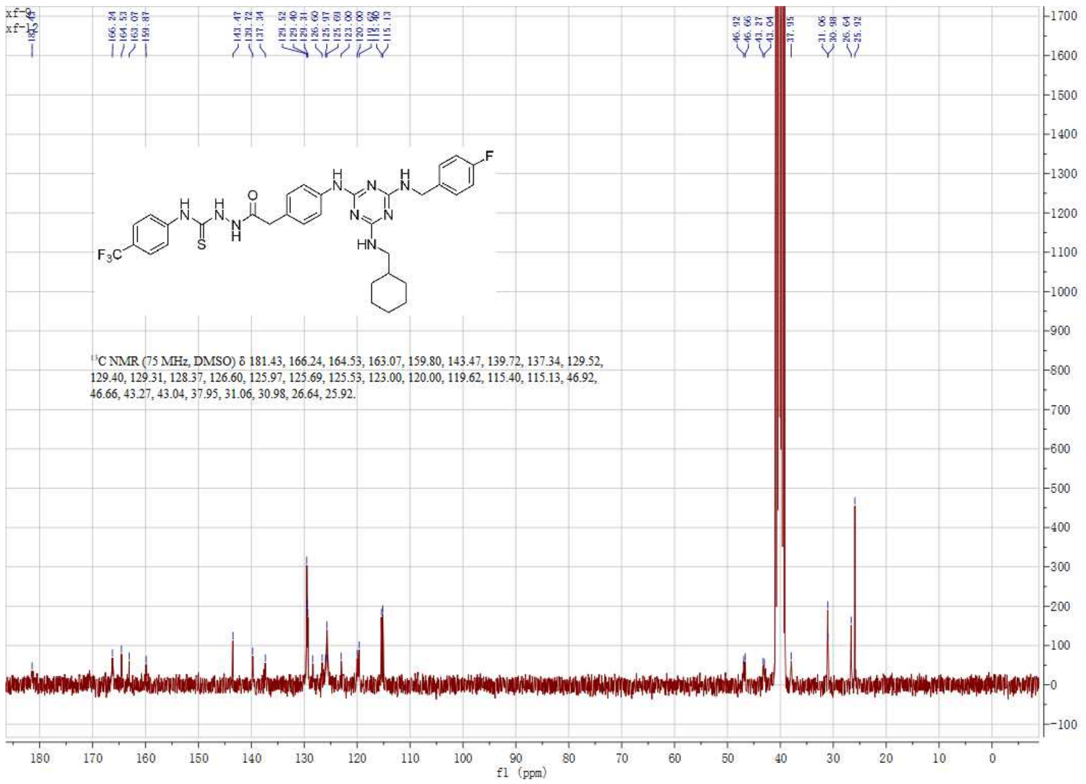

^{13}C-NMR of 10j

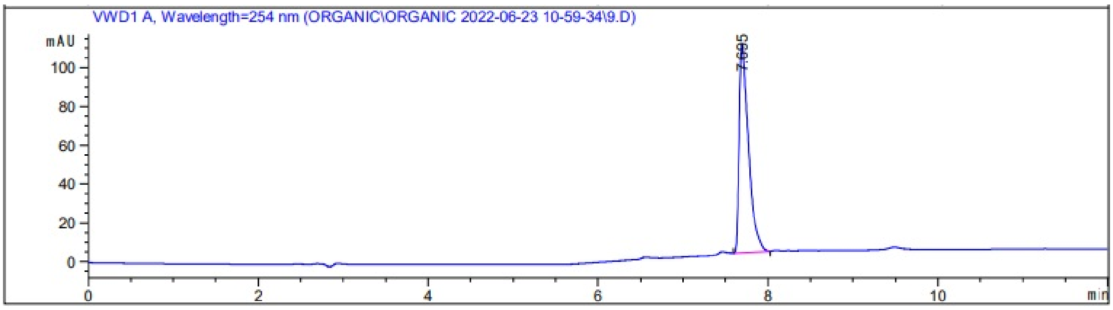

HPLC purity of 10j

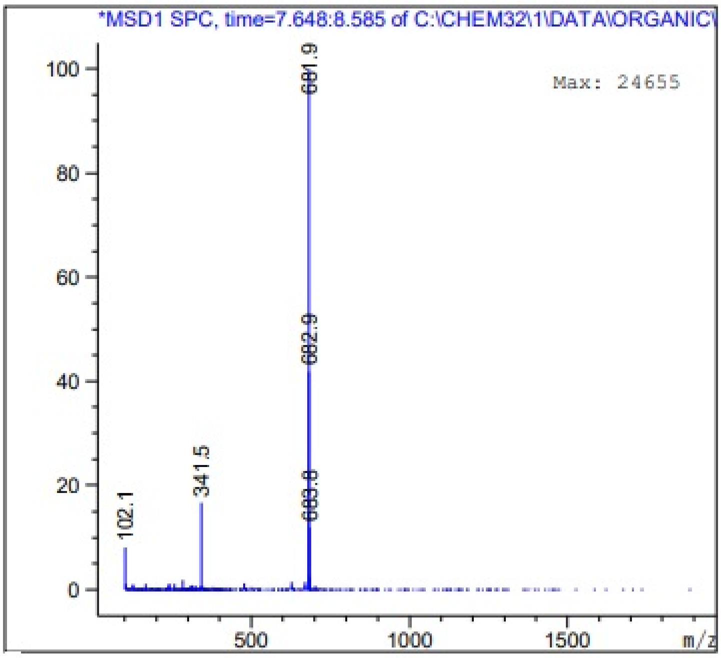

ESI-MS of 10j

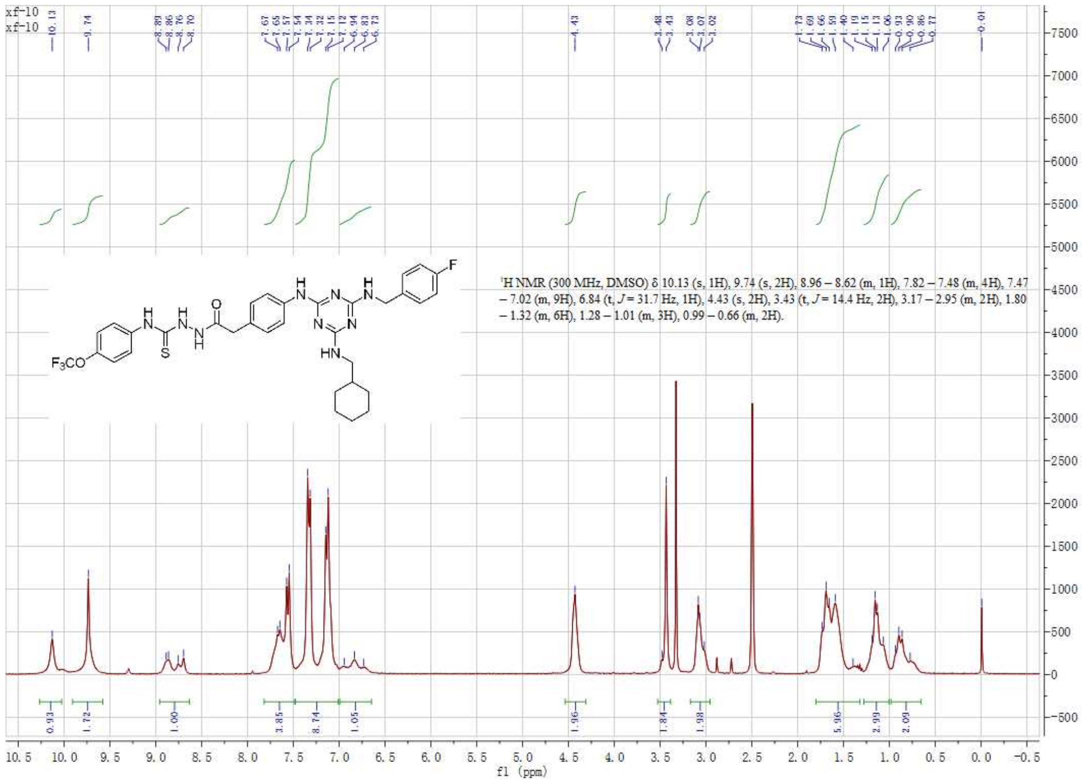

^{1}H-NMR of 10k

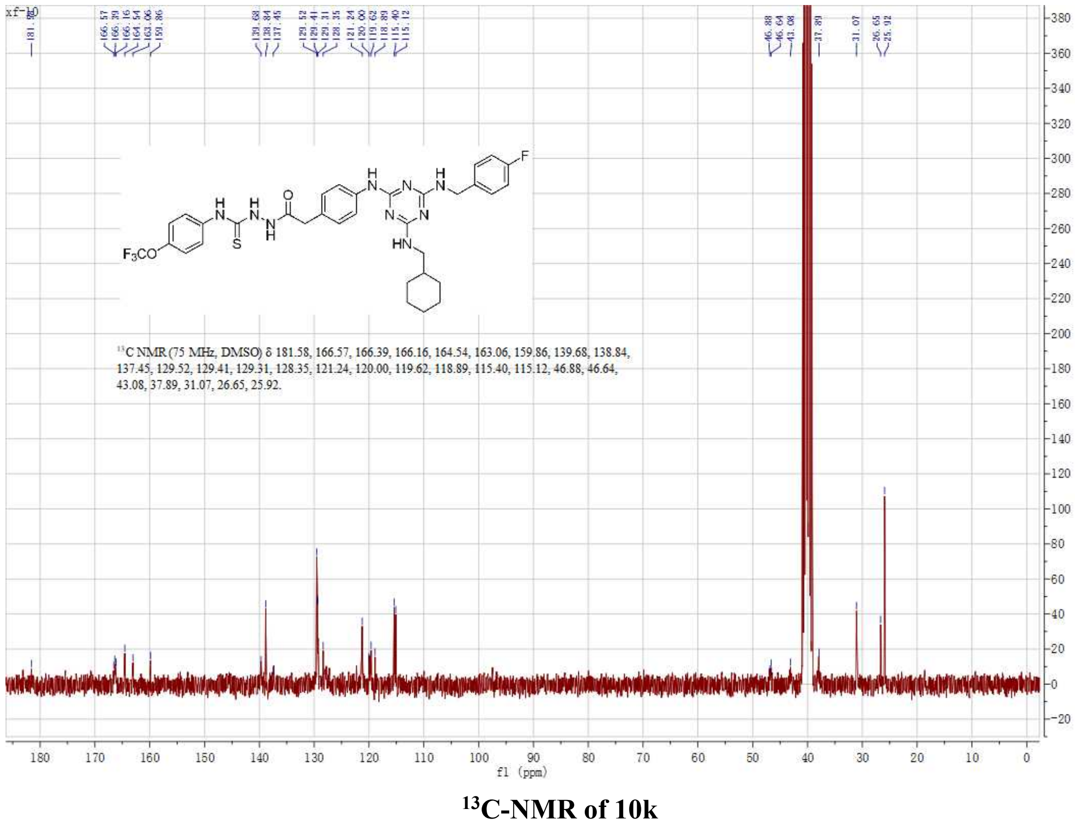

^{13}C-NMR of 10k

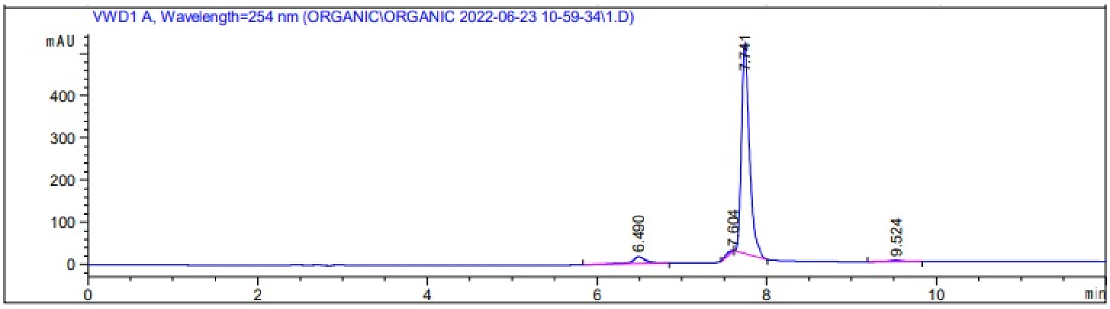

HPLC purity of 10k

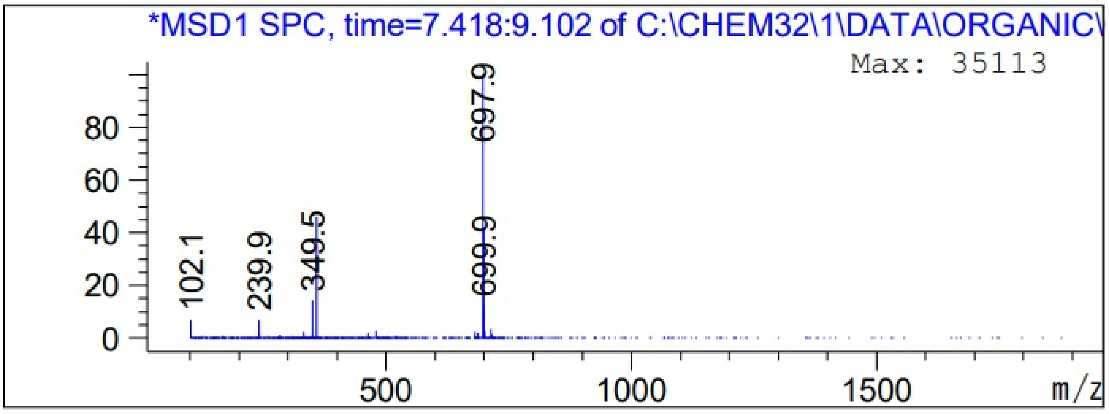

ESI-MS of 10k

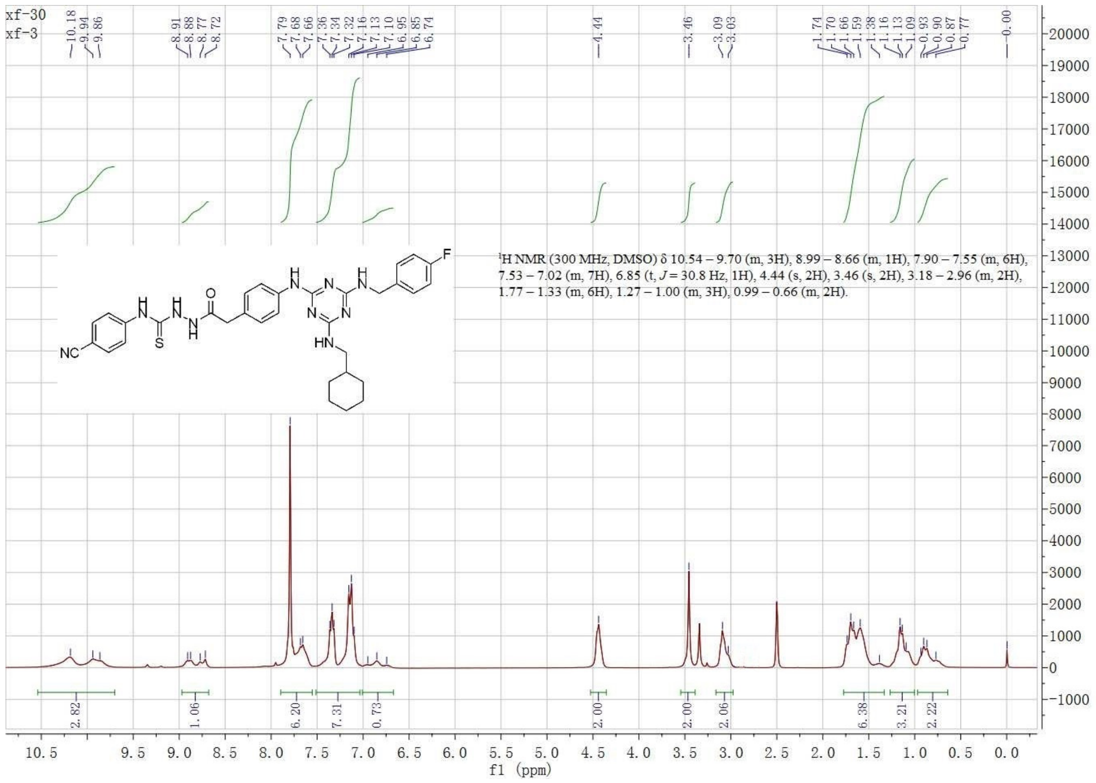

¹H-NMR of 10l

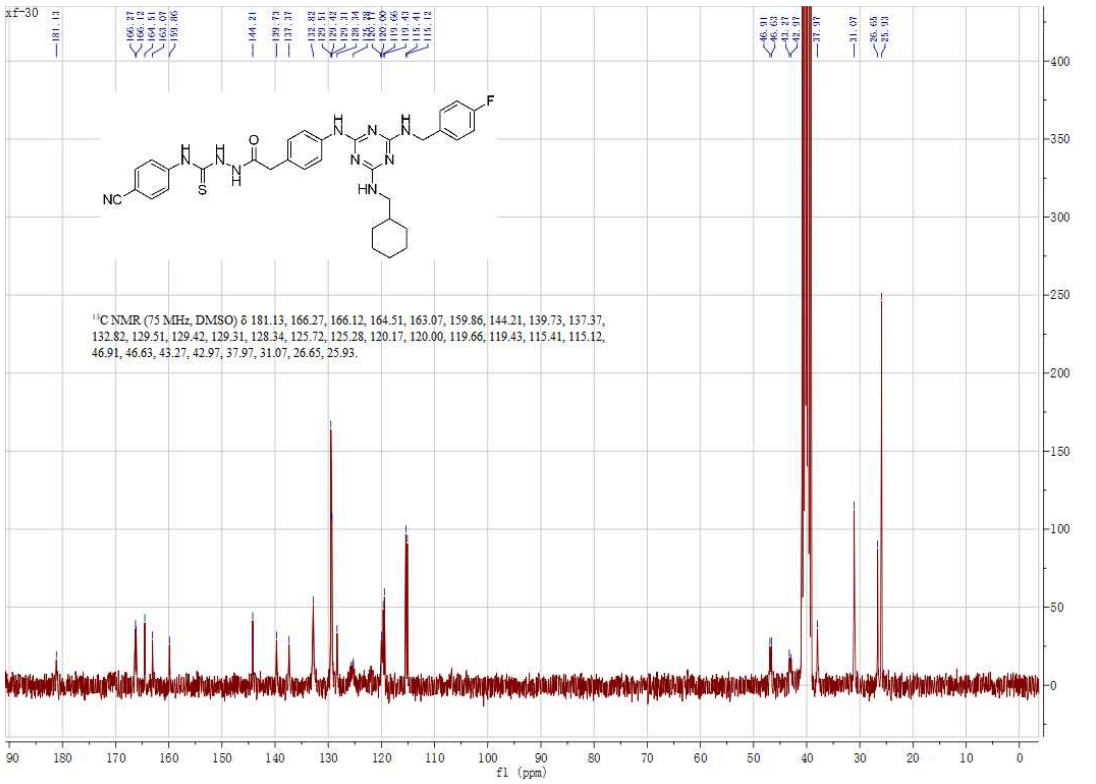

¹³C-NMR of 10l

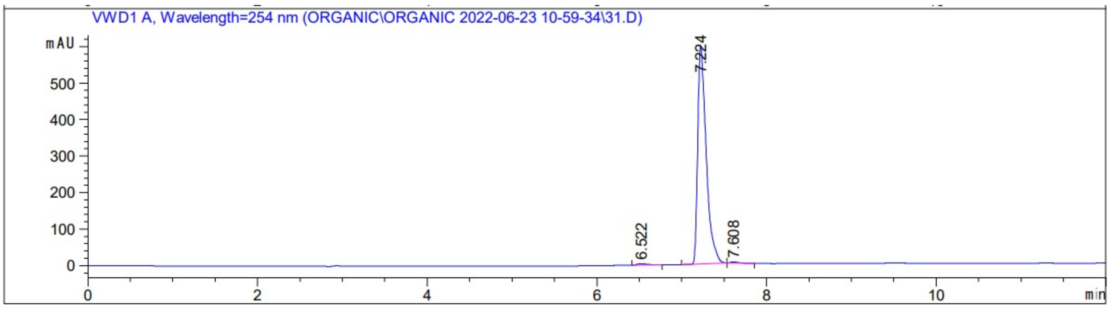

HPLC purity of 10l

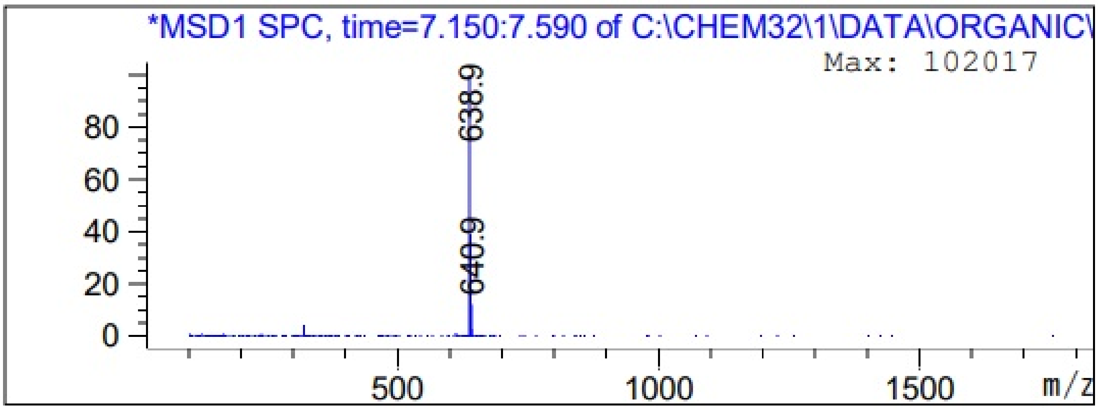

ESI-MS of 10l

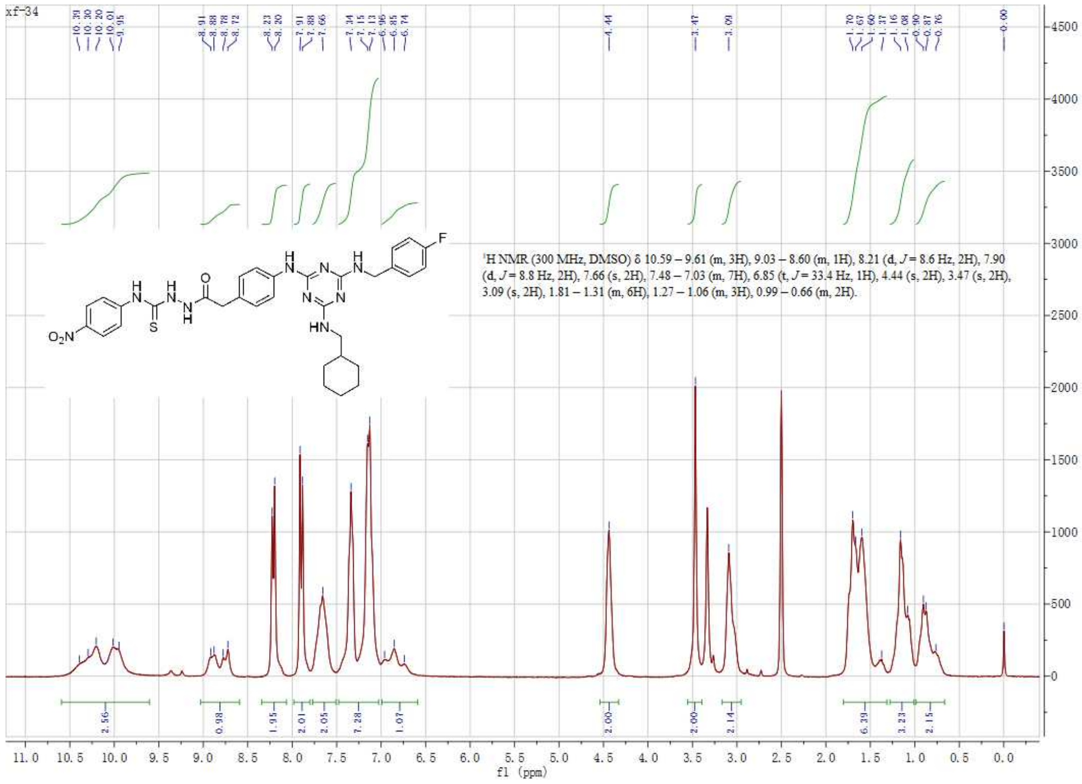

^{1}H-NMR of 10m

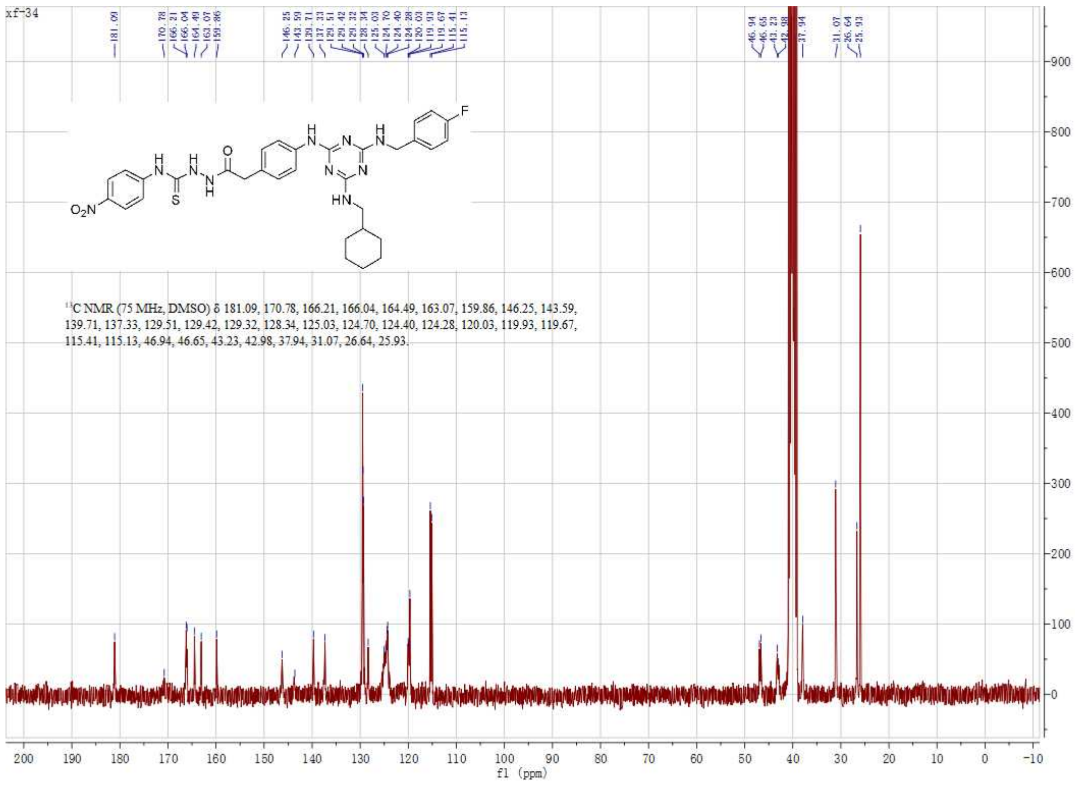

^{13}C-NMR of 10m

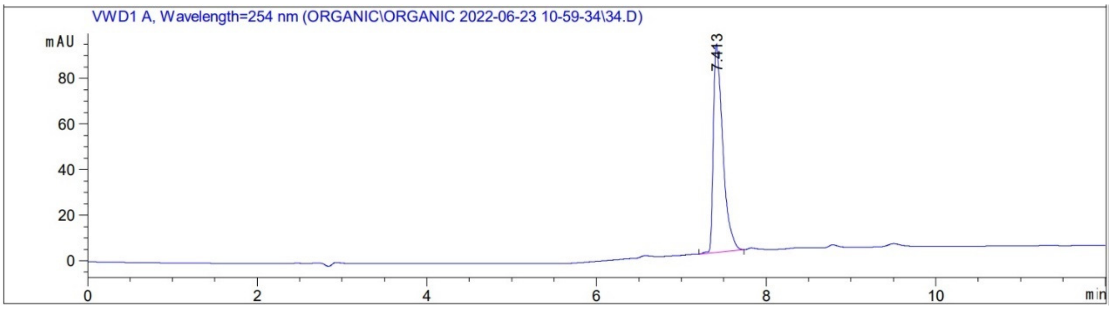

HPLC purity of 10m

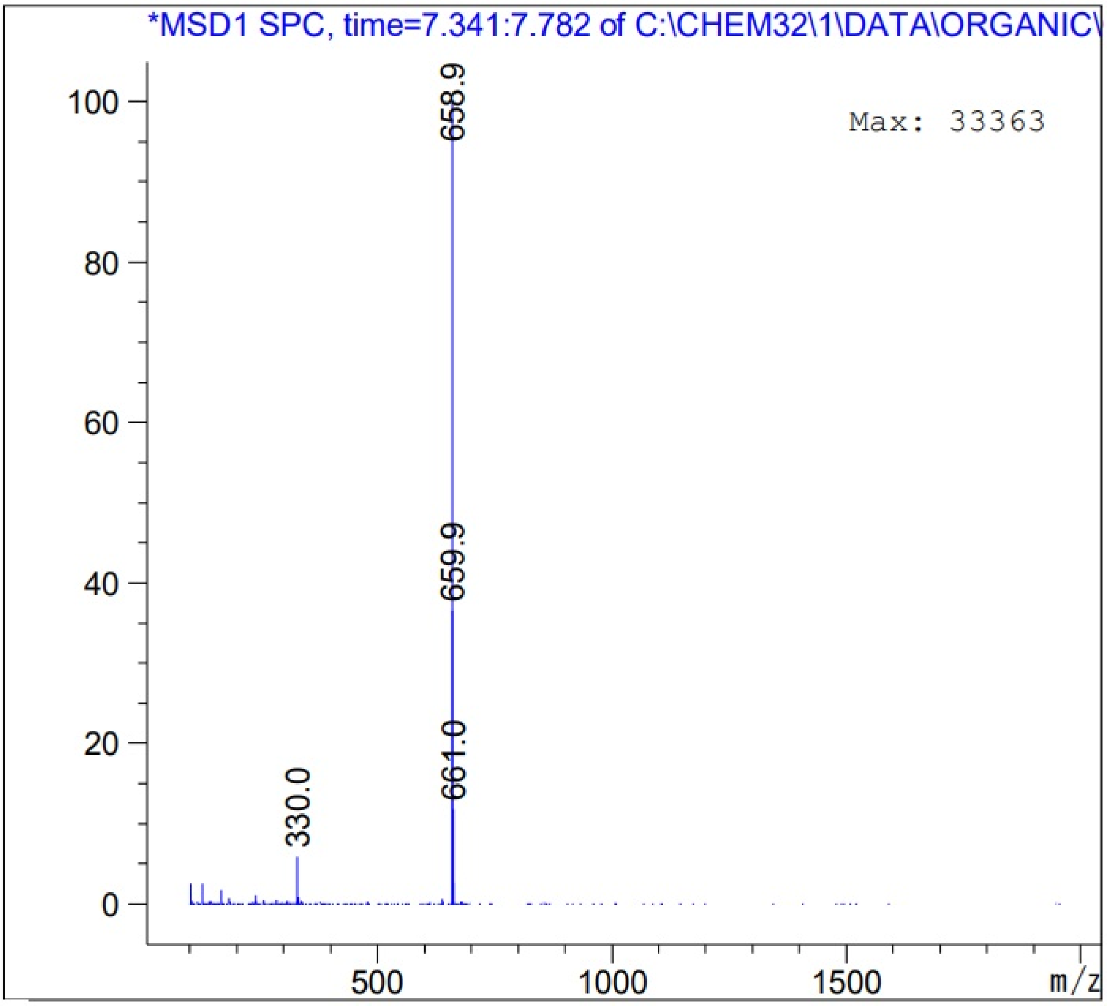

ESI-MS of 10m

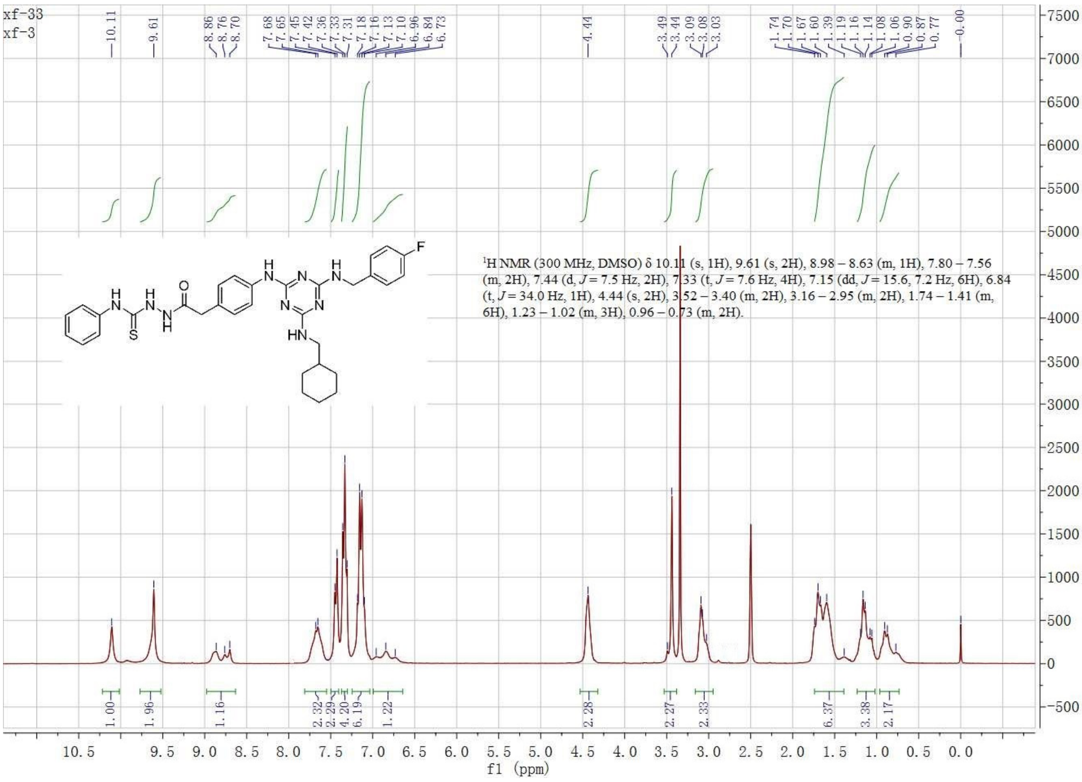

^{1}H-NMR of 10n

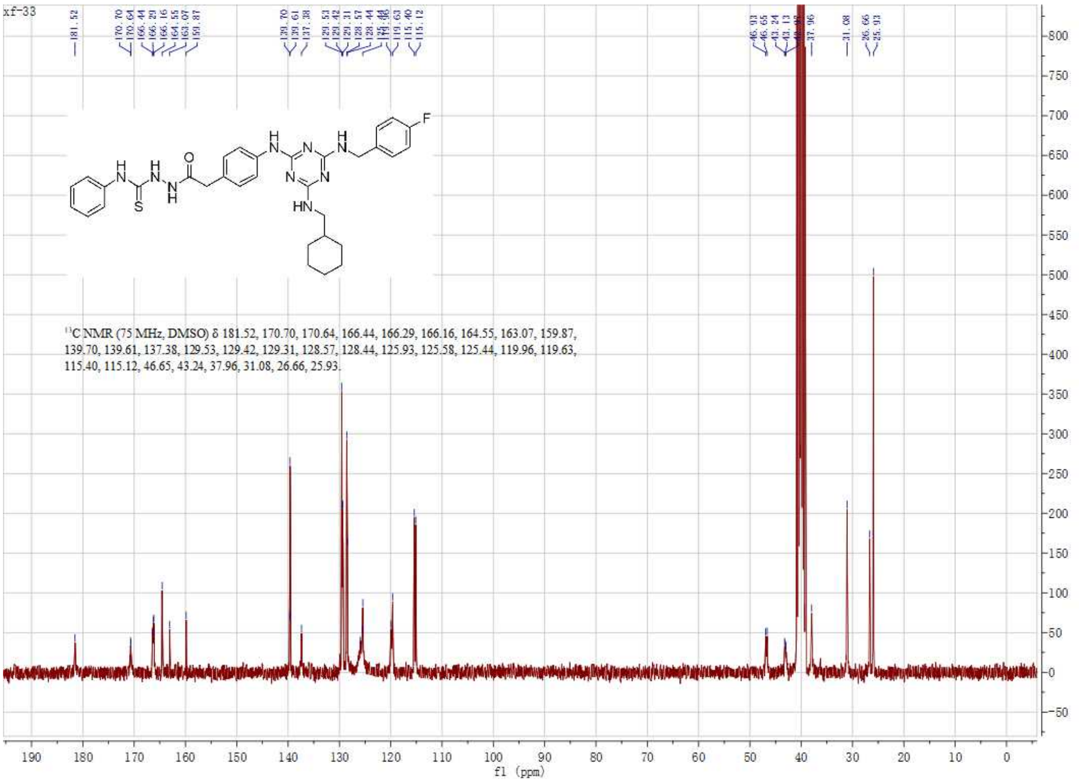

^{13}C-NMR of 10n

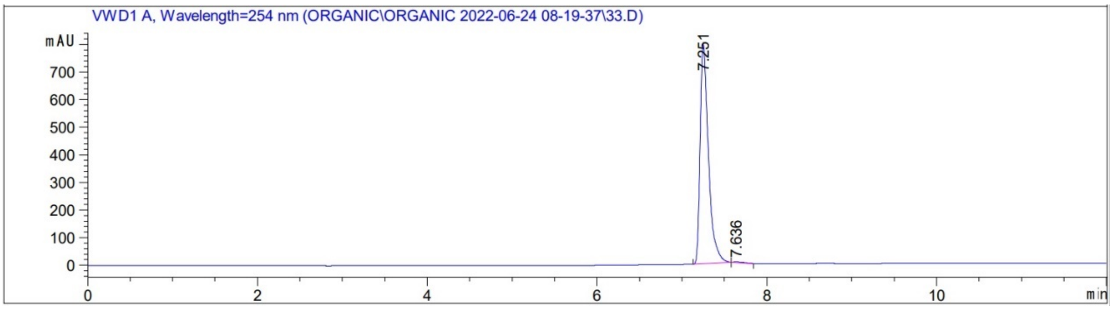

HPLC purity of 10n

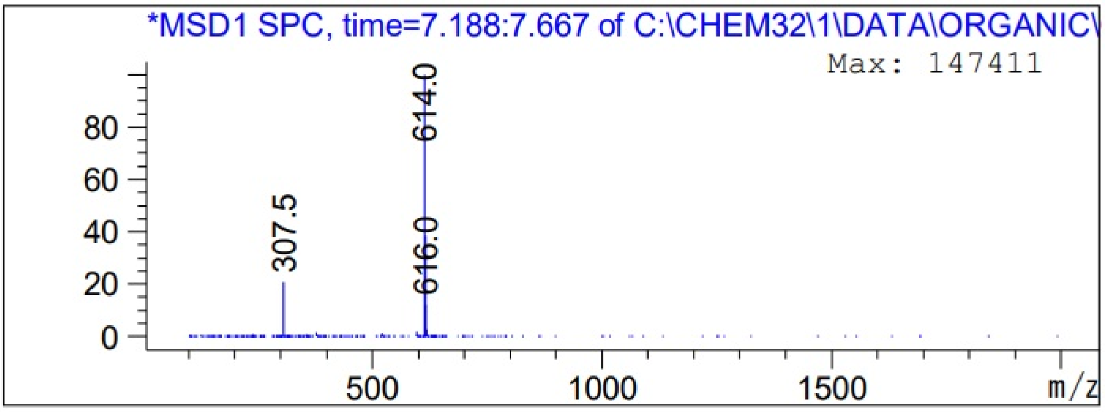

ESI-MS of 10n

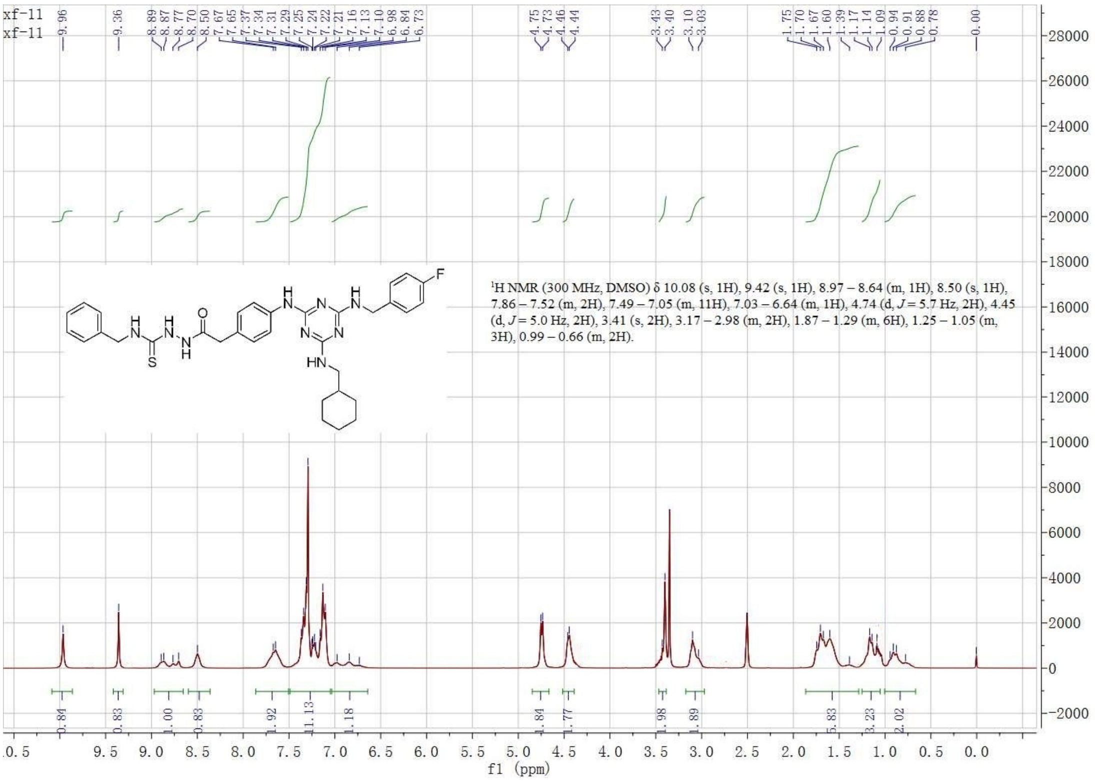

^{1}H-NMR of 10o

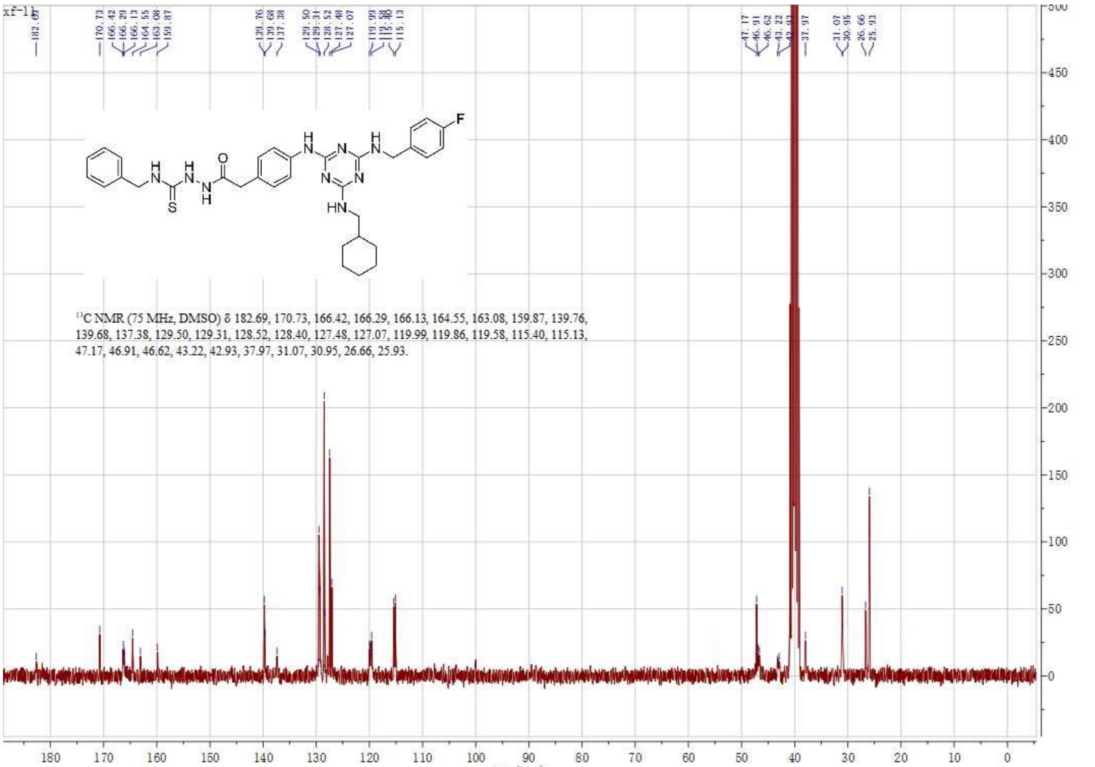

^{13}C-NMR of 10o

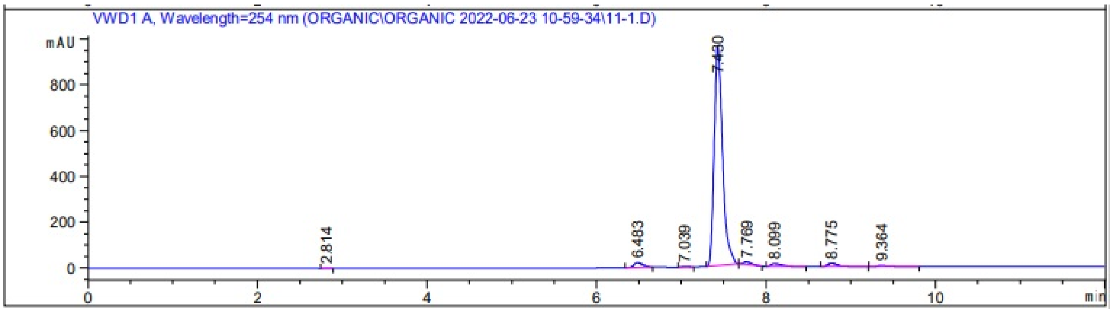

HPLC purity of 10o

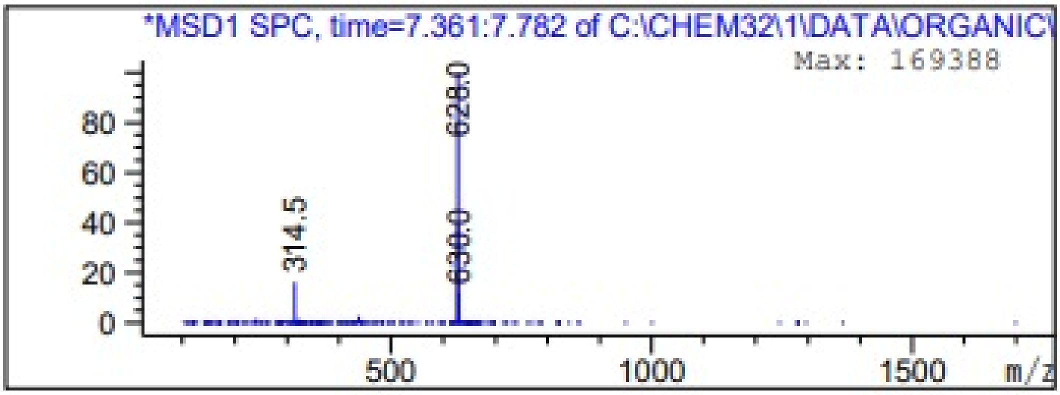

ESI-MS of 10o

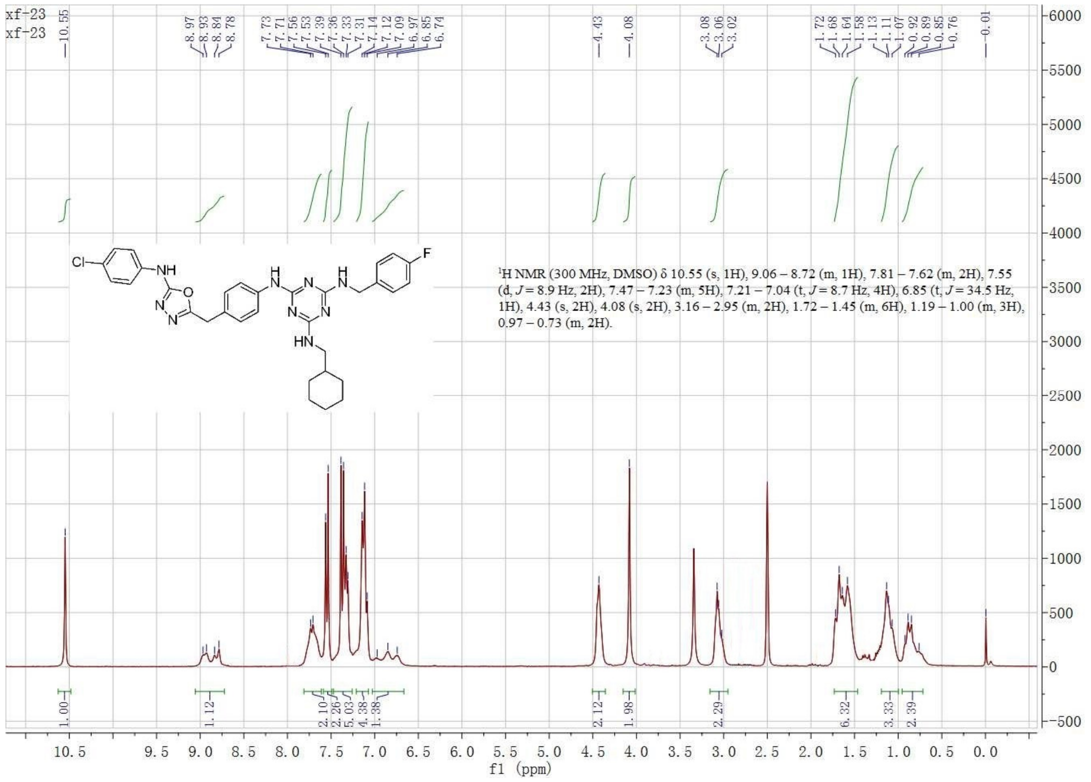

^{1}H-NMR of 11c

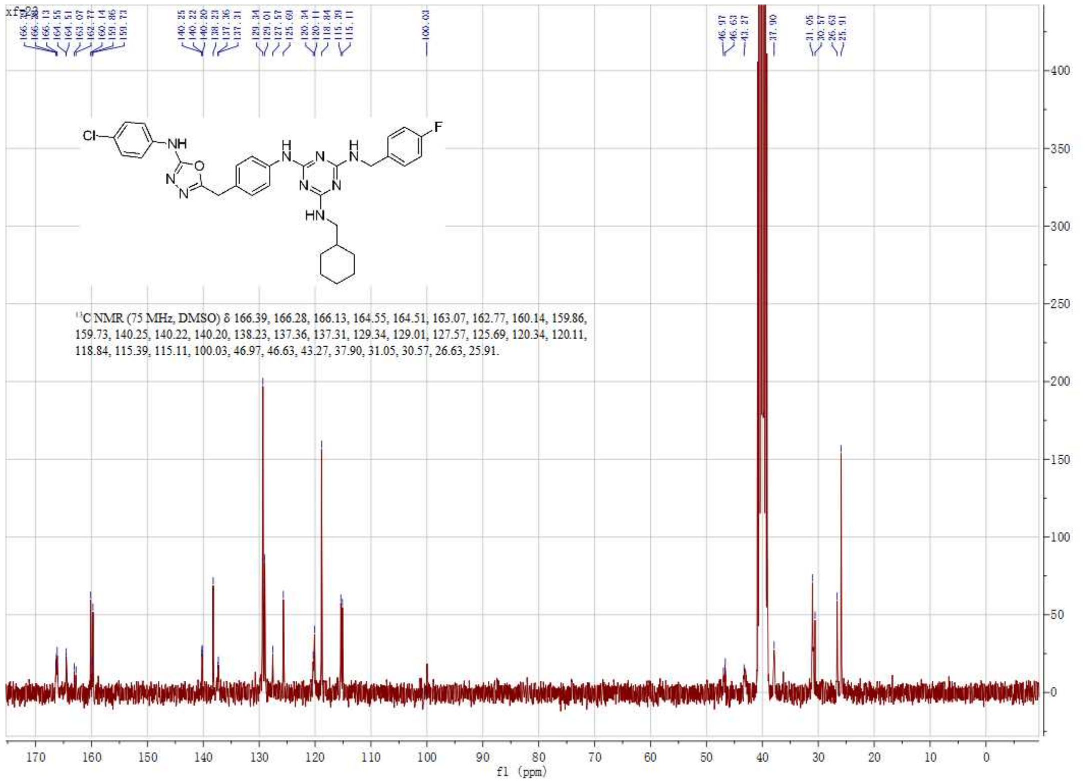

^{13}C-NMR of 11c

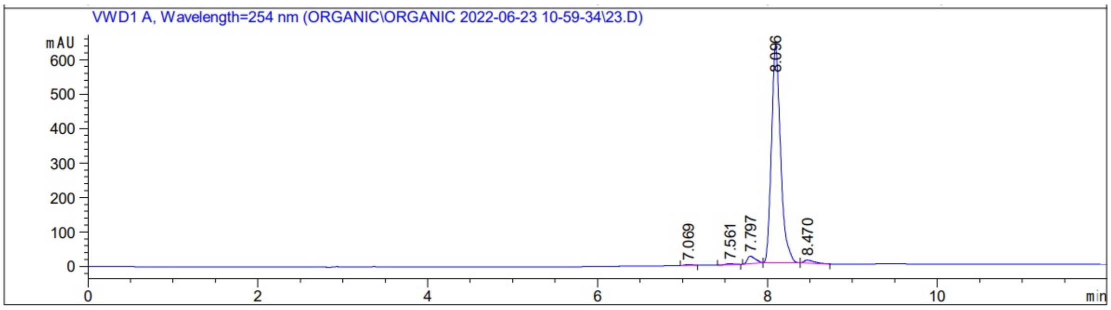

HPLC purity of 11c

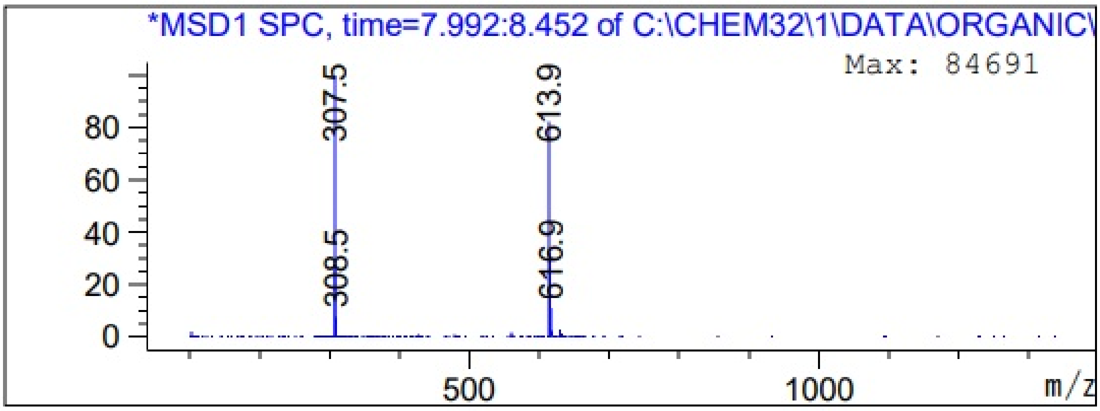

ESI-MS of 11c

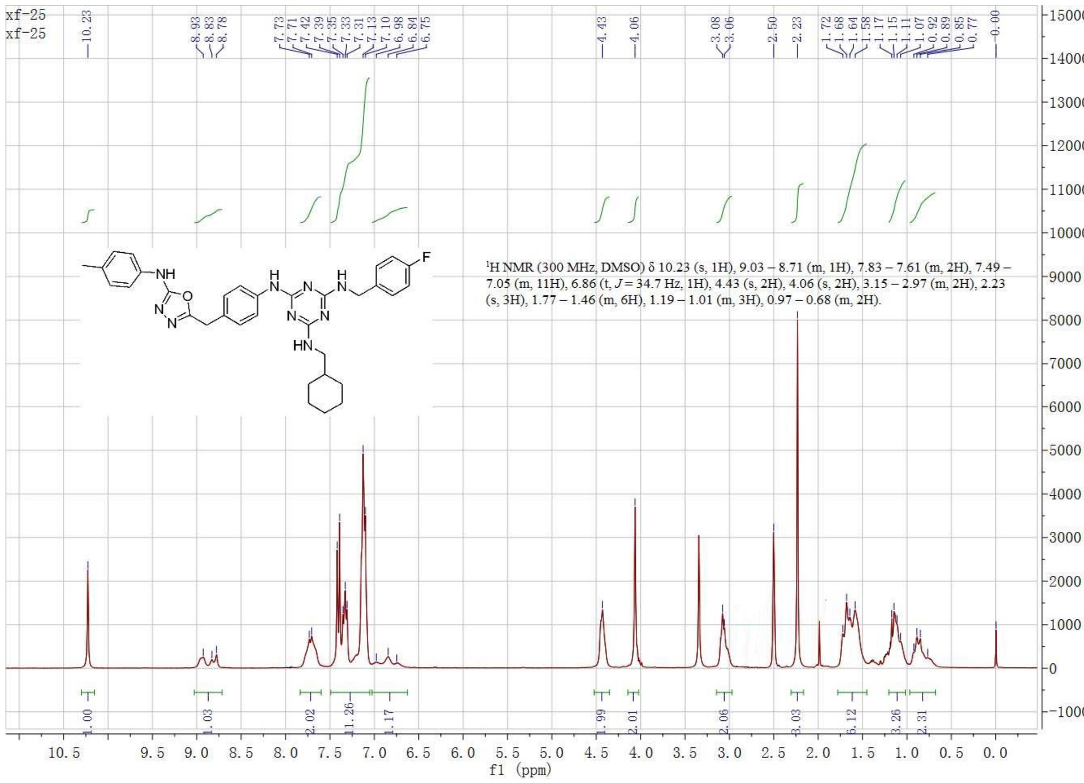

^{1}H-NMR of 11i

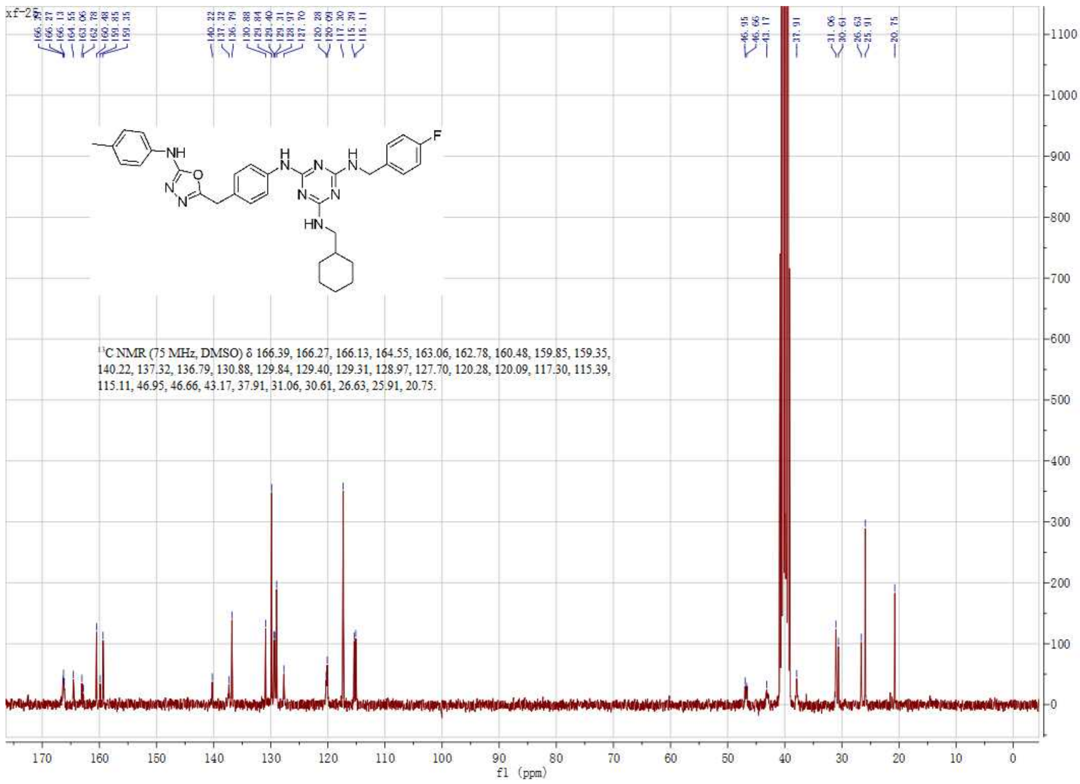

^{13}C-NMR of 11i

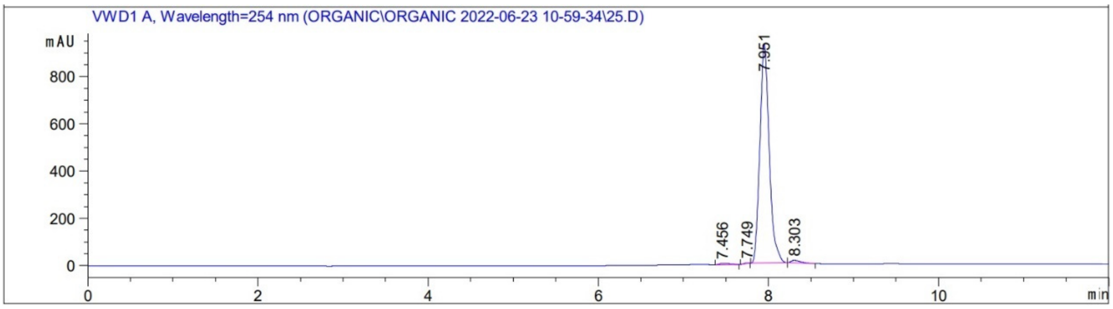

HPLC purity of 11i

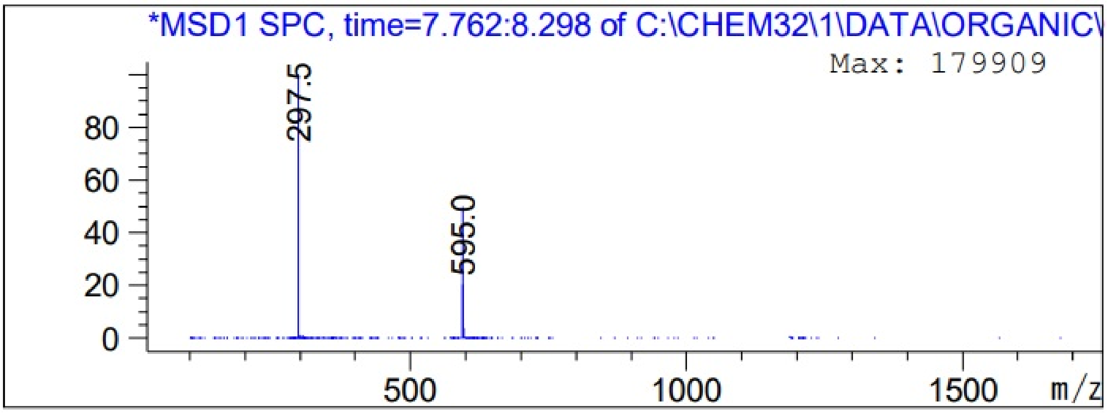

ESI-MS of 11i

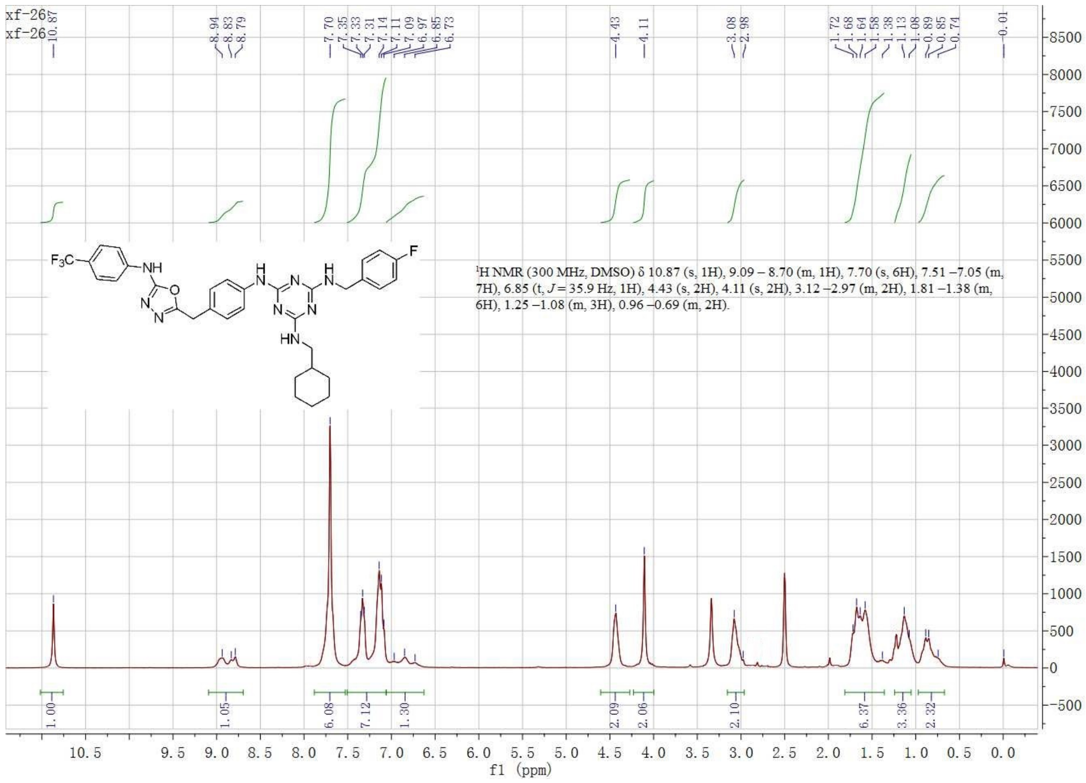

^{1}H-NMR of 11j

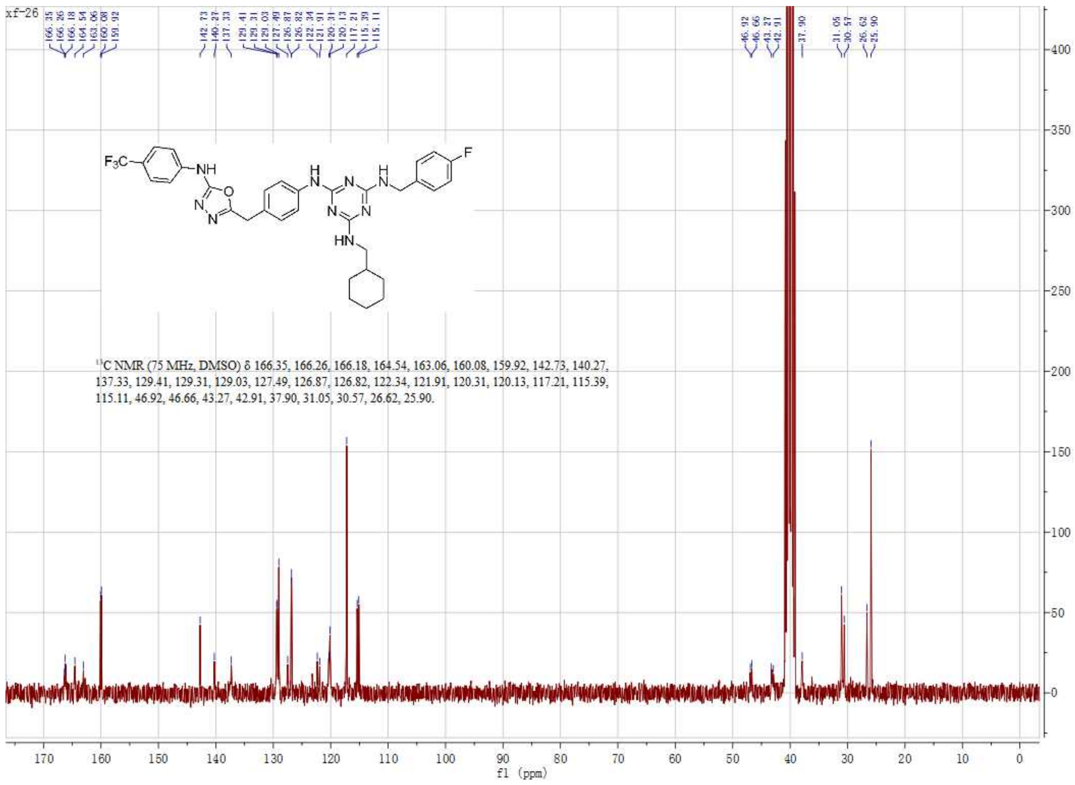

^{13}C-NMR of 11j

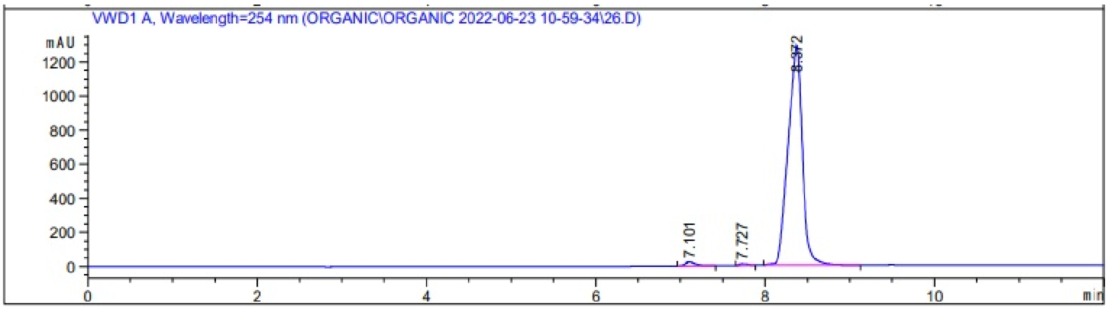

HPLC purity of 11j

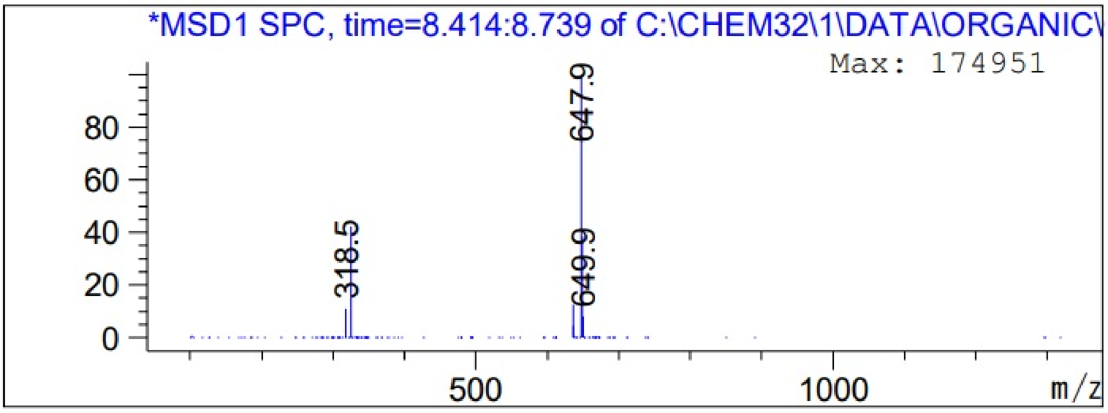

ESI-MS of 11j
